# Amplifying recombination genome-wide and reshaping crossover landscapes in *Brassicas*

**DOI:** 10.1371/journal.pgen.1006794

**Published:** 2017-05-11

**Authors:** Alexandre Pelé, Matthieu Falque, Gwenn Trotoux, Frédérique Eber, Sylvie Nègre, Marie Gilet, Virginie Huteau, Maryse Lodé, Thibaut Jousseaume, Sylvain Dechaumet, Jérôme Morice, Charles Poncet, Olivier Coriton, Olivier C. Martin, Mathieu Rousseau-Gueutin, Anne-Marie Chèvre

**Affiliations:** 1 IGEPP, INRA, Agrocampus Ouest, Université de Rennes 1, Le Rheu, France; 2 GQE-Le Moulon, INRA, Université Paris-Sud, CNRS, AgroParisTech, Université Paris-Saclay, Gif sur Yvette, France; 3 GDEC, INRA, Université de Clermont-Ferrand, France; John Innes Centre, UNITED KINGDOM

## Abstract

Meiotic recombination by crossovers (COs) is tightly regulated, limiting its key role in producing genetic diversity. However, while COs are usually restricted in number and not homogenously distributed along chromosomes, we show here how to disrupt these rules in *Brassica* species by using allotriploid hybrids (AAC, 2*n* = 3x = 29), resulting from the cross between the allotetraploid rapeseed (*B*. *napus*, AACC, 2*n* = 4x = 38) and one of its diploid progenitors (*B*. *rapa*, AA, 2*n* = 2x = 20). We produced mapping populations from different genotypes of both diploid AA and triploid AAC hybrids, used as female and/or as male. Each population revealed nearly 3,000 COs that we studied with SNP markers well distributed along the A genome (on average 1 SNP per 1.25 Mbp). Compared to the case of diploids, allotriploid hybrids showed 1.7 to 3.4 times more overall COs depending on the sex of meiosis and the genetic background. Most surprisingly, we found that such a rise was always associated with (i) dramatic changes in the shape of recombination landscapes and (ii) a strong decrease of CO interference. Hybrids carrying an additional C genome exhibited COs all along the A chromosomes, even in the vicinity of centromeres that are deprived of COs in diploids as well as in most studied species. Moreover, in male allotriploid hybrids we found that Class I COs are mostly responsible for the changes of CO rates, landscapes and interference. These results offer the opportunity for geneticists and plant breeders to dramatically enhance the generation of diversity in *Brassica* species by disrupting the linkage drag coming from limits on number and distribution of COs.

## Author summary

In organisms with sexual reproduction, meiosis generates gametes containing half of the genetic material of parents. During this process, the reciprocal exchanges between the homologous chromosomes due to crossovers (COs) ensure their proper segregation as well as the generation of diversity. However, the number of COs is limited and their location is heterogeneous along chromosomes. A major challenge is to overcome these constraints for enhancing the genetic shuffling of alleles. This work demonstrates that it is possible to do so in *Brassica* hybrids obtained by manual crossings, combining a complete set of homologous chromosomes and a haploid set provided by a related species. Specifically, by studying large segregating populations, we find that in allotriploid *Brassica* hybrids, more COs are formed all along the homologous chromosomes, especially in regions usually deprived of COs, compared to diploids. These results offer the opportunity for geneticists and plant breeders to dramatically enhance the generation of new diversity.

## Introduction

Meiotic recombination through crossovers (COs) is the key mechanism ensuring both the proper segregation of homologous chromosomes during meiosis and the generation of diversity in all sexual organisms. Indeed, following the formation of DNA Double Strand Breaks (DSBs), during the Prophase I of meiosis, their repair leading to COs allows reciprocal exchanges between homologous non-sister chromatids generating new allelic combinations in gametes [1, 2].

Because of strict regulation of recombination, modification of CO rate and positions along chromosomes is a key challenge for enhancing the genetic shuffling of diversity [3]. First, in most organisms and especially in plants, a low proportion of DSBs is repaired into COs [3, 4]. For example, in *Arabidopsis thaliana*, of the 150 to 250 DSBs generated per meiosis, on average only ~11.5 are repaired in the form of COs, the others giving rise to Non-Crossovers (NCOs) or possibly COs between sister chromatids [3, 5–7]. Several proteins were recently highlighted to promote the repair of DSBs into NCOs in *A*. *thaliana* (e.g. FANCM, RECQ4, FIGL1) [8–11], thereby limiting the overall number of COs formed in a meiosis. Furthermore, per pair of homologs, one obligate CO occurs for ensuring their proper segregation during Anaphase I [12, 13], but rarely more than three are observed due to the so-called phenomenon of CO interference [3, 14, 15]. Indeed, two adjacent COs on a chromosome are rarely very close to each other, resulting in less variability in the distances between adjacent COs than would arise from a random distribution [16, 17]. Among the two Classes of COs known to be produced, only the Class I is subject to significant CO interference; that Class depends on ZMM complex in addition to MLH1 and MLH3 proteins. The Class II COs, catalyzed by MUS81 and EME1/MMS4 proteins, seems unaffected by CO interference but contributes only marginally in plants (e.g. ~15% of all COs in *A*. *thaliana*) [3, 18, 19]. Second, the CO landscape is not homogenous along the chromosomes, at any scale in almost all species [3, 20]. For instance, 80% of COs are observed within less than 26% of the *A*. *thaliana* genome while only 13% of the 3B chromosome of *Triticum aestivum* showed COs [21, 22]. Locally, most COs cluster in genomic regions of a few kilobases called recombination hotspots [20, 23]. Recent advances in the characterization of recombination hotspots have pointed out links with genomic and epigenetic features in *A*. *thaliana*, revealing that COs preferentially occur close to gene promoters and terminators associated to an open chromatin pattern [22, 24–29]. At larger scales, the frequency of COs varies along the arms of chromosomes while the centromeric regions are entirely devoid of COs in almost all species [3, 30, 31]. Furthermore, a particular pattern for the COs distribution is observed in some plants (e.g. *Triticum turgidum*, *Triticum aestivum* and *Zea mays*), with a gradual increase of the COs frequency away from centromeres [32–34]. These last observations could be in link with different features of genome architecture such as content in genes and transposable elements (TEs). Indeed, genes are mostly located on chromosomal extremities while TEs preferentially concentrate in the vicinity of centromeres [35]. Consequently, in different plants, COs frequencies were found to positively correlate with density in genes and negatively with TE density (e.g. *T*. *aestivum*, *Z*. *mays* and *Oriza sativa*) [36–39].

Apart from the use of knock-out mutants, different factors have been related to the regulation of recombination, including environmental conditions (e.g. abiotic stress, temperature) [40, 41], sex of meiosis [41–43] or genotype [44], but the most marked variations of CO frequencies were linked to the ploidy level. The number of COs per chromosome can be higher in polyploids, which present multiple sets of homologous (autopolyploids) or homoeologous (allopolyploids) chromosomes, than in diploids as exemplified in *Arabidopsis* [45], *Gossypium* [46], *Zea* [47] or *Brassica* [48]. For instance, genetic mapping of *Brassica napus* allotetraploids (AACC, 2*n* = 4x = 38), which results from the natural hybridization between *B*. *rapa* (AA, 2*n* = 2x = 20) and *B*. *oleracea* (CC, 2*n* = 2x = 18) [49, 50], showed about twice as many COs between the homologous A07 chromosomes than in the diploid AA hybrids [48]. Similarly, in viable triploids, which exhibit a complete set of chromosomes at diploid stage and another one at haploid stage, homologous recombination frequencies also increase compared to those in the diploids as exemplified in triploids resulting from *Lolium multiflorum* (4x) x *L*. *perenne* (2x) [51], *L*. *multiflorum* (4x) x *Festuca pratensis* (2x) [52] or *B*. *napus* (4x) x *B*. *rapa* (2x) [48]. Surprisingly, in *Brassica* allotriploid hybrids (AAC, 2*n* = 3x = 29), the number of COs between the homologous A07 chromosomes was described at least four-fold higher than in diploid AA hybrids and even two-fold higher than in allotetraploid AACC hybrids [48, 53]. Such a boost in COs number was also associated with a decrease in the strength of CO interference when measured by the Gamma model [53], but these variations still remain to be assessed at the whole A genome level in allotriploid AAC hybrids. The molecular mechanisms responsible for this increase are yet not known, but they seem to be dependent on the addition of specific C chromosomes as shown by Suay *et al*. [53] who demonstrated a non-additive dosage effect. Immunolocalization on pollen mother cells of MLH1 protein-specific of Class I COs -, showed an increase of Class I COs rates by a factor 1.7 between homologous A chromosomes in allotriploid hybrids compared to diploids. However, that increase in male meiosis represented only a fraction of the increase found in female meiosis when considering both classes of COs from the genetic mapping analyses realized on progenies [48]. It was thus hypothesized that many of the extra COs generated in allotriploids could be due to an increase of Class II COs [48]. Whether this is the case or whether there is a difference between male and female meiosis still must be established, as well as the localization of the additional COs formed in allotriploids.

In the present study, we used the opportunity offered by the recent sequencing of *B*. *napus* and its diploid progenitors (*B*. *rapa* and *B*. *oleracea*) [54–57] to assess in *Brassica* allotriploids (i) the genome-wide extend of boost in CO numbers and (ii) the possible reshaping of the recombination landscapes. To do so, we generated diploid AA and allotriploid AAC hybrids, sharing the same A genotypes. Based on segregating populations obtained from these hybrids, we analyzed ~3000 COs per population with SNP markers well distributed all along the A chromosomes allowing us to reliably measure the recombination landscapes. Our results were validated on two genetic backgrounds and on male and female meiosis, enabling us to conclude that in all cases the presence of the 9 additional C chromosomes leads to a very substantial increase of COs between all homologous A chromosomes, especially in the vicinity of centromeres, with a strong decrease of interference of Class I COs compared to the case of diploid AA hybrids. Furthermore, we showed that the increase of COs depends on the genetic background as well as male and female meiosis in AAC hybrids, whereas in AA hybrids the pattern of recombination was highly conserved in all explored conditions. These results open the road to overcome recombination limits and in particular to introduce COs into cold genomic regions, providing a major breakthrough for plant breeding and genetics.

## Results

### Pairing occurs essentially between homologous chromosomes in diploid and allotriploid *Brassica* hybrids

**Fig 1:**
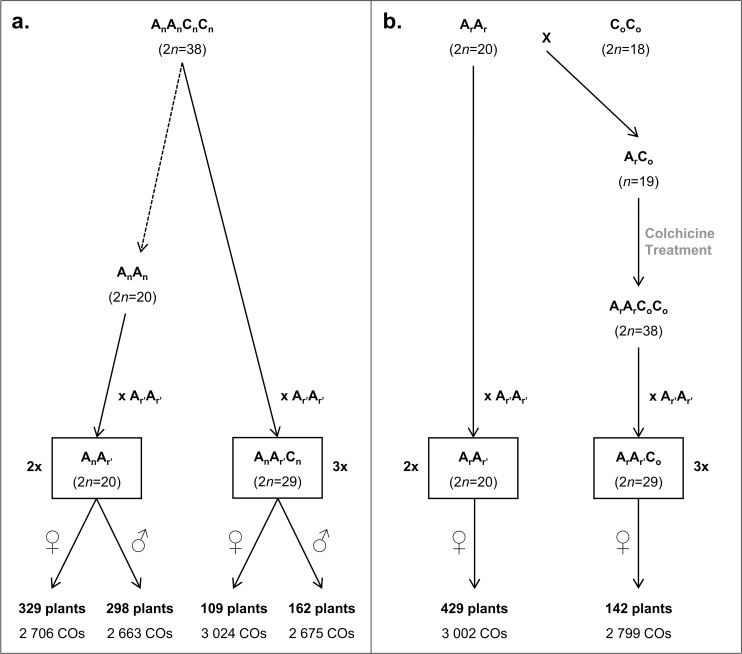
**Schematic detailing the production of (a) A**_**r**_**A**_**r’**_ **and A**_**r**_**A**_**r’**_**C**_**o**_ **and (b) A**_**n**_**A**_**r’**_ **and A**_**n**_**A**_**r’**_**C**_**n**_ **F**_**1**_ **hybrids combinations, and their progenies.** A_r_A_r_ and A_r’_A_r’_ represent *B*. *rapa* cv. ‘C1.3’ and ‘Chiifu-401’, respectively, C_o_C_o_ designates the *B*. *oleracea* cv. ‘RC34’, and A_n_A_n_C_n_C_n_ represents the *B*. *napus* cv. ‘Darmor’. The A_n_A_n_ plant corresponds to the diploid component of *B*. *napus* cv. ‘Darmor’ extracted from five generations of backcrosses by Pelé *et al*. [71]. The progenies were generated from female (♀) or male (♂) F_1_ hybrids using the *B*. *napus* cv. ‘Darmor’ for (**a**) and ‘Yudal’ for (**b**). Below each progeny are indicated the number of crossover observed from their genotyping.

To assess the immediate impact of ploidy level on homologous recombination in *Brassica*, two combinations of diploid AA (2*n* = 2x = 20) and allotriploid AAC (2*n* = 3x = 29) F_1_ hybrids were generated (Fig 1). For each combination, F_1_ hybrids presented the same AA genotype and differed only by the presence of 9 additional C chromosomes from cultivars of *B*. *oleracea* in A_r_A_r’_ *vs* A_r_A_r’_C_o_ and *B*. *napus* in A_n_A_r’_ *vs* A_n_A_r’_C_n_. We determined, from pollen mother cells in metaphase I of meiosis, that all F_1_ hybrids exhibited a regular meiotic behavior close to expectation with always 10 bivalents for AA plants and with 95 to 97.5% of cells with 10 bivalents and 9 univalents in AAC plants (S1 Fig and S1 Table). Using BAC-FISH experiments conducted with a specific BAC of the C genome, we showed that bivalents were mostly formed by A chromosomes in the AAC hybrids (S1 Fig), as already reported by Leflon *et al*. [58]. In contrast, C chromosomes remained at univalent stage and illegitimate pairing, either between A and C chromosomes or between two C chromosomes, occurred only exceptionally. Additionally, combining the specific BAC of the C genome and another one, which is specific of the homoeologous A05 and C04 chromosomes, we always observed the two A05 linked together without any homoeologous pairing with the C04 (S1 Fig).

### Genome-wide detection of crossovers between the homologous A chromosomes

The detection of CO events between the 10 homologous A chromosomes was performed by a genotyping approach on progenies derived from each AA and AAC F_1_ hybrid used as females, and also as males for the A_n_A_r’_ and A_n_A_r’_C_n_ hybrids. For that purpose, 204 SNP markers, specific of each AA and AAC F_1_ hybrids combination (with 199 SNPs in common), were chosen from a 60K Illumina Array based on their locations and polymorphisms (these choices also took into account the presence of C chromosomes in the progenies, see Methods).

These SNPs, showing the expected Mendelian segregation on A chromosomes and having concordant genetic and physical positions, covered 94.2% of the A genome of the sequenced *B*. *rapa* cultivar ‘Chiifu-401’ [57] that was used to obtain all F_1_ hybrids (Fig 1). Except for the A10 chromosome, which exhibited the lowest coverage (69.3%), the A chromosomes were almost entirely covered (from 90.9 to 98.9%). These SNPs were quite evenly distributed with a mean of 1 SNP every 1.25 Mbp (SE = 0.04, n = 194) for both combinations and spaced on average from 1.04 to 1.48 Mbp per A chromosome (S2 Table), thereby offering a solid framework to analyze recombination. For a precise assessment of recombination landscapes, we took advantage of previously published data indicating a boost of the recombination rate between the homologous A07 chromosomes in AAC hybrids compared to AA ones [48, 53]. We thus adjusted the number of progenies analyzed to get a similar total number of CO events between the homologous A genomes for all F_1_ hybrids. In total, 109 to 429 plants from each F_1_ hybrid gave rise to a number of COs ranging from 2706 to 3024 (Fig 1).

### Recombination rates receive a genome-wide boost in allotriploids with strong variations related to the sex of meiosis and the genetic background

**Fig 2:**
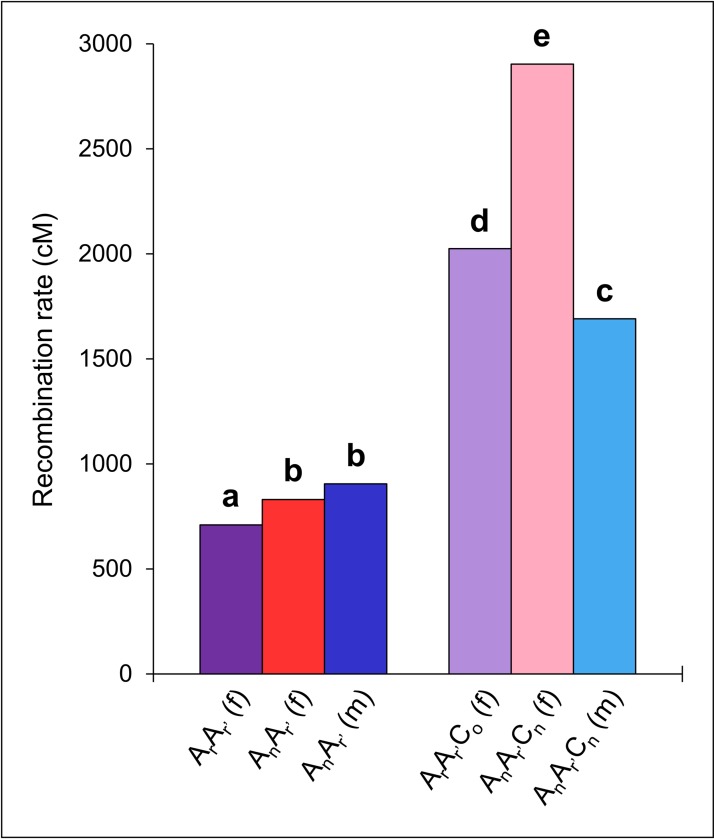
Recombination rates in Centimorgan (cM) for the cumulated A chromosomes in AA and AAC F_1_ hybrids. Values obtained for the diploid hybrids are indicated on the left of the graph from female A_r_A_r’_ (in purple), female A_n_A_r’_ (in red) and male A_n_A_r’_ (in blue). Values obtained for the allotriploid hybrids are indicated on the right of the graph from female A_r_A_r’_C_o_ (in light purple), female A_n_A_r’_C_n_ (in pink) and male A_n_A_r’_C_n_ (in light blue). Statistical differences, providing from a Bonferroni corrected Chi-squared test at a threshold of 5%, are indicated by the letters (a to e).

**Fig 3:**
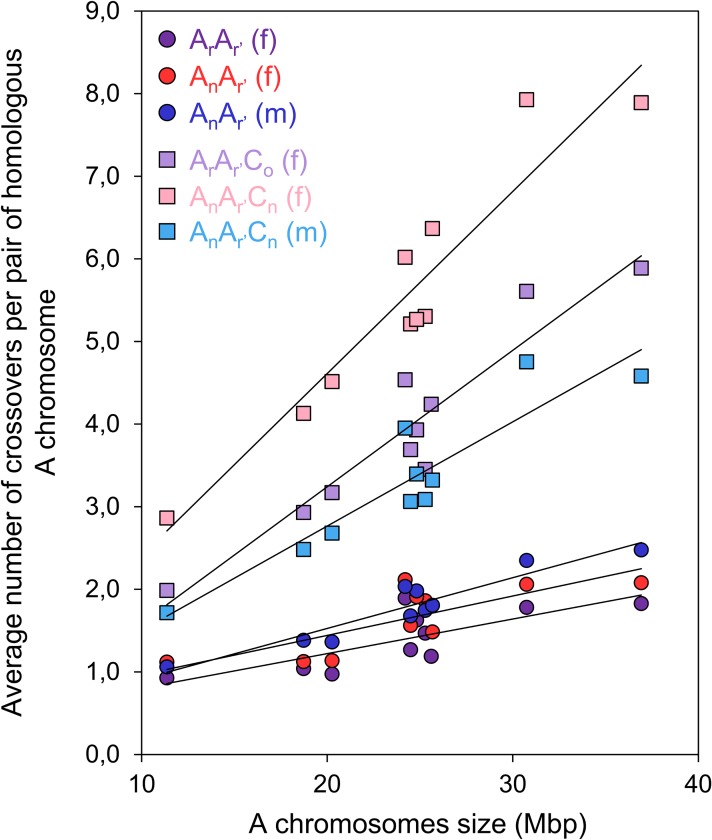
Relationship between the average numbers of crossovers formed per pair of homologous A chromosome and their physical size covered by SNP markers in Mbp for each of the AA and AAC F_1_ hybrids. Female A_r_A_r’_ (purple circle): y = 0,04185x+0,38480; R^2^ = 0.55. Female A_n_A_r’_ (red circles): y = 0,04771x+0,48786; R^2^ = 0.57. Male A_n_A_r’_ (blue circles): y = 0,061438x+0,297309; R^2^ = 0.88. Female A_r_A_r’_C_o_ (light purple squares): y = 0,16515x-0,06161; R^2^ = 0.88. Female A_n_A_r’_C_n_ (pink squares): y = 0,22049x+0,20151; R^2^ = 0.89. Male A_n_A_r’_C_n_ (light blue squares): y = 0,12617x+0,24270); R^2^ = 0.83.

According to previous results showing that the number of CO events increases between the homologous A07 chromosomes in allotriploid compared to diploid hybrids [48, 53], we extended the study of this effect to the whole A genome. Comparing in a genome-wide approach the CO rates obtained from the progenies of each F_1_ hybrid, we always reported significant variations between diploids and allotriploids, using 2-by-2 Chi-squared tests with a conservative Bonferroni-adjusted threshold of 5% (*p* < 2.2E-16, Fig 2). Per pair-wise comparison, a number of COs 1.8 to 3.4-fold higher was estimated in hybrids carrying an additional C genome. Similarly, at the level of each pair of A homologs, this observation was also verified (Corrected chi-squared test, *p* < 2.8E-13, S2 Fig), associated to a greater frequency of multiple COs in allotriploids (S3 Fig). Finding for all F_1_ hybrids significant positive linear regressions between the size of chromosomes (in Mbp) and their average number of COs (Fisher test, *p* < 0.05,), with R^2^ ranging from 0.55 to 0.89, we determined that the increase of number of COs in allotriploids was the most dramatic for the largest A chromosomes (Fig 3). For instance, every diploid exhibited on average per meiosis a unique CO for the smallest pairs of A homologs and about two for the largest. In contrast, two COs and up to eight were formed on average per pair of A homologs in allotriploids (Fig 3). Although we generalized the impact of the additional C genome, we pointed out that the increase of COs number from allotriploid compared to diploid hybrids varies from a pair-wise comparison to another.

Most specifically, from the combination of A_n_A_r’_ and A_n_A_r’_C_n_ hybrids (Fig 1), we noted that the number of extra COs formed from allotriploids was strongly impacted by the sex of meiosis. Indeed, comparing male and female meiosis, we found that the A_n_A_r’_ hybrids led to no significant variations for the rate of COs in contrast to the A_n_A_r’_C_n_ hybrids at the whole A genome level (Corrected chi-squared test, *p* < 2.2E-16, Fig 2) and per A homologs pair (Corrected chi-squared test, *p* < 2.6E-6, S2 Fig). When used as female, the A_n_A_r’_C_n_ hybrid showed a number of COs 1.7-fold higher than when used as male (1.5 to 1.9 depending on the pair of A homologs). Thus, in female meiosis, 16.4 (SE = 0.24, n = 329) and 55.5 (SE = 1.33, n = 109) COs were detected on average in A_n_A_r’_ and A_n_A_r’_C_n_ hybrids, respectively, corresponding to a 3.4-fold increase (from 2.6 to 4.3 depending on the pair of A homologs). In contrast, in male meiosis, an average of 17.9 (SE = 0.26, n = 298) and 33.0 (SE = 0.55, n = 162) COs were detected in A_n_A_r’_ and A_n_A_r’_C_n_ hybrids respectively, corresponding to a 1.8-fold increase (from 1.6 to 2.0 depending on the pair of A homologs). Interestingly, this variation observed in male meiosis was close to the one detected through the immuno-localization of MLH1 protein, which revealed a 1.7-fold increase of Class I COs in hybrids carrying an additional C genome [48].

Additionally, considering the second combination of F_1_ hybrids (Fig 1), we determined that the genetic background of both A and C genome was linked to COs rate variations. In fact, we found significant variations at the whole A genome level for the rate of COs between the diploids A_r_A_r’_ and A_n_A_r’_ used as females (Corrected chi-squared test, *p* = 1.2E-09), as well as between the allotriploids A_r_A_r’_C_o_ and A_n_A_r’_C_n_ used as females (Corrected chi-squared test, *p* < 2.2E-16) (Fig 2). However, per pair of A homologs, only the comparisons between the allotriploids showed significant variations (Corrected chi-squared test, *p* < 1.8E-04, S2 Fig). In all cases, at a same ploidy level the number of COs was more substantial when F_1_ hybrids carried genomes from the *B*. *napus* cv. ‘Darmor’, especially in allotriploids. In fact, between diploids, a number of COs only 1.2-fold higher was observed in A_n_A_r’_ hybrid (from 1.1 to 1.3 depending on the pair of A homologs) while a 1.4-fold increase was found in A_n_A_r’_C_n_ hybrid compared to the A_r_A_r’_C_o_ one (from 1.3 to 1.6 depending on the pair of A homologs). Consequently, between A_r_A_r’_ and A_r_A_r’_C_o_ female hybrids, exhibiting respectively 14.0 (SE = 0.20, n = 429) and 39.4 (SE = 0.88, n = 142) COs on average per meiosis, a lower difference was observed than in the previous comparison (A_n_A_r’_ *vs* A_n_A_r’_C_n_) with a number of COs only 2.8-fold higher at the whole A genome (from 2.1 to 3.6 depending on the pair of A homologs).

We expect the genome-wide increase in COs to be due to disruption of meiosis and CO control within the allotriploids. But one may object that there could be some post-meiotic selection, acting for instance at the level of pollen viability or seed development. Such selection forces could bias the progenies so as to increase CO rates. Undeniably there is selection in the allotriploid hybrids: their pollen viability was ~50% (using aceto-carmine coloration) and the number of seeds produced per pollinated flower was ~20% compared to diploid hybrids. To address this objection, we have tested the hypothesis that the increased CO rate genome-wide is due solely to post-meiotic selection (*cf*. Materials and Methods for the detailed procedures). We found that the levels of selection required by this hypothesis are completely incompatible with the actual levels observed. For instance, in the case of female meiosis, in the A_r_A_r'_ and A_r_A_r'_C_n_ hybrids, the post-meiotic selection hypothesis requires that only a fraction 10^−4^ of the gametes be viable, while in the A_n_A_r'_ and A_n_A_r'_C_n_ hybrids the required fraction is 3.5 10^−7^. These predictions are clearly absurd considering that we actually have on average 2 seeds per pollinated flower in the allotriploids (*vs* ~10 in the diploids). Thus, viability selection is unlikely to explain the enhanced CO numbers we deciphered, meiosis being most probably changed significantly in the allotriploid context.

### Recombination landscapes are dramatically reshaped in allotriploids, especially around centromeric regions

**Fig 4:**
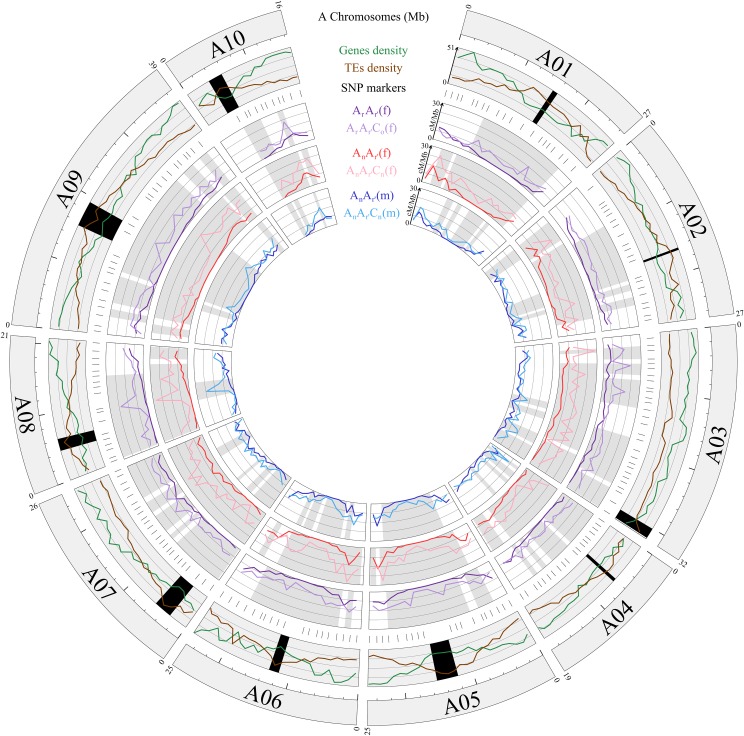
Circos diagram comparing the recombination rates along the 10 A chromosomes in cM per Mbp between the AA and AAC F_1_ hybrids. In the first outer circle are represented the 10 A chromosomes of the *B*. *rapa* cv. ‘Chiifu-401’ genome sequence version 1.5 [57]. Their sizes are indicated by the values in megabase pairs above each chromosome, and a ruler drawn underneath each chromosome, with larger and smaller tick marks every 10 and 2 Mbp, respectively. In the second outer circle, is detailed the architecture of each A chromosome, including the genes and transposable elements (TEs) densities from the version 1.5 of the *B*. *rapa* cv. ‘Chiifu-401’ genome sequence [57]. The active centromeres are delimited in black using the positions established by Mason *et al*. [81]. In the third outer circle, are indicated the positions of the 204 SNP markers used for the genotyping of the progenies of each AA and AAC F_1_ hybrid. In the three inner circles, are represented the pair-wise comparisons for the recombination landscapes (in cM per Mb) of progenies deriving from the AA and AAC F_1_ hybrids. Toward the Circos diagram center, are compared (i) the A_r_A_r’_ (purple lines) and A_r_A_r’_C_o_ (light purple lines) female hybrids, (ii) the A_n_A_r’_ (red lines) and A_n_A_r’_C_n_ (pink lines) female hybrids, and (iii) the A_n_A_r’_ (blue lines) and A_n_A_r’_C_n_ (light blue lines) male hybrids. For each interval between adjacent SNP markers, the heterogeneity of CO rates was assessed using Chi-squared tests and significant differences at a threshold of 5% were indicated for each pair-wise comparison between AA and AAC F_1_ hybrids in grey.

Having found that the substantial increase of CO numbers formed between A07 homologs in the presence of the additional C genome in *Brassica* allotriploids extends in fact to all A chromosomes, a key point we sought to clarify was the impact of such a boost on the recombination landscapes. We had two hypotheses: (i) either at the scales of each A chromosome the increase of CO rates is proportional to the ones observed in diploids, (ii) either that is not the case and CO rates increase mostly in specific genomic regions. Visually, from the representations of CO rates along the 10 A chromosomes for each pair-wise comparison of diploid and allotriploid hybrids established through their progenies (Fig 4), the second hypothesis is the most relevant. Statistically, to confirm our visual interpretation we used an approach developed by Bauer *et al*. [44] in which the shapes of the recombination landscapes are compared 2-by-2 (see Methods). When comparing the females A_r_A_r’_ and A_r_A_r’_C_o_, the females A_n_A_r’_ and A_n_A_r’_C_n_, or the males A_n_A_r’_ and A_n_A_r’_C_n_, our analyses always revealed significant differences for the whole A genome and for each individual A chromosome (Corrected chi-squared test, *p* < 0.05) (S4 Fig, S3 Table). Thus, we demonstrated that in allotriploids the increase of CO rates is not proportional to the ones observed in diploids.

Additionally, despite clear differences in profiles between diploid and allotriploid hybrids, we found that recombination landscapes did not differ much between the F_1_ hybrids presenting a same level of ploidy. Indeed, when comparing the male and female A_n_A_r’_ hybrids (S5 Fig, S4 Table) or the females A_r_A_r’_ and A_n_A_r’_ hybrids (S6 Fig, S5 Table), no significant differences were observed for the whole A genome or for each individual A chromosome. Consistently, for both of these comparisons, only one interval between the SNP markers showed a significant variation in the proportion of COs (Chi-squared test, *p* < 0.05, S7 Fig, S6 Table). Similarly, when comparing the male and female A_n_A_r’_C_n_ hybrids (S5 Fig, S4 Table) or the female A_r_A_r’_C_o_ and A_n_A_r’_C_n_ hybrids (S6 Fig, S5 Table), no significant differences were observed for the whole A genome or for each individual A chromosome. However, at the level of individual intervals, we found more significant differences in CO rates than for the two pair-wise comparisons between diploids (Chi-squared test, *p* < 0.05, S8 Fig, S6 Table). Thus, we concluded that the sex of meiosis and the genetic background were not related to global modifications of recombination landscapes within either diploids or allotriploids, but can lead to local variations especially in allotriploids.

Regarding the A genome architecture (genes density, TEs density and centromeres locations), the recombination landscapes established from the progenies of each F_1_ hybrid appeared more closely correlated in diploids than in allotriploids (Fig 4). For each diploid, the highest CO rates arose mostly in the distal parts of the A chromosomes while the lowest rates were always located around the centromeric regions. This is particularly relevant when comparing with different features of chromosome architecture in view of the observation that COs preferentially occur in genomic regions that are depleted in TEs and enriched in genes. Additionally, from regression analyses, we highlighted that CO rates tend to increase gradually from centromeres to chromosome extremities in these diploids, as often observed in plants [32, 34]. Indeed, in an A genome-wide approach (S9 Fig, S7 Table), using the relative recombination rates normalized per A chromosome (%) and their relative distance from the centromeres (%), we observed positive linear relationships within the females A_r_A_r’_ (R^2^ = 0.53) and A_n_A_r’_ (R^2^ = 0.48), and the male A_n_A_r’_ (R^2^ = 0.51) (Fisher test, *p* < 2.2E-16). Consistently, when a single A chromosome-arm or a whole A chromosome was studied, the regression analyses always showed respectively significant linear and order-2 polynomial relationships (Fisher test, *p* < 0.05) with R^2^ ranging from 0.31 to 0.93 (S10 Fig, S7 Table).

Compared to the recombination landscapes described in diploids, those of allotriploids were striking (Fig 4). Regardless of the AAC hybrids, the most astonishing result was the observation of a substantial number of COs in every interval between the adjacent SNP markers used. Specifically, we identified that COs were formed even in intervals around and including the centromeric regions while, for all diploid AA hybrids, these genomic regions were totally deprived of COs although representing between 8.1 and 11.9% of the A genome. Additionally, by comparing the proportion of COs arising in given intervals, we revealed significant differences for most of the 194 intervals between AA and AAC F_1_ hybrids, including surrounding regions of the centromeres but not only (chi-squared test, *p* < 0.05) (Fig 4, S6 Table). Indeed, that result concerns 168 (86.6%) and 48 (24.7%) intervals when comparing A_n_A_r’_ and A_n_A_r’_C_n_, respectively in female and male meiosis, and 129 (66.5%) for the A_r_A_r’_-A_r_A_r’_C_o_ pair, with in all cases a higher frequency of COs in allotriploids. Although these significant variations concern almost the whole of the A chromosomes, it seems that distal genomic regions were the least impacted (Fig 4, S6 Table). Consequently, the CO rates in allotriploids seem more homogenous along the A chromosomes compared to diploid AA hybrids. Consistently, we determined by regression analyses that CO rates were less related to the centromeres’ location in allotriploids compared to what was previously found in diploids. Indeed, at the A genome scale we detected significant positive linear relationships (Fisher test, *p* < 2.2E-16) but explaining only 9 to 15% of the variation *vs* about 50% in diploids (S9 Fig). Furthermore, for most A chromosome-arms or whole A chromosomes, no significant relationships were found by the regression analyses (S10 Fig, S7 Table). Thus, in regards to TEs and genes densities, the recombination landscapes along the A chromosomes could be unrelated in allotriploids, whether used as female or male (Fig 4).

### Crossover interference is strongly suppressed in allotriploids

**Fig 5:**
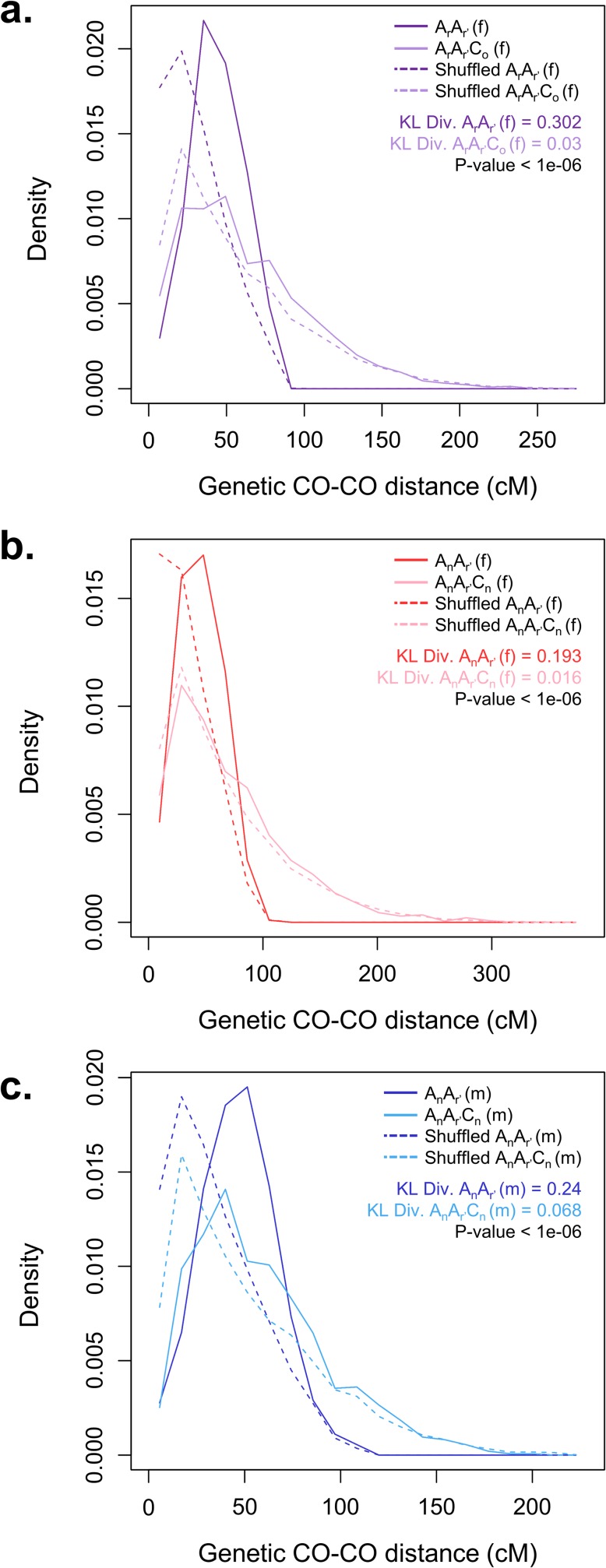
Distributions of inter-crossover genetic distances in AA and AAC F_1_ hybrids when pooling all 10 chromosomes. Comparison of the distribution of genetic distances between successive COs from populations deriving of (**a**) females A_r_A_r’_ (in purple) *vs* A_r_A_r’_C_o_ (in light purple), (**b**) females A_n_A_r’_ (in red) *vs* A_n_A_r’_C_n_ (in pink) and (**c**) males A_n_A_r’_ (in blue) *vs* A_n_A_r’_C_n_ (in light blue). Data are pooled over the 10 A chromosomes. X-axis: genetic distance between successive COs. Solid lines correspond to experimental data. Dashed lines indicate the corresponding distributions in the "no-interference" situation, obtained by re-shuffling CO positions of experimental data (see Methods). For each population, the Küllback-Leibler divergence (KL Div.) from the experimental to the "no-interference" distribution provides a quantitative measurement of interference strength. p-value: one-sided *p*-value of the H_0_ hypothesis that the diploids and allotriploids have the same KL Div. index (and thus interference strength). Sufficiently small values indicate significantly higher interference in diploids than in allotriploids (see details in Methods).

Crossover interference, that is the non-independence of CO events in a meiosis, generally transpires as a deficit in close-by COs. To reveal such an effect in a model-independent way, we determined the distribution of genetic distances between adjacent COs for each chromosome in our AA and AAC hybrids. Note that such an analysis is thus necessarily based only on chromosomes of progenies inheriting at least 2 COs from the meiotic bivalent and follows directly the procedures used in Barchi *et al*. [59]. We found that in all diploids the distributions were quite peaked around their mean (Fig 5 shows the result pooled over chromosomes), with a clear deficit at small values, thereby indicating strong interference. In contrast, we found that in all allotriploids the peak was less pronounced and there was a smaller deficit at small distances, indicating less interference. And as expected, one also saw that the distribution was broader in the case of the allotriploids, again indicating less interference there. Furthermore, we can reject the hypothesis H_0_ that the additional C chromosomes have no effect on these distributions by using the Kolmogorow-Smironov test (S8 Table provides the *p*-values for the different tests). For the pools of the three comparisons A_r_A_r'_ *vs* A_r_A_r'_C_o_ used as females, A_n_A_r'_ *vs* A_n_A_r'_C_n_ used as females, and A_n_A_r'_ *vs* A_n_A_r'_C_n_ used as males, the *p*-values were all less than 10^−6^. For each comparison, the two distributions corresponding to diploid and allotriploid cases can thus be considered as different in a statistical sense. The same trends were seen at the individual chromosome levels (S11 Fig, S8 Table).

To provide further support to the claim that allotriploids have less interference than diploids, we have used random shufflings of the data to generate the distributions expected in the absence of interference (*cf*. Methods for the associated technical explanations). These “no-interference” distributions are shown *via* dashed lines in Fig 5 for pools and S11 Fig for individual chromosomes. At a qualitative level, we saw that whereas the two distributions (experimental and “no-interference”) obtained from diploids differed pretty much everywhere, the distributions for the allotriploids were quite similar to one another except at very short distances. To render a quantitative assessment, we have calculated for each F_1_ hybrid the Kullback-Leibler (KL) divergence (see Methods for definitions) between the experimental and “no-interference” distributions. These measures of KL divergences are given in S8 Table for pools and S11 Fig for individual chromosomes. For instance, the KL divergence was 0.302 for the A_r_A_r'_ female diploid, whereas for the A_r_A_r'_C_o_ female allotriploid the KL divergence was 0.030 when pooling over the 10 chromosomes. For each comparison, we found that the KL divergence between the experimental curve and the “no interference” curve was higher in the diploids than in the allotriploids, indicating that interference was lowered in the allotriploids. To assess the statistical significance of these differences, we used a permutation-based approach (see Methods). We were able to reject the hypothesis that the female A_r_A_r'_ and female A_r_A_r'_C_o_ hybrids have the same KL divergence (one sided *p*-value < 10^−6^ when pooling all chromosomes) and similarly for the other two diploid-allotriploid comparisons (*cf*. values indicated in Fig 5 when chromosomes were pooled, and S11 Fig and S8 Table for individual chromosomes). We thus here again reach the conclusion that CO interference is strongly suppressed in the AAC hybrids.

Lastly, let us mention that interference strength was only weakly affected by the genetic background of hybrids as can be seen in S12 Fig. Nevertheless, the interference strengths as indicated by the Küllback-Leibler divergence differed significantly among the genotypes for the diploids (A_r_A_r'_ *vs* A_n_A_r'_) and allotriploids (A_r_A_r'_C_o_ *vs* A_n_A_r'_C_n_). Similarly, when comparing male *vs* female types of meiosis, S13 Fig suggests that there was little difference in interference strengths here again. Nevertheless, in the case of allotriploids (male A_n_A_r'_C_n_ *vs* female A_n_A_r'_C_n_), the greater interference in male meiosis was statistically significant (two-sided *p*-value < 10^−6^).

### Crossover formation modeling suggests that interference amongst Class I COs is strongly suppressed in allotriploids.

The genotyping of progenies allows one to detect COs but does not say which are of Class I or of Class II. In most organisms ZMM-dependent Class I COs are strongly interfering while MUS81-dependent Class II COs seem to be compatible with no interference. If overall interference between COs is strongly suppressed in allotriploids as shown in the previous paragraph, this may be because the interference amongst Class I COs is diminished or because there are more Class II COs. We tackled this question by fitting models of CO formation to the data, extracting (i) the strength of interference within Class I COs and (ii) the proportion of COs that are in Class II. Two frequently used models are the Beam Film [60, 61] and the Gamma [62] models. In the Beam Film model, interference strength corresponds to a distance *lambda* over which interference acts strongly: the greater that distance the stronger the interference strength (in practice interference effects decay exponentially with the ratio: distance over *lambda*). In the Gamma model [62], the interference strength parameter *nu* is associated with the regularity of inter-CO distances. If *nu* = 1, there is no interference, and as *nu* increases the coefficient of variation of inter-CO distances goes to zero.

Our results are summarized in S9 Table. Focusing on each model's measure of interference strength (*lambda* in the Beam Film model, *nu* in the Gamma model), there was a clear trend whereby interference in Class I COs was reduced in the allotriploids compared to the diploids. For instance, whatever the genetic background or the sex of meiosis, the average over the 10 A chromosomes of the interference strength was higher for the diploids than the allotriploids. To ensure that this conclusion is supported by objective criteria, we have performed a test of the hypothesis that diploids and allotriploids have the same mean interference strengths when averaging over chromosomes (see Methods). That hypothesis can be rejected for both the Beam Film model and the Gamma model (one-sided *p*-value < 10^−5^) when pooling all three diploid-allotriploid comparisons, providing strong evidence that average interference amongst Class I COs is higher in the diploids than in the allotriploids. The analyses at the level of each separate diploid-allotriploid comparison also obeyed this trend: all *p*-values obtained with either model were less than 10^−3^ with the following exceptions: *p*-value = 0.065 for A_r_A_r'_ *vs* A_r_A_r'_C_o_ used as females with the Beam-Film model, and *p*-value = 0.152 for A_n_A_r'_ *vs* A_n_A_r'_C_n_ used as females with the Beam-Film model. One may thus conclude that both the Beam Film and Gamma models predict that the interference of Class I COs is strongly suppressed in the presence of the additional C genome.

## Discussion

In the present study, we provided a whole genome characterization of the impact of additional C chromosomes on homologous recombination, based on analyses of segregating populations from diploid AA (2*n* = 2x = 20) and allotriploid AAC (2*n* = 3x = 29) hybrids of *Brassicas*. Extending previous published results limited to one homologous pair of chromosomes [48, 53], we have now established that the number of COs strongly increases between all the 10 homologous A chromosomes in hybrids carrying an additional C genome. Furthermore, whatever the AAC hybrid, striking modifications in the shapes of recombination landscapes were observed from their progenies, including surprisingly major increases in recombination rates in genomic regions close to centromeres. Associated with these changes we also found a strong decrease of interference between COs, be-it from model-independent features or *via* fitting our data to two standard interference models.

### In *Brassica* hybrids, the additional C genome always results in extra crossovers between homologous chromosomes which belong to Class I in male meiosis

The analysis of meiotic behavior revealed that chromosome pairing is limited to homologous A chromosomes in case of allotriploids as already described by several authors [48, 53, 58], indicating that all extra COs formed result only from homologous recombination. Based on our genome-wide data, we observed a total number of COs (Class I and II COs) in male meiosis that was very similar to the number of Class I COs from MLH1 immuno-localization reported by Leflon *et al*. [48] between AA and AAC hybrids (the factor of enhancement when going from diploid to allotriploid was 1.8 *vs* 1.7, respectively). Specifically, an average of 29.3 Class I COs per meiosis was observed in male AAC hybrids by Leflon *et al*. [48] while we found an average of 33.0 COs. Clearly, based on this comparison, the great majority of the extra COs generated in the presence of the additional C genomes belong in fact to Class I for male meiosis. The minor differences between 29.3 and 33.0 might be attributed to Class II COs, giving a predicted proportion of 11.2% in agreement with several observations indicating that the proportion of Class II COs varies from 5 to 20% in most species [3]. The information provided by these MLH1 measurements thus reinforces our claim that the enhanced recombination rates in progenies of allotriploid hybrids are most probably due to changes in the meiotic processes themselves rather than consequences of post-meiotic selection. Concerning female meiosis, a 1.7-fold higher CO rate on the whole A genome was observed compared to the case of male meiosis in AAC hybrids. The origin of these extra COs remains unknown and needs further investigation. This could be done either by knocking-out genes involved in the formation of Class I or II COs as already performed in *A*. *thaliana* [63–65], or by immunolocalization of MLH1 in female meiosis, which is still highly challenging in plants even if first results were reported in *A*. *thaliana* [66]. In contrast, for all AA hybrids, COs which belong to Class II were estimated to contribute from 4% to 7% of the total, based on the parameter *p* estimated from fitting the Beam-Film-sprinkling and Gamma-sprinkling models, corresponding to on average one or fewer Class II COs per meiosis (S9 Table). Considering this estimation, our male AA hybrid exhibits on average 16.9 Class I COs per meiosis, which is very consistent with Leflon *et al*. [48] who reported on average 16.5 MLH1 foci per meiosis. Thus, in diploid AA hybrids, Class II COs constitute a very small minority of total COs, smaller than in studied plants such as *A*. *thaliana* for which Class II COs represent about 15% of total COs [3]. Additionally, we identified that the number of COs increases linearly with the chromosome length in all our hybrids (Fig 3), in agreement with the results reported in a large range of species, especially in plants [3]. This could be related to the size of the Synaptonemal Complex, which is greater for the largest chromosomes and positively correlated with the number of COs formed per pair of homologous chromosomes in many species [43, 67].

These sex-related differences in CO numbers were observed exclusively between AAC hybrids (Fig 2, S2 Fig). In many organisms including plants, variations in CO numbers between male and female meiosis are observed, a phenomenon called heterochiasmy [68]. Depending on the species, these differences can be highly variable. For instance, *A*. *thaliana* male meiosis exhibited significantly more COs than female meiosis [43], while the opposite pattern was observed in *B*. *oleracea* [69], and no variations were detected in *B*. *napus* [70]. Although the mechanisms responsible for these variations are not yet known, our results suggest that diploid AA hybrids are not subject to heterochiasmy.

We also observed that the genetic background influences the frequency of COs (Fig 2, S2 Fig). Indeed, a proportion of COs 1.2 and 1.4-fold higher per meiosis was measured respectively in AA and in AAC hybrids carrying either A_n_ or A_n_ and C_n_ genomes of *B*. *napus* compared to *B*. *rapa* A_r_ or A_r_ and C_o_ from resynthesized rapeseed (Fig 1). In the case of diploids, there is no doubt that this variation is attributed to the A genome origin (*B*. *napus vs B*. *rapa*), the A_n_ genome presenting about 70% of identity with the *B*. *napus* one due to the genome extraction strategy [71]. Such variation at the same ploidy level has been reported in several species such as *Hordeum vulgare*, *A*. *thaliana*, *Z*. *mays* or *B*. *napus* [44, 72–74]. Genetic factors controlling such variations (i.e. trans-acting QTLs affecting the genome-wide recombination rate) were identified in *A*. *thaliana*, *Z*. *mays*, and *T*. *aestivum* [75]. For AAC hybrids, the variation of COs number was clearly more pronounced with a CO frequency 1.4-fold higher in A_n_A_r’_C_n_ hybrid compared to the A_r_A_r’_C_o_ hybrid obtained from resynthesized rapeseed (Fig 1). We can hypothesize that we cumulated the effects of distinct A and C genomes. Indeed, the A and C genomes of *B*. *napus*, present in A_n_A_r’_C_n_ plant, diverged from the one of their current progenitors, present in A_r_A_r’_C_o_ plant [54]. Additionally, a genetic control of homoeologous recombination between A and C genomes was described by Jenczewski *et al*. [76], with a major QTL, called *PrBn*, carried by the C09 chromosome [77]. It was suggested that this genetic control might be responsible of the recombination rate differences observed between AAC hybrids produced from different *B*. *napus* varieties [74]. Similarly, the role of the C09 chromosome has also been described on the control of homologous pairing in AAC hybrids by Suay *et al*. [53].

### In *Brassica* hybrids, the additional C genome reshapes CO landscapes between all the homologous A chromosomes regardless of sex of meiosis or genetic background

We showed for all pair-wise comparisons between AA and AAC hybrids that the presence of the additional C genome drives a dramatic reshaping of the recombination landscapes as measured from the progenies along each of the 10 homologous A chromosomes (Fig 4, S4 Fig, S3 Table).

In contrast, the progenies deriving from the diploid AA hybrids showed similar CO landscapes regardless of sex of meiosis or genetic background (S5–S7 Figs, S4 and S5 Tables). Differences in recombination landscapes across different genotypes have been reported in *Z*. *mays* [44], as well as sex-related differences in *A*. *thaliana* [42, 43], especially in distal part of chromosomes. In our study on *Brassicas*, we showed that all diploids led to a gradual increase of CO rate towards chromosome extremities (S9 and S10 Figs, S7 Table). This trend was reported in several other plants [32–34] and could be related to the genome architecture. Indeed, CO frequencies were found to positively correlate with gene density and negatively with TE density in plants [36–39]. Consistently, we always showed that diploids led to higher CO rates in the distal part of the A chromosomes, which are enriched in genes and depleted in TEs. In contrast the genomic regions around centromeres, which are depleted in genes and enriched in TEs, were totally deprived of COs in the case of all our diploid AA hybrids, representing ~8 to 12% of the A genome (Fig 4). This feature remains quite conserved across eukaryotic species, suggesting that diploid AA hybrids are subject to similar controls for meiotic recombination in these particular genomic regions [30–31].

Compared to what was previously described within *Brassica*s diploids, the allotriploid AAC hybrids led to different recombination landscapes among the 10 A chromosomes, especially around centromeres (Fig 4, S4 Fig, S3 Table), whatever the sex of meiosis or the genetic background (S5–S8 Figs, S4 and S5 Tables). The main result we obtained is that COs occur in all marker intervals including those totally deprived of COs in progenies of AA hybrids, even though we ensured a similar number of genome-wide COs observed (~3000). Thus, the additional C genome induces new recombining regions on the A chromosomes colocalized with the surrounding regions of centromeres (Fig 4). Note that the appearance of these new regions with COs does not seem compatible with the hypothesis that the extra COs are due to post-meiotic selection: indeed, if there are no COs in these regions during meiosis, they cannot arise *de novo* after. In yeast and human, centromere-proximal COs were associated with improper chromosome segregation during meiosis, causing aneuploidy [78, 79]. In our case, it was previously shown that the segregation of the A chromosomes during meiosis of *Brassica* allotriploid hybrids is regular [58, 80]. A justification might be that the centromere itself is not that close to these extra COs. However, it was not possible to assess the exact position of the centromeres [81] or to design specific markers due to low polymorphism in centromeric regions enriched in repeated sequences [82, 83]. In spite of the small size of *Brassica* chromosomes, a possible strategy could be to combine the immuno-localization of MLH1 protein with histone marks specific of euchromatic or heterochromatic regions (i.e. H3K4me3, H3K9me2), as already realized in barley [84].

The mechanisms likely to be involved in the AAC hybrids allowing the modifications of the recombination landscapes and the reduced CO interference for all the homologous A chromosomes are yet not known. However, for each pair-wise comparison realized between AA and AAC hybrids, the A genotypes were identical, especially for A_r_A_r’_ *vs* A_r_A_r’_C_o_ (Fig 1). Thus, it seems unlikely that genomic features of the A chromosomes were directly involved in the localization of extra COs detected through the progenies of AAC hybrids, in contrast with what is now known for recombination hotspots. Indeed, in plants and especially in *A*. *thaliana*, hotspots were reported to occur preferentially in the vicinity of transcription start sites of genes [22, 24], which are enriched in A-, CTT- and/or CCN-repeats [22, 25, 26, 29]. However, we cannot exclude that a mobility of Transposable Elements (TEs) could be induced by additional C chromosomes and associated to modifications of the recombination landscapes. Indeed, TEs as Mutator-like elements were correlated to an increase of CO frequency when they transposed in *Z*. *mays* and *A*. *thaliana* [29, 85, 86]. However, in the case of *B*. *napus*, low TEs mobility were reported after hybridization between *B*. *oleracea* and *B*. *rapa* [87].

In contrast, in resynthesized *B*. *napus* several modifications for DNA methylation were found following the polyploidisation event [88–90] and a reasonable assumption is that C chromosomes could affect the epigenetic features of homologous A chromosomes. Indeed, it was recently reported that knock-out of genes involved in DNA methylation (*ddm1* and *met1*) impact the COs landscapes [91–94]. Although the change in DNA methylation induced by the C genome is an attractive hypothesis, most of those authors reported exclusive variations in euchromatic regions and no change in the total number of COs while we always observed a global variation of the CO number with AAC hybrids (Fig 4). Only the knock-out of *met1* has showed centromere-proximal COs [94] or modification of recombination hot-spots [95]. However, for this latter case, the characterization of an euchromatic hotspot increasing its activity in defective *met1* also revealed a low nucleosome density [95]. Thus, a change in the chromatin compaction could be associated to the variation in recombination patterns observed from AAC hybrids. This feature is particularly relevant as it is now known that the DSBs, initiating the formation of COs, are dependent on low nucleosome density and histone marks involved in open chromatin (H3K9ac and H3K4me3) in yeast and mammals [96–98]. Thus, the modification of DSBs localization can influence CO formation. However, CO landscapes do not fully mirror the DSBs activity [20]. It is also possible that a change in the chromatin compaction could directly modify the COs location. Indeed, a change of Class I COs distribution in *A*. *thaliana* mutants defective in E1 enzyme of the neddylation complex, involved in chromatin compaction, was reported [99]. This mechanism could perhaps occur in the vicinity of centromeric regions, well known to be in heterochromatic regions [30, 31]. Additionally, in these mutants [99], Class I COs were found to cluster together, modifying CO interference. However, for our material, only manual crosses are used without gene knock-out. We can hypothesize that putative changes in CO regulation might be induced by the gene balance modification in AA *vs* AAC hybrids in which the additional C genes cannot contribute to CO formation between C chromosomes due to the absence of C homologs. This impact of gene balance modifications on transcriptomic regulation was recently described in *Brassica* [100] but also on phenotypic traits in maize [101]. As meiotic genes return to single copy after whole genome duplication [102,103], changes in meiotic gene balance are likely to induce dramatic changes in COs regulation but may involve specific genes carried by some C chromosomes as it has been reported that the dosage of C chromosomes has no additive effect [53].

### Additional C chromosomes affects the intensity of interference between crossovers

In our comparisons of diploids and allotriploids, we found that the presence of the additional C genome systematically lowered CO interference (Fig 5, S11 Fig, S8 Table). This decrease was previously hypothesized to be due to increased numbers of non-interfering Class II COs [48]. But as demonstrated in the beginning of this discussion, the extra COs in the male AAC hybrid are mainly of the Class I type. Thus, the decrease in CO interference induced by the additional C genomes is due to lower interference amongst Class I COs. This surprising result was corroborated by our model-based analyses (using both Beam-Film and Gamma models in a two-pathway framework). This loss of interference calls for further work to determine whether it might be due to changes in the properties of the axes or to disruption of the chronology of the different events required for proper repair of the double-strand breaks.

Our results open a new avenue to overcome the meiotic recombination rules in *Brassica* species, providing new perspectives for geneticists and plant breeders to enhance the genetic shuffling of diversity by generating new allelic combinations. In particular, for *Brassica* breeding applications, the use of allotriploids offers the opportunity to speed the introgression of agronomical traits of interest from the diploid progenitors *B*. *rapa* and *B*. *oleracea* into rapeseed by backcrossing [48].

## Materials and methods

### Plant materials production

Two combinations of diploid AA (2*n* = 2x = 20) and allotriploid AAC (2*n* = 3x = 29) F_1_ hybrids were generated using *B*. *rapa*, *B*. *oleracea* and *B*. *napus* seeds available at the Genetic Resource Center, BrACySol (UMR IGEPP, Ploudaniel, France).

For the first combination, detailed in Fig 1, an old non-homogeneous French forage variety *B*. *rapa* var. *rapifera* ‘C1.3’ (A_r_A_r_, 2*n* = 2x = 20) was crossed to a homozygous doubled haploid line *B*. *oleracea* var. *alboglabra* ‘RC34’ (C_o_C_o_, 2*n* = 2x = 18). The resulting A_r_C_o_ amphihaploid was treated with colchicine to resynthesize an allotetraploid *B*. *napus* individual called ‘RCC S0’ (A_r_A_r_C_o_C_o_, 2*n* = 4x = 38) [104]. Then, RCC S0 as well as a *B*. *rapa* cv. ‘C1.3’ were crossed as female with the sequenced Chinese cabbage variety *B*. *rapa* var. *pekinensis* ‘Chiifu-401’ (A_r’_A_r’_, 2*n* = 2x = 20) [57] to obtain a diploid A_r_A_r’_ (2*n* = 2x = 20) and an allotriploid A_r_A_r’_C_o_ (2*n* = 3x = 29) F_1_ hybrid, respectively. These F_1_ hybrids, presenting exactly the same pairs of homologous A chromosomes, were then cytogenetically characterized to confirm their chromosome composition. Finally, progenies were generated by crossing these F_1_ hybrids as female to *B*. *napus* var. *oleifera* ‘Darmor’ (A_n_A_n_C_n_C_n_, 2*n* = 4x = 38), a winter cultivar recently sequenced by Chalhoub *et al*. [54].

For the second combination detailed in Fig 1, the natural *B*. *napus* cv. ‘Darmor’ (A_n_A_n_C_n_C_n_, 2*n* = 4x = 38) and its diploid A_n_A_n_ component (2*n* = 2x = 20) extracted by Pelé *et al*. [71] were both crossed as female with *B*. *rapa* cv. ‘Chiifu-401’ to generate one diploid A_n_A_r’_ (2*n* = 2x = 20) and one allotriploid A_n_A_r’_C_n_ (2*n* = 3x = 29) F_1_ hybrid, respectively. These F_1_ hybrids, presenting close A genotypes, were cytogenetically characterized and crossed as male and female to *B*. *napus* var. *oleifera* ‘Yudal’ (2*n* = 4x = 38), a Korean spring rapeseed line, to generate progenies.

In all cases, progenies were generated by manual pollination in the same environmental conditions, considering that all F_1_ hybrids were grown at the same time in the same greenhouse.

### Cytogenetic characterization of the F_1_ hybrids

Young floral buds were harvested from either AA or AAC F_1_ hybrids in order to characterize their meiotic behavior from at least 20 Pollen Mother Cells (PMCs) at Metaphase I of meiosis following the protocol of Suay *et al*. [53]. BAC-FISH experiments were performed for the AAC F_1_ hybrids using the *B*. *olerace*a BAC clone Bob014O06 [105] and the *B*. *rapa* BAC clone KBrH033J07 [106] that were labelled by random priming with Alexa 488-5-dUTP and biotin-14-dUTP (Invitrogen, life technologies), respectively. The BAC KBrH033J07 hybridizes to A05 and C04 chromosome pairs in *B*. *napus* whereas the BAC Bob014O06 was used as “genomic *in situ* hybridization (GISH)-like” to distinguish specifically all C chromosomes in *B*. *napus*.

### DNA extraction

Genomic DNA from lyophilized young leaves of F_1_ hybrids, their progenies as well as the *B*. *rapa* (‘C1.3’ and ‘Chiifu-401’), *B*. *oleracea* (‘RC34’) and *B*. *napus* (‘Darmor’, ‘RCC S0’ and ‘Yudal’) varieties were extracted with the sbeadex maxi plant kit (LGC Genomics, Teddington Middlesex, UK) on the oKtopure robot at the GENTYANE platform (INRA, Clermont-Ferrand, France). The DNA concentrations were then adjusted for each sample to 60 ng.µL^-1^.

### Genotyping analysis and SNPs selection

A first step of genotyping was performed using the *Brassica* 60K Illumina Infinium SNP array, developed and released for *B*. *napus* with 52,157 Single Nucleotide Polymorphisms (SNPs) (http://www.illumina.com/). Hybridizations were run according to the standard procedures provided by the manufacturer for each genomic DNA extracted from the F_1_ hybrids as well as 3 technical replicates of the *B*. *rapa*, *B*. *oleracea* and *B*. *napus* varieties. The genotyping data obtained were visualized with the Genome Studio V2011.1 software (Illumina, Inc., San Diego, CA, USA) and processed using a manually adapted cluster file.

In order to perform the genotyping of the A genome for the progenies deriving from each AA and AAC F_1_ hybrid, we took care to retrieve only the SNPs presenting a pattern of polymorphism in the flanking sequences which was adapted for this analysis, even in the presence of C chromosomes in progenies. Thus, only the SNPs for which the sequenced *B*. *rapa* cv. ‘Chiifu-401’ (used as parent for all F_1_ hybrids) was polymorph to all the other parents used for the production of F_1_ hybrids and their progenies, were selected. The sequence context of these SNP markers (size ranging between 96 to 301 bp) were then blasted [107] against the 10 A chromosomes representatives of *B*. *rapa* cv. ‘Chiifu-401’ genome sequence version 1.5 [57]. Only SNPs presenting at least one Blast hit with a minimum of 50% global overlap were considered and their top Blast hit was taken as the SNP physical location. Applying this approach, a total of 5,093 SNPs anchored along the 10 A chromosomes were retrieved for the A_r_A_r’_ and A_r_A_r’_C_o_ F_1_ hybrids combination and 3,636 for the A_n_A_r’_ and A_n_A_r’_C_n_ F_1_ hybrids combination with 1,814 SNPs in common between both combinations.

A second genotyping step was conducted by the GENTYANE platform (INRA, Clermont-Ferrand, France) using the Biomark HD system (Fluidigm technology) and KASPar chemistry [108]. Hybridization were run in two phases according to the GENTYANE platform procedures using the 96.96 Dynamic Array IFC component. Firstly, 672 of the previously retrieved SNPs were selected, prioritizing them so that were well distributed along the 10 A chromosomes of *B*. *rapa* cv. ‘Chiifu-401’ genome sequence and were in common between both F_1_ hybrids combinations. Their primers were synthesized (LGC Genomics, Teddington Middlesex, UK) and hybridizations were run for 2 technical replicates of genomic DNA extracted from each F_1_ hybrid as well as from the *B*. *rapa*, *B*. *oleracea* and *B*. *napus* varieties. The obtained genotyping data were visualized using Fluidigm SNP Genotyping Analysis V4.1.2 software [108] and processed manually. The polymorphisms identified per SNP were then compared to those provided from the *Brassica* 60K Illumina Infinium SNP array revealing 73.2% of accordance (492 SNPs). Secondly, 204 SNPs homogenously distributed along the A genome and with identical polymorphisms between Illumina and KASPar technologies were selected for each F_1_ hybrid combination (S2 Table). Among these selected SNPs, 199 were common between both F_1_ hybrid combinations and five were specific of a single combination but at equivalent positions in order to limit the gap size. From these 204 SNPs, hybridizations were run for the progenies of the AA and AAC F_1_ hybrids including within each 96.96 Dynamic Array IFC component one technical replicate of the F_1_ hybrids as well as the *B*. *rapa*, *B*. *oleracea* and *B*. *napus* varieties. Additionally, among the 204 SNP markers, a technical validation was realized for 38 SNPs for all progenies. The obtained genotyping data were processed as previously described.

### Construction of genetic linkage maps

From the genotyping data processed from progenies of the AA and AAC F_1_ hybrids, only the samples deprived of missing data and repeatable for the 38 SNPs duplicated in the genotyping analysis were retained for linkage analysis. Specifically, for the progenies of female F_1_ hybrids, 429 samples from A_r_A_r’_, 142 from A_r_A_r’_C_o_, 329 from A_n_A_r’_ and 109 from A_n_A_r’_C_n_ were considered. Similarly, for the progenies of male F_1_ hybrids, 298 samples from A_n_A_r’_ and 162 from A_n_A_r’_C_n_ were took into account. Genotyping data obtained for these samples with the 204 corresponding SNP markers are provided in S10–S15 Tables.

Before to realize the linkage analyses, the expected Mendelian segregation on A chromosomes was verified for each SNP marker with chi-squared test at a significance threshold of 5%. The linkage analyses were then performed separately for the genotyping data obtained from each F_1_ hybrid with the 204 corresponding SNPs using CarthaGene software version 1.3 [109]. Firstly, the linkage groups were established with a Logarithm of Odds Score (LOD) threshold of 4.0. The order of the SNPs was then estimated per linkage group by using the multiple 2-point maximum likelihood method at a LOD threshold of 3.0 and a maximum recombination frequency of 0.4. Finally, following the validation of concordant genetic and physical location on the same A chromosome for all the 204 SNPs, the Kosambi function was applied to evaluate the genetic distances in centimorgan (cM) between linked SNP markers [110].

### Statistical analysis

The heterogeneity of CO rates among progenies was assessed for every interval between adjacent SNP markers using a 2-by-2 chi-squared analysis considering a significance threshold of 5%. Additionally, the heterogeneity of CO rates among progenies was evaluated at chromosome and genome scales using 2-by-2 chi-squared tests. For these test, a conservative Bonferroni-corrected threshold of 5% [111] was applied, using the number of intervals between adjacent SNP markers per A chromosomes or for the A genome-wide.

The shapes of recombination landscapes per A chromosome were compared among pairs of maps using the approach developed by Bauer *et al*. [44]. The local CO rates were normalized by the A chromosome-wide rate, allowing us to compare the shape of recombination landscapes regardless of overall variations in genetic lengths. The normalized genetic positions of the SNP markers were determined as a function of their physical positions to obtain the Marey maps. Then, to construct the two landscapes, each chromosome was divided into 10 consecutive bins each containing the same (total over both maps) number of COs and a value for each landscape in each bin was defined by the frequency of COs in that bin. Two-by-2 chi-squared comparisons were applied for the associated coarse-grained landscapes (with 10 bins) and the threshold of 5% was used for the establishment of significant differences at the chromosome wide scale.

The following relationships were studied by regression analyses conducted after the visual validation of the normality of residuals: (i) the average number of COs formed per A chromosome in a meiosis *vs* their physical length covered by SNP markers (in Mbp); (ii) the recombination rates per interval between linked SNP markers (in cM per Mbp) *vs* their physical locations along each of the A chromosomes (in Mbp); (iii) the relative recombination rates normalized per A chromosome (%) *vs* their relative distance from the centromeres (%). For linear (y = ax+b) and order 2 polynomial (y = ax^2^+bx+c) regressions, only the ‘a’ p-values provided by the Fisher-Test were considered. The locations associated to each value of the recombination rates were the middle of the interval of adjacent linked SNP markers. The centromeres’ locations were taken from Mason *et al*. [81].

### Testing post-meiotic selection as the driver of enhanced recombination rates

The recombination rates seen in the progenies of allotriploids could be affected by post-meiotic selection. Clearly, some selection does arise: allotriploids lead to about 50% pollen viability (using aceto-carmine coloration) and produce on average only 2 seeds per pollinated flower (to be compared to 10 in the diploid). Such selection forces could lead to increased CO rates when comparing the progenies to the meiotic products. We thus tested the hypothesis that post-meiotic selection is responsible for the increased recombination rates found in the allotriploids.

The framework we developed for our test consists in considering that a meiotic product is viable only if its number of COs is sufficiently high. Let X be that number. We assume that there is no post-meiotic selection in the diploid case. Let meiosis there lead to a genome-wide genetic length of L_G_ cM (the value measured in the progenies of the diploid hybrid), corresponding to a mean of L_G_ /100 COs per gamete. But when measuring the genome-wide genetic length using the progeny of the allotriploid hybrid, one obtains a larger genetic length, say L_G_' cM, corresponding to a mean of L_G_' /100 COs per (viable) gamete. Recall that this increase is due to having selected gametes having at least X COs. In the absence of CO interference, the number of COs *k* before selection follows a Poisson distribution. It is easy to calculate the mean of *k* after post-meiotic selection. Under the hypothesis being tested, selection strength, that is X, has to be adjusted so that this mean is L_G_' /100 or more. The adjustment is performed numerically. By summing the probabilities in the Poisson distribution when *k* is larger or equal to that value of X, we obtain the fraction of viable gametes produced, *i*.*e*., the fraction surviving the post-meiotic selection in the allotriploid hybrid. Finally, the test of the post-meiotic selection hypothesis is obtained by comparing this predicted fraction (level of selection) to the experimental one. If, as will turn out in practice, this selection intensity is far greater than the experimental one, one can conclude that the post-meiotic selection hypothesis cannot plausibly explain the enhanced CO rates.

For the calculation presented above, we chose a Poisson distribution for *k*, which corresponds to having no CO interference. In reality, the presence of CO interference will reduce the variance of *k*. As a consequence, the fraction of the distribution beyond the value X will be even lower than within the Poisson hypothesis. This means that the selection intensity required with interference would be still more severe, reinforcing our conclusion that the data are not compatible with the selection hypothesis.

### Analysis of distances between adjacent crossovers

One major consequence of CO interference is to change the distribution of distances between adjacent COs (hereafter referred to as ICD, for Inter-CO Distance). In particular, interference lowers the probability of having small ICDs, and reduces the overall variance of ICD distributions. Then, to investigate differences in interference between two populations (for instance diploids and triploids), we first compared their ICD distributions.

CO genetic positions were estimated as the mid-value of the positions of the two flanking markers on the genetic map. ICDs were then calculated for each plant having at least two COs. Comparisons between ICD distributions from two different experimental populations were achieved using Kolmogorov-Smirnov test implemented in the R software (ks.test function).

Each distribution of ICDs was also compared to the corresponding distribution arising in the *absence* of interference. This no-interfernce distribution was obtained by randomly shuffling CO positions over the different gametes. Specifically: (1) for each plant, the number of COs was determined from the experimental data, (2) if this number was greater or equal to two, then the same number of CO positions were randomly drawn from the list of all CO positions of all plants of the population (if two identical CO positions were drawn for the same plant, the drawing for that plant was discarded and repeated until successful), and (3) ICDs were calculated from these shuffled data. In practice, we cumulated the data for (non-interfering) ICDs by repeating the randomization over all plants a total of 1000 times. The resulting distribution of ICDs is then the expectation for what arises in the absence of interference. A proxy for the strength of interference in a cross is the degree of divergence between the distributions of the experimental ICDs and of the ICDs without interference (randomized). Thus, for each population, we calculated the Küllback-Leibler divergence (KL.plugin function from the “entropy” package in R) between those two corresponding distributions.

Then, we asked whether the greater interference strengths found in the diploids compared to the triploids (as indicated by the larger values of the KL divergence) were statistically significant. We thus considered the H_0_ hypothesis that the observed KL divergence values in allotriploids is not smaller than the value in the corresponding diploids. In other words, this H_0_ hypothesis considers that interference is not lower in allotriploids than in diploids, the KL divergence serving here as a proxy for interference strength. To derive a *p*-value for this H_0_ hypothesis in a given genetic background of the A genome, we took the difference between the two Küllback-Leibler divergences (diploid minus allotriploid) as a score, and we compared the experimental value of this score to the distribution of the score under H_0_ obtained by shuffling. This new shuffling was performed as follows. If n_1_ (respectively n_2_) is the number of ICD values obtained from population 1 (respectively population 2), we drew randomly n_1_ values in the pooled list of ICD values of both populations taken together, and we assigned them to population 1, the rest being assigned to population 2. The associated distributions without interference were taken as the weighted average of the distributions without interference obtained from populations 1 and 2, the weight of a population being the proportion of ICD values actually taken in this population by the random drawing procedure. The Küllback-Leibler divergences were then calculated for each of the two (shuffled) populations, and the distribution of the score was obtained by repeating 10^6^ times this shuffling process. The one-sided *p*-value for H_0_ (used for the diploid *vs* allotriploid comparisons) was then taken as the proportion of drawings for which the score was higher than the score obtained experimentally. The two-sided *p*-value for H_0_ (used to compare male *vs* female meiosis and also the two genetic backgrounds) was then taken as the proportion of drawings for which the absolute value of the score was higher than that obtained experimentally.

### Model-based two-pathway analysis of crossover interference

Many quantitative measures of CO interference strength can be defined. Such quantitative frameworks typically rely on mathematical modelling of meiotic processes but then the measured interference strength will depend on the model used. We thus performed our analyses with both of the two most widely used interference models, which are very different in their concept: the *Beam-Film* model and the *Gamma* model. The Beam-Film model [60] is mechanically motivated, based on the idea that the occurrence of a first CO relaxes a mechanical property on the chromosome axis, which prevents a second CO from occurring near the first one. The parameter *Lambda* of the Beam-Film model represents the distance out to which this inhibition acts. The Gamma model [62] is statistically motivated and measures the level of clustering between CO positions in genetic distance. Indeed, stronger interference leads to a deficit in close-by COs and thus to more regularly spaced COs. To take into account the non-interfering pathway of CO formation, it is common practice to use the “sprinkling” procedure whereby non-interfering Class II COs are simply superposed to the Class I COs from the interfering pathway. The interfering pathway is described either by the Beam-Film model or by the Gamma model, and then involves either the parameter *Lambda* or the parameter *nu* to describe the intensity of interference between Class I COs. Furthermore, the proportion of COs formed through the non-interfering pathway is denoted by *p*. To adjust the model parameters to the data, we follow the inference approach described in Falque *et al*. [112], and freely available in Gauthier *et al*. [113]. Confidence intervals on these parameters were obtained using a re-simulation approach as described in Falque *et al*. [112].

### Comparison tests for mean interference strength

Given the values inferred for the interference strength parameter for each cross and chromosome, it is possible to test the H_0_ hypothesis that two crosses have the same mean interference strength. Let V_1_ be the mean interference strength for the first cross, defined as the average over all 10 chromosomes of the inferred interference strengths, and similarly for V_2_. We introduce the score as V2—V_1_ and use 1024 permutations to obtain the exact distribution of this score under H_0_. A one-sided *p*-value is then obtained as the proportion of shuffles having a score greater or equal to the experimental score. We have extended this test to pool together all three pairs of crosses comparing diploids to allotriploids (A_r_A_r'_ (f) *vs* A_r_A_r'_C_o_ (f), A_n_A_r'_ (f) *vs* A_n_A_r'_C_n_ (f), and A_n_A_r'_ (m) *vs* A_n_A_r'_C_n_ (m)). For this extended test, we simply went from 10 to 30 chromosomes, pooling together parameter values from the 10 chromosomes of the 3 crosses. In this case, the larger number of experimental values led us to use 10^5^ permutations in order to obtain the expected distribution of the score under H0.

## Supporting information

S1 Fig Meiotic observations of AA and AAC F_1_ hybrids at metaphase I. (**a**-**d**) Pollen Mother Cells showing ten bivalents for the diploids (**a**) A_r_A_r’_ and (**b**) A_n_A_r’_, or ten bivalents and nine univalents for the allotriploids (**c**) A_r_A_r’_C_o_ and (**d**) A_n_A_r’_C_n_. (**e-j**) FISH analyses for (**e-g**) A_r_A_r’_C_o_ and (**h-j**) A_n_A_r’_C_n_ F_1_ hybrids. BAC FISH was carried out using Bob014O06 and BAC KBrH033J07 which identify all the C chromosomes (**f-i**, green) and the A05 and C04 homoeologous chromosomes (**g-j**, in red), respectively. Univalents are indicated by a red star. Bars, 5 µm.
(TIF)

S2 Fig Recombination rates in Centimorgan (cM) for each of the 10 homologous A chromosomes in AA and AAC F_1_ hybrids. Values obtained for the diploid hybrids are indicated from female A_r_A_r’_ in purple, female A_n_A_r’_ in red, and male A_n_A_r’_ in blue. Values obtained for the allotriploid hybrids are indicated from female A_r_A_r’_C_o_ in light purple, female A_n_A_r’_C_n_ in pink, and male A_n_A_r’_C_n_ in light blue. Statistical differences, providing from a Bonferroni corrected Chi-squared test at a threshold of 5%, are indicated by the letters (a to c).
(TIF)

S3 Fig Frequency of crossovers per chromatid of the progenies deriving from the AA and AAC F_1_ hybrids. Values obtained for the diploid hybrids are indicated from female A_r_A_r’_ in purple, female A_n_A_r’_ in red, and male A_n_A_r’_ in blue. Values obtained for the allotriploid hybrids are indicated from female A_r_A_r’_C_o_ in light purple, female A_n_A_r’_C_n_ in pink, and male A_n_A_r’_C_n_ in light blue.
(TIF)

S4 Fig Illustration of the statistical test used to compare recombination landscapes between the AA and AAC F_1_ hybrids along the 10 homologous A chromosomes. The chromosome length is divided into 10 bins whose length is set to ensure that all bins contain the same total number of crossover when pooling data of both populations. Solid colored lines represent the Marey maps normalized to a total arbitrary length of 100 to focus on differences in the shape of the recombination landscapes and not on differences in the values of chromosome genetic lengths. Dashed colored lines represent derivative of the Marey maps, indicating local recombination rate in cM per Mbp. Vertical colored bars indicate 95% confidence intervals of recombination rates in cM per Mbp for each map over each bin. Heavy black bars represent average recombination rate in cM per Mbp over both maps for each bin. Black arrows connect the average recombination rates of the two maps over each bin.
(PDF)

S5 Fig Illustration of the statistical test used to compare recombination landscapes between male and female meiosis of F_1_ hybrids along the 10 homologous A chromosomes. The chromosome length is divided into 10 bins whose length is set to ensure that all bins contain the same total number of crossover when pooling data of both populations. Solid colored lines represent the Marey maps normalized to a total arbitrary length of 100 to focus on differences in the shape of the recombination landscapes and not on differences in the values of chromosome genetic lengths. Dashed colored lines represent derivative of the Marey maps, indicating local recombination rate in cM per Mbp. Vertical colored bars indicate 95% confidence intervals of recombination rates in cM per Mbp for each map over each bin. Heavy black bars represent average recombination rate in cM per Mbp over both maps for each bin. Black arrows connect the average recombination rates of the two maps over each bin.
(PDF)

S6 Fig Illustration of the statistical test used to compare recombination landscapes between the two genetic backgrounds of F_1_ hybrids along the 10 homologous A chromosomes. The chromosome length is divided into 10 bins whose length is set to ensure that all bins contain the same total number of crossover when pooling data of both populations. Solid colored lines represent the Marey maps normalized to a total arbitrary length of 100 to focus on differences in the shape of the recombination landscapes and not on differences in the values of chromosome genetic lengths. Dashed colored lines represent derivative of the Marey maps, indicating local recombination rate in cM per Mbp. Vertical colored bars indicate 95% confidence intervals of recombination rates in cM per Mbp for each map over each bin. Heavy black bars represent average recombination rate in cM per Mbp over both maps for each bin. Black arrows connect the average recombination rates of the two maps over each bin.
(PDF)

S7 Fig Circos diagram comparing the recombination rates along the 10 A chromosomes in cM per Mbp between the AA F_1_ hybrids. In the first outer circle are represented the 10 A chromosomes of the *B*. *rapa* cv. ‘Chiifu-401’ genome sequence version 1.5 [57]. Their sizes are indicated by the values in megabase pairs above each chromosome, and a ruler drawn underneath each chromosome, with larger and smaller tick marks every 10 and 2 Mbp, respectively. In the second outer circle, is detailed the architecture of each A chromosome, including the genes and transposable elements (TEs) densities from the version 1.5 of the *B*. *rapa* cv. ‘Chiifu-401’ genome sequence [57]. The active centromeres are delimited in black using the positions established by Mason *et al*. [81]. In the third outer circle, are indicated the positions of the 204 SNP markers used for the genotyping of the progenies of each AA F_1_ hybrid. In the two inner circles, are represented the pair-wise comparisons for the recombination landscapes (in cM per Mb) of progenies deriving from the AA F_1_ hybrids. Toward the Circos diagram center, are compared (i) the A_r_A_r’_ (purple lines) and A_n_A_r’_ (red lines) female hybrids, and (ii) the female A_n_A_r’_ (red lines) and male A_n_A_r’_ (blue lines) hybrids. For each interval between adjacent SNP markers, the heterogeneity of CO rates was assessed using Chi-squared tests and significant differences at a threshold of 5% were indicated for each pair-wise comparison between AA F_1_ hybrids in grey.
(TIF)

S8 Fig Circos diagram comparing the recombination rates along the 10 A chromosomes in cM per Mbp between the AAC F_1_ hybrids. In the first outer circle are represented the 10 A chromosomes of the *B*. *rapa* cv. ‘Chiifu-401’ genome sequence version 1.5 [57]. Their sizes are indicated by the values in megabase pairs above each chromosome, and a ruler drawn underneath each chromosome, with larger and smaller tick marks every 10 and 2 Mbp, respectively. In the second outer circle, is detailed the architecture of each A chromosome, including the genes and transposable elements (TEs) densities from the version 1.5 of the *B*. *rapa* cv. ‘Chiifu-401’ genome sequence [57]. The active centromeres are delimited in black using the positions established by Mason *et al*. [81]. In the third outer circle, are indicated the positions of the 204 SNP markers used for the genotyping of the progenies of each AAC F_1_ hybrid. In the two inner circles, are represented the pair-wise comparisons for the recombination landscapes (in cM per Mb) of progenies deriving from the AAC F_1_ hybrids. Toward the Circos diagram center, are compared (i) the A_r_A_r’_C_o_ (light purple lines) and A_n_A_r’_C_n_ (pink lines) female hybrids, and (ii) the female A_n_A_r’_C_n_ (pink lines) and male A_n_A_r’_C_n_ (light blue lines) hybrids. For each interval between adjacent SNP markers, the heterogeneity of CO rates was assessed using Chi-squared tests and significant differences at a threshold of 5% were indicated for each pair-wise comparison between AAC F_1_ hybrids in grey.
(TIF)

S9 Fig Relationship between the relative recombination rates normalized per A chromosome (%) and their relative distance from the centromeres (%) for each AA and AAC F_1_ hybrid. Female A_r_A_r’_ (red circles): y = 0,135257x-1,195111; R^2^ = 0.53. Female A_r_A_r’_C_o_ (pink squares): y = 0,027539x+3,861802; R^2^ = 0.09. Female A_n_A_r’_ (red circles): y = 0,117461x-0,360060; R^2^ = 0.48. Female A_n_A_r’_C_n_ (pink squares): y = 0,03699x+3,41789; R^2^ = 0.15. Male A_n_A_r’_ (blue circles): y = 0,113281x-0,163805; R^2^ = 0.51. Male A_n_A_r’_C_n_ (light blue squares): y = 0,035645x-3,481115; R^2^ = 0.10.
(TIF)

S10 Fig Illustration of the relationships between the recombination rates per interval between linked SNP markers (in cM per Mbp) and their physical locations along each of the 10 A chromosomes (in Mbp) per AA and AAC F_1_ hybrid. The p-values and R^2^ are indicated in S7 Table.
(PDF)

S11 Fig Distributions of inter-crossover genetic distances in AA and AAC F_1_ hybrids for individual chromosomes. Comparison of the distribution of genetic distances between successive COs from populations deriving of females A_r_A_r’_ (in purple) *vs* A_r_A_r’_C_o_ (in light purple), females A_n_A_r’_ (in red) *vs* A_n_A_r’_C_n_ (in pink) and males A_n_A_r’_ (in blue) *vs* A_n_A_r’_C_n_ (in light blue). X-axis: genetic distance between successive COs. Solid lines correspond to experimental data. Dashed lines indicate the corresponding distributions in the "no-interference" situation, obtained by re-shuffling CO positions of experimental data (see Methods). For each population, the Küllback-Leibler divergence (KL Div.) from the experimental to the "no-interference" distribution provides a quantitative measurement of interference strength. p-value: one-sided *p*-value of the H_O_ hypothesis that the diploids and triploids have the same the KL Div. index (and thus interference strength for the considered chromosome). Sufficiently small values indicate significantly higher interference in diploids than in allotriploids (see details in Methods).
(PDF)

S12 Fig Distributions of inter-crossover genetic distances in the two genetic backgrounds of F_1_ hybrids when pooling all 10 chromosomes. Comparison of the distribution of genetic distances between successive COs from populations deriving of females A_r_A_r’_ (in purple) *vs* A_n_A_r’_ (in red), and females A_r_A_r’_C_o_ (in light purple) *vs* A_n_A_r’_C_n_ (in pink). Data are pooled over the 10 A chromosomes. X-axis: genetic distance between successive COs. Solid lines correspond to experimental data. Dashed lines indicate the corresponding distributions in the "no-interference" situation, obtained by re-shuffling CO positions of experimental data (see Methods). For each population, the Küllback-Leibler divergence (KL Div.) from the experimental to the "no-interference" distribution provides a quantitative measurement of interference strength. p-value: two-sided *p*-value of the H_O_ hypothesis that the ArAr' and AnAr' hybrids have the same the KL Div. index (and thus interference strength). Sufficiently small values indicate significantly different interference in A_r_A_r'_ and A_n_A_r'_ hybrids (see details in Methods).
(PDF)

S13 Fig Distributions of inter-crossover genetic distances in male and female meiosis of F_1_ hybrids when pooling all 10 chromosomes. Comparison of the distribution of genetic distances between successive COs from populations deriving of female A_n_A_r’_ (in red) *vs* male A_n_A_r’_ (in blue), and female A_n_A_r’_C_n_ (in pink) *vs* male A_n_A_r’_C_n_ (in light blue). Data are pooled over the 10 A chromosomes. X-axis: genetic distance between successive COs. Solid lines correspond to experimental data. Dashed lines indicate the corresponding distributions in the "no-interference" situation, obtained by re-shuffling CO positions of experimental data (see Methods). For each population, the Küllback-Leibler divergence (KL Div.) from the experimental to the "no-interference" distribution provides a quantitative measurement of interference strength. p-value: two-sided *p*-value of the H_O_ hypothesis that the male and female meioses have the same the KL Div. index (and thus interference strength). Sufficiently small values indicate significantly different interference in female than in male meiosis of allotriploid hybrid (see details in Methods).
(PDF)

S1 Table Meiotic behavior established from Pollen Mother Cells of each AA and AAC F_1_ hybrid produced. % Cells: percentage of cells with the expected behavior. I and II are respectively univalents and bivalents.
(XLSX)

S2 Table Characteristics of SNP markers genetically mapped on the 10 homologous A chromosomes of each of the AA and AAC F_1_ hybrids combinations. (XLSX)

S3 Table Statistical comparisons of the shapes of recombination landscapes along the homologous A chromosomes in AA and AAC F_1_ hybrids. The line "ALL" in the column "Chromosomes" correspond to the pooled analysis of the 10 chromosomes together. The "pvalue" column is the p-value for the H0 hypothesis that the shapes of the recombination landscapes along the chromosome are identical in both maps (recombination landscapes are normalized, so only differences in the shapes of the landscapes and not in the chromosome-wide values of recombination rates are examined).
(XLSX)

S4 Table Statistical comparisons of the shapes of recombination landscapes along the homologous A chromosomes in male and female meiosis. The line "ALL" in the column "Chromosomes" correspond to the pooled analysis of the 10 chromosomes together. The "pvalue" column is the p-value for the H0 hypothesis that the shapes of the recombination landscapes along the chromosome are identical in both maps (recombination landscapes are normalized, so only differences in the shapes of the landscapes and not in the chromosome-wide values of recombination rates are examined).
(XLSX)

S5 Table Statistical comparisons of the shapes of recombination landscapes along the homologous A chromosomes in the two genetic backgrounds. The line "ALL" in the column "Chromosomes" correspond to the pooled analysis of the 10 chromosomes together. The "pvalue" column is the p-value for the H0 hypothesis that the shapes of the recombination landscapes along the chromosome are identical in both maps (recombination landscapes are normalized, so only differences in the shapes of the landscapes and not in the chromosome-wide values of recombination rates are examined).
(XLSX)

S6 Table Chi-squared comparisons in the crossover rate heterogeneity per interval between adjacent linked SNP markers. (XLSX)

S7 Table Relationship between the recombination rates between adjacent SNP markers and their physical locations along each of the homologous A chromosomes in AA and AAC F_1_ hybrids. For the line "ALL" in the column "Chromosomes", all the relative recombination rates between adjacent SNP markers were considered, and normalized per A chromosome, to test the relationship with their relative distance from the centromeres. The P-value for the regression analyses are indicated by: NS (Not Significant) > 0.05 > * > 0.01 > ** > 0.001> ***.
(XLSX)

S8 Table Statistical comparisons of distributions of distances between successive COs in AA and AAC F_1_ hybrids. K-S test: Kolmogorov-Smirnov test for differences between the two experimental distributions. K-L Div. Kullback-Leibler divergence between the experimental distribution and the « no-interference » distribution. K-L Div. p-value: p-value for the H0 hypothesis that the K-L divergence is the same in diploids and allotriploids (see Methods).
(XLSX)

S9 Table Quantification of CO interference parameters using the Gamma-sprinkling and Beam-Film models for each of the AA and AAC F_1_ hybrids. The line "Average" in the column "Chromosome" corresponds to the average of the values obtained for the 10 chromosomes. The nu and Lambda parameters correspond to the strength of CO interference, and p to the proportion of CO formed through the non-interfering pathway (Type II COs). _Inf and _Sup suffixes indicate 95% confidence intervals.
(XLSX)

S10 Table Genotyping data for the samples deriving from the female A_r_A_r'_ F_1_ hybrid used for linkage analysis. (XLSX)

S11 Table Genotyping data for the samples deriving from the female A_r_A_r'_C_o_ F_1_ hybrid used for linkage analysis. (XLSX)

S12 Table Genotyping data for the samples deriving from the female A_n_A_r'_ F_1_ hybrid used for linkage analysis. (XLSX)

S13 Table Genotyping data for the samples deriving from the female A_n_A_r'_C_n_ F_1_ hybrid used for linkage analysis. (XLSX)

S14 Table Genotyping data for the samples deriving from the male A_n_A_r'_ F_1_ hybrid used for linkage analysis. (XLSX)

S15 Table Genotyping data for the samples deriving from the male A_n_A_r'_C_n_ F_1_ hybrid used for linkage analysis. (XLSX)
