# Supplementary figures and images for: Amplifying recombination genome-wide and reshaping crossover landscapes in Brassicas
Source: PLoS Genet. 2017 May 11;13(5):e1006794. doi: 10.1371/journal.pgen.1006794 (PMC5444851; doi:10.1371/journal.pgen.1006794)

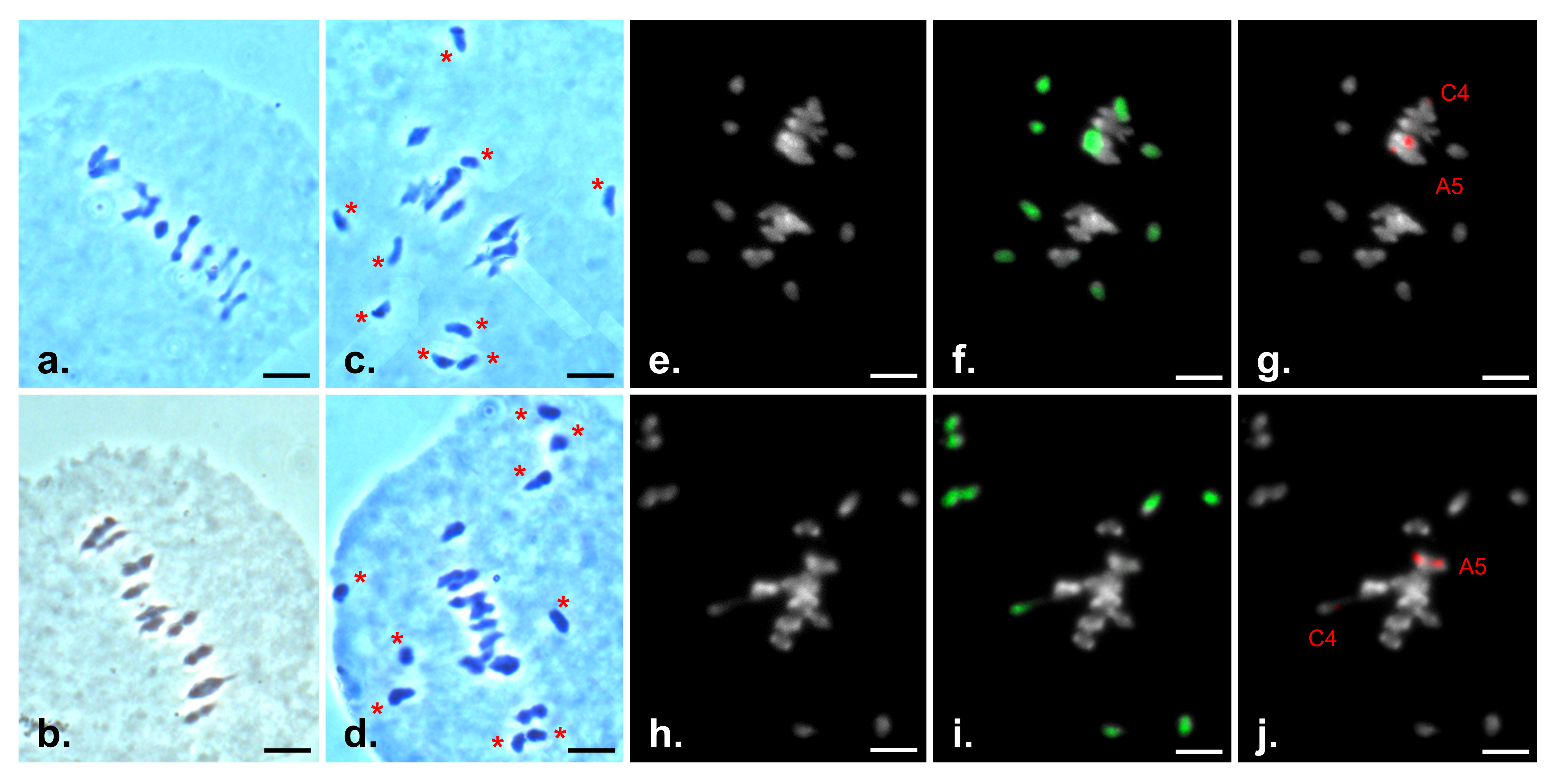

Supplement: S1 Fig — (a-d) Pollen Mother Cells showing ten bivalents for the diploids (a) ArAr’ and (b) AnAr’, or ten bivalents and nine univalents for the allotriploids (c) ArAr’Co and (d) AnAr’Cn. (e-j) FISH analyses for (e-g) ArAr’Co and (h-j) AnAr’Cn F1 hybrids. BAC FISH was carried out using Bob014O06 and BAC KBrH033J07 which identify all the C chromosomes (f-i, green) and the A05 and C04 homoeologous chromosomes (g-j, in red), respectively. Univalents are indicated by a red star. Bars, 5 µm. (TIF) [file pgen.1006794.s001.tif]

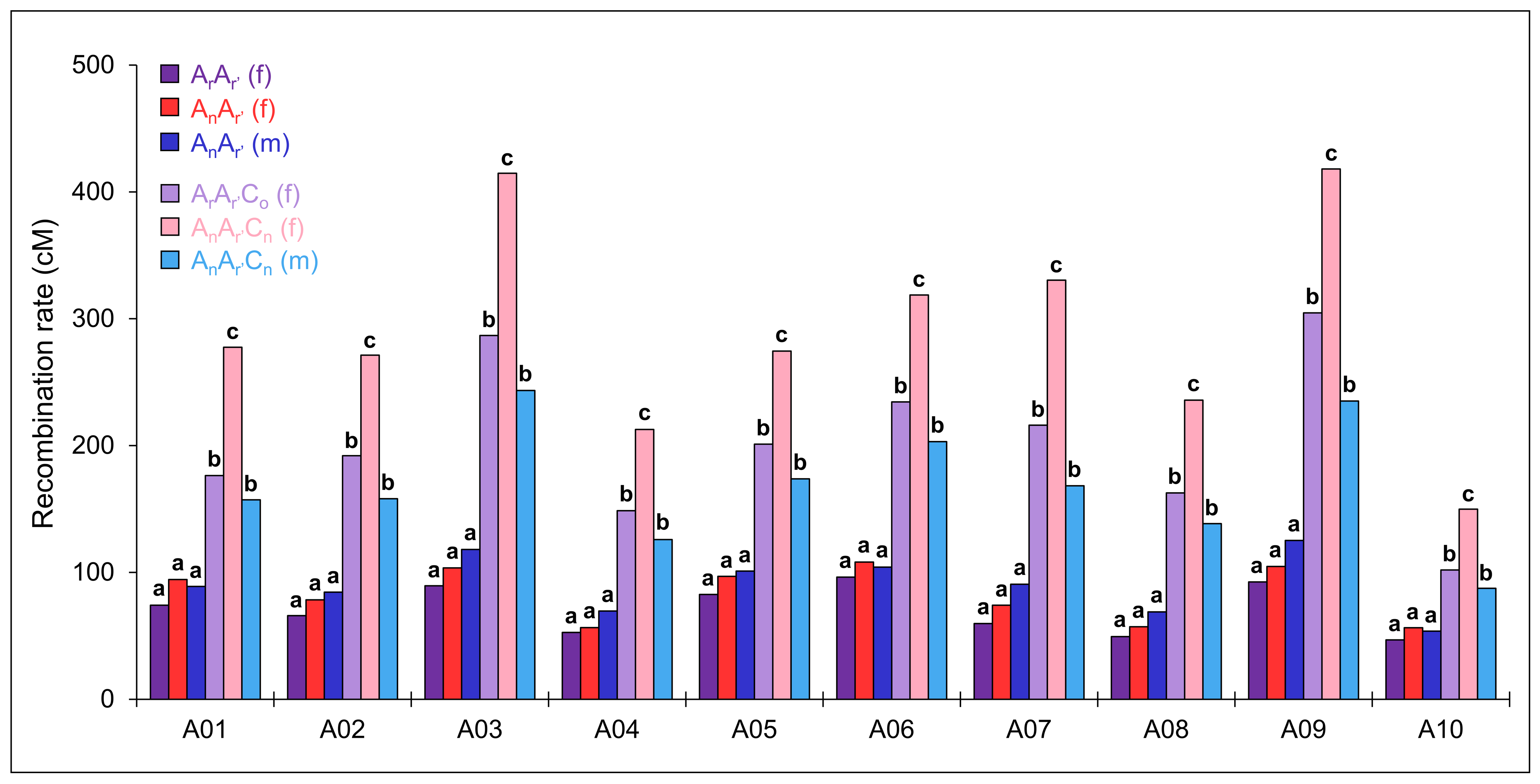

Supplement: S2 Fig — Values obtained for the diploid hybrids are indicated from female ArAr’ in purple, female AnAr’ in red, and male AnAr’ in blue. Values obtained for the allotriploid hybrids are indicated from female ArAr’Co in light purple, female AnAr’Cn in pink, and male AnAr’Cn in light blue. Statistical differences, providing from a Bonferroni corrected Chi-squared test at a threshold of 5%, are indicated by the letters (a to c). (TIF) [file pgen.1006794.s002.tif]

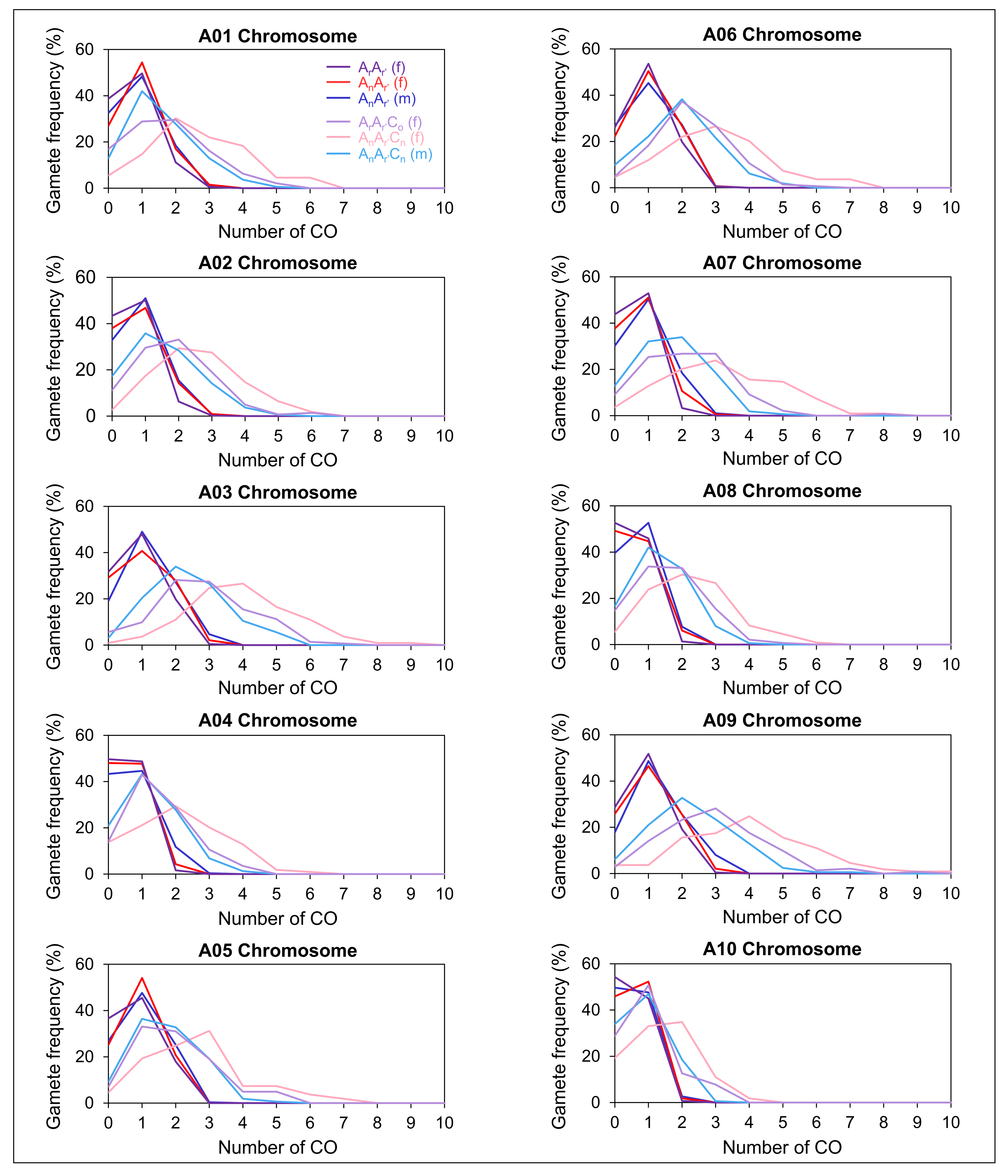

Supplement: S3 Fig — Values obtained for the diploid hybrids are indicated from female ArAr’ in purple, female AnAr’ in red, and male AnAr’ in blue. Values obtained for the allotriploid hybrids are indicated from female ArAr’Co in light purple, female AnAr’Cn in pink, and male AnAr’Cn in light blue. (TIF) [file pgen.1006794.s003.tif]

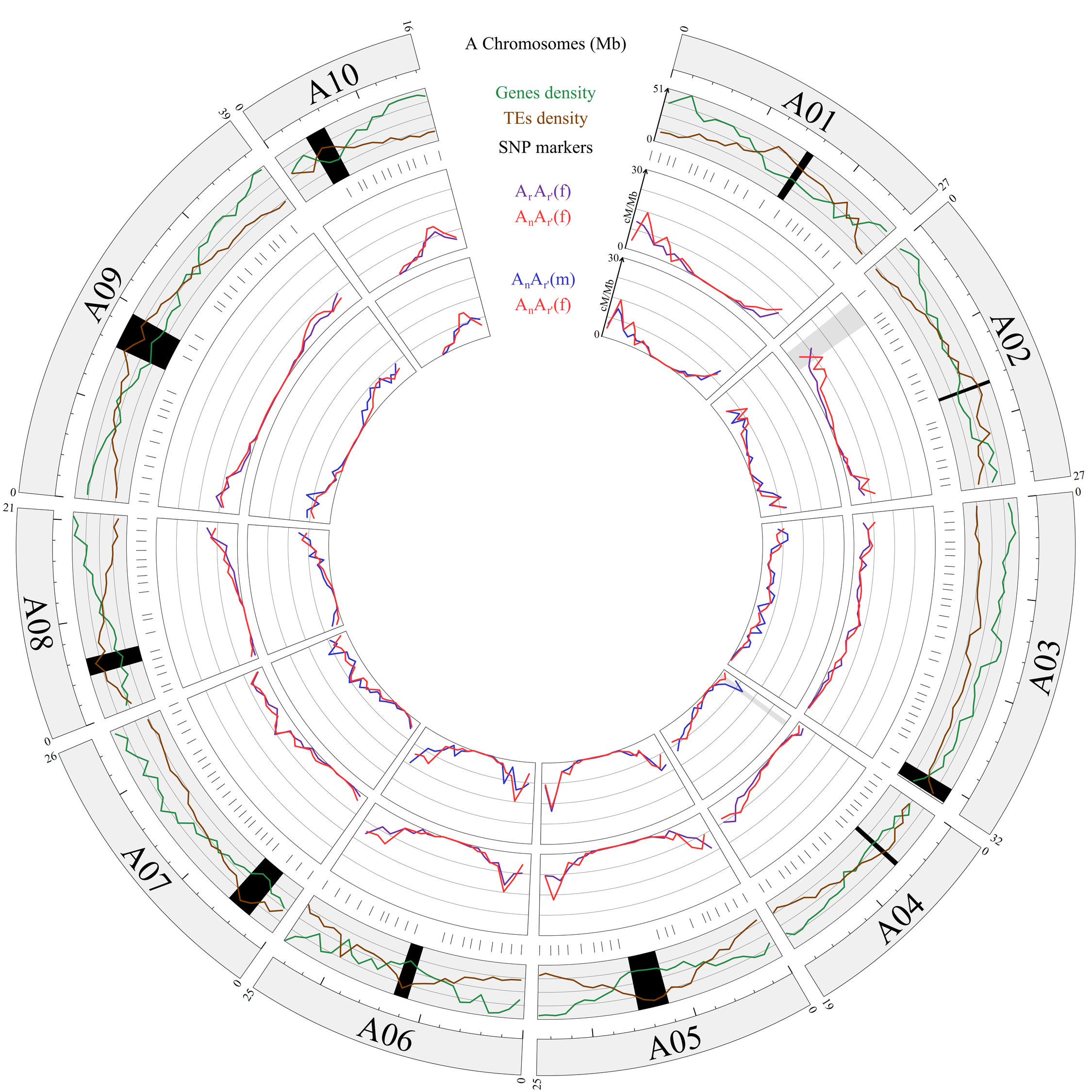

Supplement: S7 Fig — In the first outer circle are represented the 10 A chromosomes of the B. rapa cv. ‘Chiifu-401’ genome sequence version 1.5 [57]. Their sizes are indicated by the values in megabase pairs above each chromosome, and a ruler drawn underneath each chromosome, with larger and smaller tick marks every 10 and 2 Mbp, respectively. In the second outer circle, is detailed the architecture of each A chromosome, including the genes and transposable elements (TEs) densities from the version 1.5 of the B. rapa cv. ‘Chiifu-401’ genome sequence [57]. The active centromeres are delimited in black using the positions established by Mason et al. [81]. In the third outer circle, are indicated the positions of the 204 SNP markers used for the genotyping of the progenies of each AA F1 hybrid. In the two inner circles, are represented the pair-wise comparisons for the recombination landscapes (in cM per Mb) of progenies deriving from the AA F1 hybrids. Toward the Circos diagram center, are compared (i) the ArAr’ (purple lines) and AnAr’ (red lines) female hybrids, and (ii) the female AnAr’ (red lines) and male AnAr’ (blue lines) hybrids. For each interval between adjacent SNP markers, the heterogeneity of CO rates was assessed using Chi-squared tests and significant differences at a threshold of 5% were indicated for each pair-wise comparison between AA F1 hybrids in grey. (TIF) [file pgen.1006794.s007.tif]

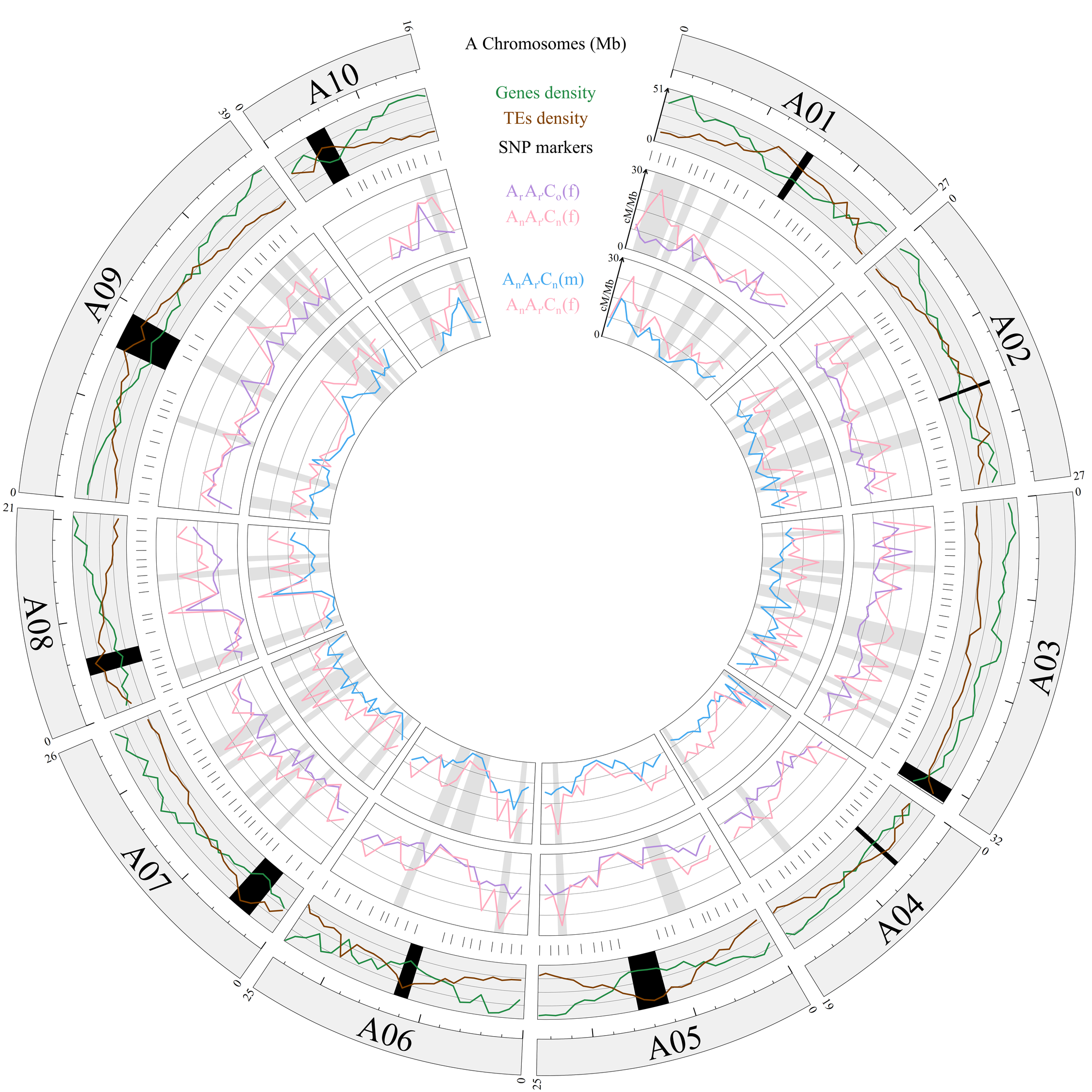

Supplement: S8 Fig — In the first outer circle are represented the 10 A chromosomes of the B. rapa cv. ‘Chiifu-401’ genome sequence version 1.5 [57]. Their sizes are indicated by the values in megabase pairs above each chromosome, and a ruler drawn underneath each chromosome, with larger and smaller tick marks every 10 and 2 Mbp, respectively. In the second outer circle, is detailed the architecture of each A chromosome, including the genes and transposable elements (TEs) densities from the version 1.5 of the B. rapa cv. ‘Chiifu-401’ genome sequence [57]. The active centromeres are delimited in black using the positions established by Mason et al. [81]. In the third outer circle, are indicated the positions of the 204 SNP markers used for the genotyping of the progenies of each AAC F1 hybrid. In the two inner circles, are represented the pair-wise comparisons for the recombination landscapes (in cM per Mb) of progenies deriving from the AAC F1 hybrids. Toward the Circos diagram center, are compared (i) the ArAr’Co (light purple lines) and AnAr’Cn (pink lines) female hybrids, and (ii) the female AnAr’Cn (pink lines) and male AnAr’Cn (light blue lines) hybrids. For each interval between adjacent SNP markers, the heterogeneity of CO rates was assessed using Chi-squared tests and significant differences at a threshold of 5% were indicated for each pair-wise comparison between AAC F1 hybrids in grey. (TIF) [file pgen.1006794.s008.tif]

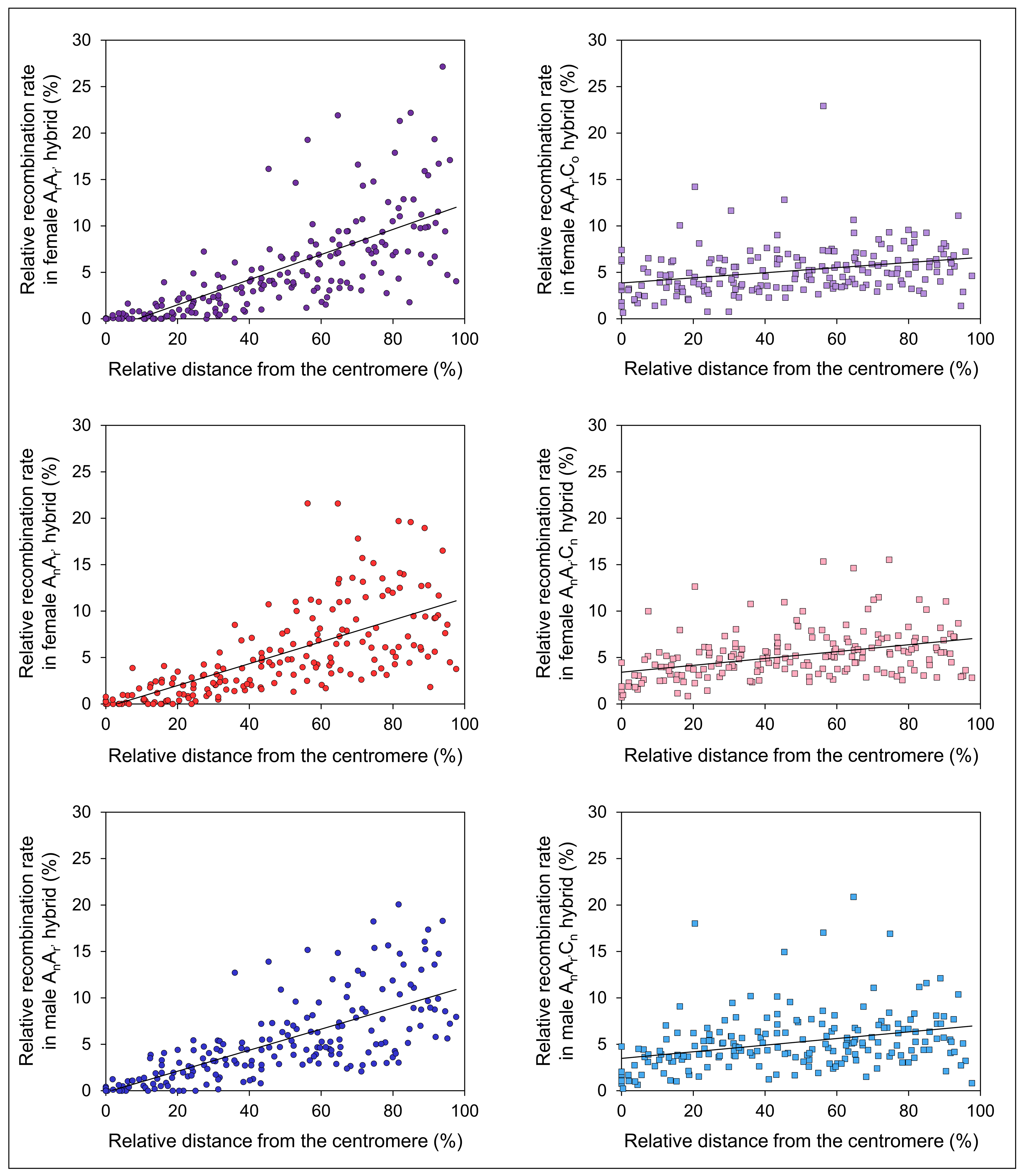

Supplement: S9 Fig — Female ArAr’ (red circles): y = 0,135257x-1,195111; R2 = 0.53. Female ArAr’Co (pink squares): y = 0,027539x+3,861802; R2 = 0.09. Female AnAr’ (red circles): y = 0,117461x-0,360060; R2 = 0.48. Female AnAr’Cn (pink squares): y = 0,03699x+3,41789; R2 = 0.15. Male AnAr’ (blue circles): y = 0,113281x-0,163805; R2 = 0.51. Male AnAr’Cn (light blue squares): y = 0,035645x-3,481115; R2 = 0.10. (TIF) [file pgen.1006794.s009.tif]

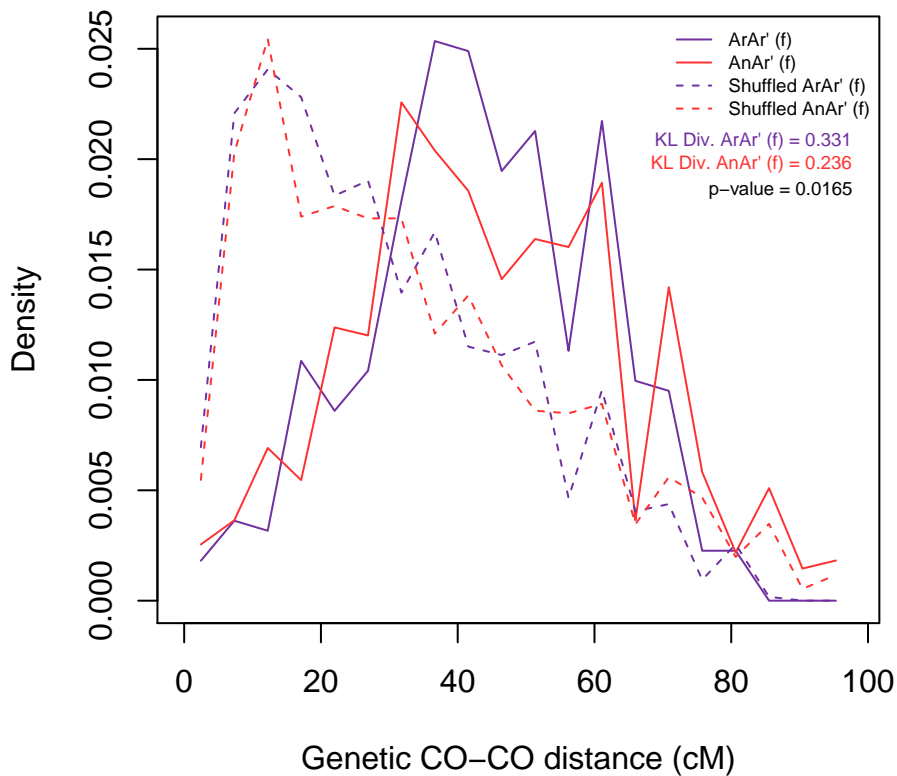

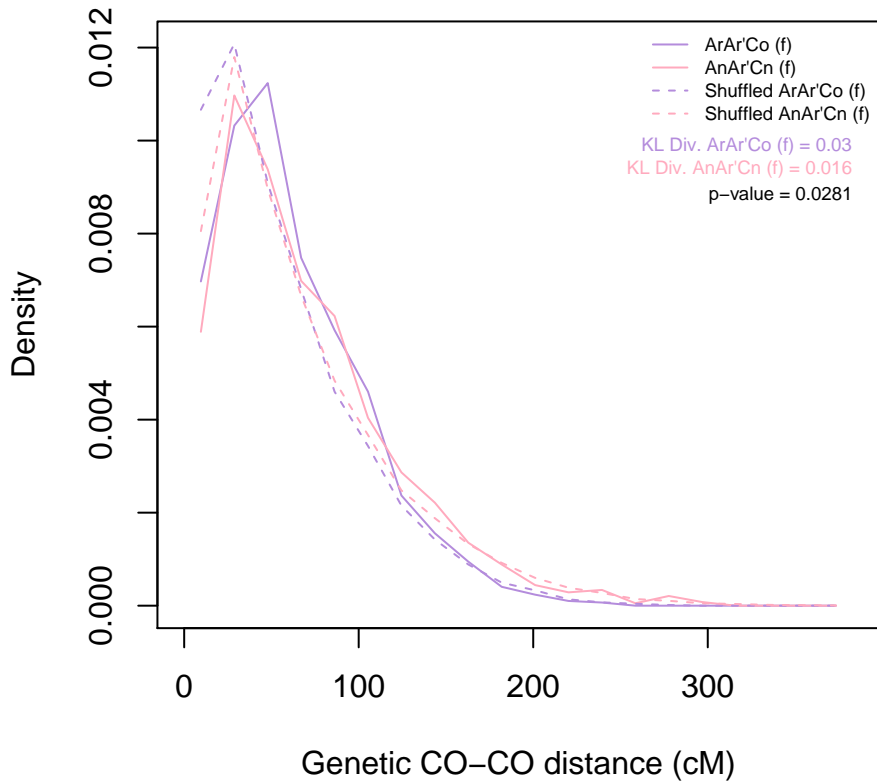

Supplement: S12 Fig — Comparison of the distribution of genetic distances between successive COs from populations deriving of females ArAr’ (in purple) vs AnAr’ (in red), and females ArAr’Co (in light purple) vs AnAr’Cn (in pink). Data are pooled over the 10 A chromosomes. X-axis: genetic distance between successive COs. Solid lines correspond to experimental data. Dashed lines indicate the corresponding distributions in the "no-interference" situation, obtained by re-shuffling CO positions of experimental data (see Methods). For each population, the Küllback-Leibler divergence (KL Div.) from the experimental to the "no-interference" distribution provides a quantitative measurement of interference strength. p-value: two-sided p-value of the HO hypothesis that the ArAr' and AnAr' hybrids have the same the KL Div. index (and thus interference strength). Sufficiently small values indicate significantly different interference in ArAr' and AnAr' hybrids (see details in Methods). (PDF) [file pgen.1006794.s012.pdf]

Density

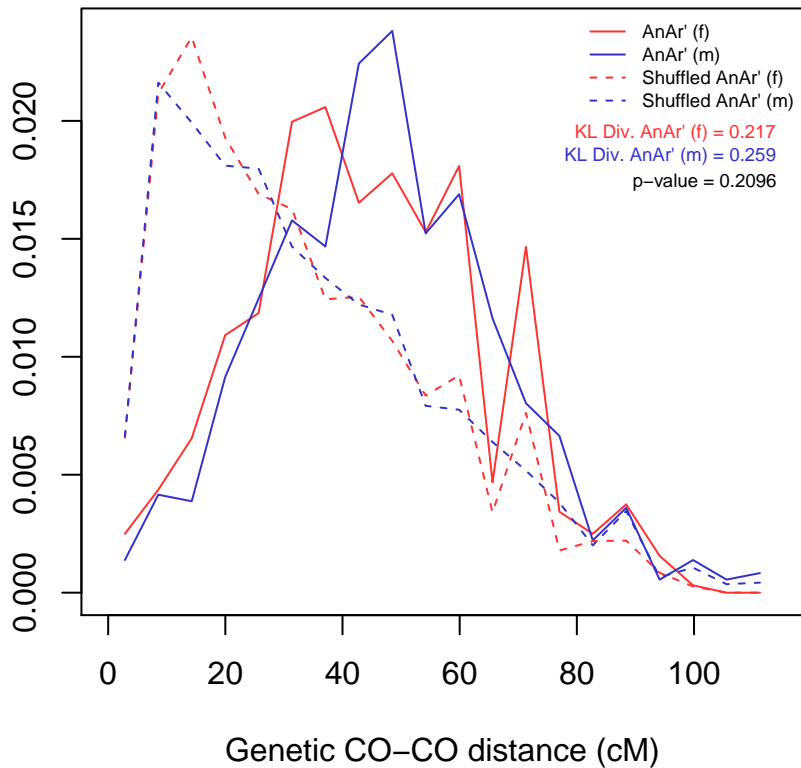

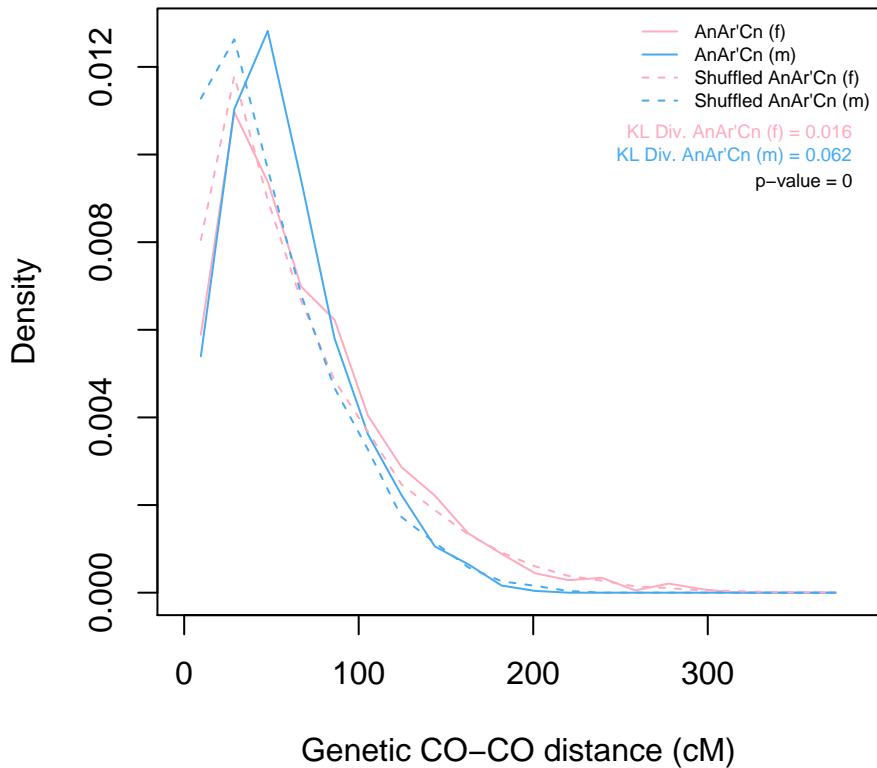

Supplement: S13 Fig — Comparison of the distribution of genetic distances between successive COs from populations deriving of female AnAr’ (in red) vs male AnAr’ (in blue), and female AnAr’Cn (in pink) vs male AnAr’Cn (in light blue). Data are pooled over the 10 A chromosomes. X-axis: genetic distance between successive COs. Solid lines correspond to experimental data. Dashed lines indicate the corresponding distributions in the "no-interference" situation, obtained by re-shuffling CO positions of experimental data (see Methods). For each population, the Küllback-Leibler divergence (KL Div.) from the experimental to the "no-interference" distribution provides a quantitative measurement of interference strength. p-value: two-sided p-value of the HO hypothesis that the male and female meioses have the same the KL Div. index (and thus interference strength). Sufficiently small values indicate significantly different interference in female than in male meiosis of allotriploid hybrid (see details in Methods). (PDF) [file pgen.1006794.s013.pdf]
