# Supplementary material for: Amplifying recombination genome-wide and reshaping crossover landscapes in Brassicas
Source: PLoS Genet. 2017 May 11;13(5):e1006794. doi: 10.1371/journal.pgen.1006794 (PMC5444851; doi:10.1371/journal.pgen.1006794)
Supplement: S4 Fig — The chromosome length is divided into 10 bins whose length is set to ensure that all bins contain the same total number of crossover when pooling data of both populations. Solid colored lines represent the Marey maps normalized to a total arbitrary length of 100 to focus on differences in the shape of the recombination landscapes and not on differences in the values of chromosome genetic lengths. Dashed colored lines represent derivative of the Marey maps, indicating local recombination rate in cM per Mbp. Vertical colored bars indicate 95% confidence intervals of recombination rates in cM per Mbp for each map over each bin. Heavy black bars represent average recombination rate in cM per Mbp over both maps for each bin. Black arrows connect the average recombination rates of the two maps over each bin. (PDF) [file pgen.1006794.s004.pdf]

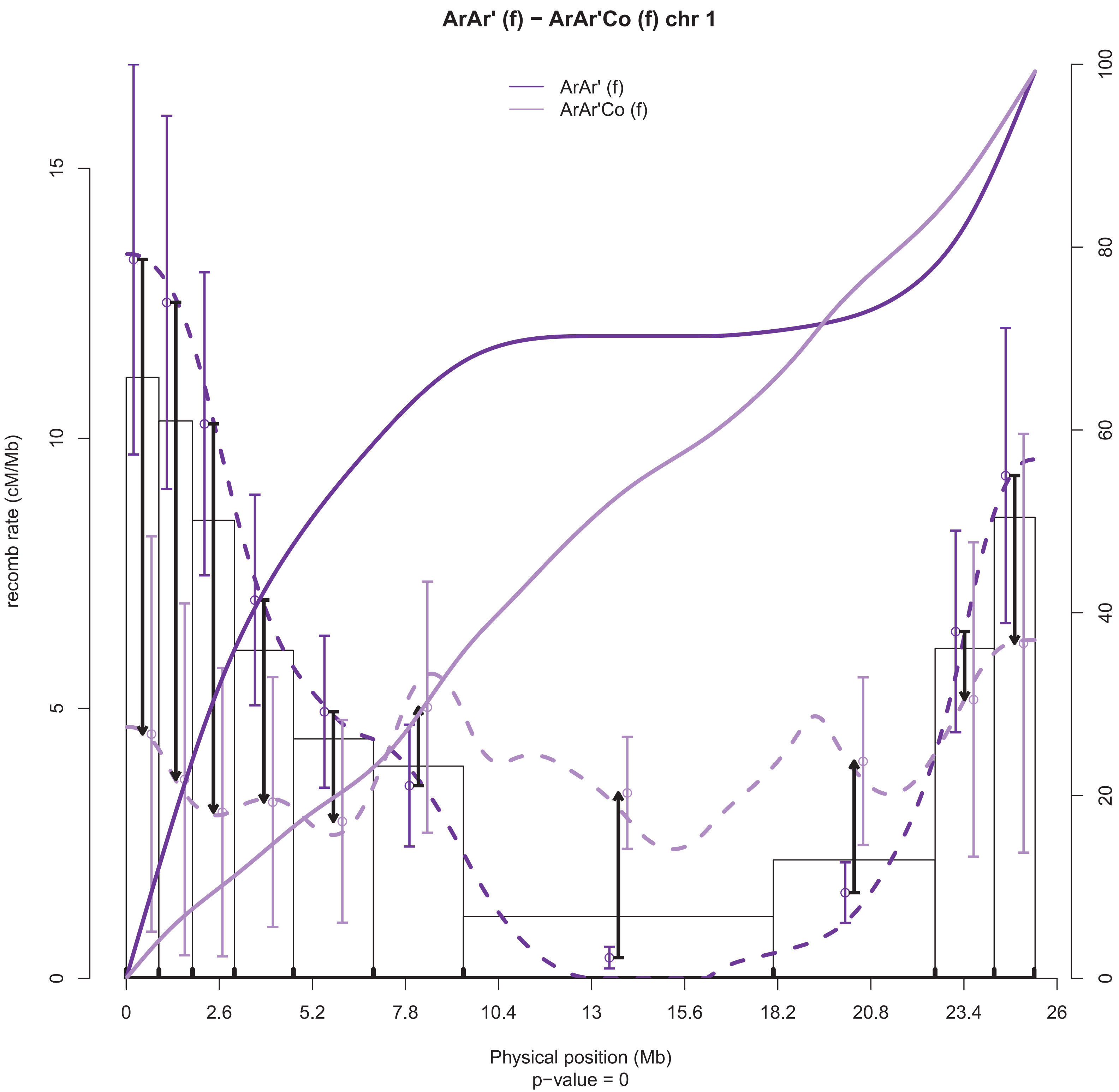

ArAr' (f) − ArAr'Co (f) chr 1
ArAr' (f)
ArAr'Co (f)
recomb rate (cM/Mb)
0
5
10
15
0
20
40
60
80
100
0
2.6
5.2
7.8
10.4
13
15.6
18.2
20.8
23.4
26
Physical position (Mb)
p−value = 0

ArAr' (f) − ArAr'Co (f) chr 2

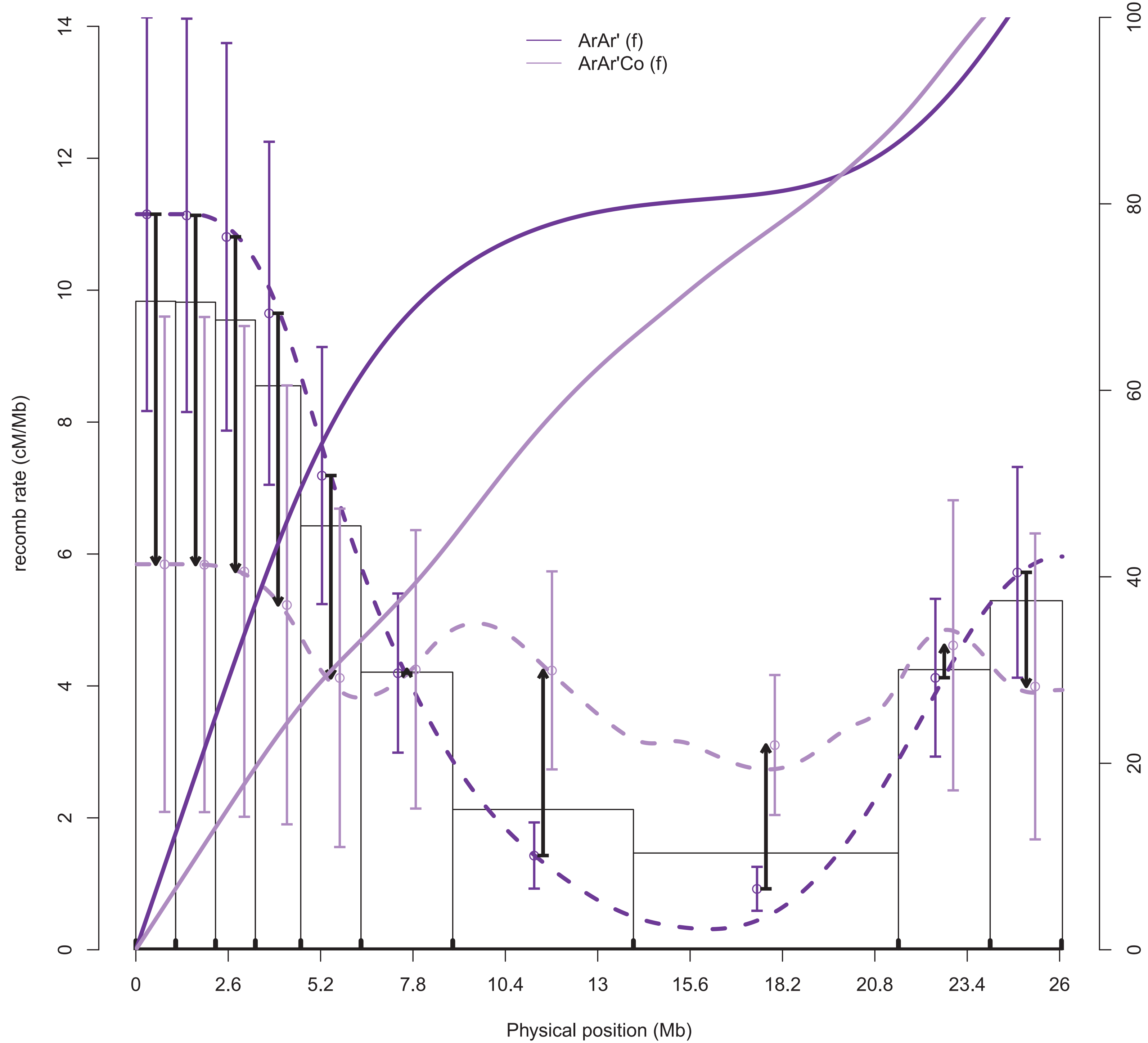

p−value = 9.37932509437189e−10

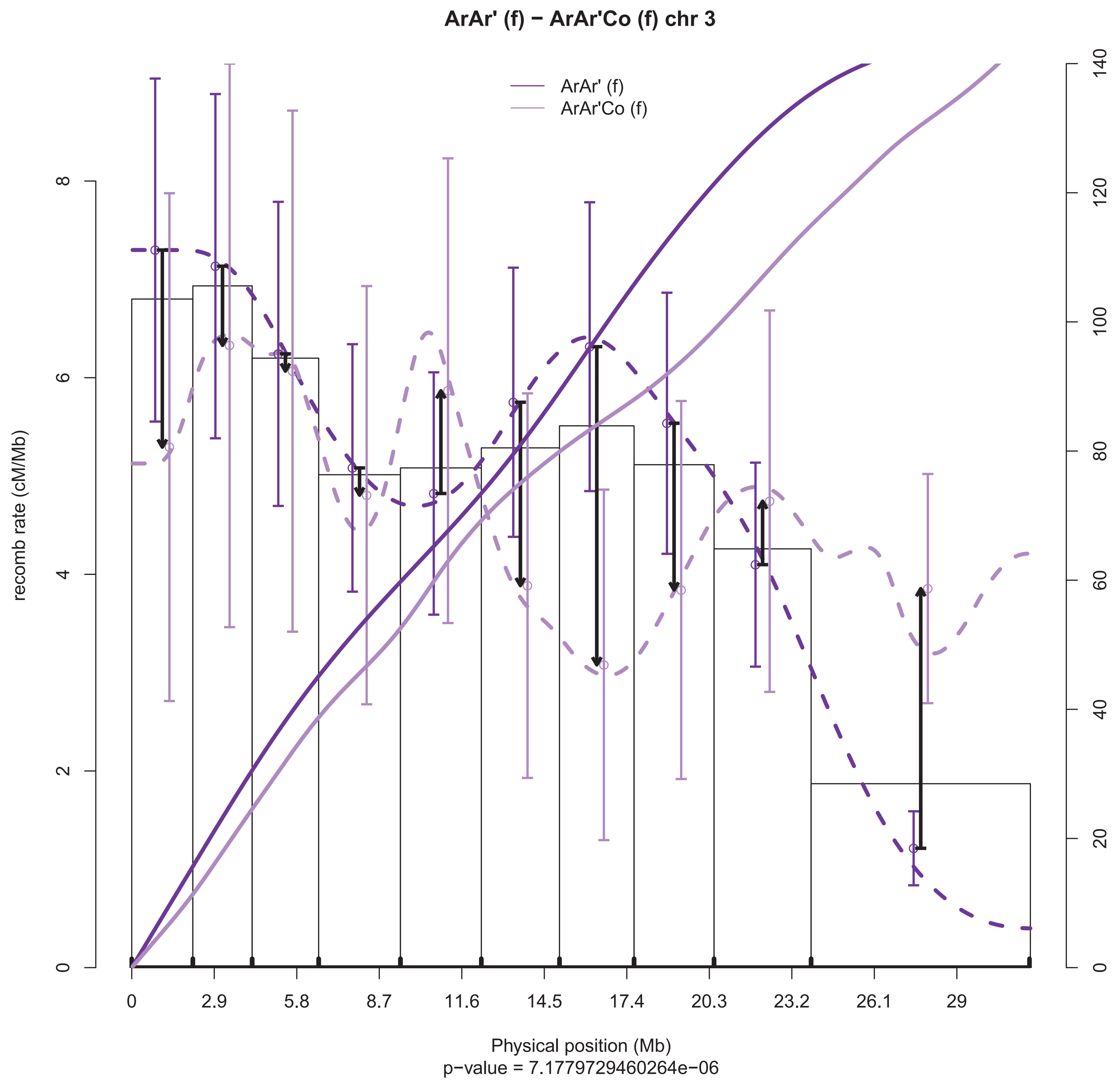
ArAr' (f) − ArAr'Co (f) chr 3
ArAr' (f)
ArAr'Co (f)
recomb rate (cM/Mb)
0
2
4
6
8
0
20
40
60
80
100
120
140
0
2.9
5.8
8.7
11.6
14.5
17.4
20.3
23.2
26.1
29
Physical position (Mb)
p−value = 7.1779729460264e−06

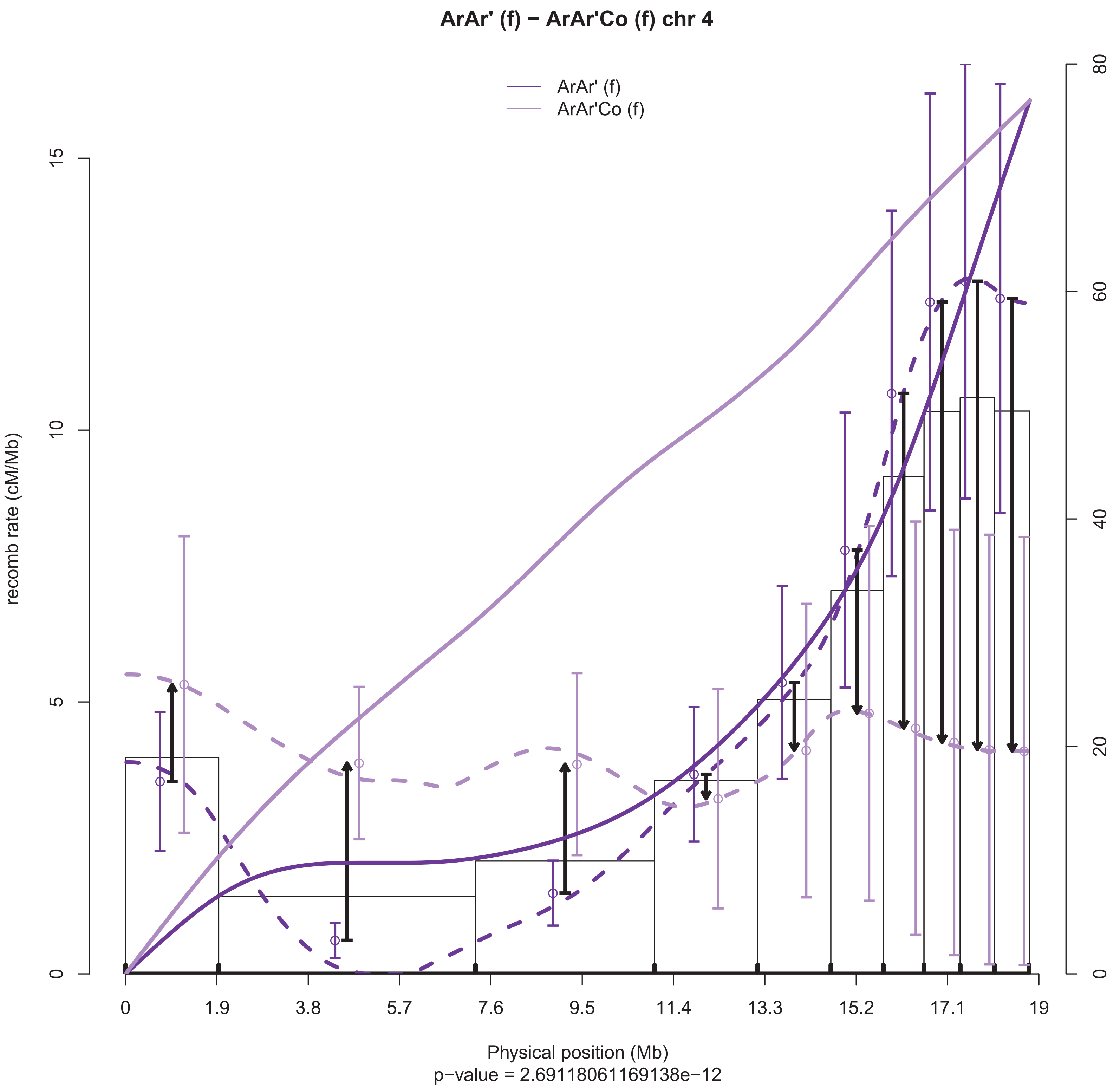

ArAr' (f) − ArAr'Co (f) chr 4
ArAr' (f)
ArAr'Co (f)
recomb rate (cM/Mb)
0
5
10
15
0
20
40
60
80
0
1.9
3.8
5.7
7.6
9.5
11.4
13.3
15.2
17.1
19
Physical position (Mb)
p−value = 2.69118061169138e−12

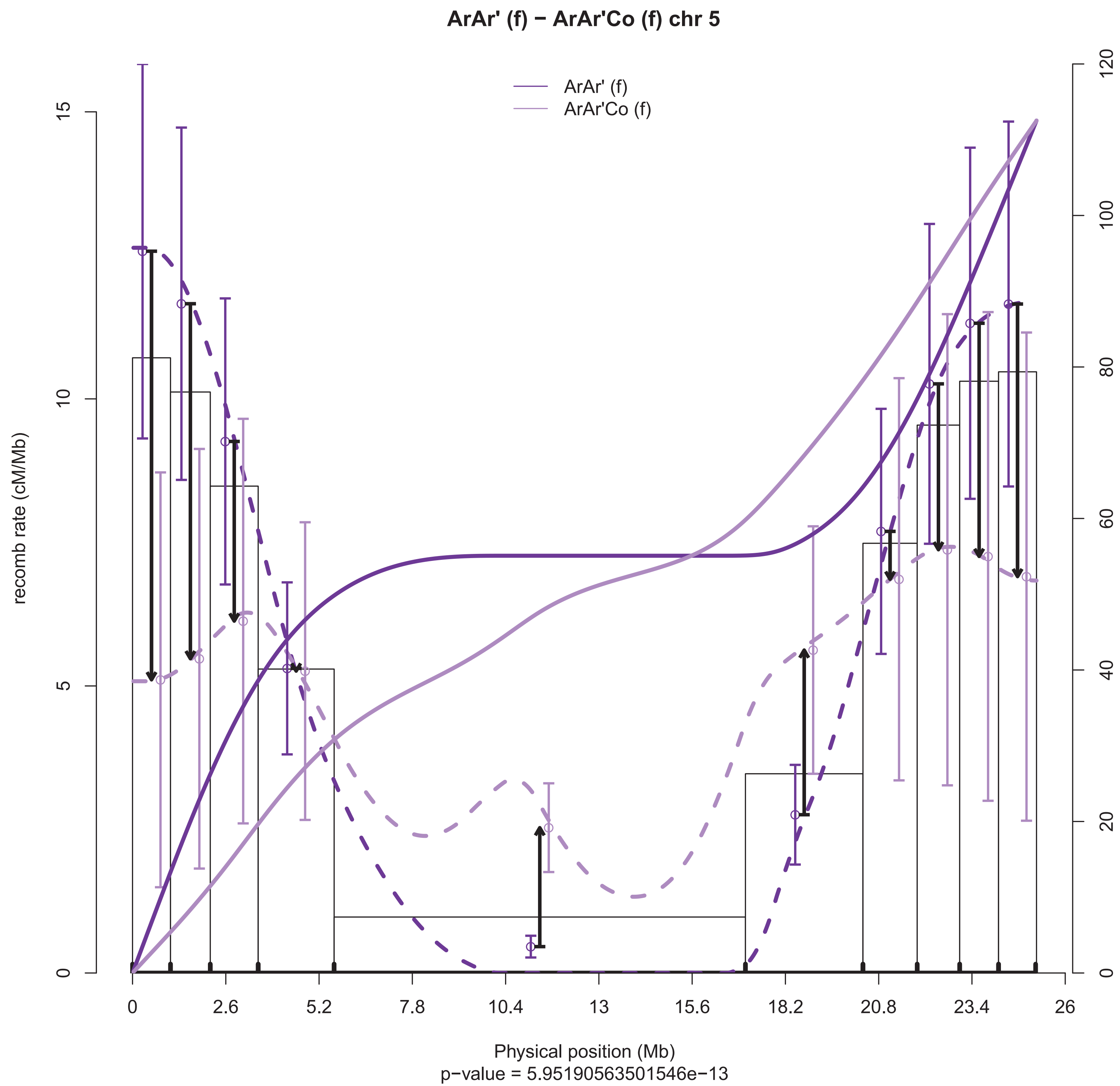

ArAr' (f) − ArAr'Co (f) chr 5
ArAr' (f)
ArAr'Co (f)
recomb rate (cM/Mb)
0
5
10
15
0
20
40
60
80
100
120
0
2.6
5.2
7.8
10.4
13
15.6
18.2
20.8
23.4
26
Physical position (Mb)
p−value = 5.95190563501546e−13

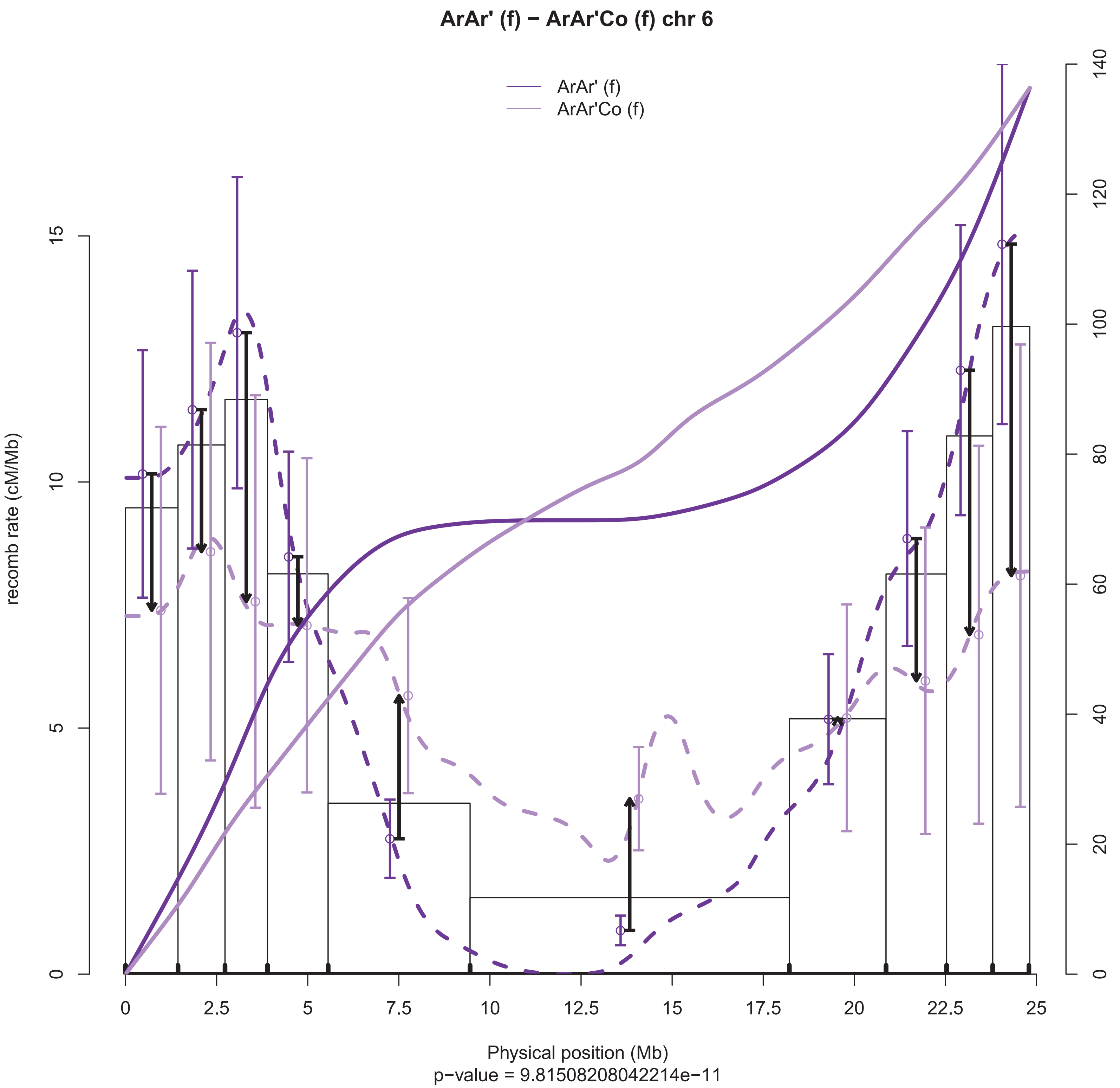
ArAr' (f) − ArAr'Co (f) chr 6
ArAr' (f)
ArAr'Co (f)
recomb rate (cM/Mb)
0
5
10
15
0
20
40
60
80
100
120
140
0
2.5
5
7.5
10
12.5
15
17.5
20
22.5
25
Physical position (Mb)
p−value = 9.81508208042214e−11

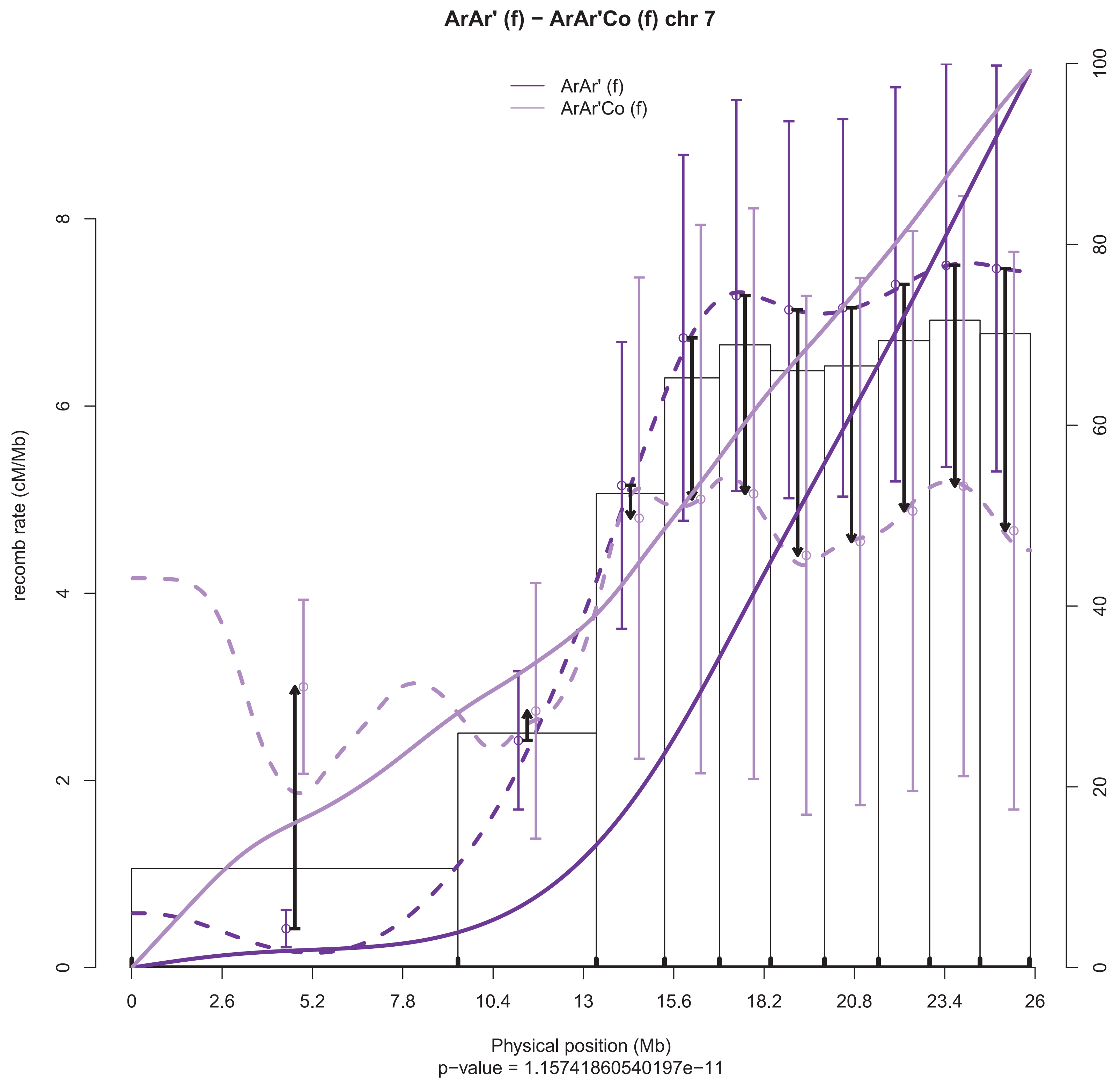

ArAr' (f) − ArAr'Co (f) chr 7
ArAr' (f)
ArAr'Co (f)
recomb rate (cM/Mb)
0
2
4
6
8
0
20
40
60
80
100
0
2.6
5.2
7.8
10.4
13
15.6
18.2
20.8
23.4
26
Physical position (Mb)
p−value = 1.15741860540197e−11

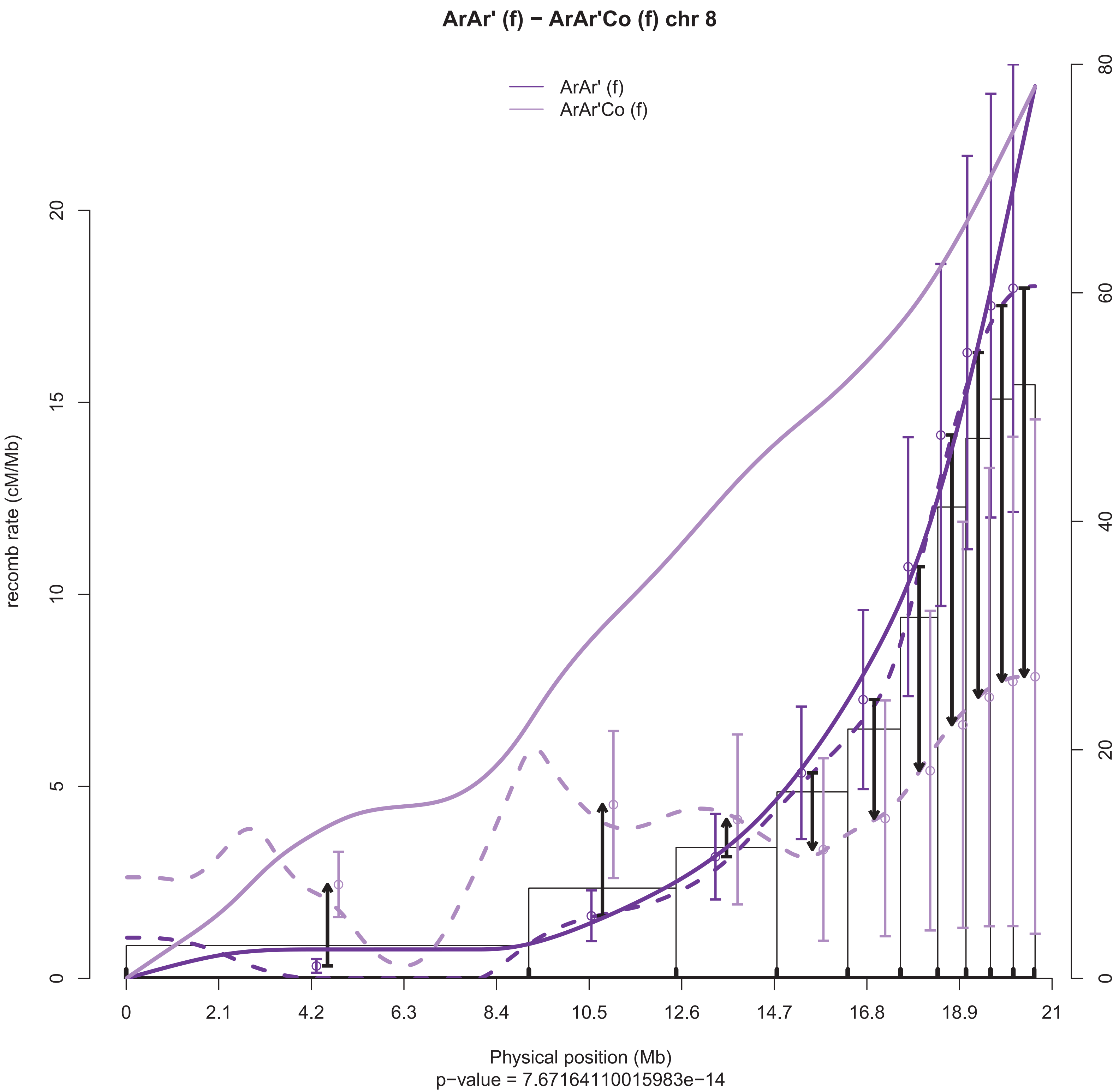
ArAr' (f) − ArAr'Co (f) chr 8
ArAr' (f)
ArAr'Co (f)
recomb rate (cM/Mb)
0
5
10
15
20
0
20
40
60
80
0
2.1
4.2
6.3
8.4
10.5
12.6
14.7
16.8
18.9
21
Physical position (Mb)
p−value = 7.67164110015983e−14

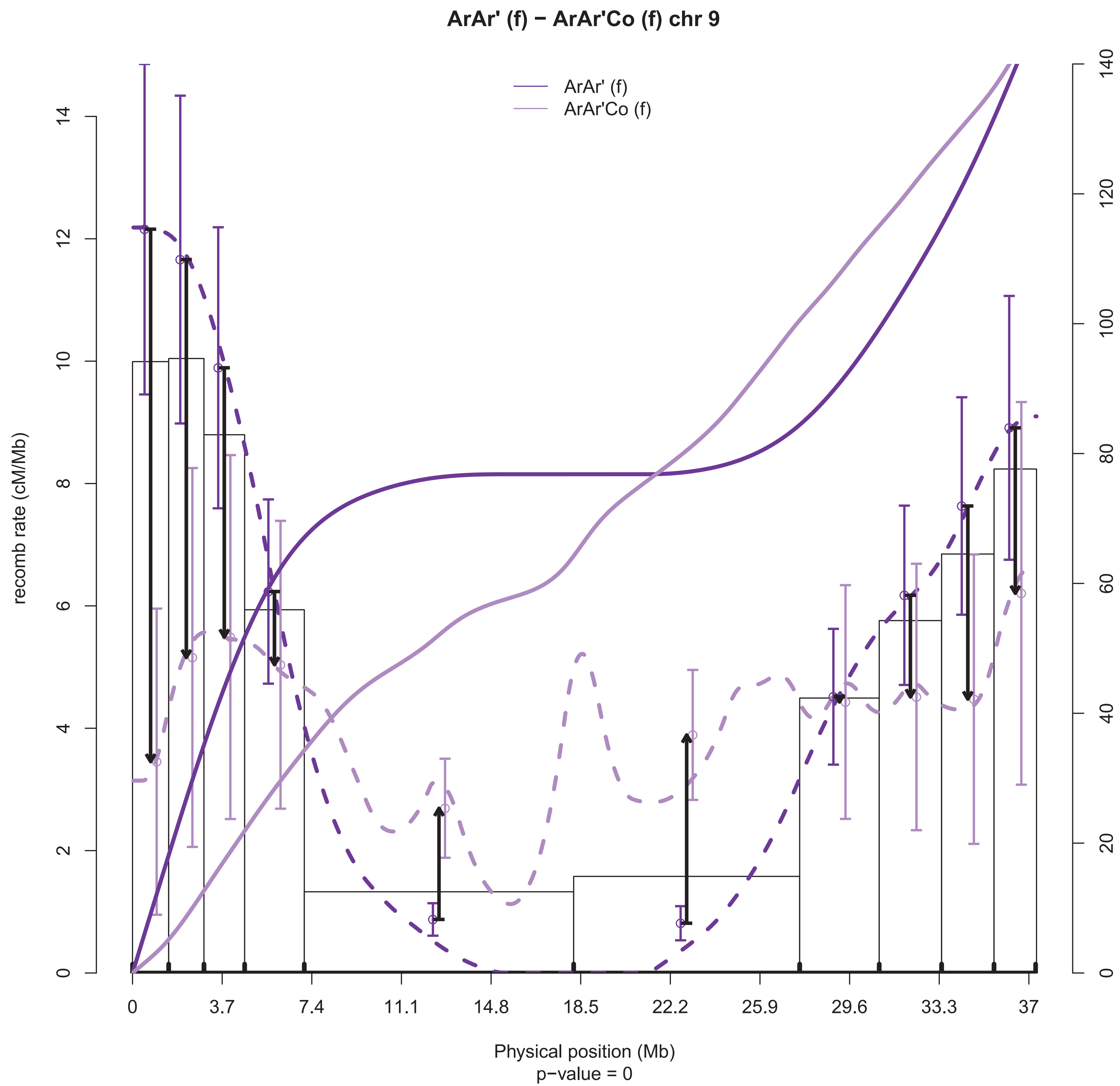
ArAr' (f) − ArAr'Co (f) chr 9
ArAr' (f)
ArAr'Co (f)
recomb rate (cM/Mb)
0
2
4
6
8
10
12
14
0
20
40
60
80
100
120
140
0
3.7
7.4
11.1
14.8
18.5
22.2
25.9
29.6
33.3
37
Physical position (Mb)
p−value = 0

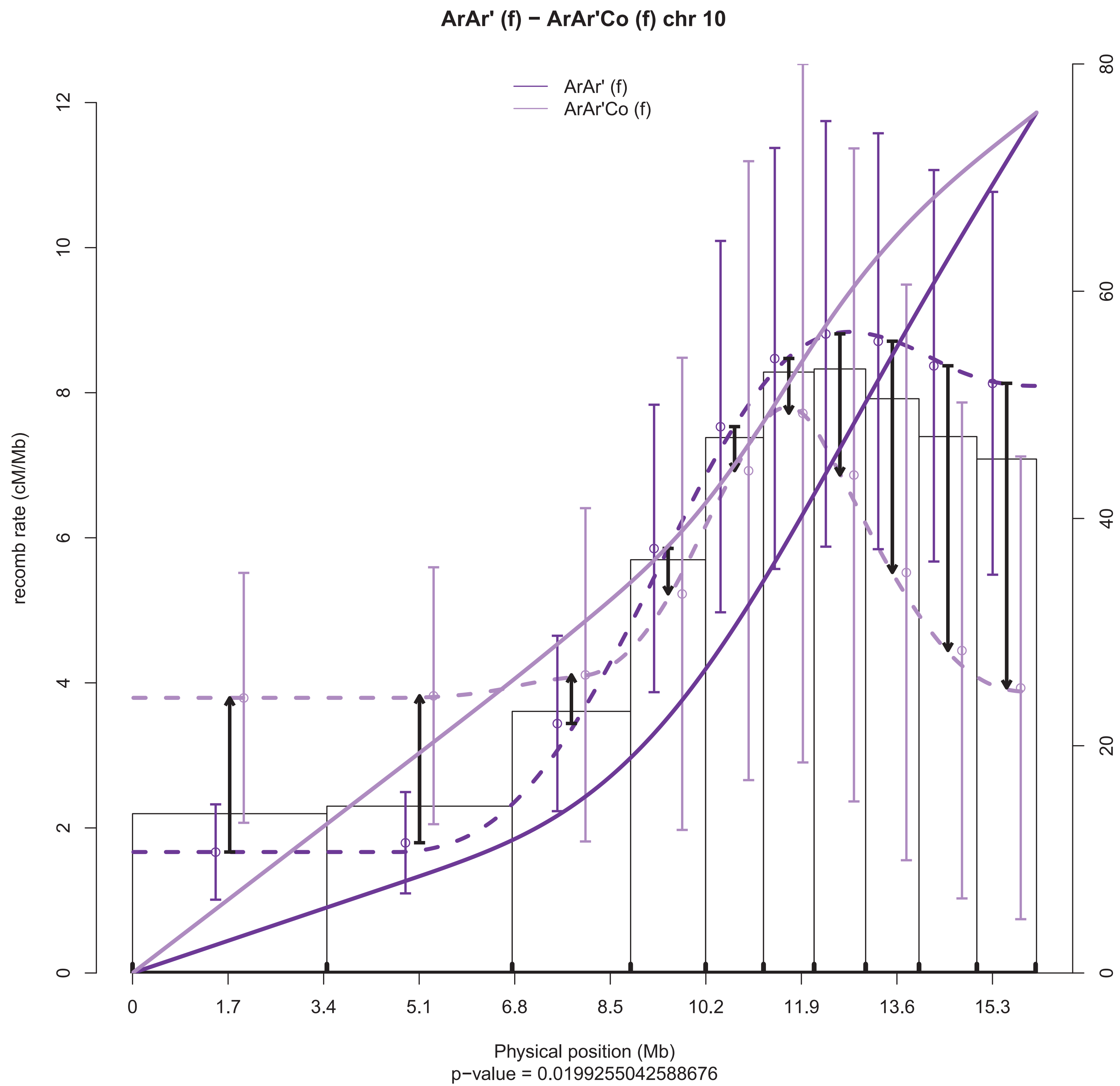

ArAr' (f) − ArAr'Co (f) chr 10
ArAr' (f)
ArAr'Co (f)
recomb rate (cM/Mb)
0
2
4
6
8
10
12
0
20
40
60
80
0
1.7
3.4
5.1
6.8
8.5
10.2
11.9
13.6
15.3
Physical position (Mb)
p−value = 0.0199255042588676

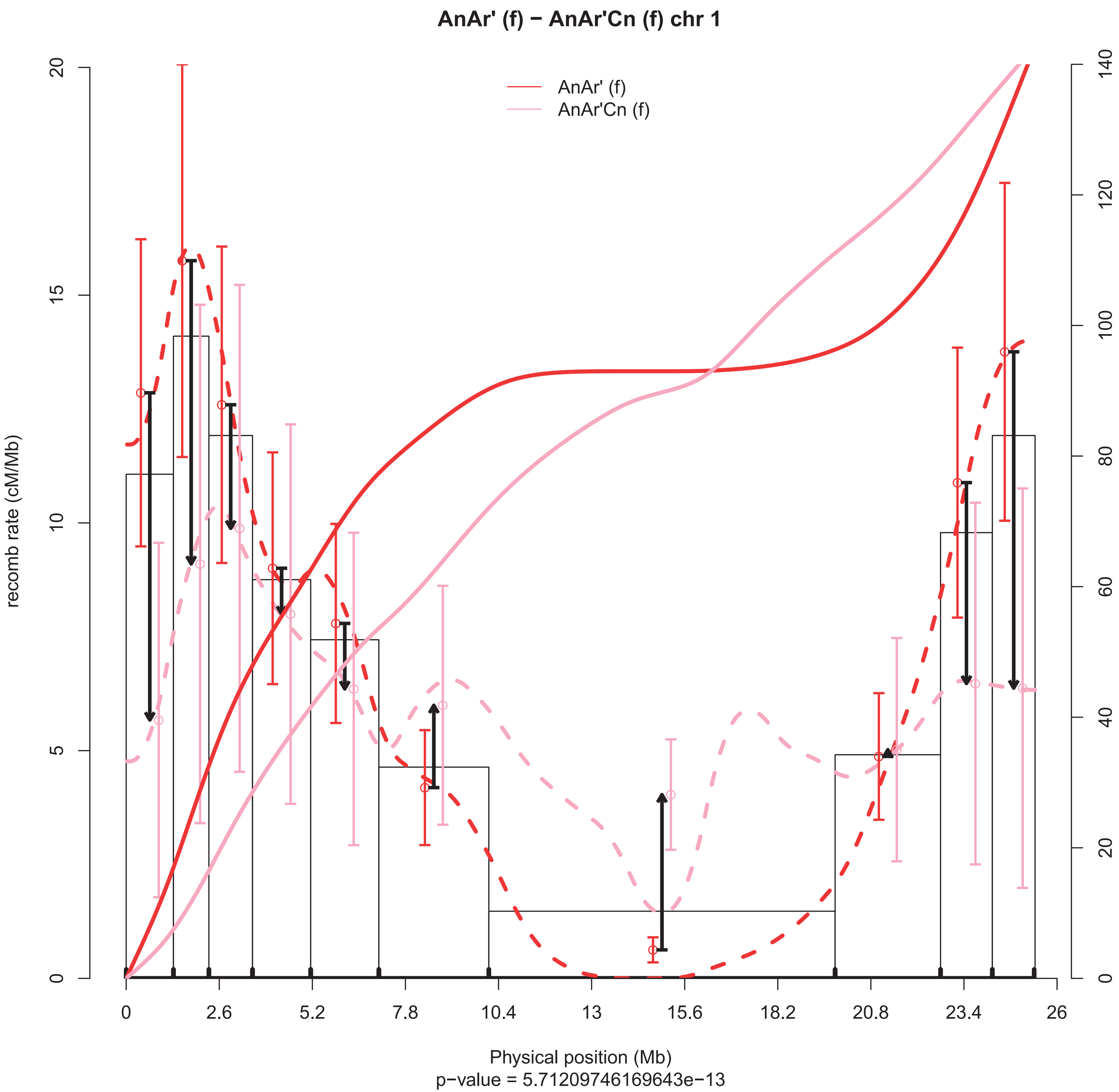
AnAr' (f) − AnAr'Cn (f) chr 1
AnAr' (f)
AnAr'Cn (f)
recomb rate (cM/Mb)
0
5
10
15
20
0
2.6
5.2
7.8
10.4
13
15.6
18.2
20.8
23.4
26
0
20
40
60
80
100
120
140
Physical position (Mb)
p−value = 5.71209746169643e−13

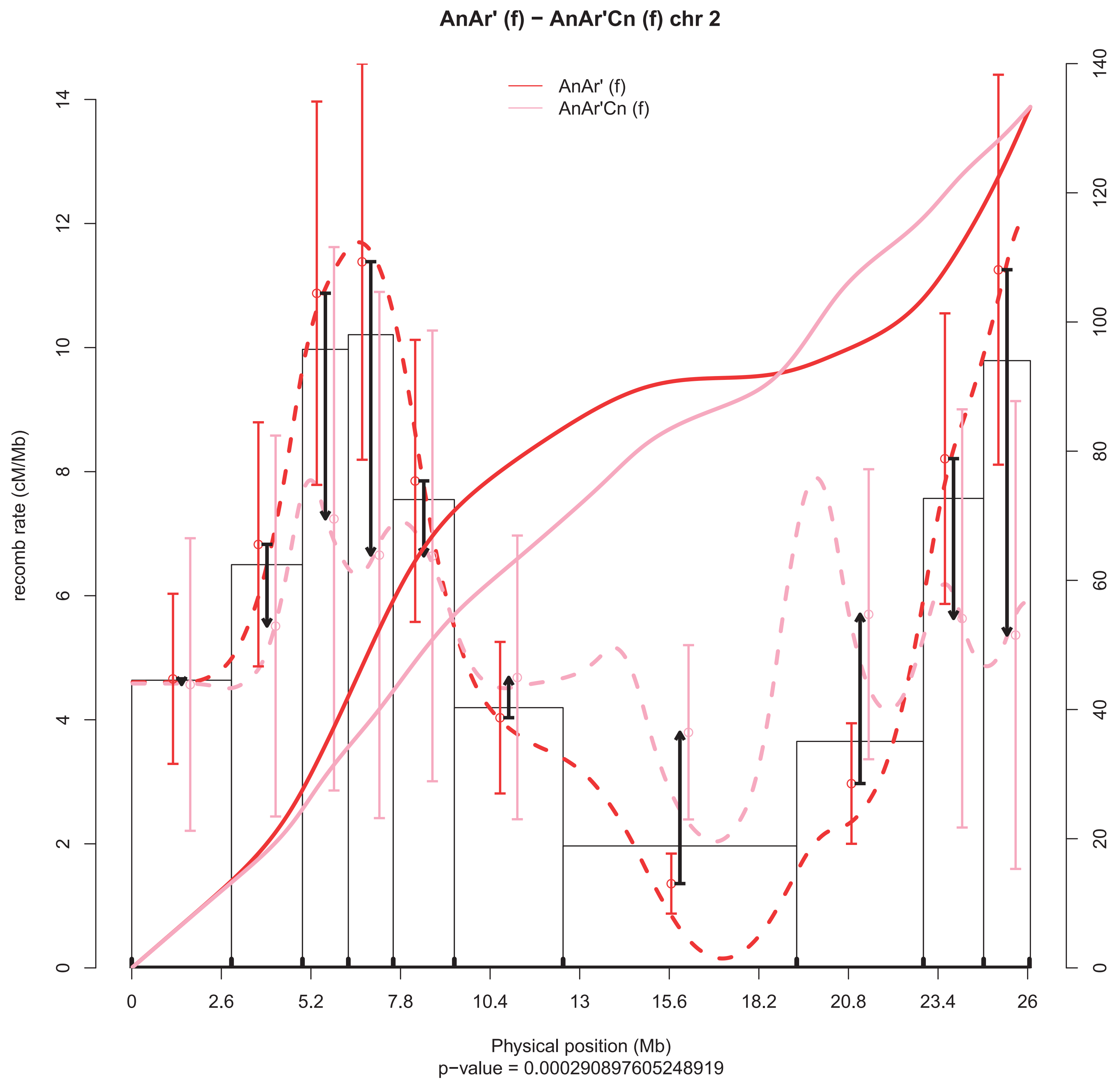

AnAr' (f) − AnAr'Cn (f) chr 2
AnAr' (f)
AnAr'Cn (f)
recomb rate (cM/Mb)
0
2
4
6
8
10
12
14
0
20
40
60
80
100
120
140
0
2.6
5.2
7.8
10.4
13
15.6
18.2
20.8
23.4
26
Physical position (Mb)
p−value = 0.000290897605248919

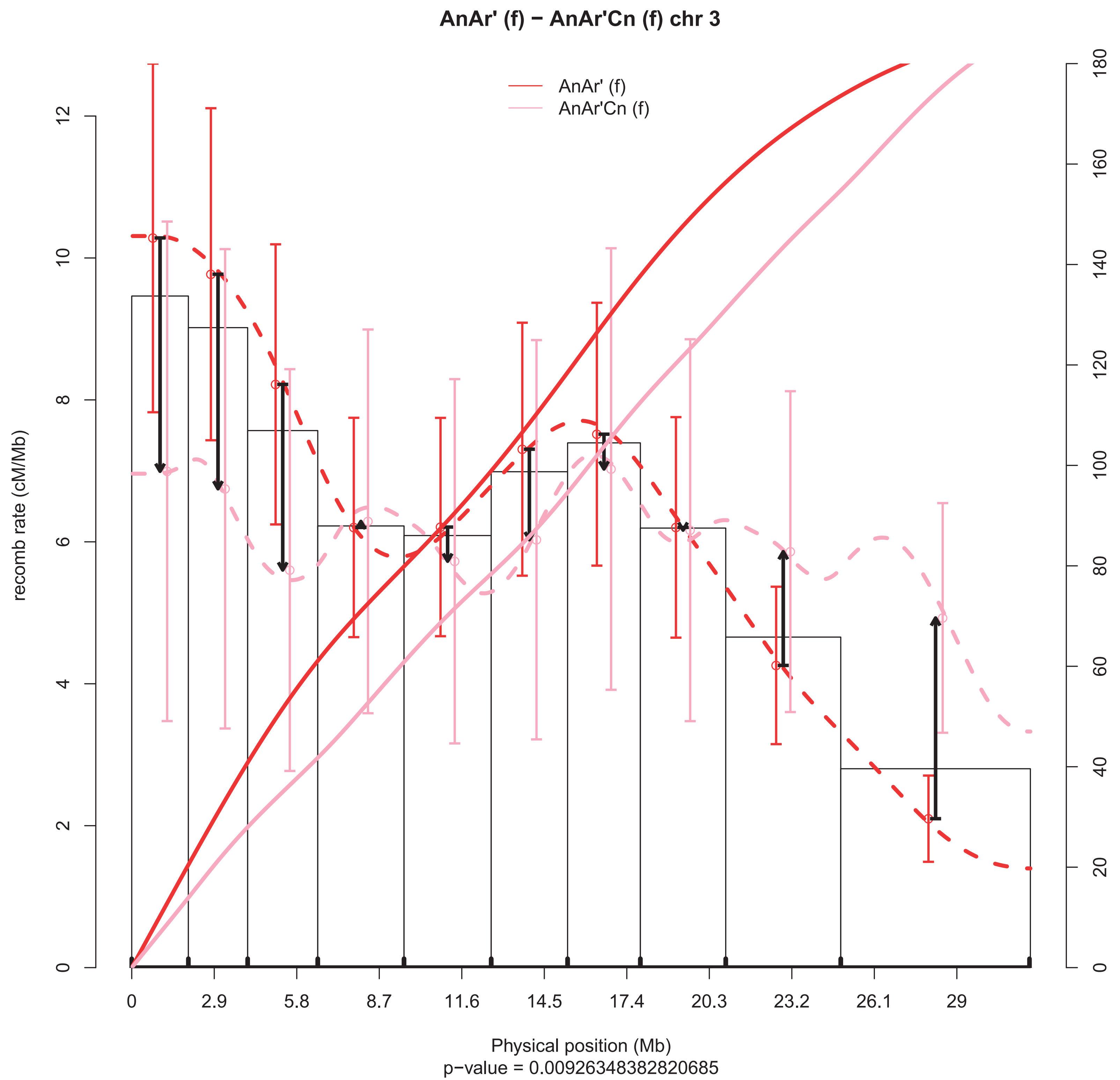

AnAr' (f) − AnAr'Cn (f) chr 3
AnAr' (f)
AnAr'Cn (f)
recomb rate (cM/Mb)
0
2
4
6
8
10
12
0
20
40
60
80
100
120
140
160
180
0
2.9
5.8
8.7
11.6
14.5
17.4
20.3
23.2
26.1
29
Physical position (Mb)
p−value = 0.00926348382820685

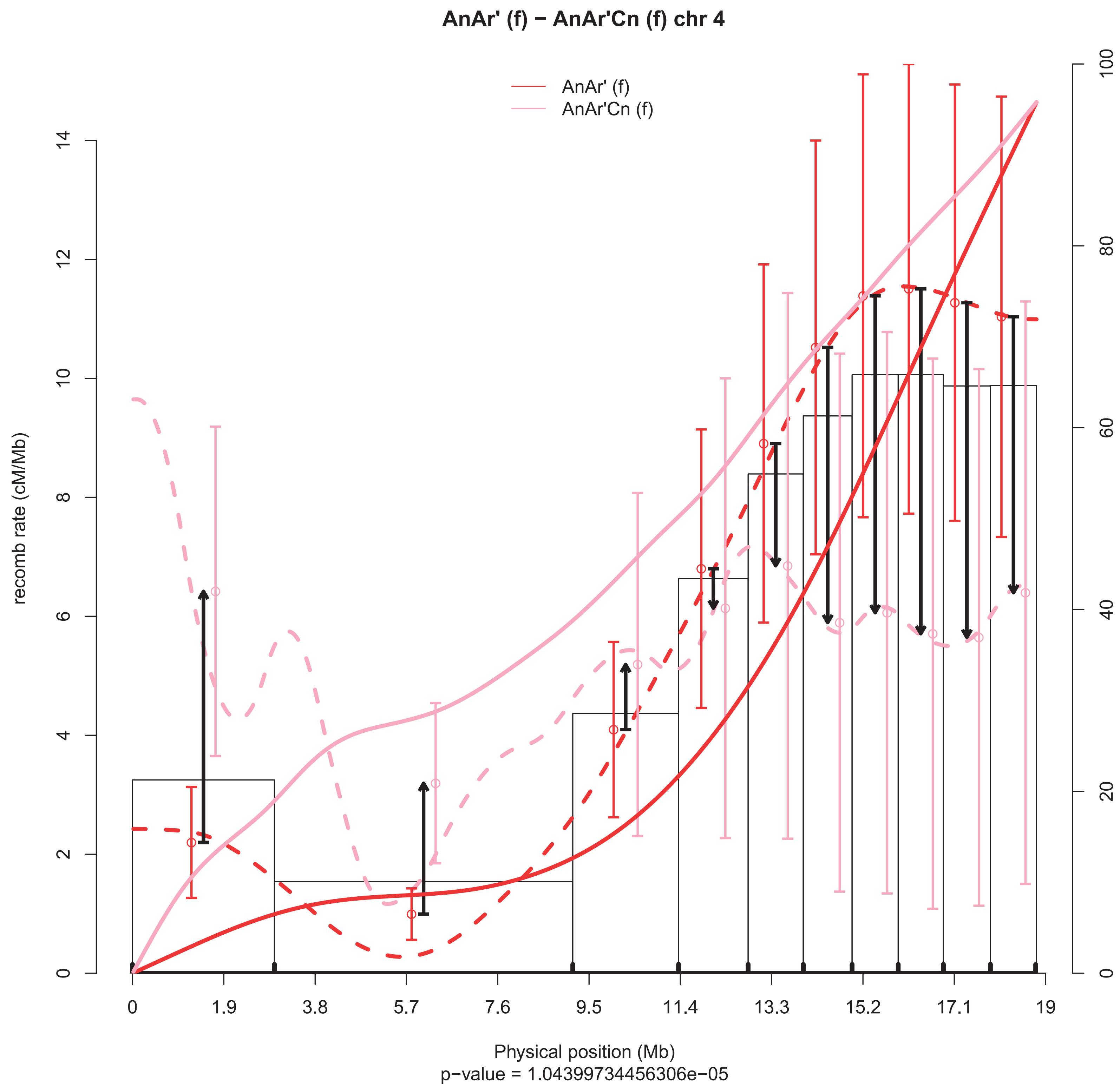

AnAr' (f) − AnAr'Cn (f) chr 4
AnAr' (f)
AnAr'Cn (f)
recomb rate (cM/Mb)
0
2
4
6
8
10
12
14
0
20
40
60
80
100
0
1.9
3.8
5.7
7.6
9.5
11.4
13.3
15.2
17.1
19
Physical position (Mb)
p−value = 1.04399734456306e−05

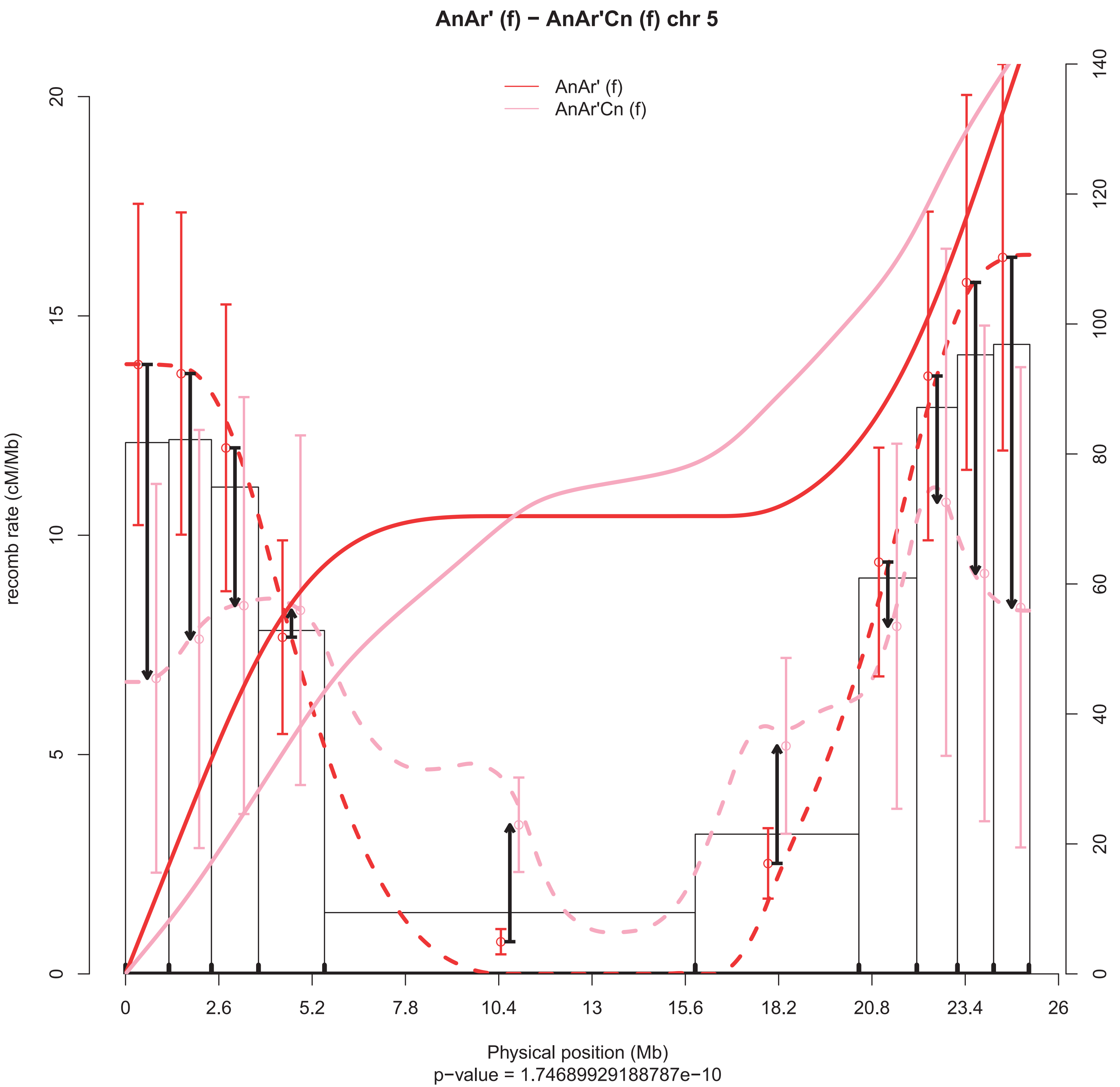

AnAr' (f) − AnAr'Cn (f) chr 5
AnAr' (f)
AnAr'Cn (f)
recomb rate (cM/Mb)
0
5
10
15
20
0
20
40
60
80
100
120
140
0
2.6
5.2
7.8
10.4
13
15.6
18.2
20.8
23.4
26
Physical position (Mb)
p−value = 1.74689929188787e−10

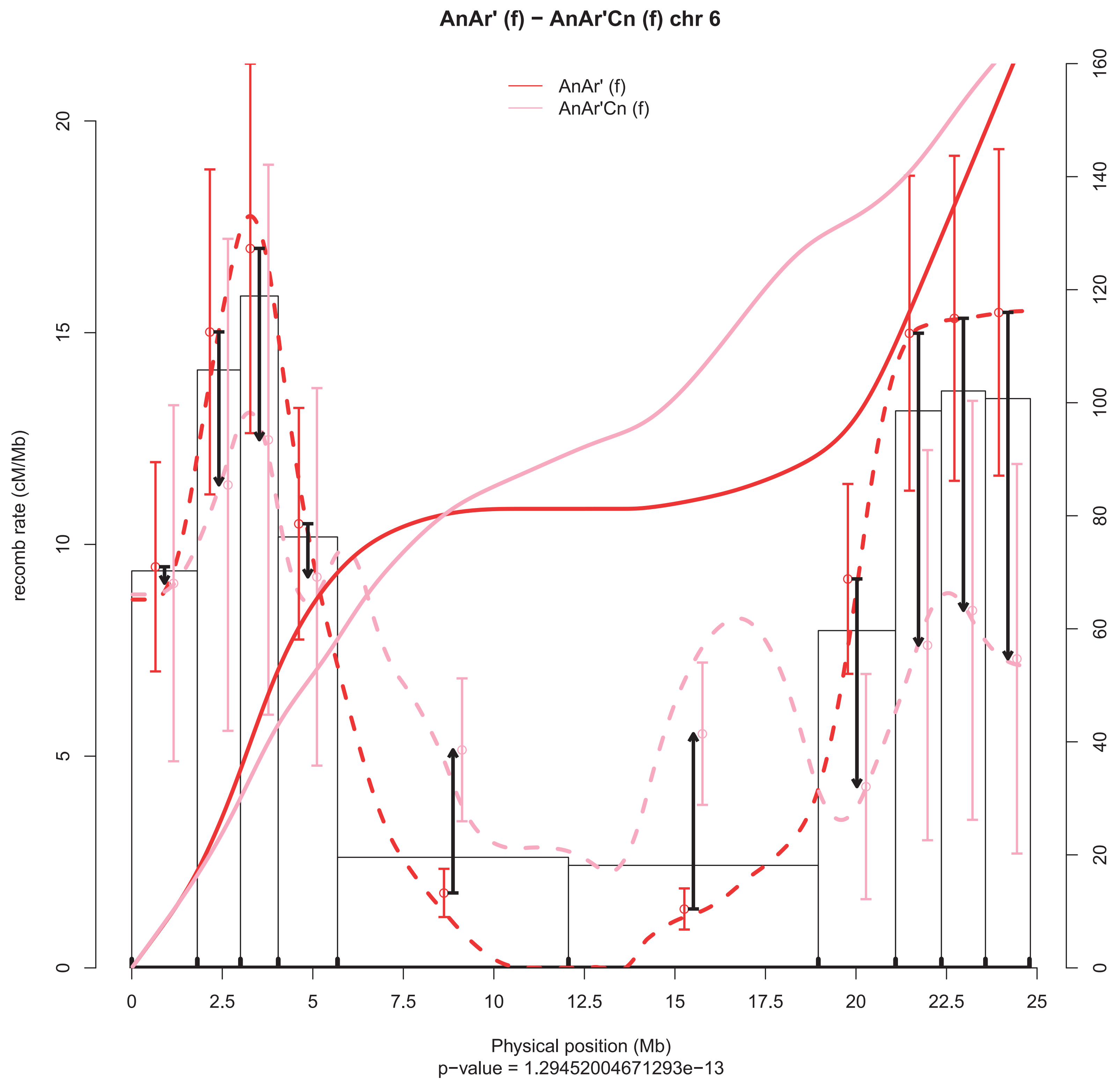
AnAr' (f) − AnAr'Cn (f) chr 6
AnAr' (f)
AnAr'Cn (f)
recomb rate (cM/Mb)
0
5
10
15
20
0
20
40
60
80
100
120
140
160
0
2.5
5
7.5
10
12.5
15
17.5
20
22.5
25
Physical position (Mb)
p−value = 1.29452004671293e−13

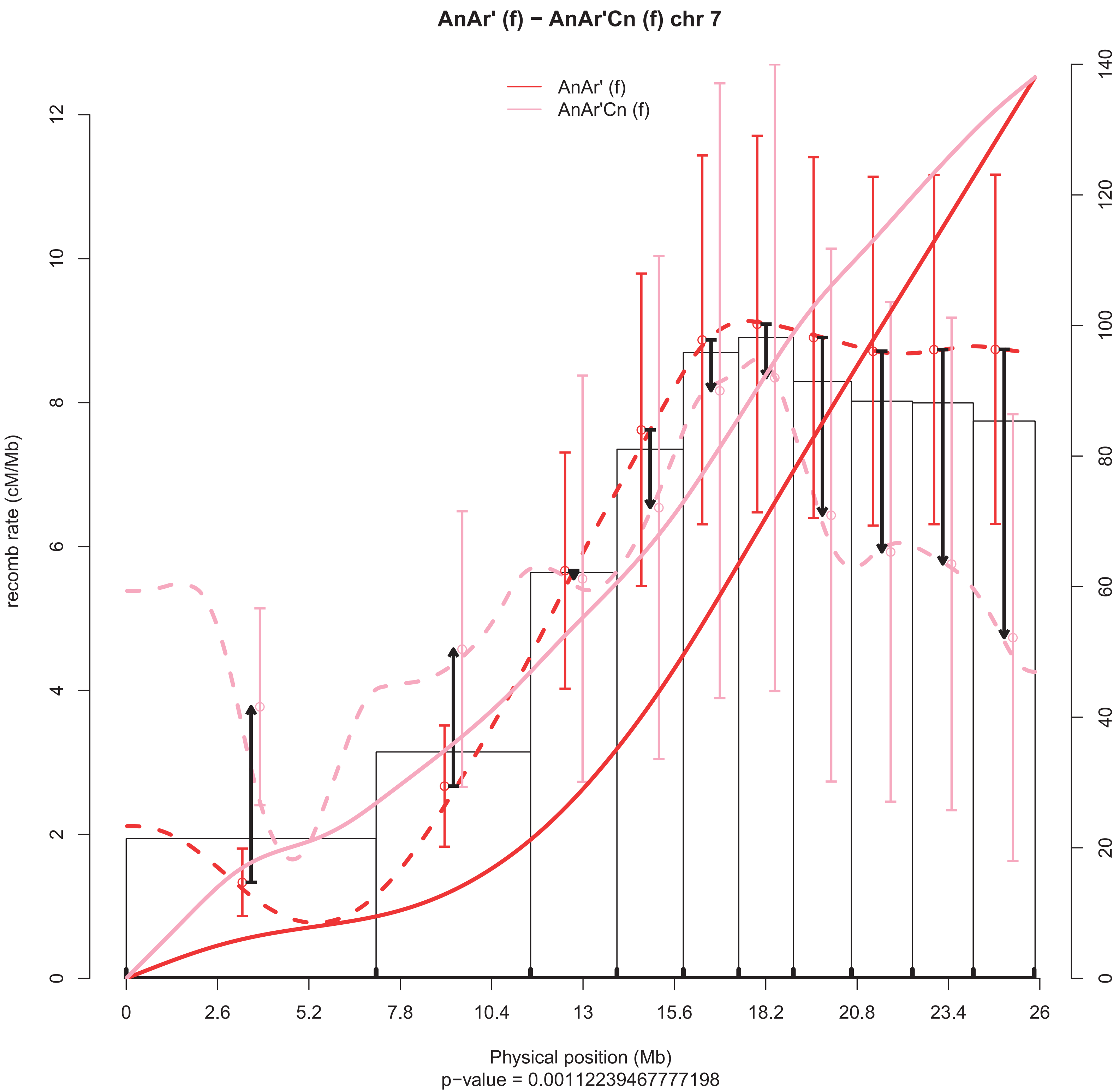

AnAr' (f) − AnAr'Cn (f) chr 7
AnAr' (f)
AnAr'Cn (f)
recomb rate (cM/Mb)
0
2
4
6
8
10
12
0
20
40
60
80
100
120
140
0
2.6
5.2
7.8
10.4
13
15.6
18.2
20.8
23.4
26
Physical position (Mb)
p−value = 0.00112239467777198

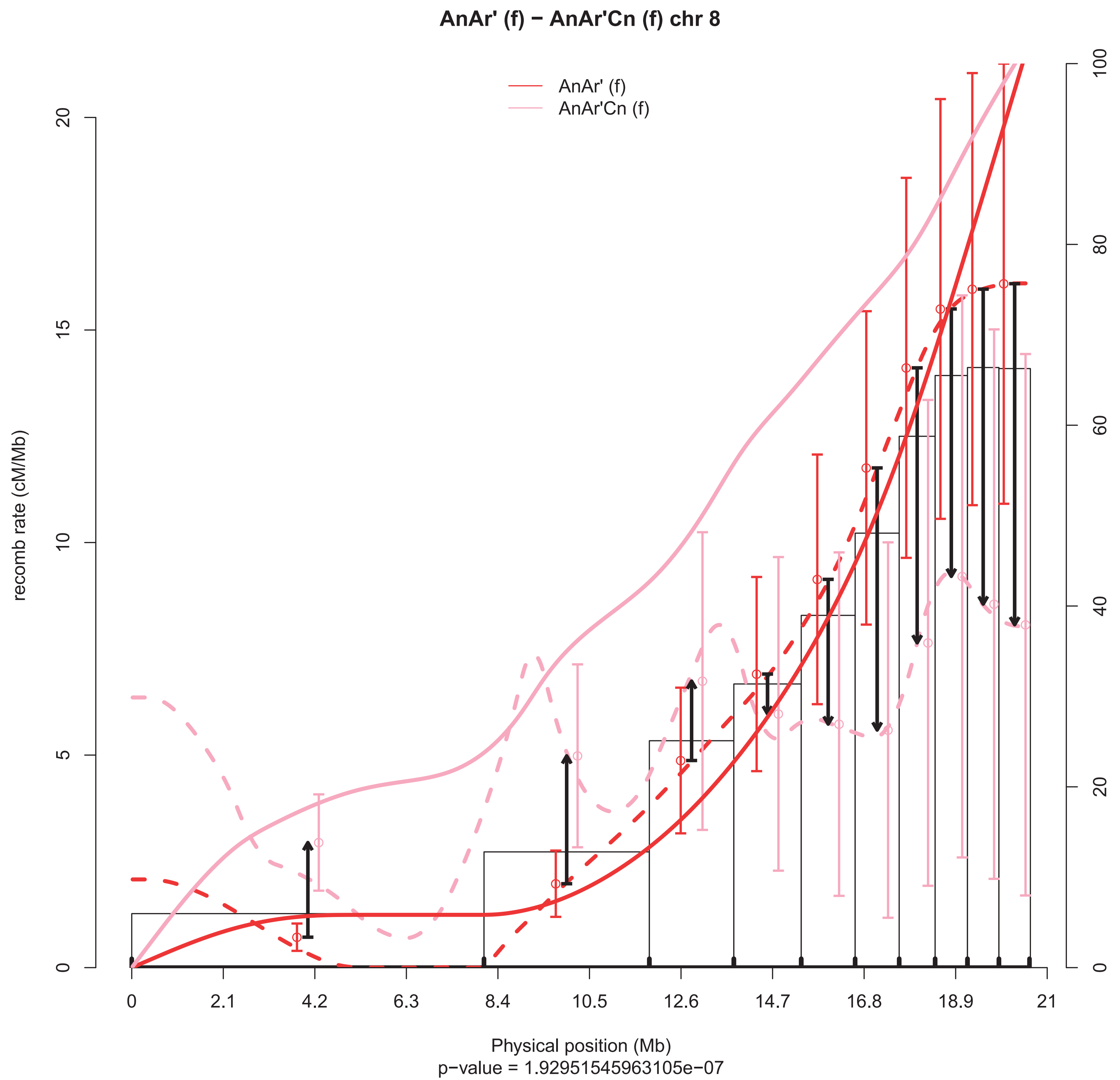

AnAr' (f) − AnAr'Cn (f) chr 8
AnAr' (f)
AnAr'Cn (f)
recomb rate (cM/Mb)
0
5
10
15
20
0
20
40
60
80
100
0
2.1
4.2
6.3
8.4
10.5
12.6
14.7
16.8
18.9
21
Physical position (Mb)
p−value = 1.92951545963105e−07

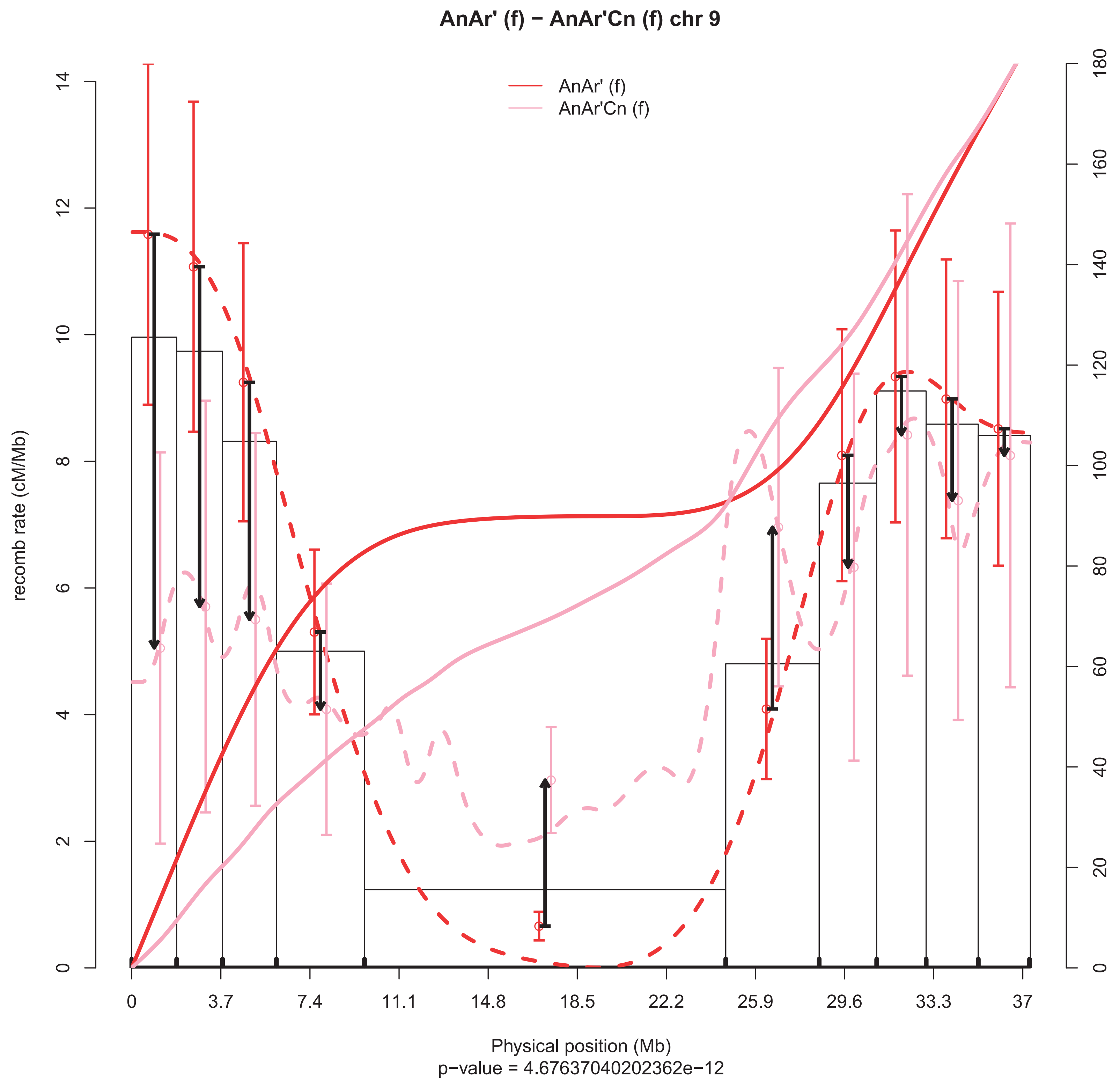
AnAr' (f) − AnAr'Cn (f) chr 9
AnAr' (f)
AnAr'Cn (f)
recomb rate (cM/Mb)
0
2
4
6
8
10
12
14
0
20
40
60
80
100
120
140
160
180
0
3.7
7.4
11.1
14.8
18.5
22.2
25.9
29.6
33.3
37
Physical position (Mb)
p−value = 4.67637040202362e−12

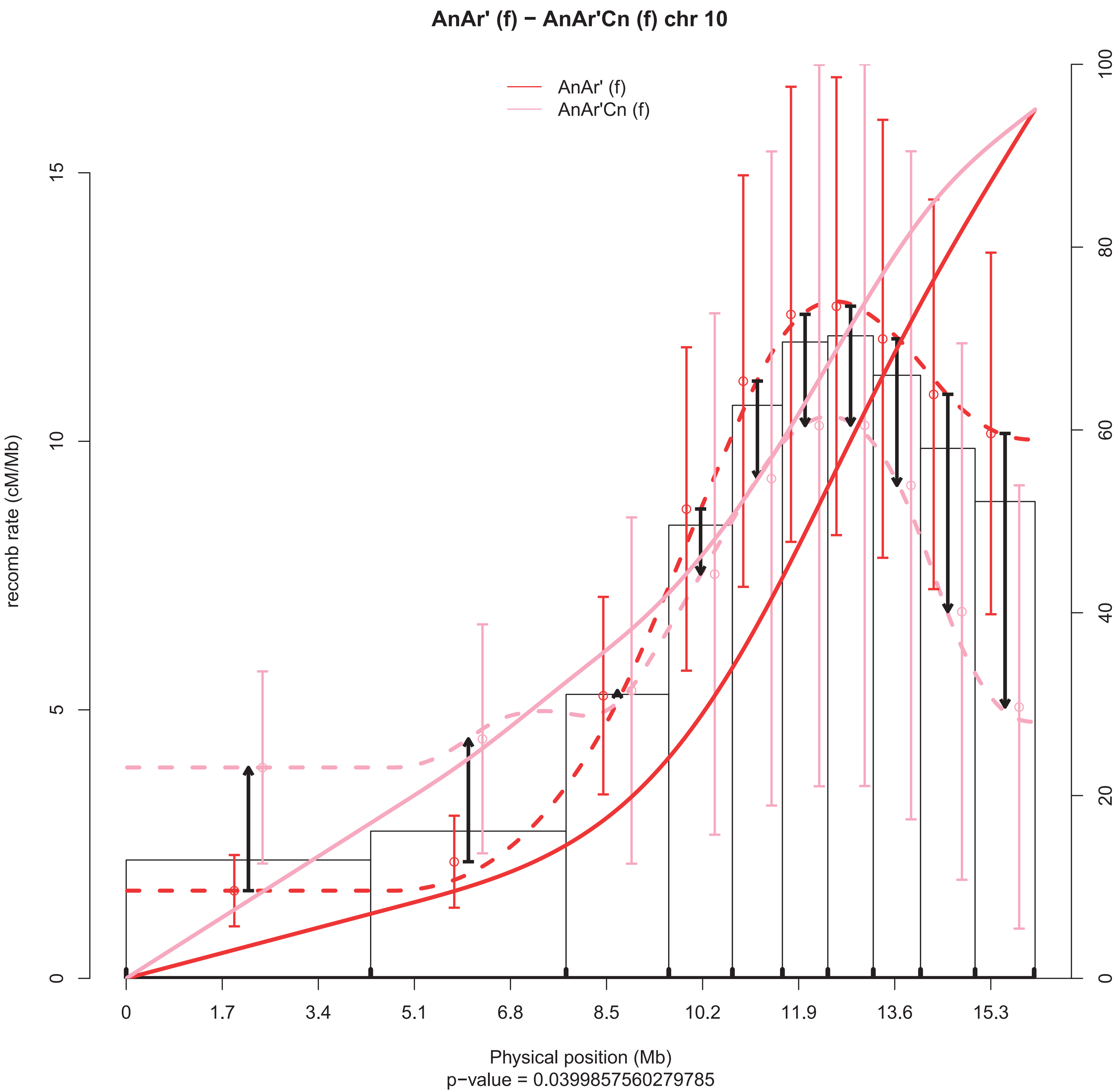
AnAr' (f) − AnAr'Cn (f) chr 10
AnAr' (f)
AnAr'Cn (f)
recomb rate (cM/Mb)
0
5
10
15
0
20
40
60
80
100
0
1.7
3.4
5.1
6.8
8.5
10.2
11.9
13.6
15.3
Physical position (Mb)
p−value = 0.0399857560279785

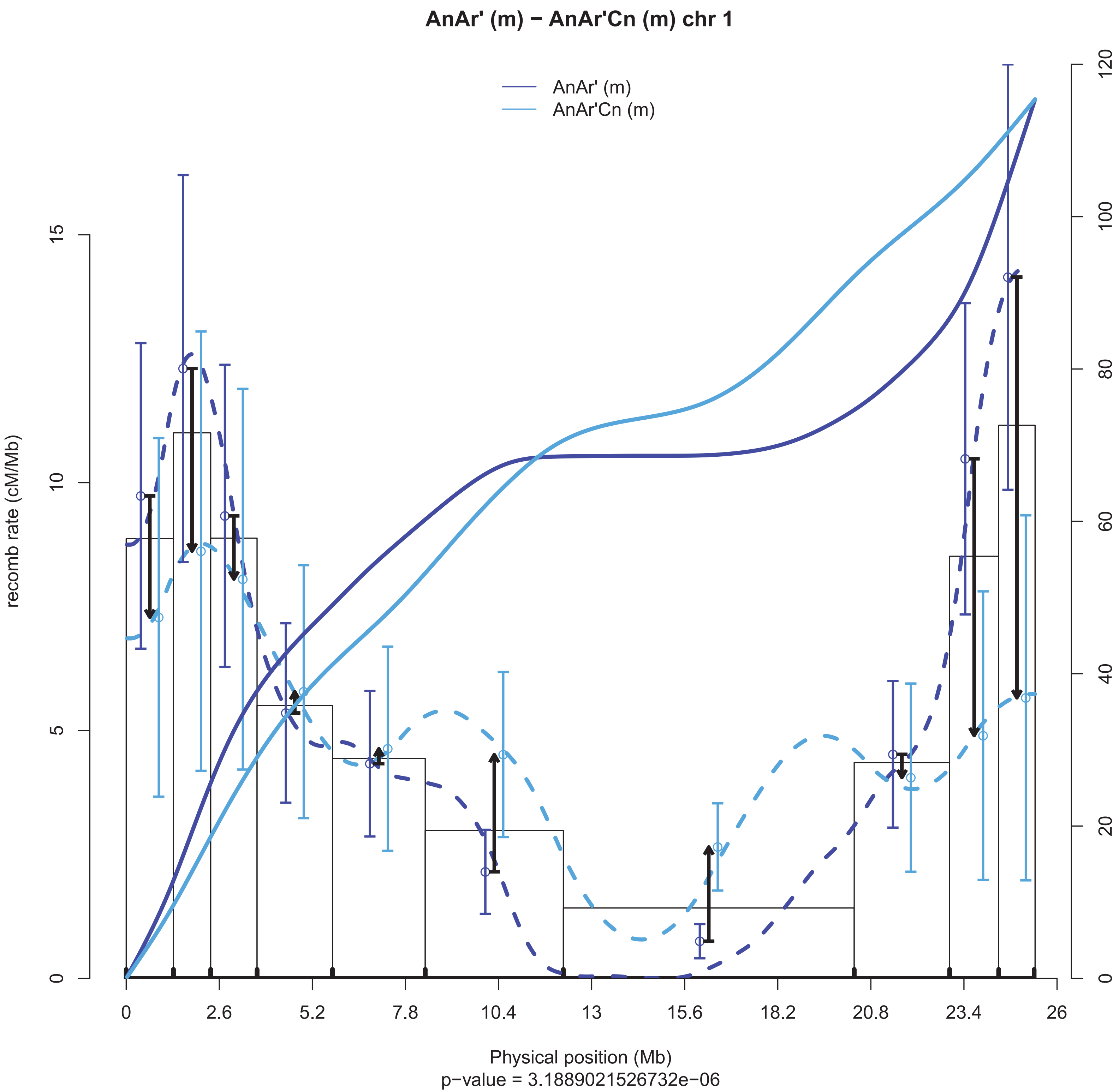
AnAr' (m) − AnAr'Cn (m) chr 1
AnAr' (m)
AnAr'Cn (m)
recomb rate (cM/Mb)
0
5
10
15
0
20
40
60
80
100
120
0
2.6
5.2
7.8
10.4
13
15.6
18.2
20.8
23.4
26
Physical position (Mb)
p−value = 3.1889021526732e−06

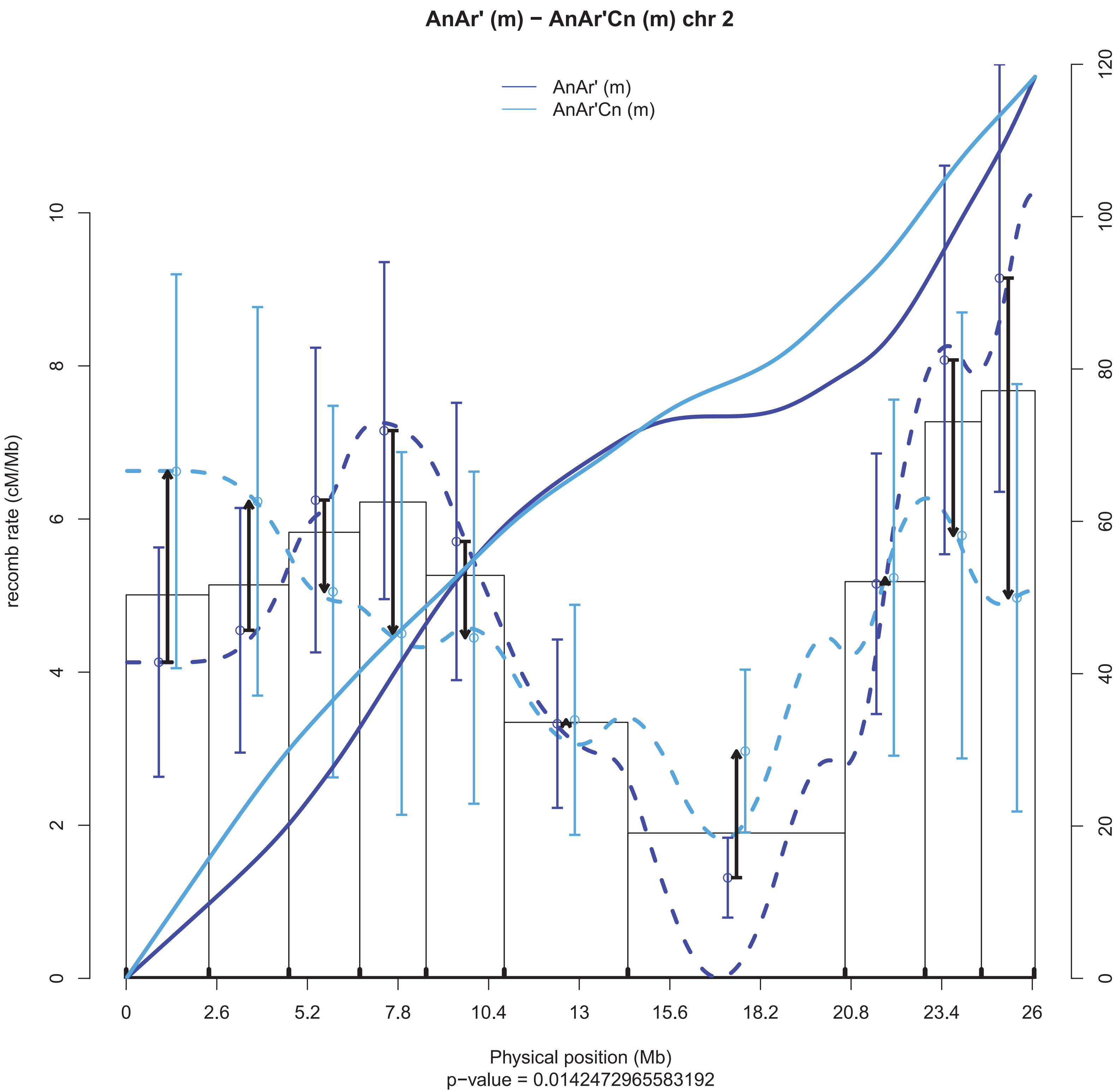

AnAr' (m) − AnAr'Cn (m) chr 2
AnAr' (m)
AnAr'Cn (m)
recomb rate (cM/Mb)
0
2
4
6
8
10
0
20
40
60
80
100
120
0
2.6
5.2
7.8
10.4
13
15.6
18.2
20.8
23.4
26
Physical position (Mb)
p−value = 0.0142472965583192

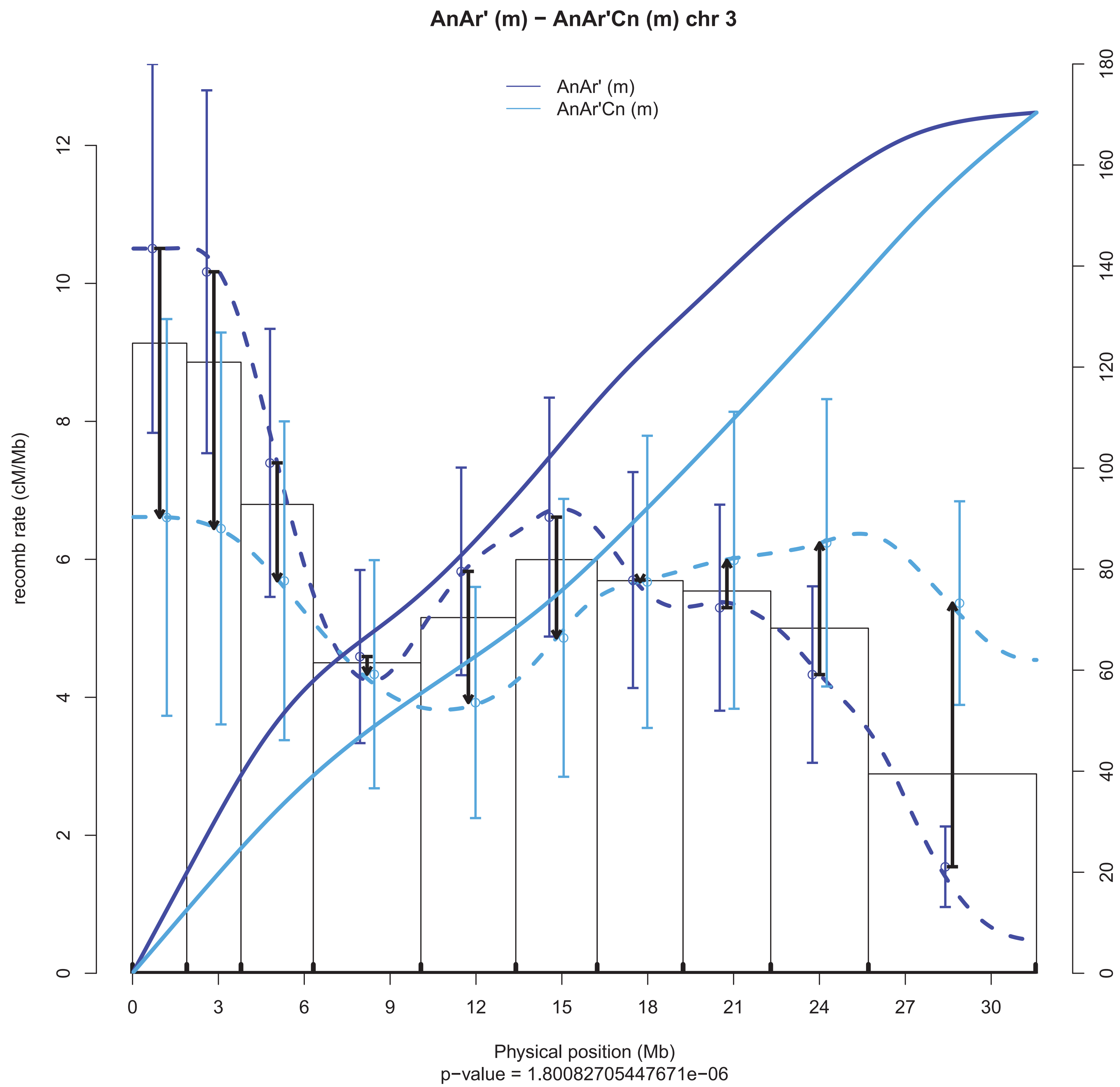

AnAr' (m) − AnAr'Cn (m) chr 3
AnAr' (m)
AnAr'Cn (m)
recomb rate (cM/Mb)
0
2
4
6
8
10
12
0
20
40
60
80
100
120
140
160
180
0
3
6
9
12
15
18
21
24
27
30
Physical position (Mb)
p−value = 1.80082705447671e−06

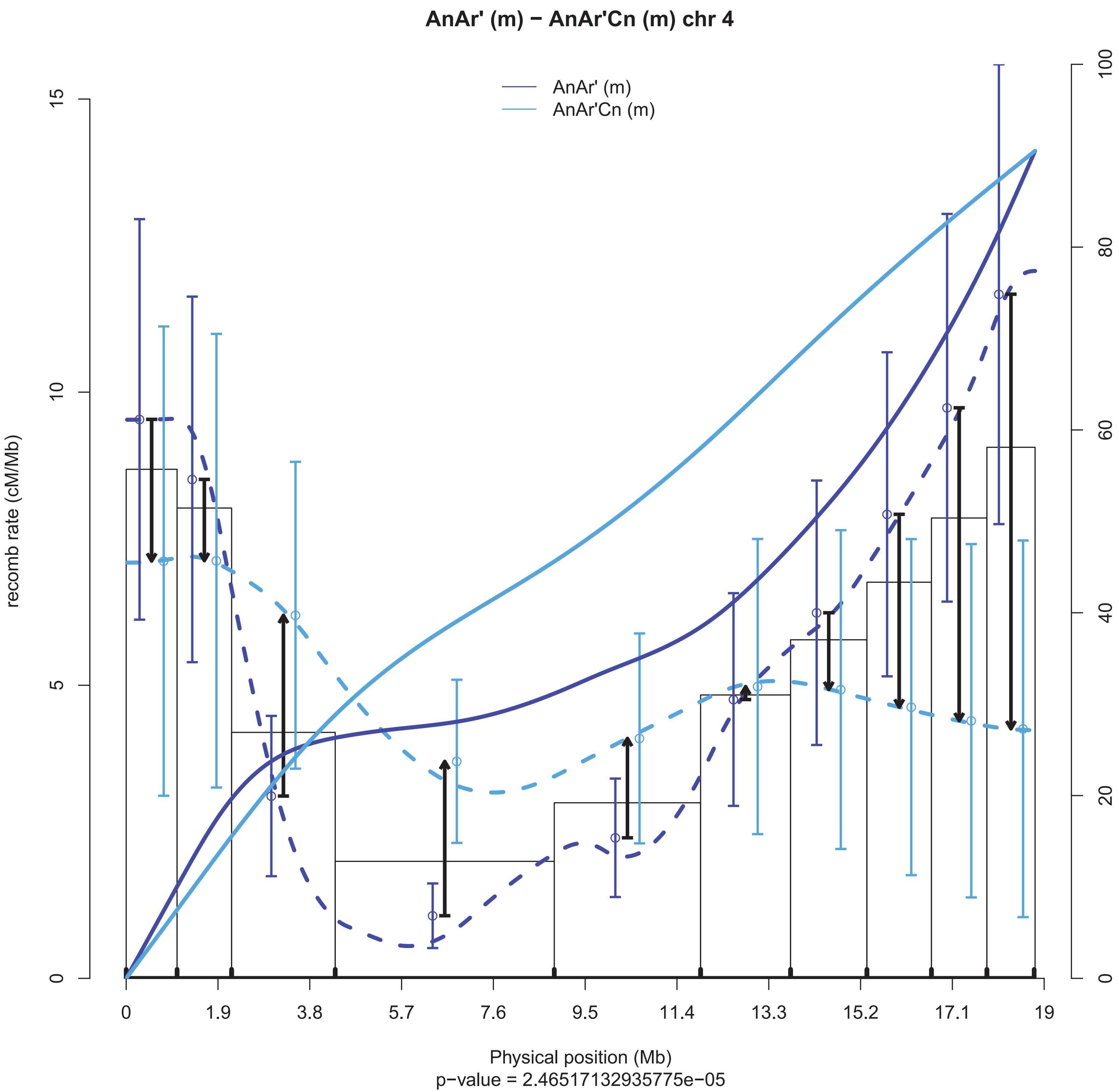

AnAr' (m) − AnAr'Cn (m) chr 4
AnAr' (m)
AnAr'Cn (m)
recomb rate (cM/Mb)
0
5
10
15
0
20
40
60
80
100
0
1.9
3.8
5.7
7.6
9.5
11.4
13.3
15.2
17.1
19
Physical position (Mb)
p−value = 2.46517132935775e−05

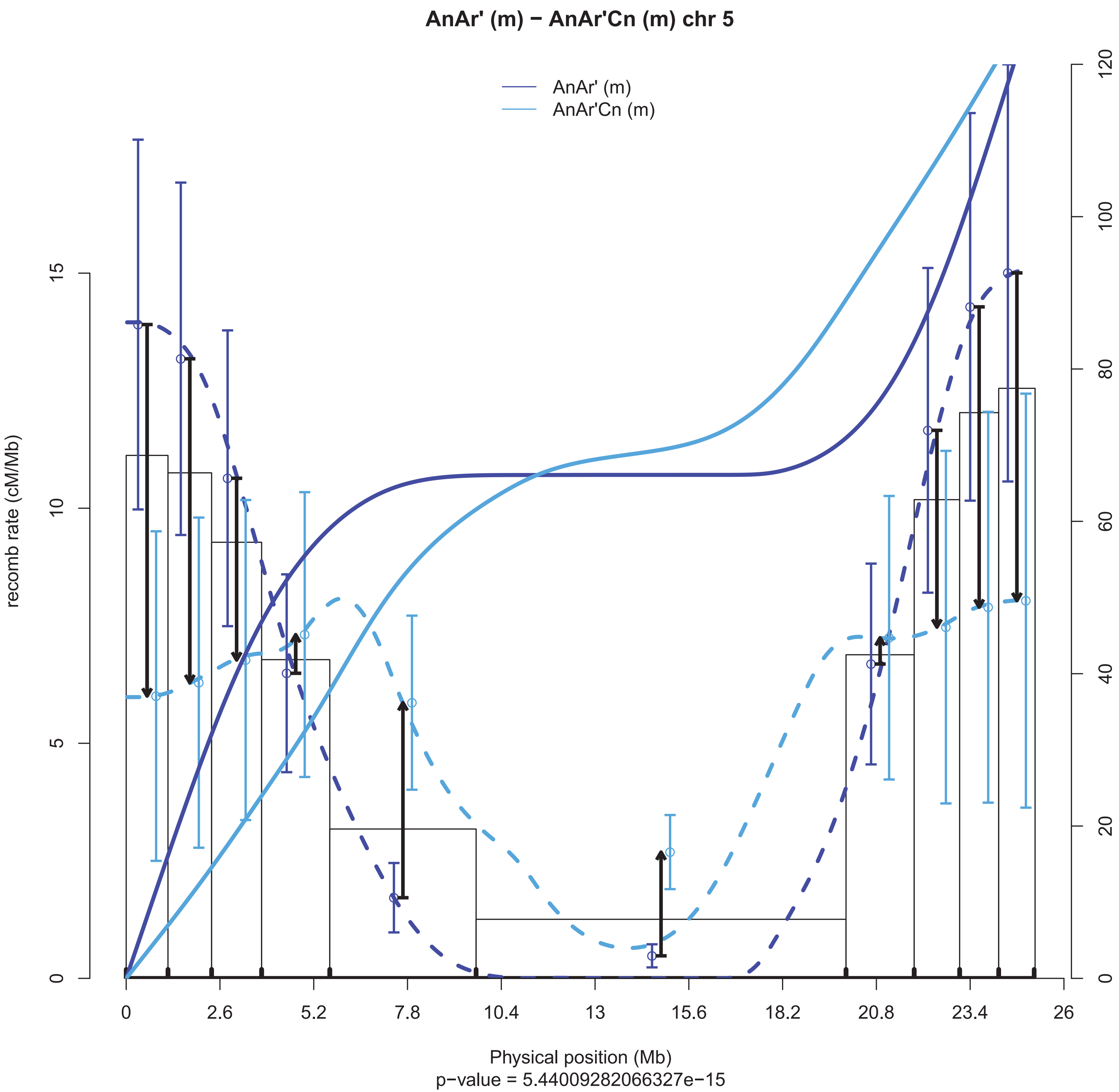

AnAr' (m) − AnAr'Cn (m) chr 5
AnAr' (m)
AnAr'Cn (m)
recomb rate (cM/Mb)
0
5
10
15
0
20
40
60
80
100
120
0
2.6
5.2
7.8
10.4
13
15.6
18.2
20.8
23.4
26
Physical position (Mb)
p−value = 5.44009282066327e−15

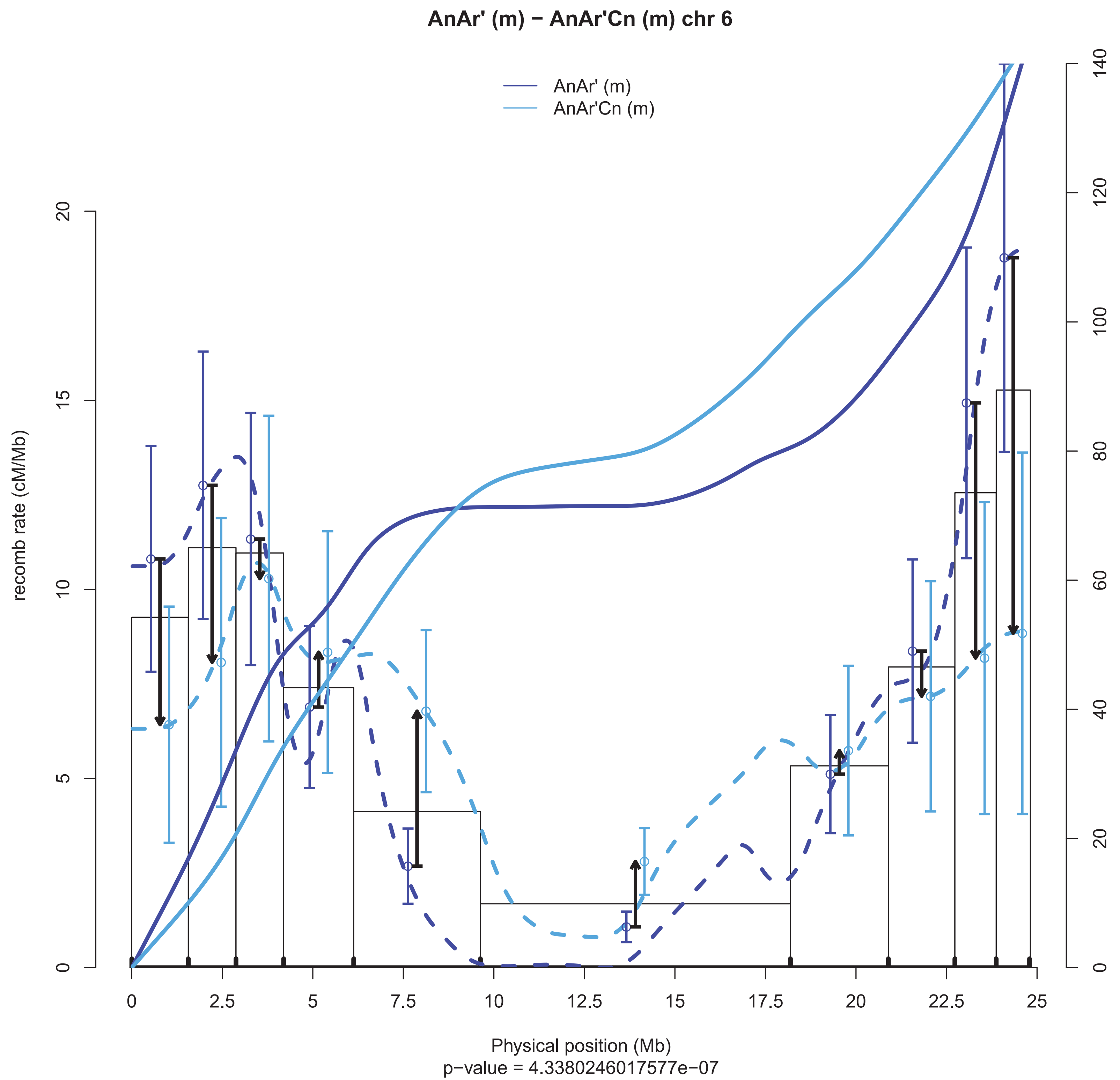
AnAr' (m) − AnAr'Cn (m) chr 6
AnAr' (m)
AnAr'Cn (m)
recomb rate (cM/Mb)
0
5
10
15
20
0
20
40
60
80
100
120
140
0
2.5
5
7.5
10
12.5
15
17.5
20
22.5
25
Physical position (Mb)
p−value = 4.3380246017577e−07

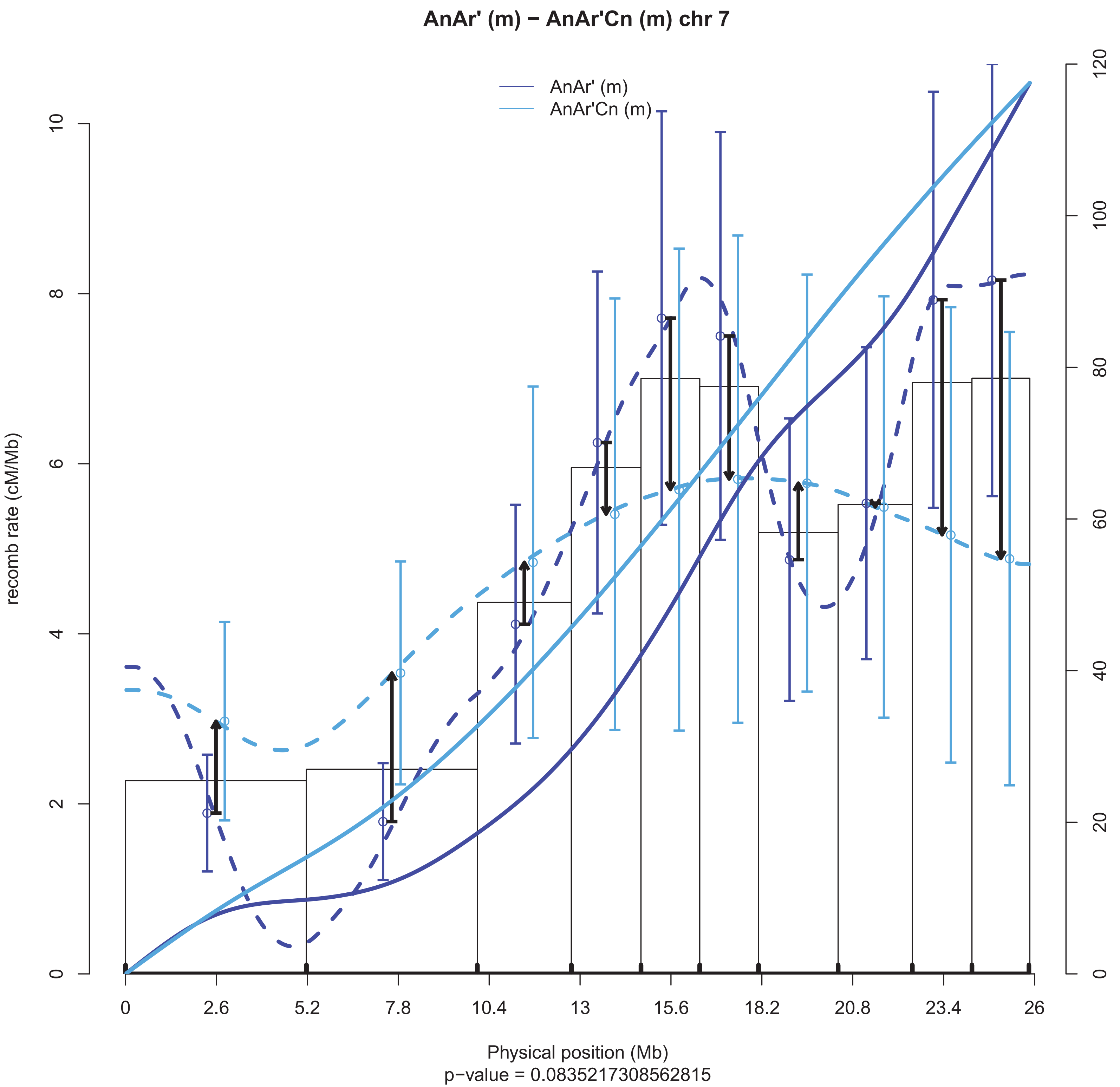

AnAr' (m) − AnAr'Cn (m) chr 7
AnAr' (m)
AnAr'Cn (m)
recomb rate (cM/Mb)
0
2
4
6
8
10
0
20
40
60
80
100
120
0
2.6
5.2
7.8
10.4
13
15.6
18.2
20.8
23.4
26
Physical position (Mb)
p−value = 0.0835217308562815

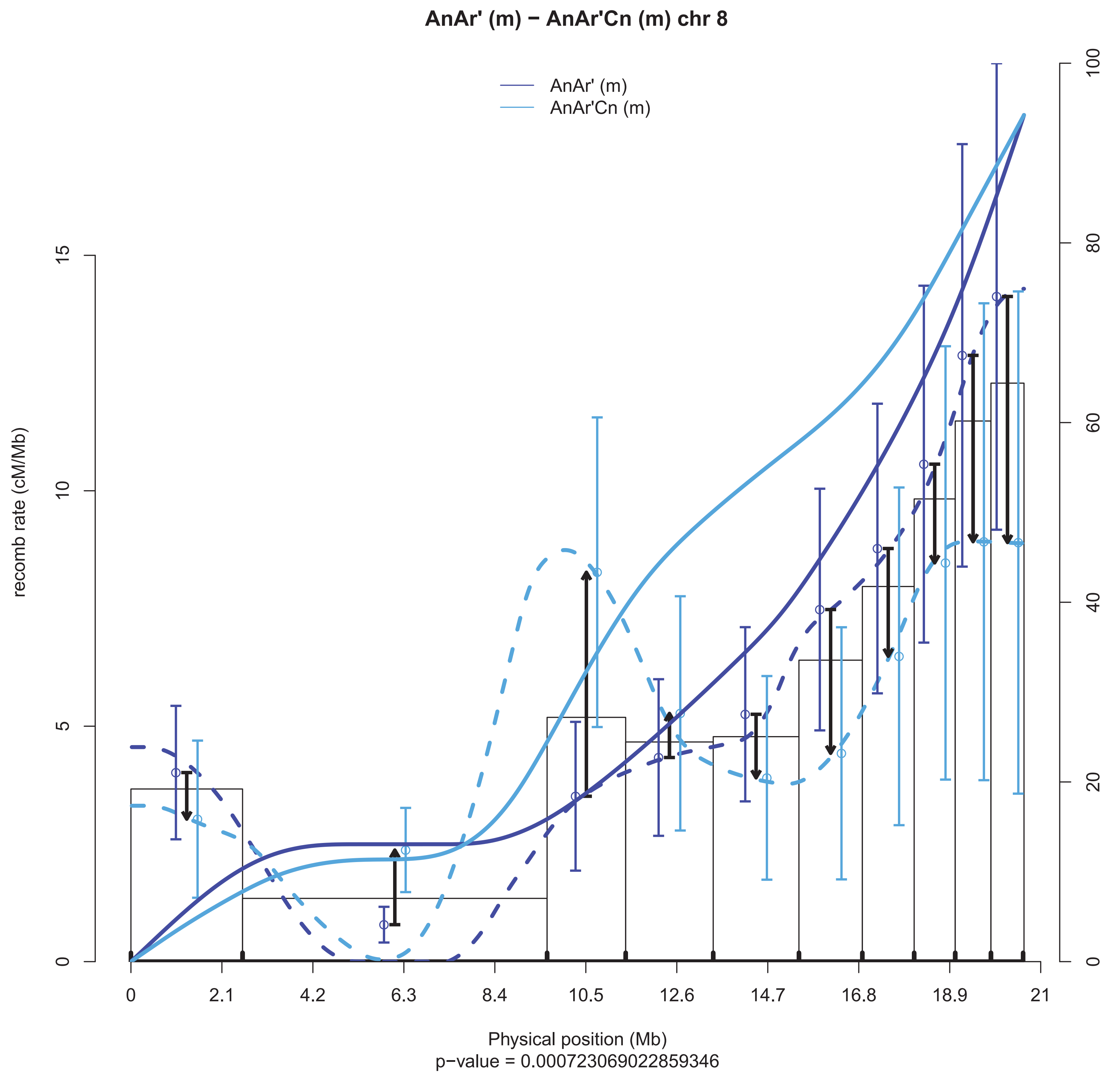

AnAr' (m) − AnAr'Cn (m) chr 8
AnAr' (m)
AnAr'Cn (m)
recomb rate (cM/Mb)
0
5
10
15
0
20
40
60
80
100
0
2.1
4.2
6.3
8.4
10.5
12.6
14.7
16.8
18.9
21
Physical position (Mb)
p−value = 0.000723069022859346

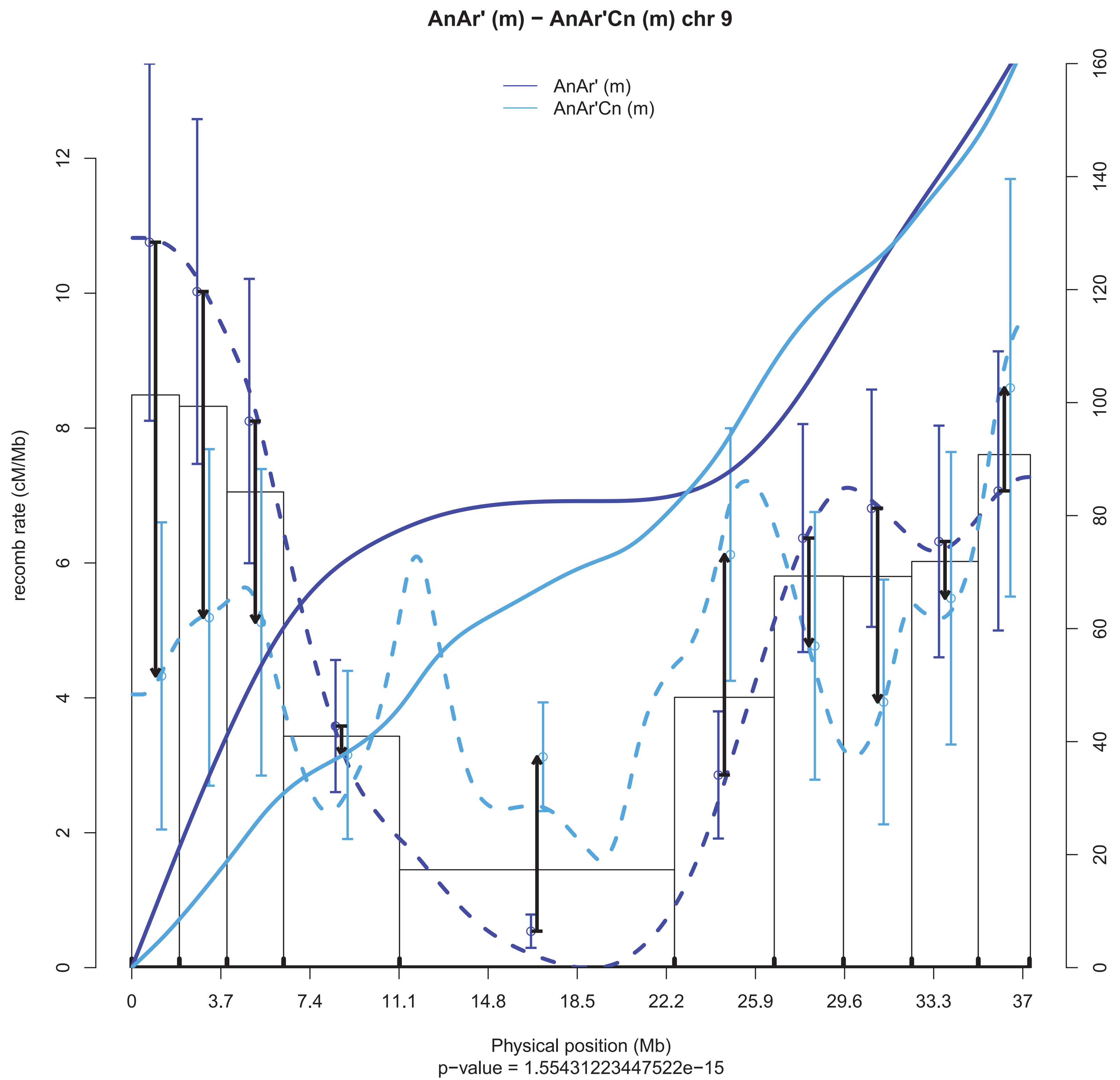
AnAr' (m) − AnAr'Cn (m) chr 9
AnAr' (m)
AnAr'Cn (m)
recomb rate (cM/Mb)
0
2
4
6
8
10
12
0
20
40
60
80
100
120
140
160
0
3.7
7.4
11.1
14.8
18.5
22.2
25.9
29.6
33.3
37
Physical position (Mb)
p−value = 1.55431223447522e−15

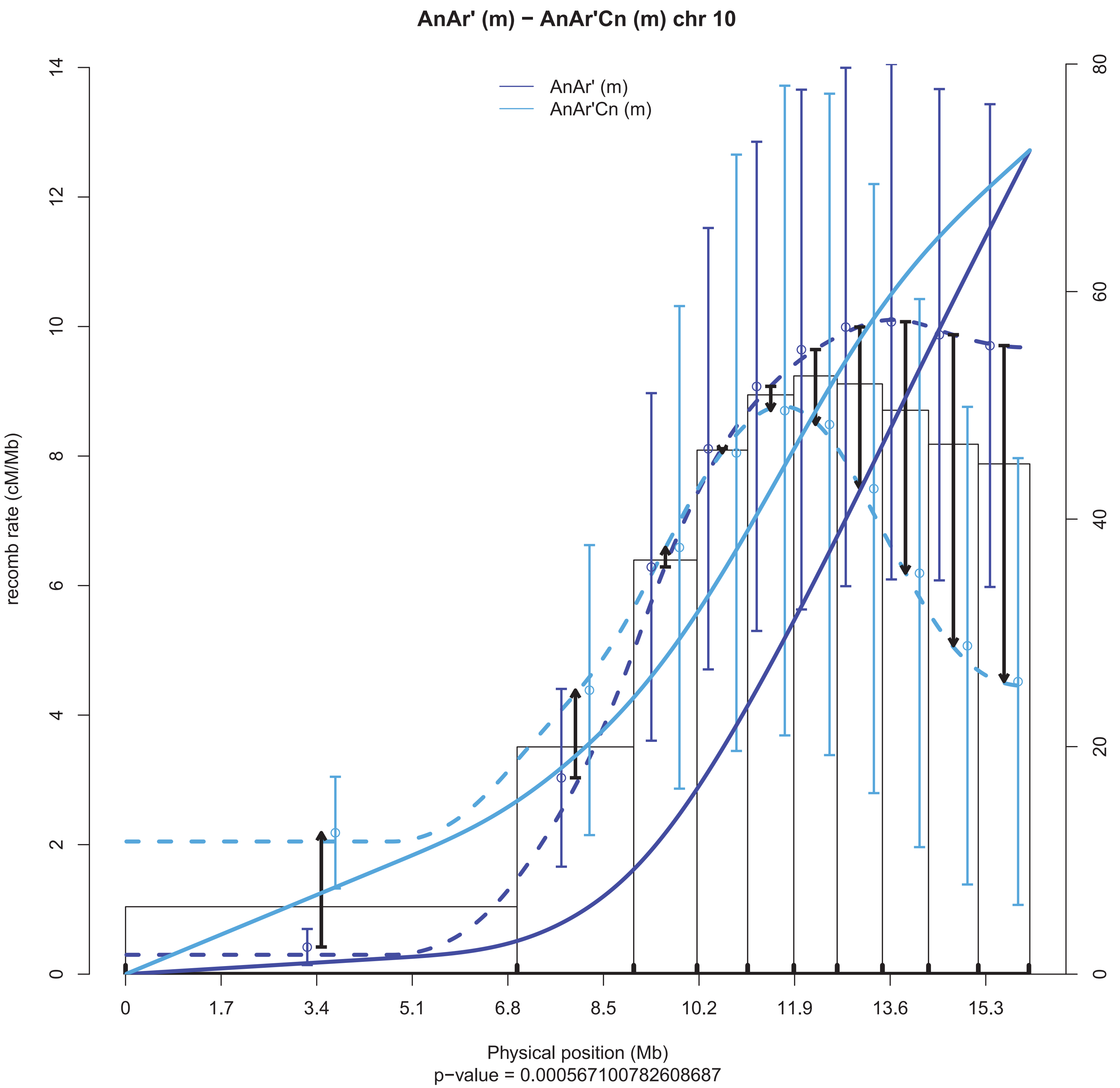

AnAr' (m) − AnAr'Cn (m) chr 10
AnAr' (m)
AnAr'Cn (m)
recomb rate (cM/Mb)
0
2
4
6
8
10
12
14
0
20
40
60
80
0
1.7
3.4
5.1
6.8
8.5
10.2
11.9
13.6
15.3
Physical position (Mb)
p−value = 0.000567100782608687
