# Supplementary material for: Amplifying recombination genome-wide and reshaping crossover landscapes in Brassicas
Source: PLoS Genet. 2017 May 11;13(5):e1006794. doi: 10.1371/journal.pgen.1006794 (PMC5444851; doi:10.1371/journal.pgen.1006794)
Supplement: S5 Fig — The chromosome length is divided into 10 bins whose length is set to ensure that all bins contain the same total number of crossover when pooling data of both populations. Solid colored lines represent the Marey maps normalized to a total arbitrary length of 100 to focus on differences in the shape of the recombination landscapes and not on differences in the values of chromosome genetic lengths. Dashed colored lines represent derivative of the Marey maps, indicating local recombination rate in cM per Mbp. Vertical colored bars indicate 95% confidence intervals of recombination rates in cM per Mbp for each map over each bin. Heavy black bars represent average recombination rate in cM per Mbp over both maps for each bin. Black arrows connect the average recombination rates of the two maps over each bin. (PDF) [file pgen.1006794.s005.pdf]

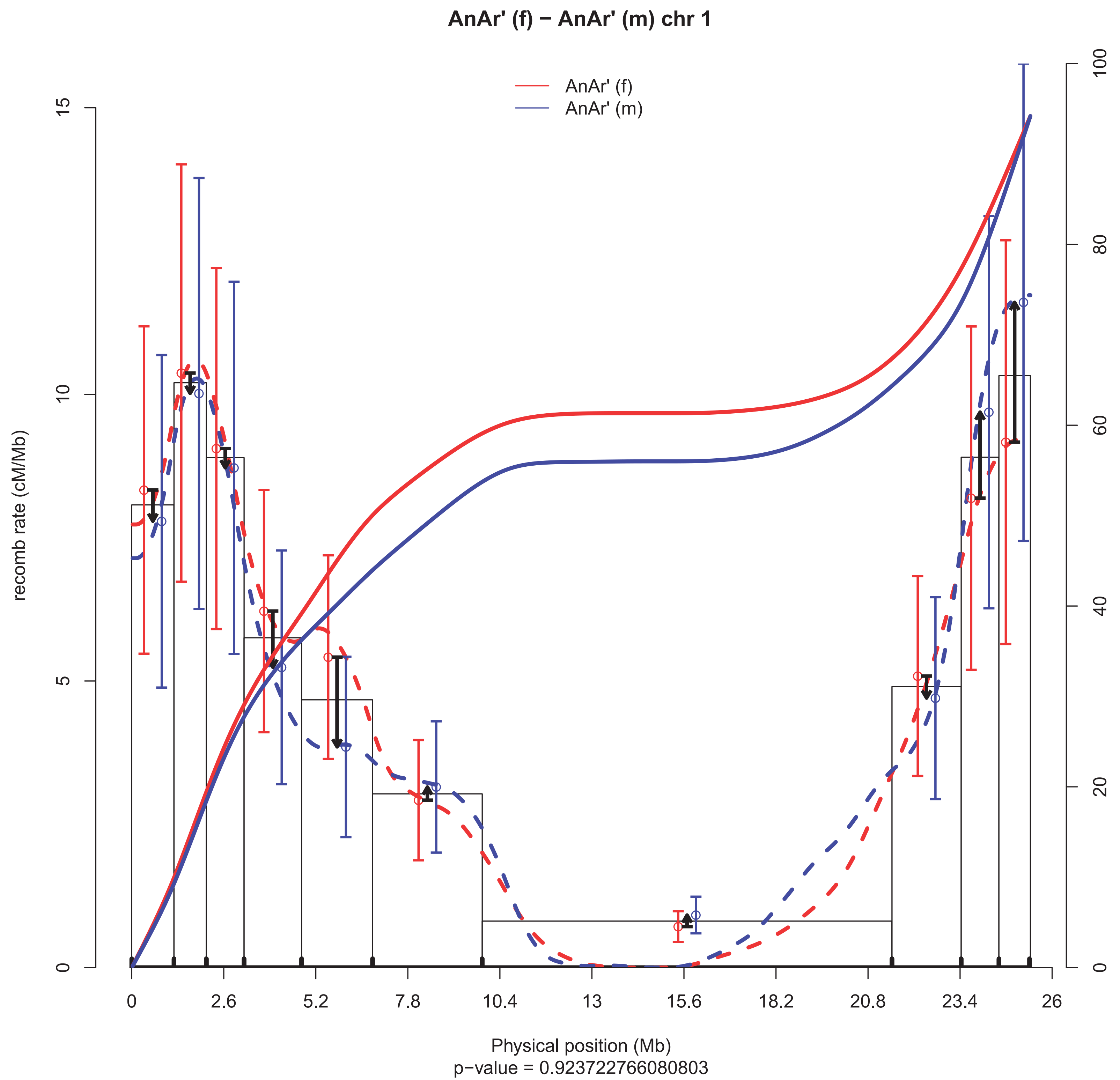

AnAr' (f) − AnAr' (m) chr 1
AnAr' (f)
AnAr' (m)
recomb rate (cM/Mb)
0
5
10
15
0
2.6
5.2
7.8
10.4
13
15.6
18.2
20.8
23.4
26
0
20
40
60
80
100
Physical position (Mb)
p−value = 0.923722766080803

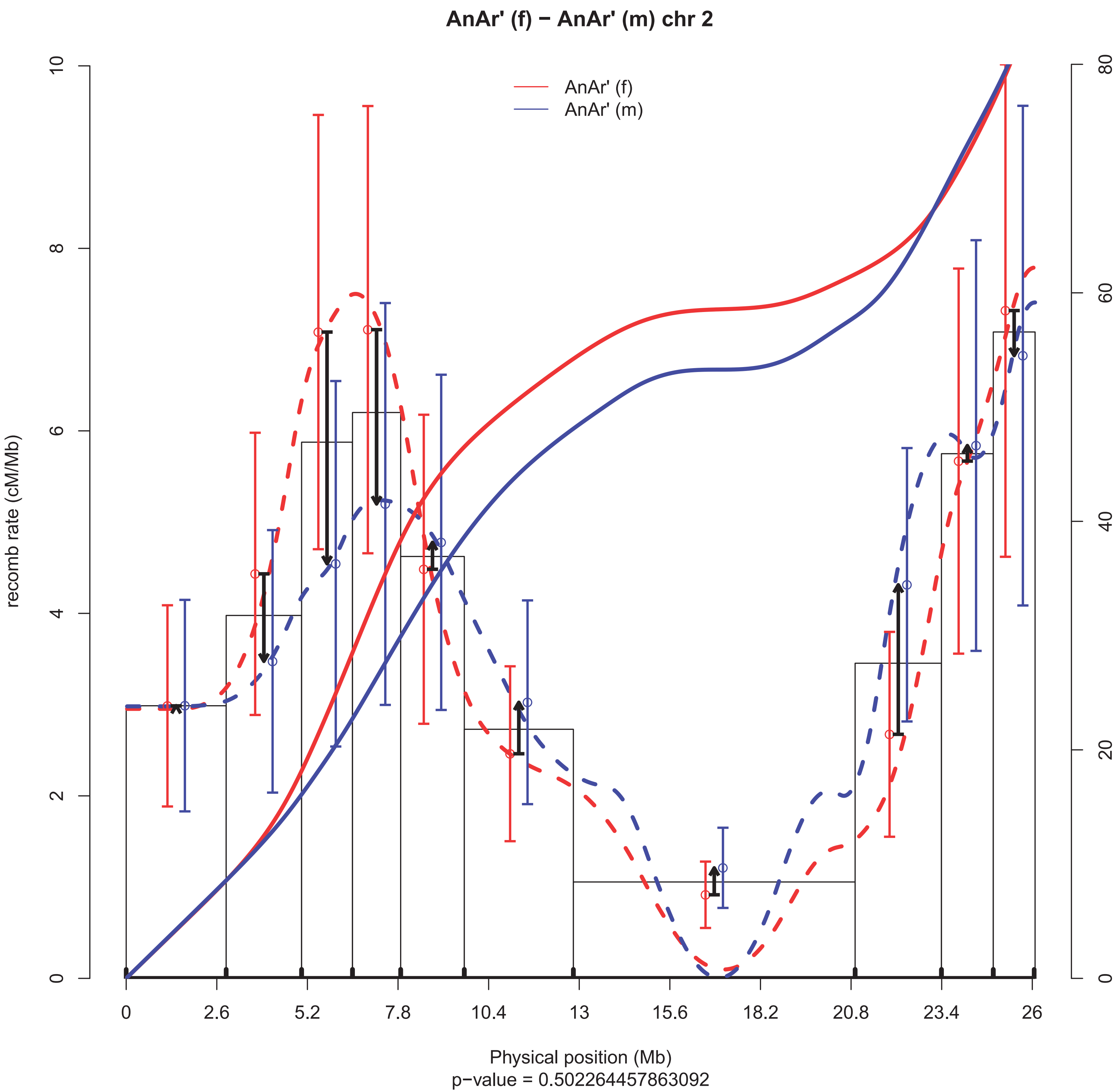

AnAr' (f) − AnAr' (m) chr 2
AnAr' (f)
AnAr' (m)
recomb rate (cM/Mb)
0
2
4
6
8
10
0
20
40
60
80
0
2.6
5.2
7.8
10.4
13
15.6
18.2
20.8
23.4
26
Physical position (Mb)
p−value = 0.502264457863092

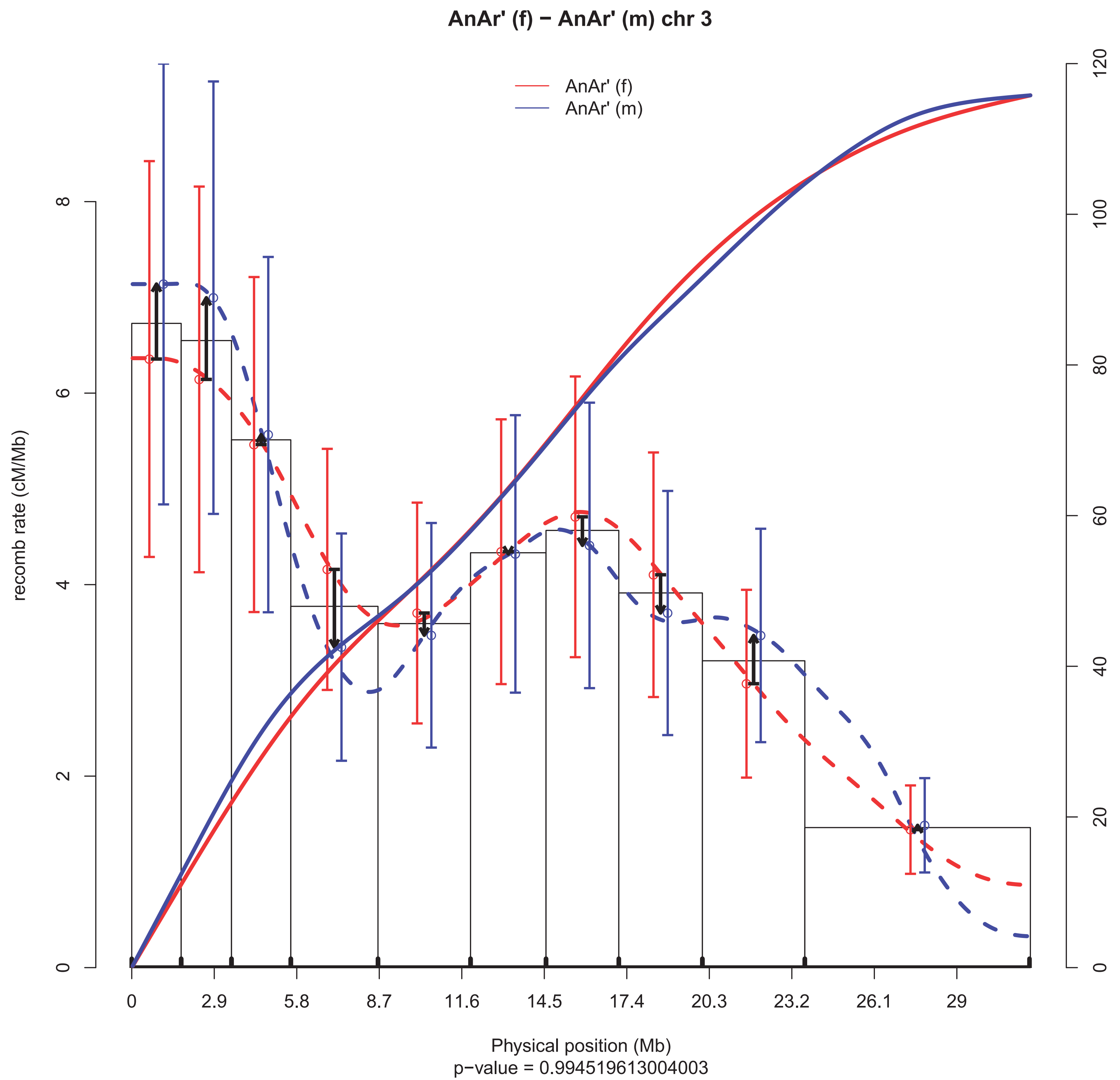

AnAr' (f) − AnAr' (m) chr 3
AnAr' (f)
AnAr' (m)
recomb rate (cM/Mb)
0
2
4
6
8
0
20
40
60
80
100
120
0
2.9
5.8
8.7
11.6
14.5
17.4
20.3
23.2
26.1
29
Physical position (Mb)
p−value = 0.994519613004003

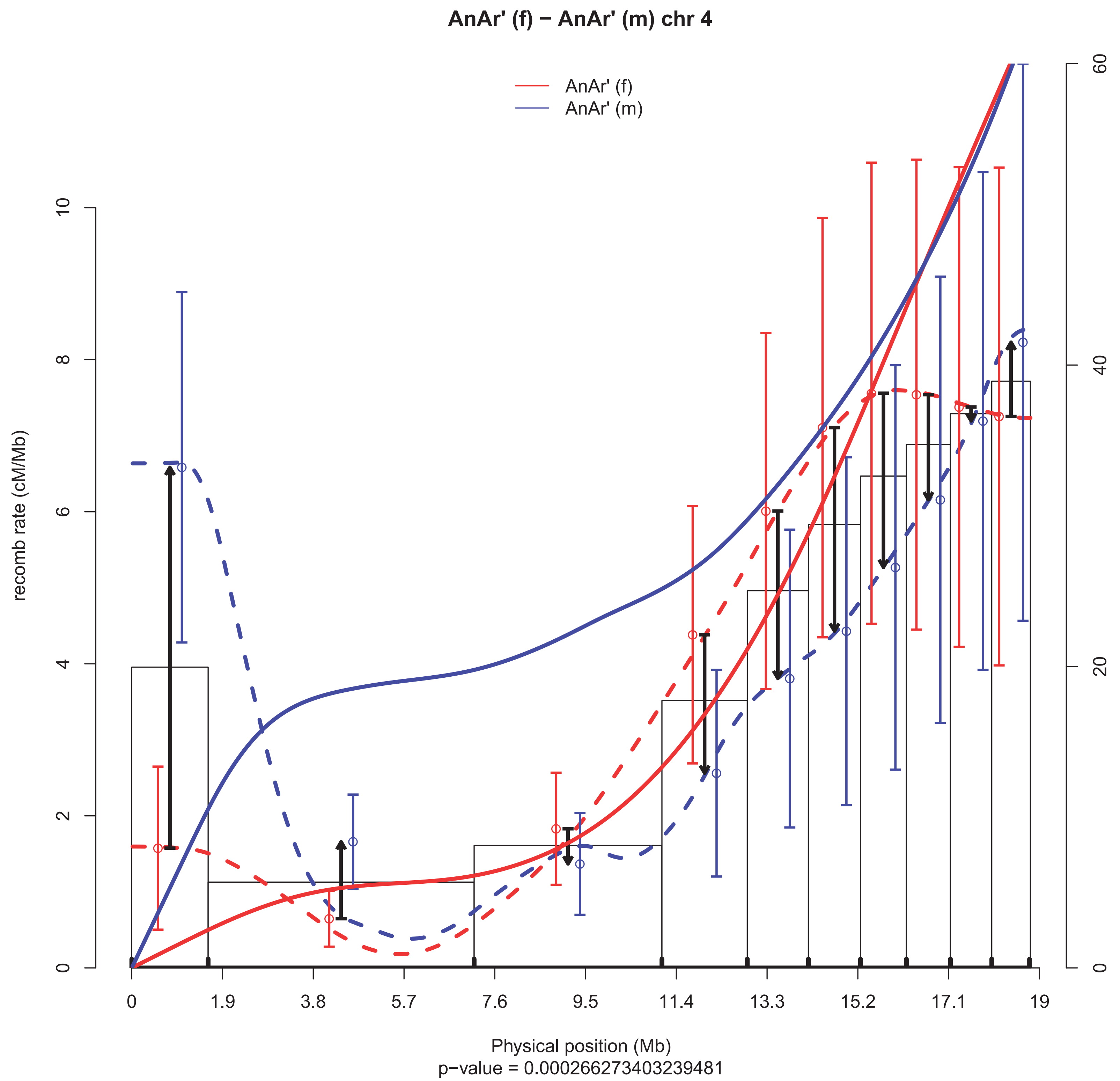
AnAr' (f) − AnAr' (m) chr 4
AnAr' (f)
AnAr' (m)
recomb rate (cM/Mb)
0
2
4
6
8
10
0
20
40
60
0
1.9
3.8
5.7
7.6
9.5
11.4
13.3
15.2
17.1
19
Physical position (Mb)
p−value = 0.000266273403239481

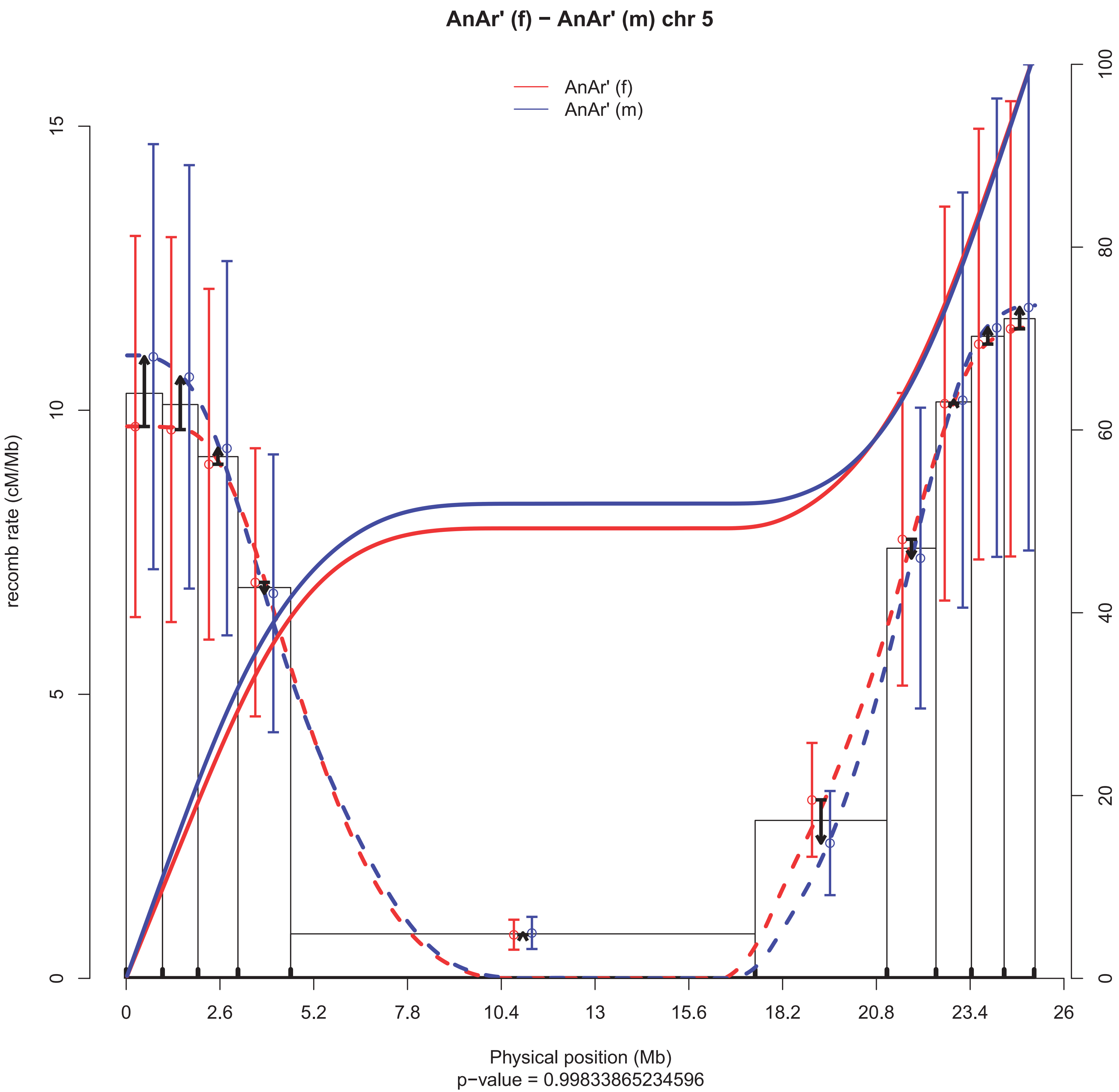

AnAr' (f) − AnAr' (m) chr 5
AnAr' (f)
AnAr' (m)
recomb rate (cM/Mb)
0
5
10
15
0
20
40
60
80
100
0
2.6
5.2
7.8
10.4
13
15.6
18.2
20.8
23.4
26
Physical position (Mb)
p−value = 0.99833865234596

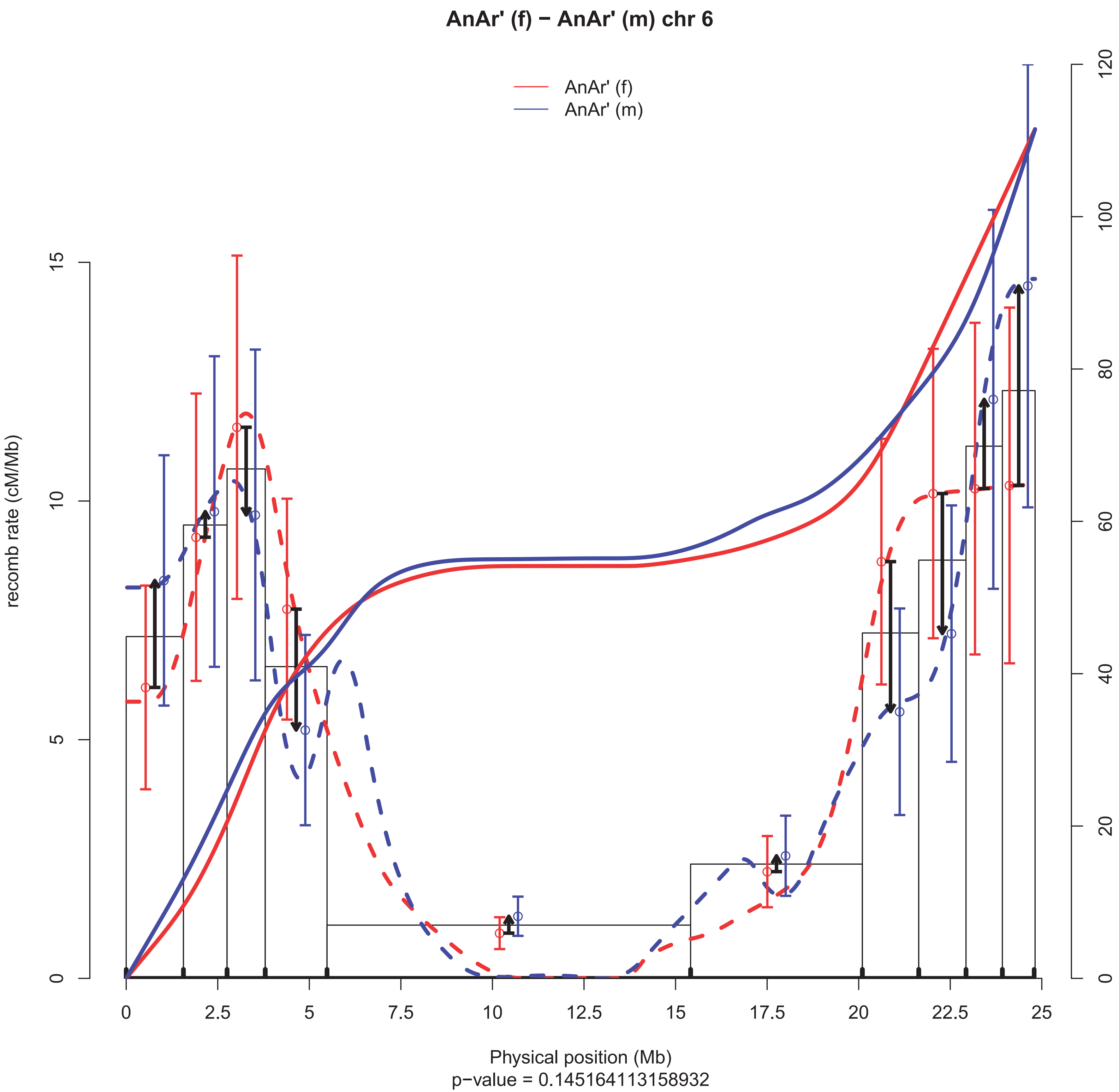
AnAr' (f) − AnAr' (m) chr 6
AnAr' (f)
AnAr' (m)
recomb rate (cM/Mb)
0
5
10
15
0
20
40
60
80
100
120
0
2.5
5
7.5
10
12.5
15
17.5
20
22.5
25
Physical position (Mb)
p−value = 0.145164113158932

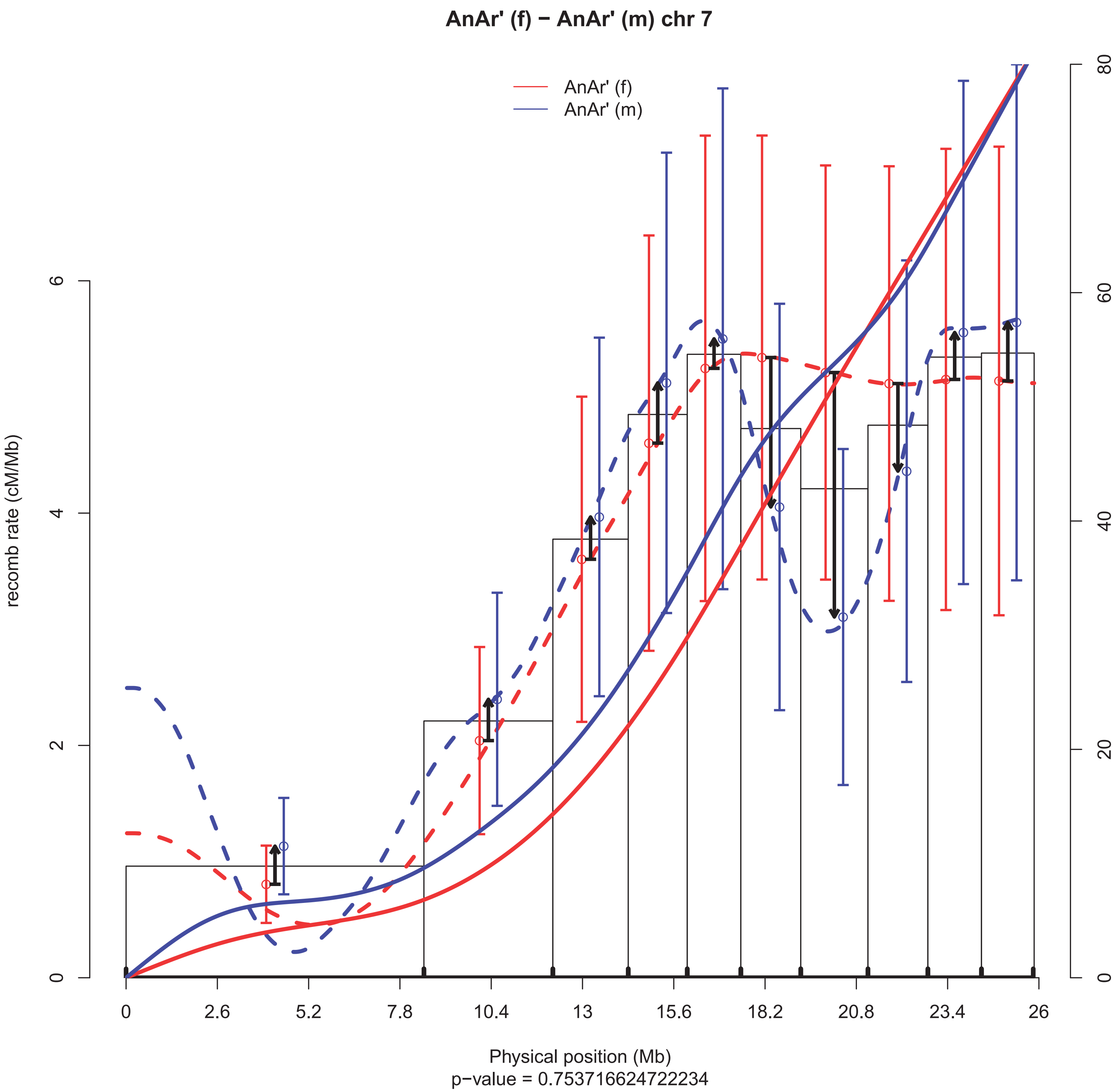

AnAr' (f) − AnAr' (m) chr 7
AnAr' (f)
AnAr' (m)
recomb rate (cM/Mb)
0
2
4
6
0
20
40
60
80
0
2.6
5.2
7.8
10.4
13
15.6
18.2
20.8
23.4
26
Physical position (Mb)
p−value = 0.753716624722234

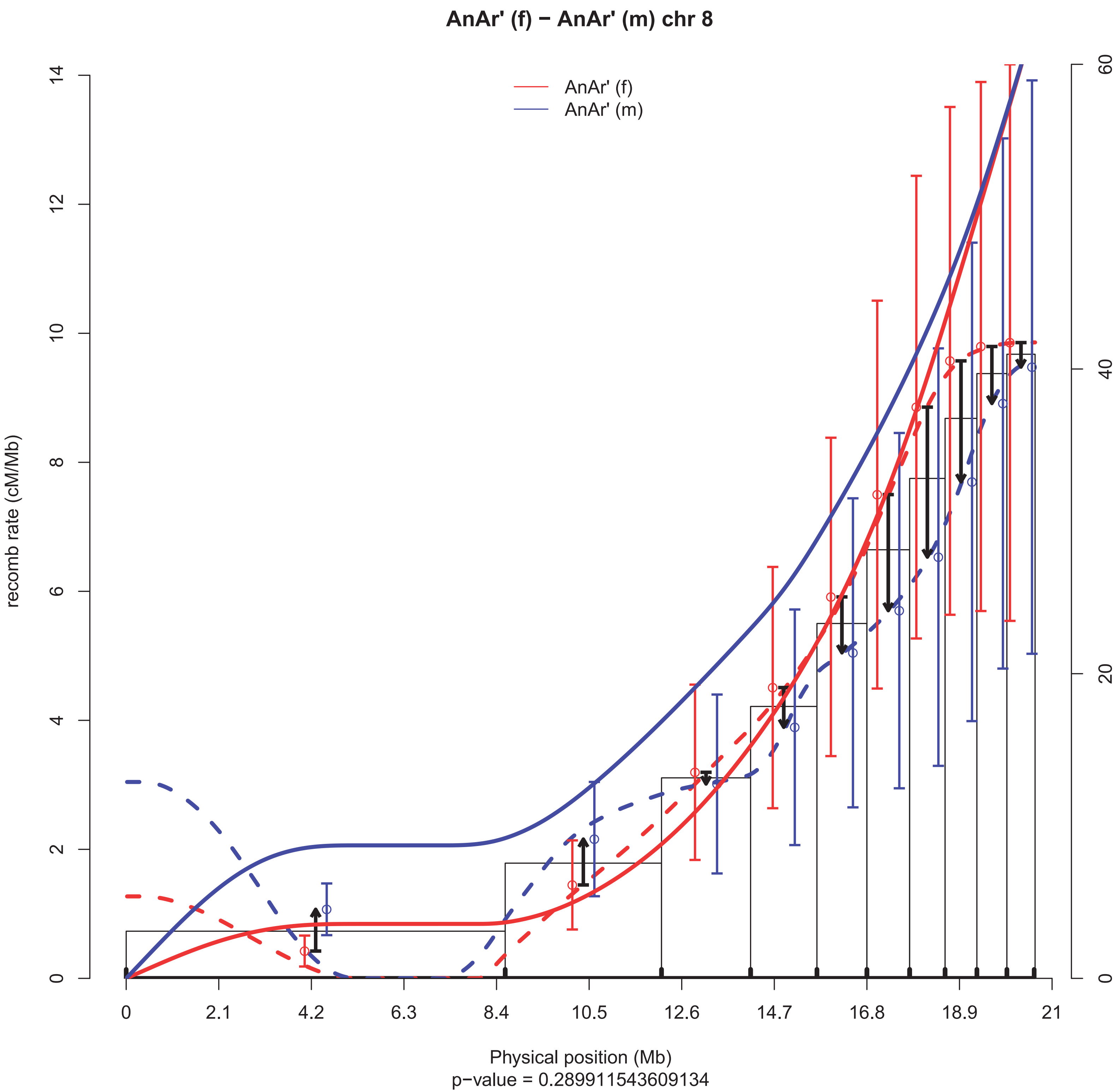
AnAr' (f) − AnAr' (m) chr 8
AnAr' (f)
AnAr' (m)
recomb rate (cM/Mb)
0
2
4
6
8
10
12
14
0
20
40
60
0
2.1
4.2
6.3
8.4
10.5
12.6
14.7
16.8
18.9
21
Physical position (Mb)
p−value = 0.289911543609134

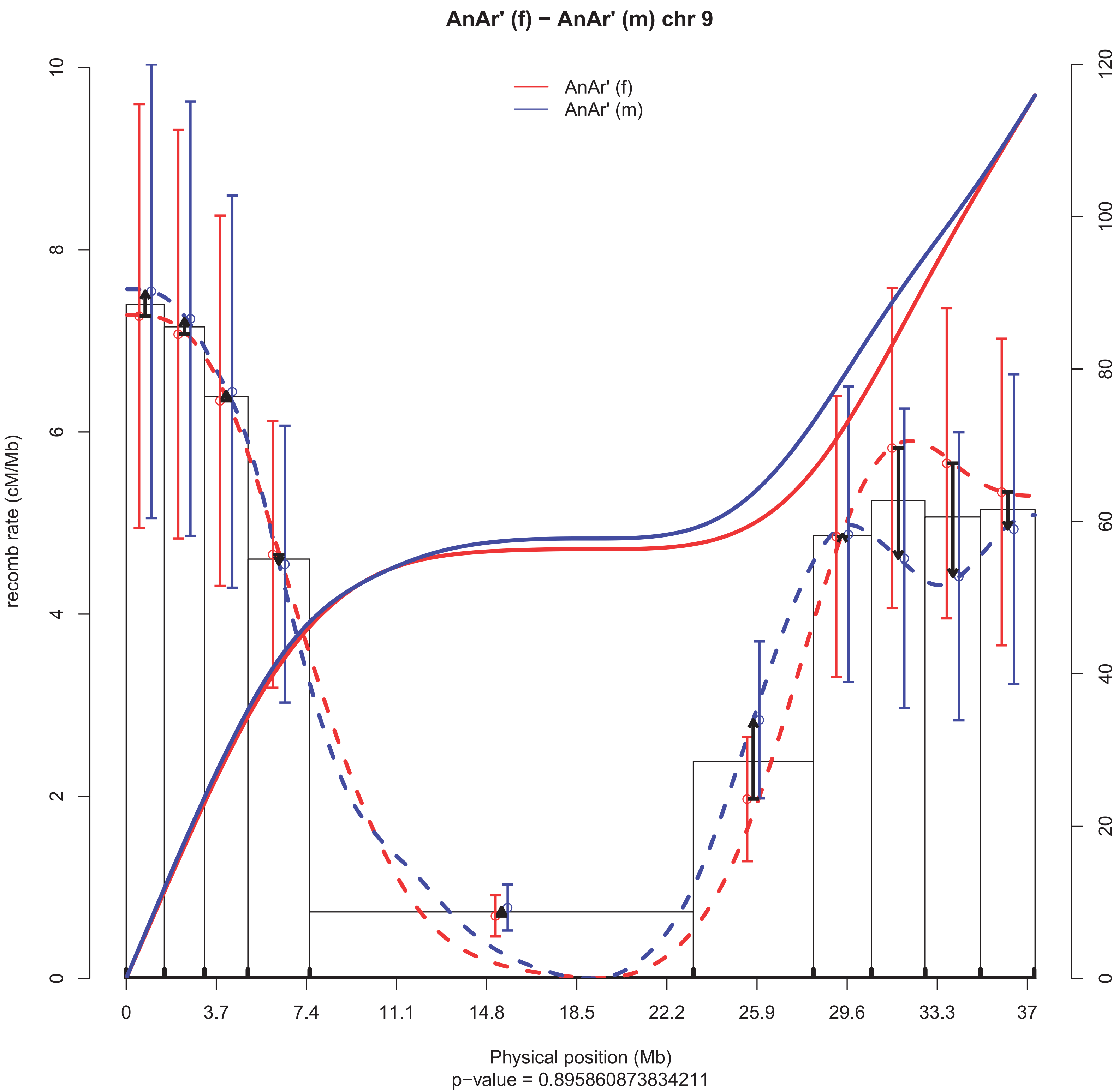
AnAr' (f) − AnAr' (m) chr 9
AnAr' (f)
AnAr' (m)
recomb rate (cM/Mb)
0
2
4
6
8
10
0
20
40
60
80
100
120
0
3.7
7.4
11.1
14.8
18.5
22.2
25.9
29.6
33.3
37
Physical position (Mb)
p−value = 0.895860873834211

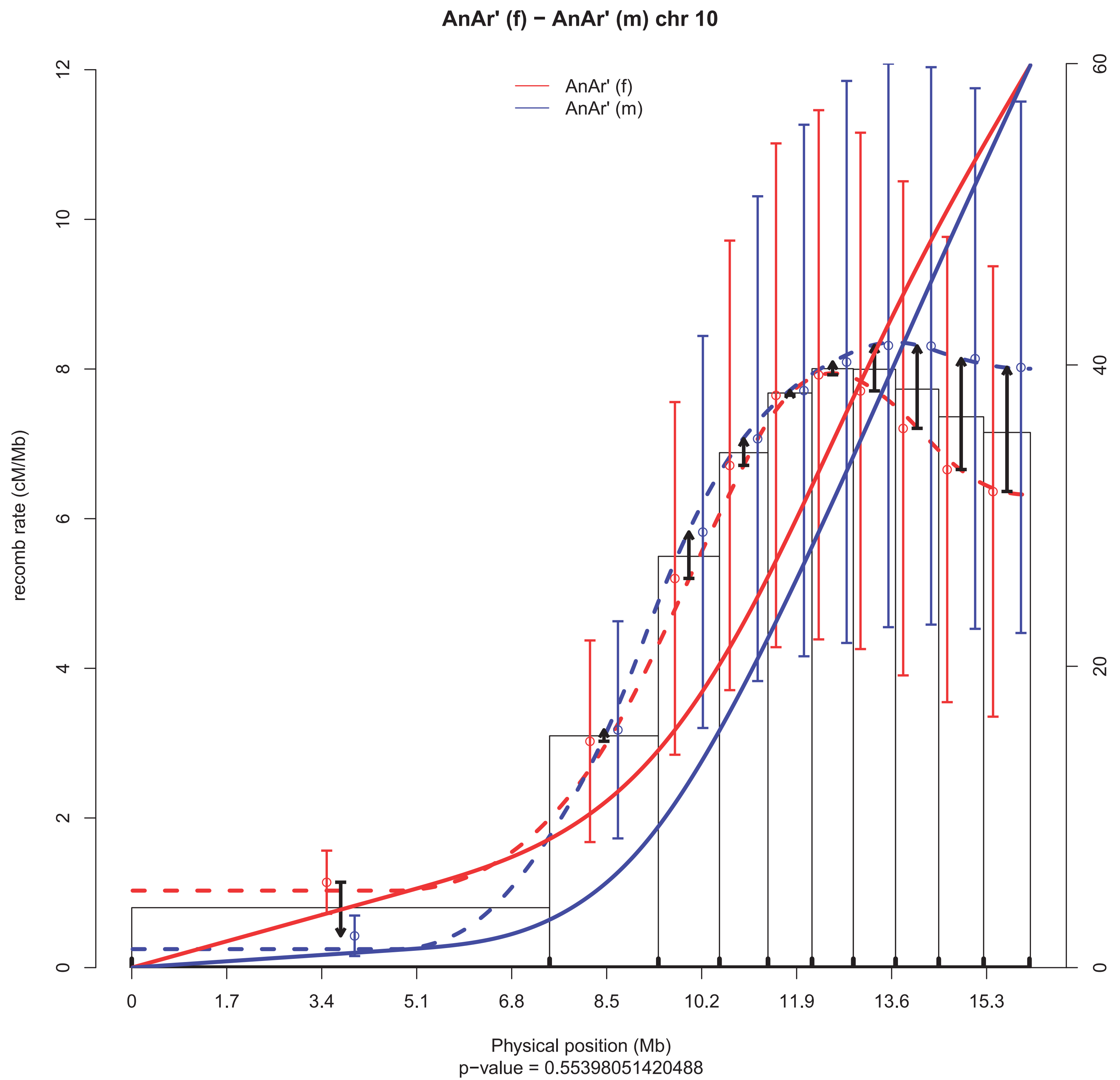

AnAr' (f) − AnAr' (m) chr 10
AnAr' (f)
AnAr' (m)
recomb rate (cM/Mb)
0
2
4
6
8
10
12
0
20
40
60
0
1.7
3.4
5.1
6.8
8.5
10.2
11.9
13.6
15.3
Physical position (Mb)
p−value = 0.55398051420488

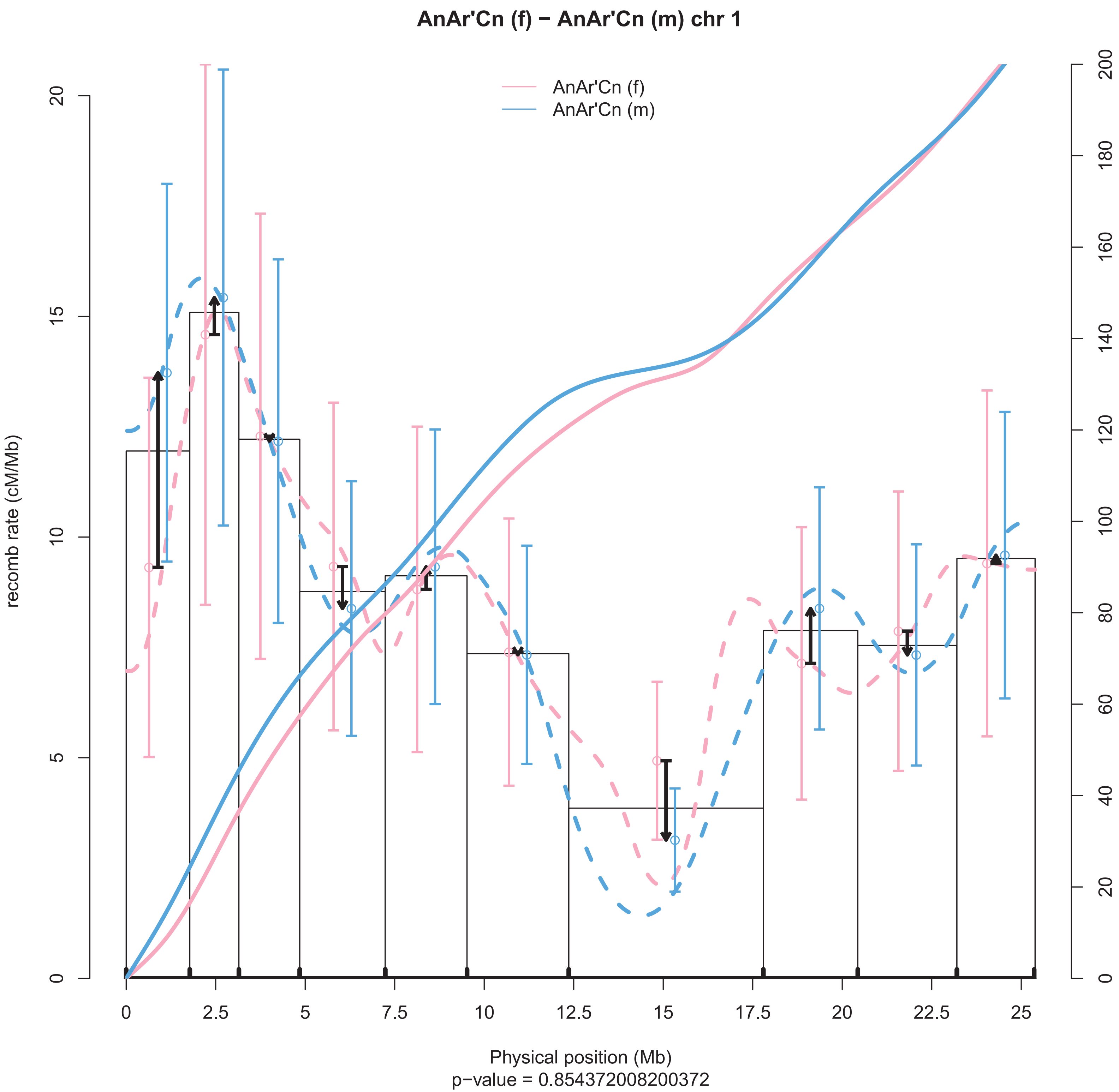
AnAr'Cn (f) − AnAr'Cn (m) chr 1
AnAr'Cn (f)
AnAr'Cn (m)
recomb rate (cM/Mb)
0
5
10
15
20
0
2.5
5
7.5
10
12.5
15
17.5
20
22.5
25
0
20
40
60
80
100
120
140
160
180
200
Physical position (Mb)
p−value = 0.854372008200372

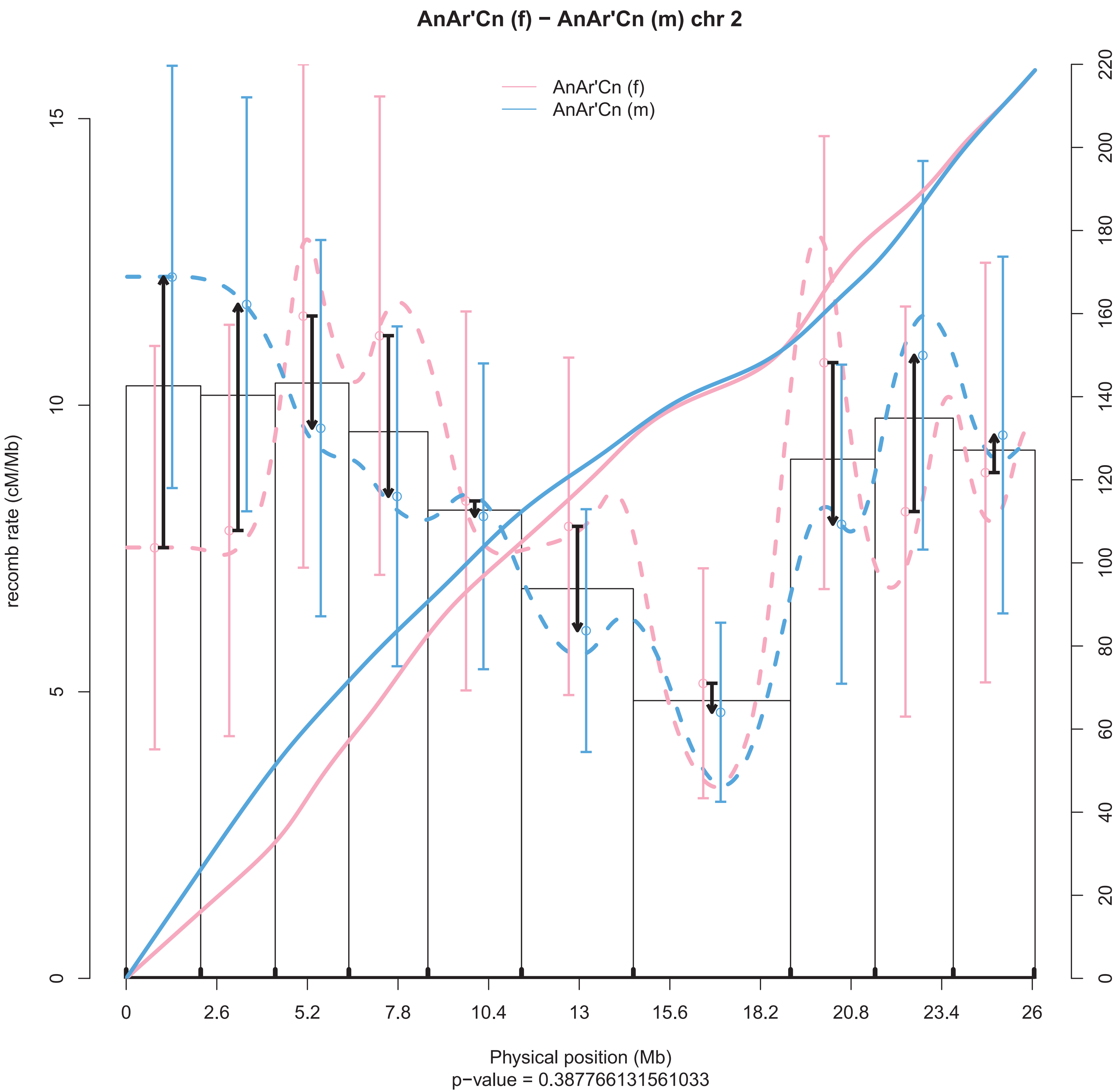
AnAr'Cn (f) − AnAr'Cn (m) chr 2
AnAr'Cn (f)
AnAr'Cn (m)
recomb rate (cM/Mb)
0
5
10
15
0
20
40
60
80
100
120
140
160
180
200
220
0
2.6
5.2
7.8
10.4
13
15.6
18.2
20.8
23.4
26
Physical position (Mb)
p−value = 0.387766131561033

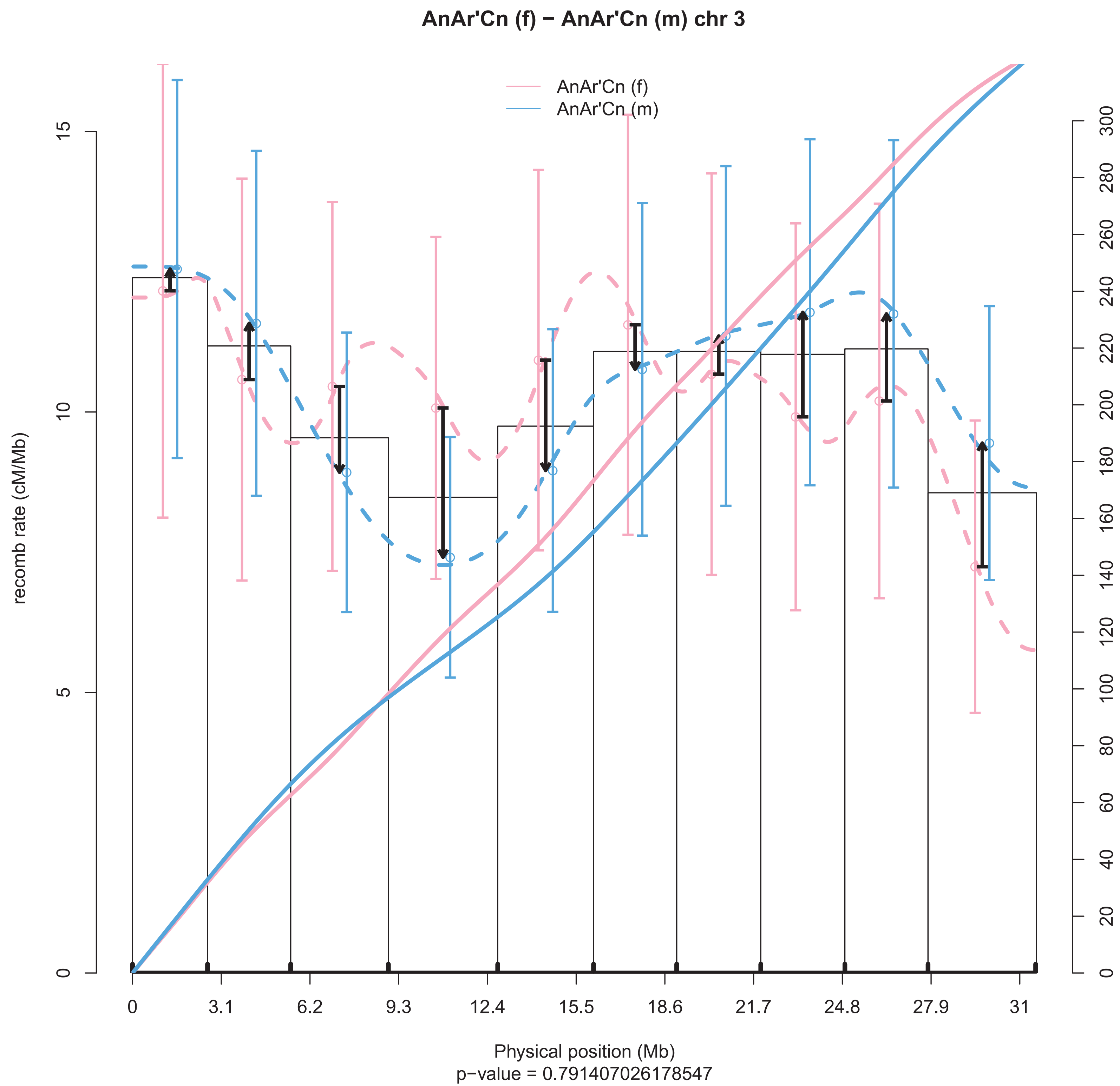

AnAr'Cn (f) − AnAr'Cn (m) chr 3
AnAr'Cn (f)
AnAr'Cn (m)
recomb rate (cM/Mb)
0
5
10
15
0
20
40
60
80
100
120
140
160
180
200
220
240
260
280
300
0
3.1
6.2
9.3
12.4
15.5
18.6
21.7
24.8
27.9
31
Physical position (Mb)
p−value = 0.791407026178547

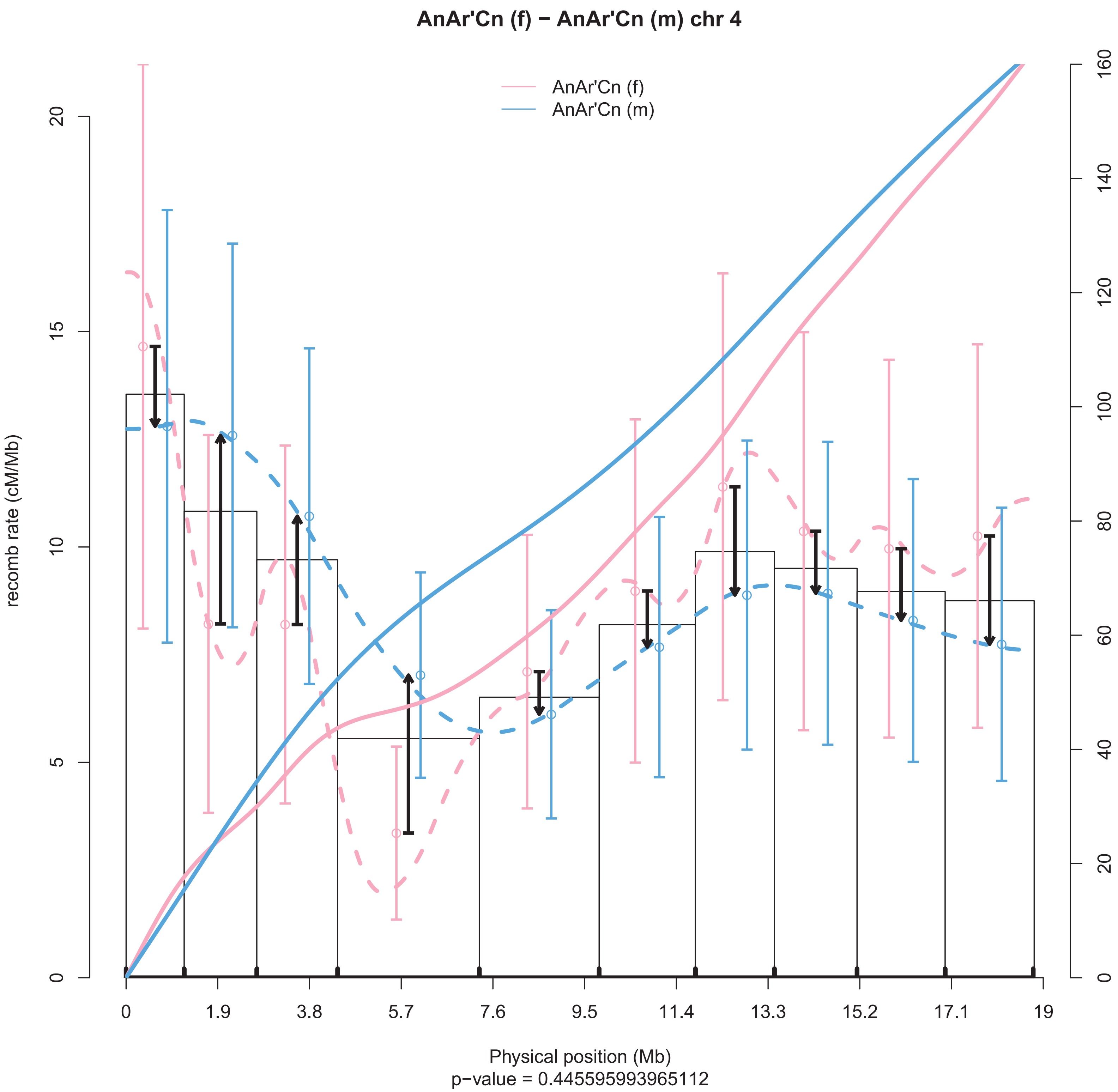
AnAr'Cn (f) − AnAr'Cn (m) chr 4
AnAr'Cn (f)
AnAr'Cn (m)
recomb rate (cM/Mb)
0
5
10
15
20
0
20
40
60
80
100
120
140
160
0
1.9
3.8
5.7
7.6
9.5
11.4
13.3
15.2
17.1
19
Physical position (Mb)
p−value = 0.445595993965112

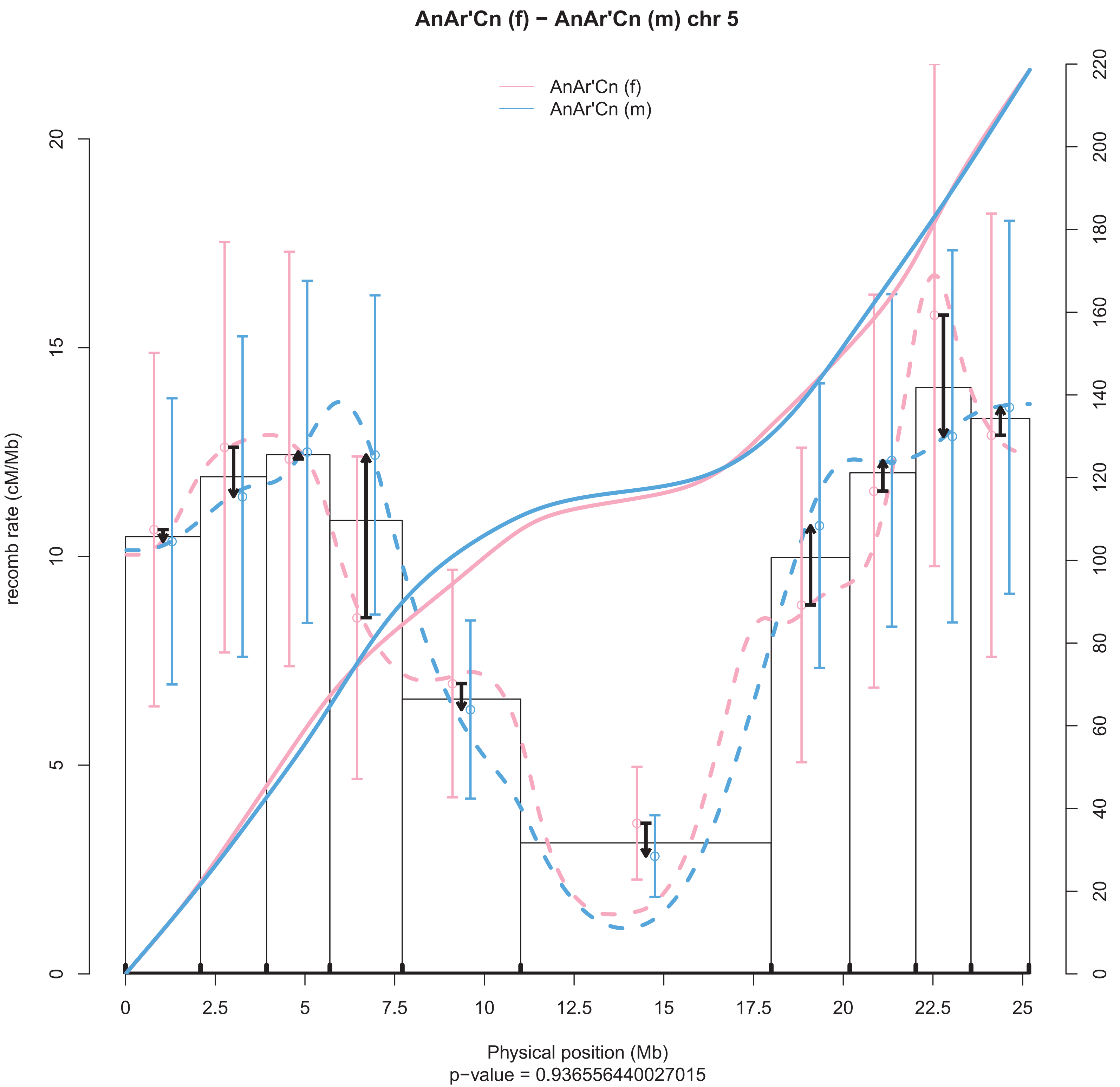

AnAr'Cn (f) − AnAr'Cn (m) chr 5
AnAr'Cn (f)
AnAr'Cn (m)
recomb rate (cM/Mb)
0
5
10
15
20
0
20
40
60
80
100
120
140
160
180
200
220
0
2.5
5
7.5
10
12.5
15
17.5
20
22.5
25
Physical position (Mb)
p−value = 0.936556440027015

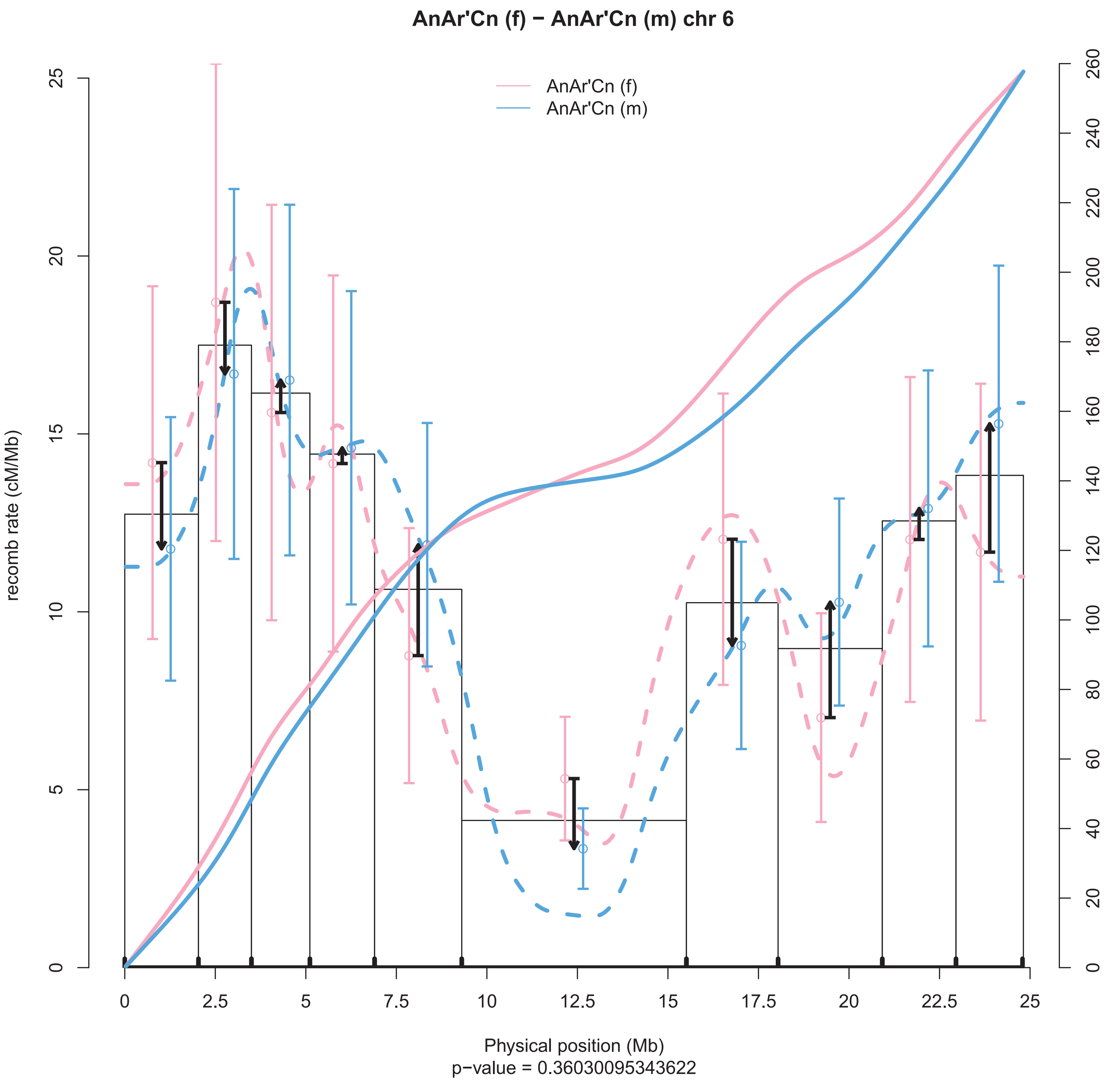

AnAr'Cn (f) − AnAr'Cn (m) chr 6
AnAr'Cn (f)
AnAr'Cn (m)
recomb rate (cM/Mb)
0
5
10
15
20
25
0
20
40
60
80
100
120
140
160
180
200
220
240
260
0
2.5
5
7.5
10
12.5
15
17.5
20
22.5
25
Physical position (Mb)
p−value = 0.36030095343622

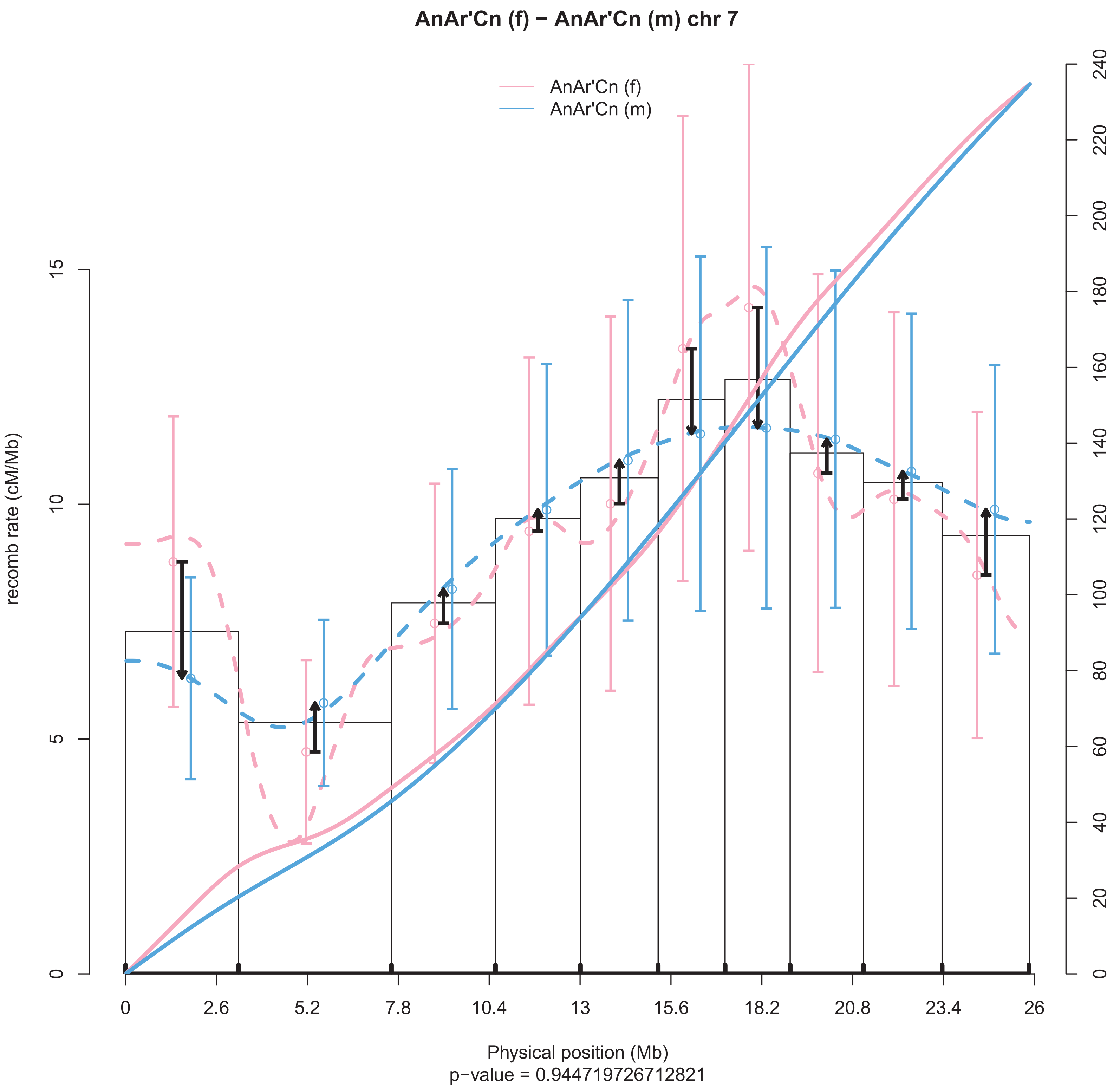
AnAr'Cn (f) − AnAr'Cn (m) chr 7
AnAr'Cn (f)
AnAr'Cn (m)
recomb rate (cM/Mb)
0
5
10
15
0
20
40
60
80
100
120
140
160
180
200
220
240
0
2.6
5.2
7.8
10.4
13
15.6
18.2
20.8
23.4
26
Physical position (Mb)
p−value = 0.944719726712821

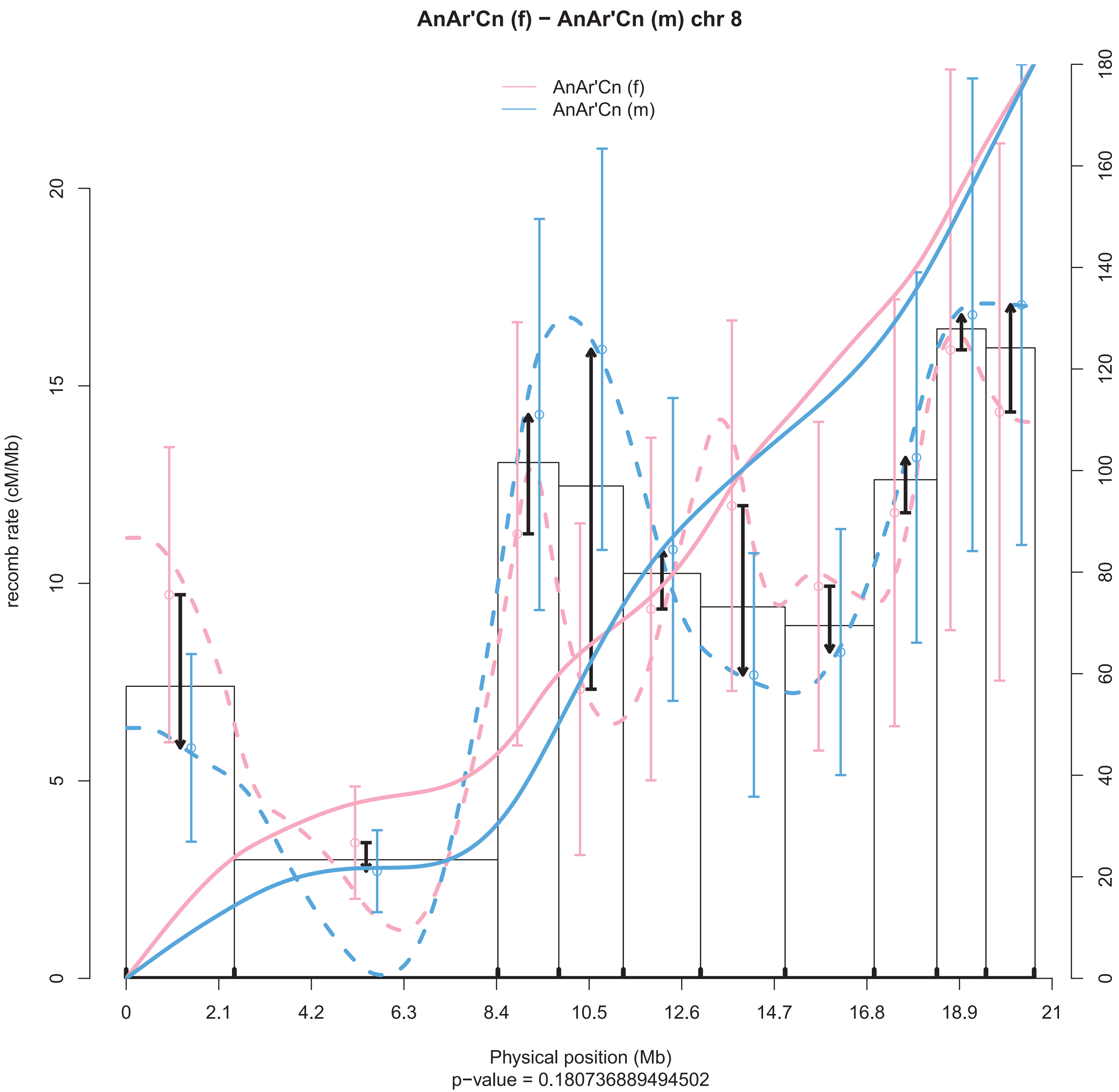
AnAr'Cn (f) − AnAr'Cn (m) chr 8
AnAr'Cn (f)
AnAr'Cn (m)
recomb rate (cM/Mb)
0
5
10
15
20
0
20
40
60
80
100
120
140
160
180
0
2.1
4.2
6.3
8.4
10.5
12.6
14.7
16.8
18.9
21
Physical position (Mb)
p−value = 0.180736889494502

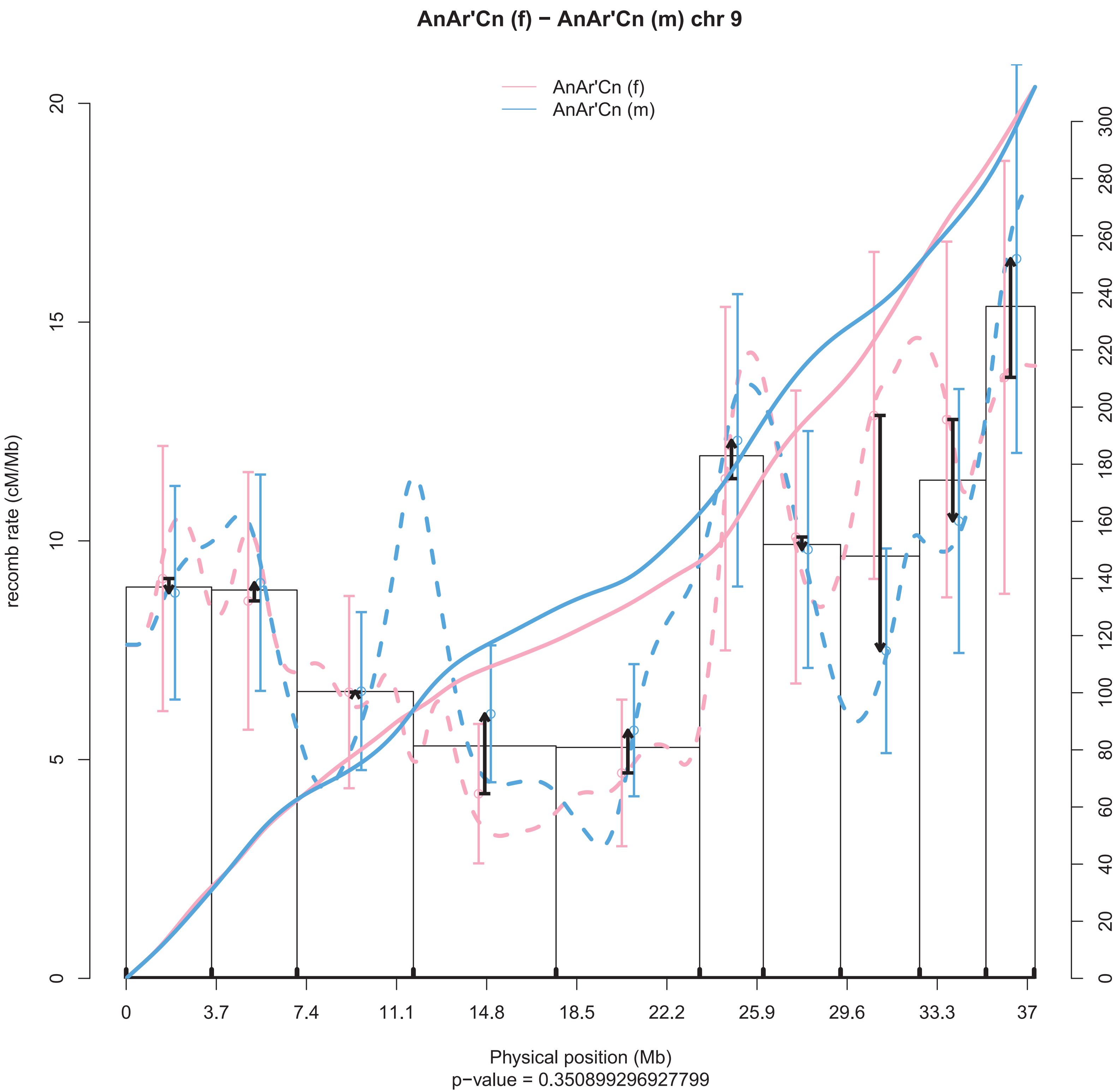

AnAr'Cn (f) − AnAr'Cn (m) chr 9
AnAr'Cn (f)
AnAr'Cn (m)
recomb rate (cM/Mb)
0
5
10
15
20
0
20
40
60
80
100
120
140
160
180
200
220
240
260
280
300
0
3.7
7.4
11.1
14.8
18.5
22.2
25.9
29.6
33.3
37
Physical position (Mb)
p−value = 0.350899296927799

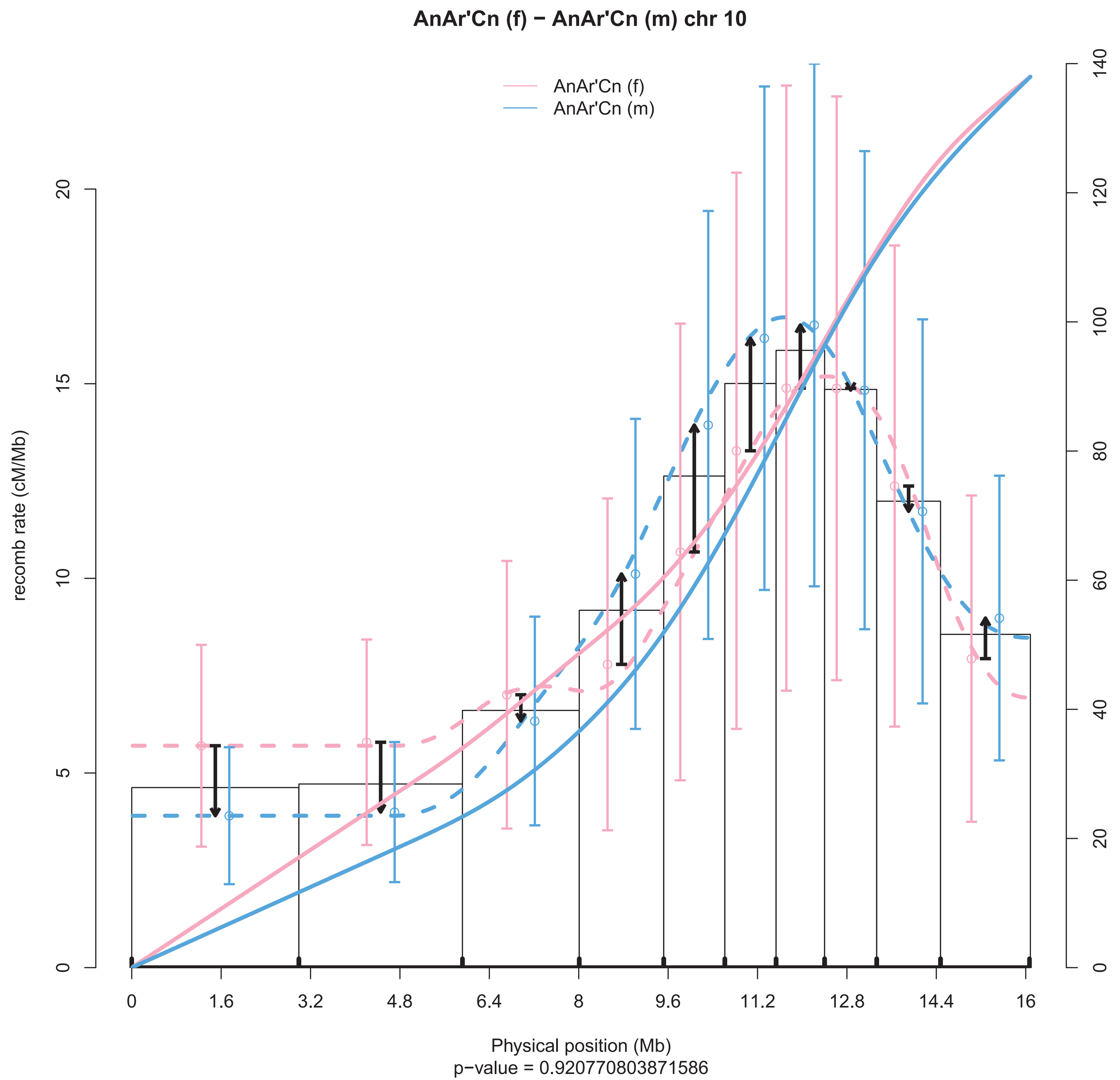

AnAr'Cn (f) − AnAr'Cn (m) chr 10
AnAr'Cn (f)
AnAr'Cn (m)
recomb rate (cM/Mb)
0
5
10
15
20
0
20
40
60
80
100
120
140
0
1.6
3.2
4.8
6.4
8
9.6
11.2
12.8
14.4
16
Physical position (Mb)
p−value = 0.920770803871586
