# Supplementary material for: Amplifying recombination genome-wide and reshaping crossover landscapes in Brassicas
Source: PLoS Genet. 2017 May 11;13(5):e1006794. doi: 10.1371/journal.pgen.1006794 (PMC5444851; doi:10.1371/journal.pgen.1006794)
Supplement: S6 Fig — The chromosome length is divided into 10 bins whose length is set to ensure that all bins contain the same total number of crossover when pooling data of both populations. Solid colored lines represent the Marey maps normalized to a total arbitrary length of 100 to focus on differences in the shape of the recombination landscapes and not on differences in the values of chromosome genetic lengths. Dashed colored lines represent derivative of the Marey maps, indicating local recombination rate in cM per Mbp. Vertical colored bars indicate 95% confidence intervals of recombination rates in cM per Mbp for each map over each bin. Heavy black bars represent average recombination rate in cM per Mbp over both maps for each bin. Black arrows connect the average recombination rates of the two maps over each bin. (PDF) [file pgen.1006794.s006.pdf]

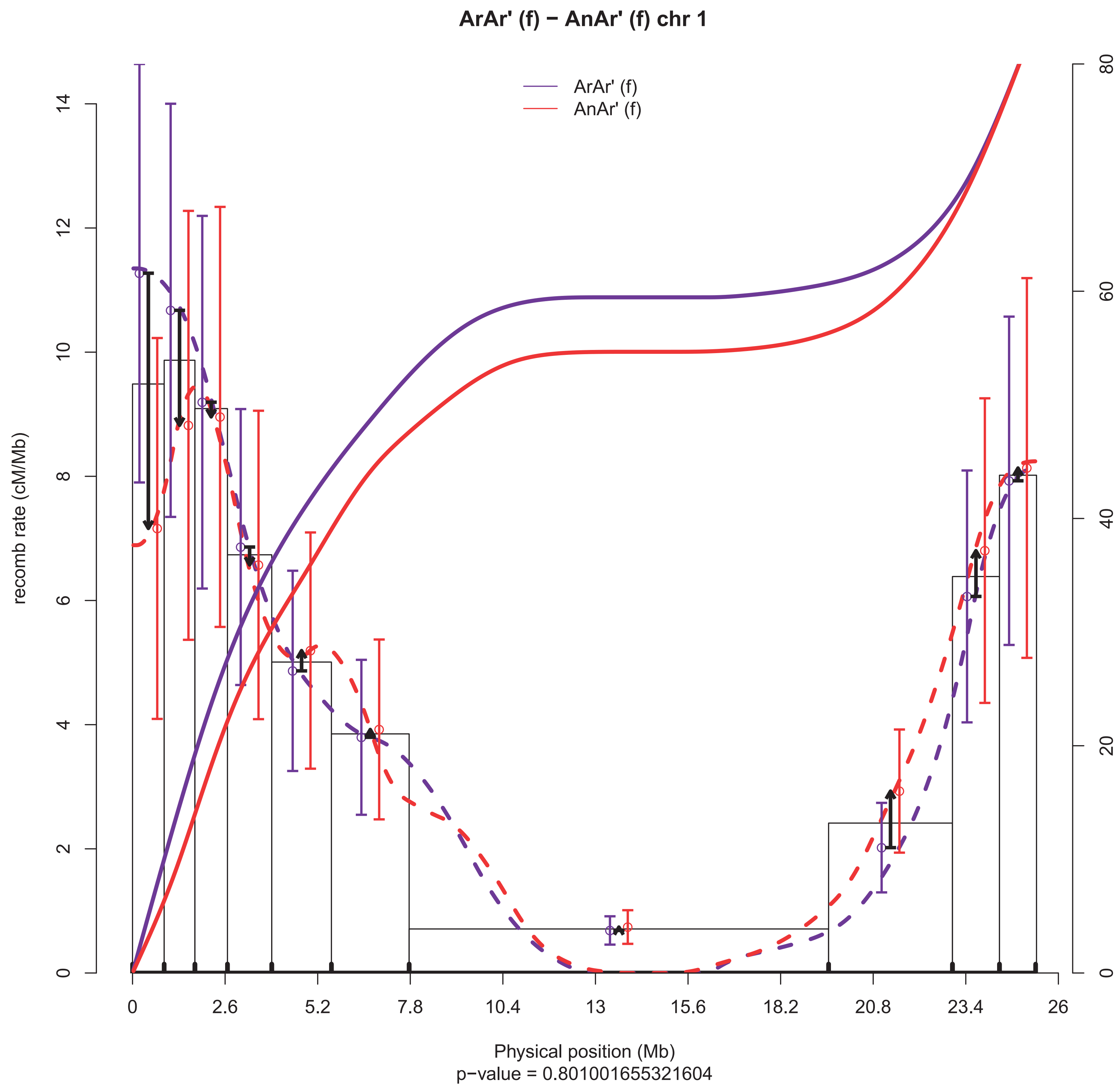

ArAr' (f) − AnAr' (f) chr 1
ArAr' (f)
AnAr' (f)
recomb rate (cM/Mb)
0
2
4
6
8
10
12
14
0
20
40
60
80
0
2.6
5.2
7.8
10.4
13
15.6
18.2
20.8
23.4
26
Physical position (Mb)
p−value = 0.801001655321604

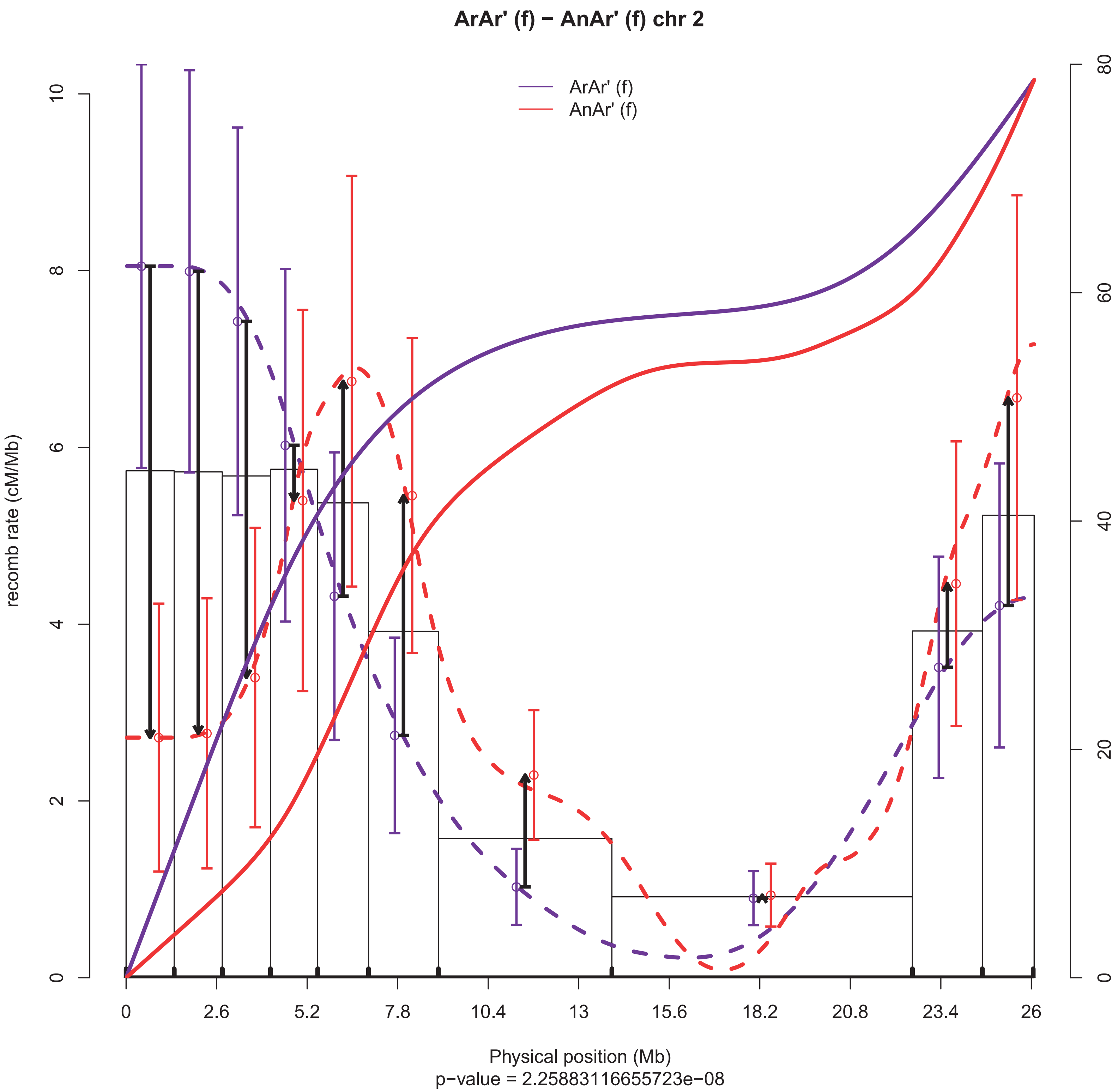
ArAr' (f) − AnAr' (f) chr 2
ArAr' (f)
AnAr' (f)
recomb rate (cM/Mb)
0
2
4
6
8
10
0
20
40
60
80
0
2.6
5.2
7.8
10.4
13
15.6
18.2
20.8
23.4
26
Physical position (Mb)
p−value = 2.25883116655723e−08

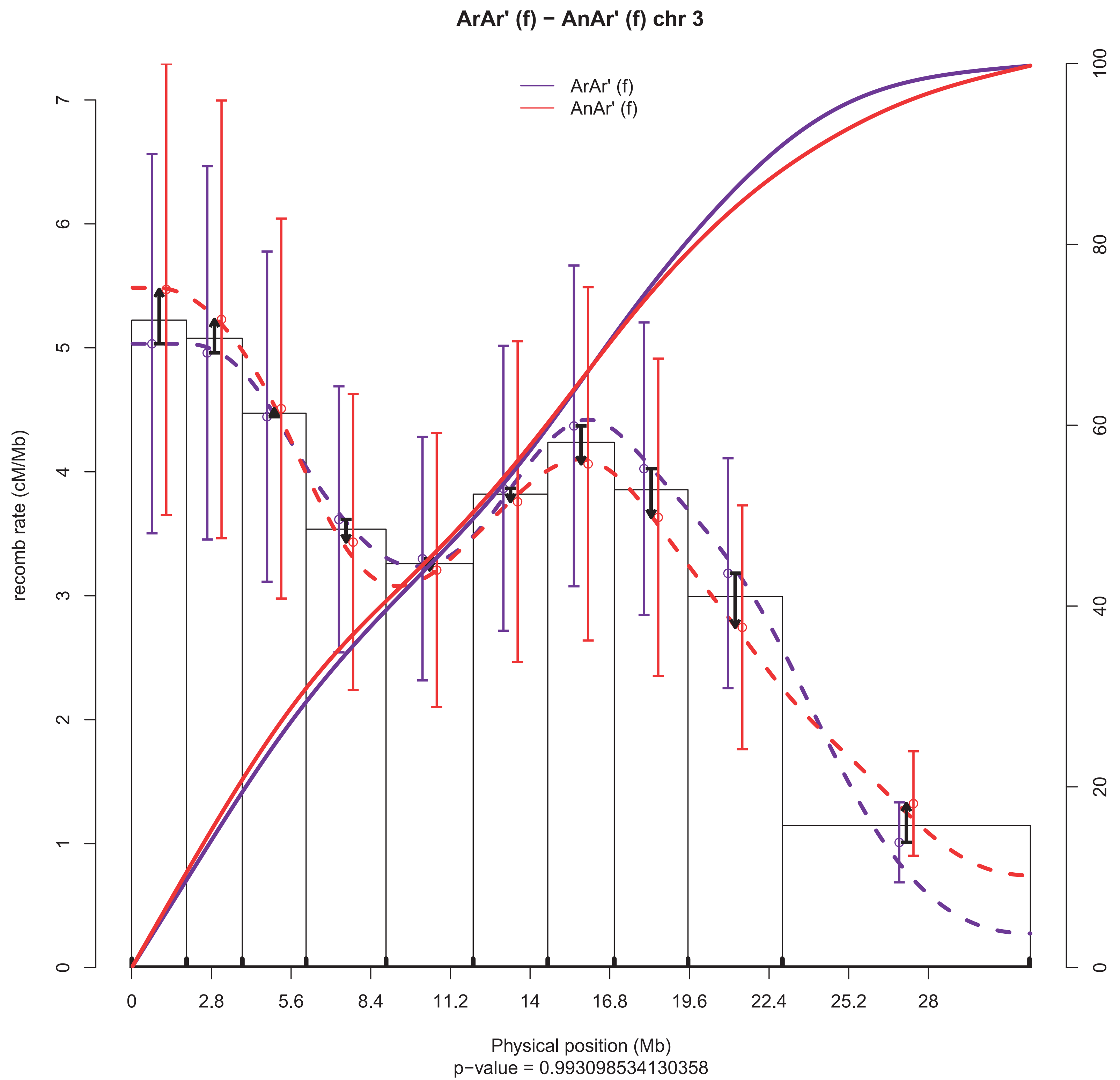

ArAr' (f) − AnAr' (f) chr 3
ArAr' (f)
AnAr' (f)
recomb rate (cM/Mb)
0
1
2
3
4
5
6
7
0
20
40
60
80
100
0
2.8
5.6
8.4
11.2
14
16.8
19.6
22.4
25.2
28
Physical position (Mb)
p−value = 0.993098534130358

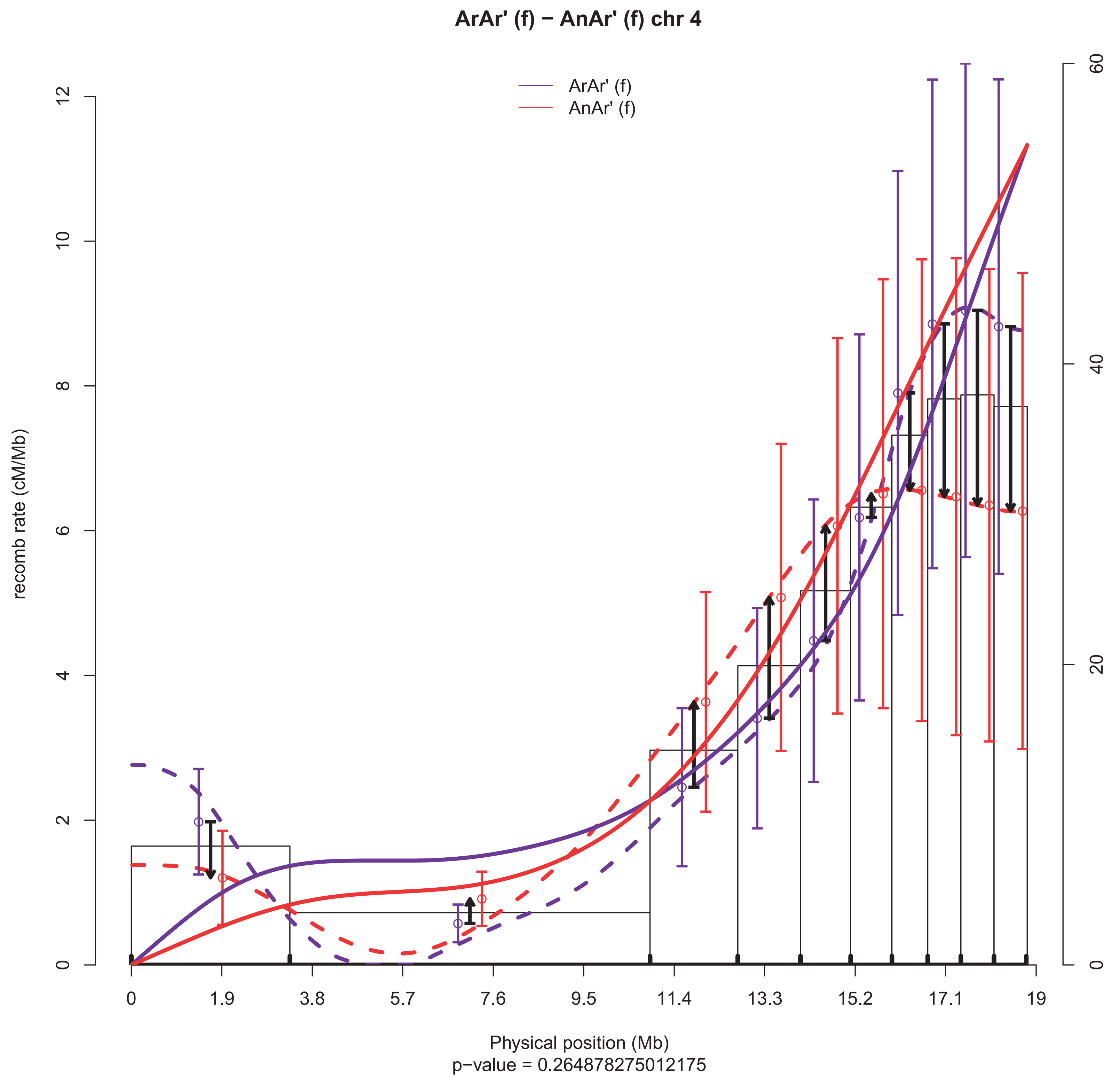

ArAr' (f) − AnAr' (f) chr 4
ArAr' (f)
AnAr' (f)
recomb rate (cM/Mb)
0
2
4
6
8
10
12
0
20
40
60
0
1.9
3.8
5.7
7.6
9.5
11.4
13.3
15.2
17.1
19
Physical position (Mb)
p−value = 0.264878275012175

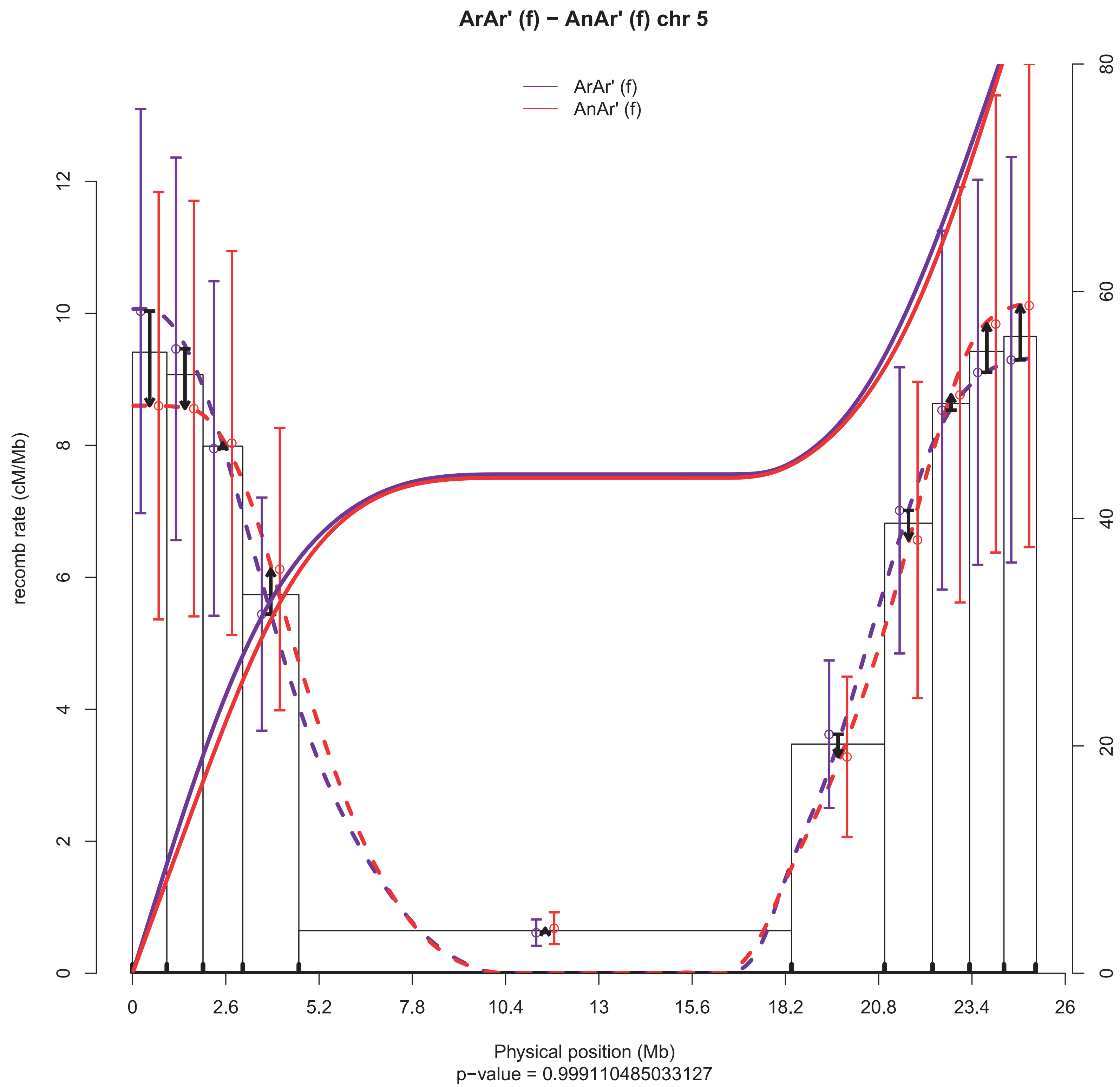
ArAr' (f) − AnAr' (f) chr 5
ArAr' (f)
AnAr' (f)
recomb rate (cM/Mb)
0
2
4
6
8
10
12
0
20
40
60
80
0
2.6
5.2
7.8
10.4
13
15.6
18.2
20.8
23.4
26
Physical position (Mb)
p−value = 0.999110485033127

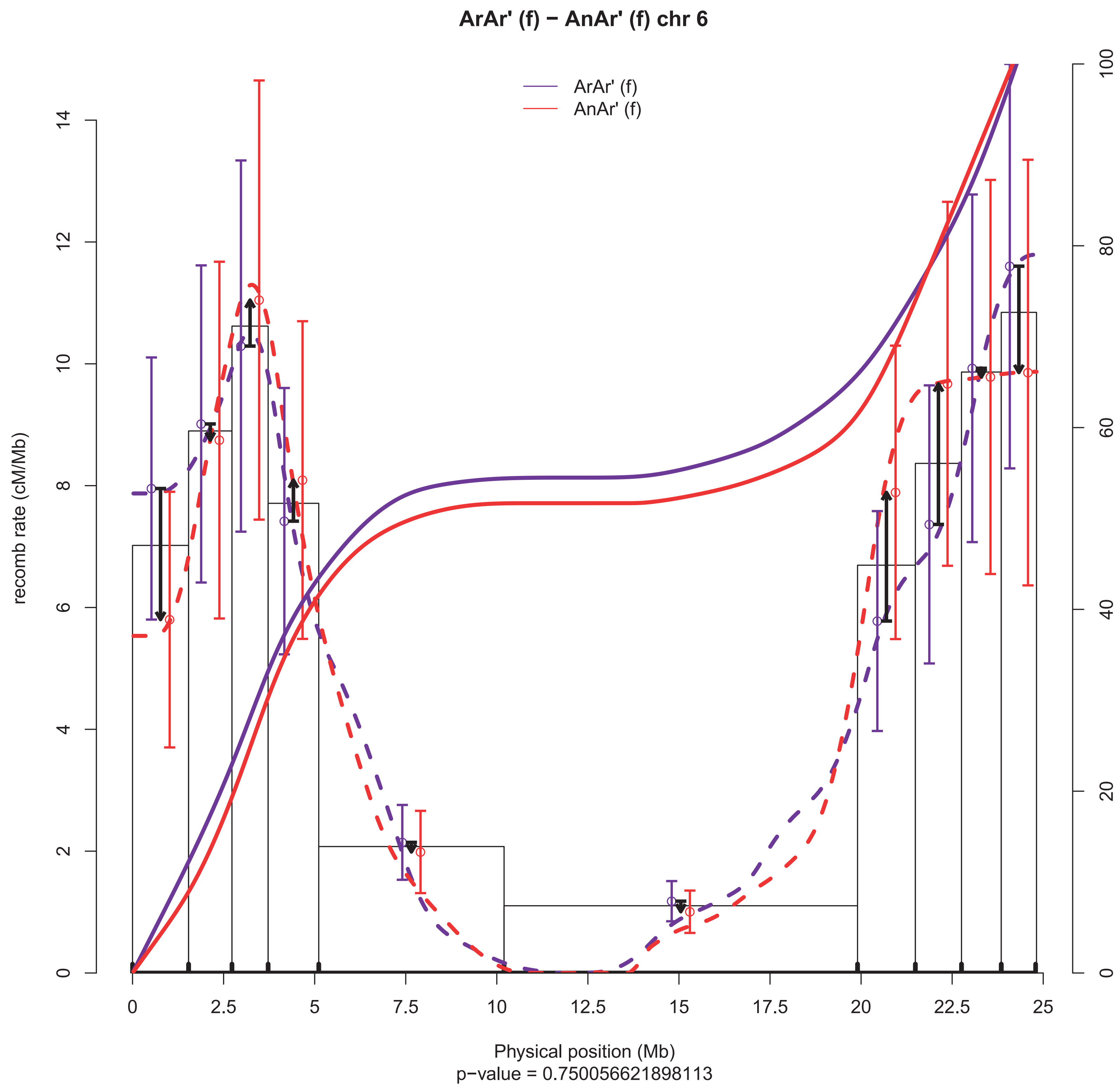

ArAr' (f) − AnAr' (f) chr 6
ArAr' (f)
AnAr' (f)
recomb rate (cM/Mb)
0
2
4
6
8
10
12
14
0
20
40
60
80
100
0
2.5
5
7.5
10
12.5
15
17.5
20
22.5
25
Physical position (Mb)
p−value = 0.750056621898113

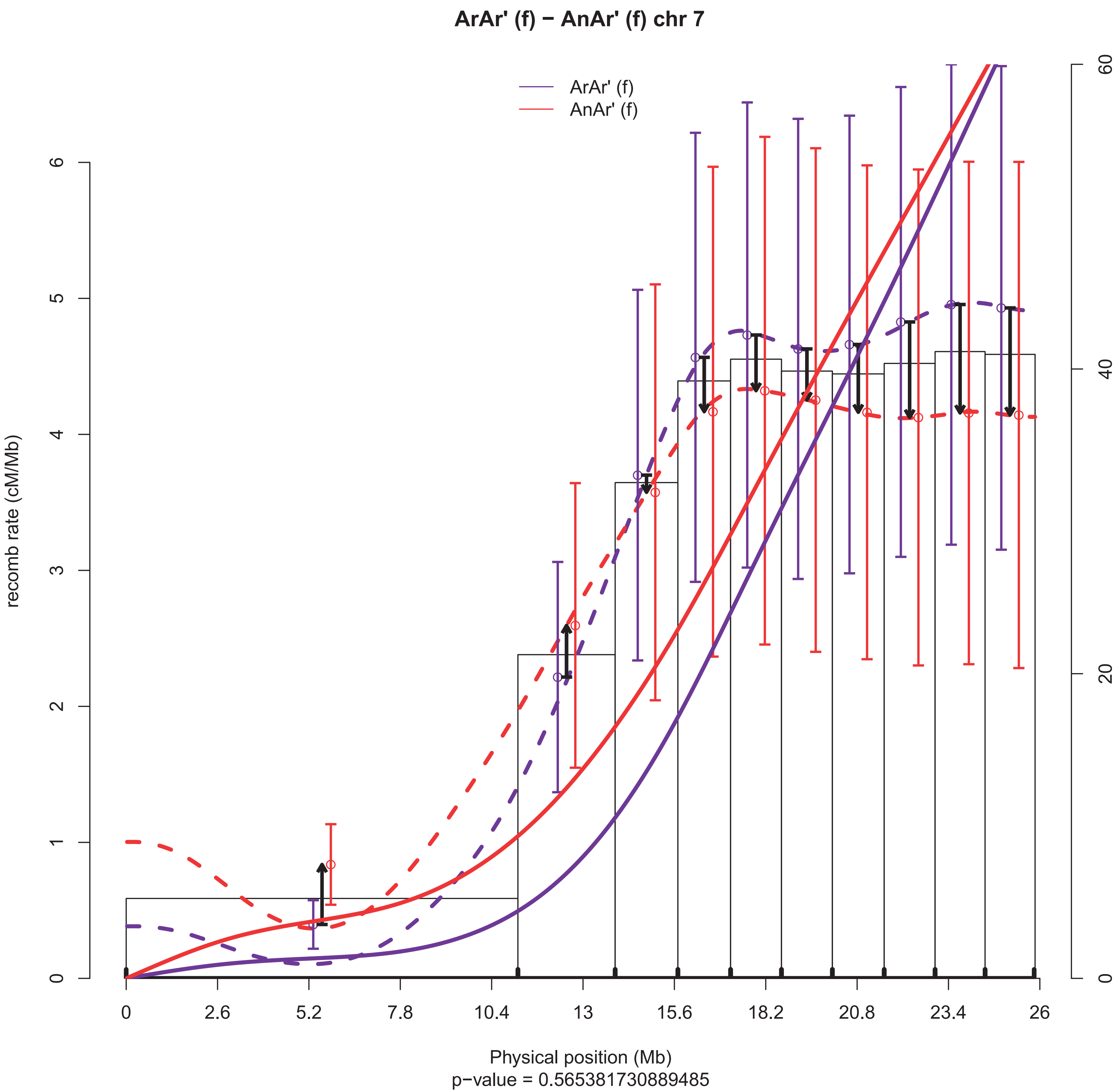

ArAr' (f) − AnAr' (f) chr 7
ArAr' (f)
AnAr' (f)
recomb rate (cM/Mb)
0
1
2
3
4
5
6
0
20
40
60
0
2.6
5.2
7.8
10.4
13
15.6
18.2
20.8
23.4
26
Physical position (Mb)
p−value = 0.565381730889485

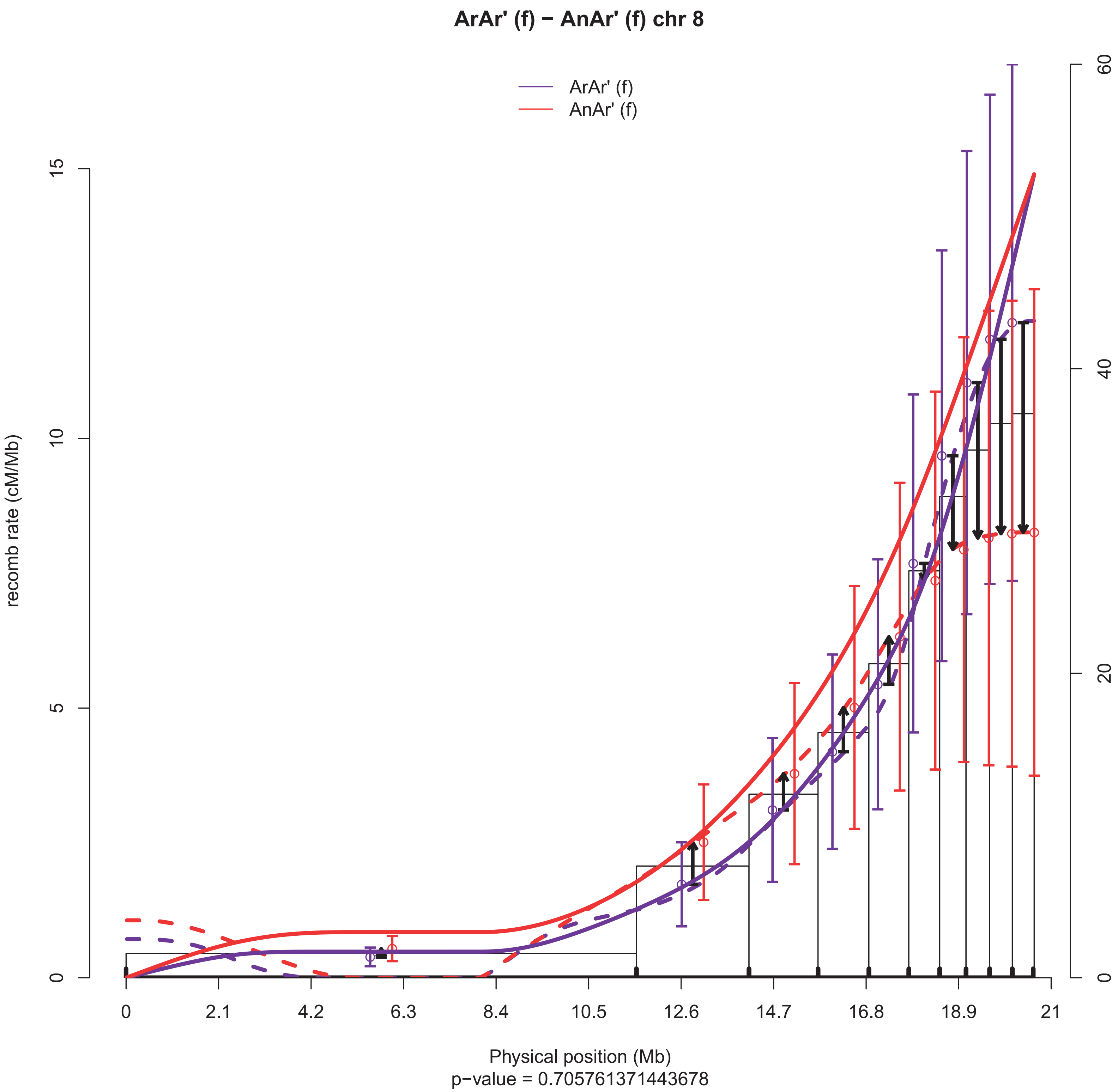
ArAr' (f) − AnAr' (f) chr 8
ArAr' (f)
AnAr' (f)
recomb rate (cM/Mb)
0
5
10
15
0
20
40
60
0
2.1
4.2
6.3
8.4
10.5
12.6
14.7
16.8
18.9
21
Physical position (Mb)
p−value = 0.705761371443678

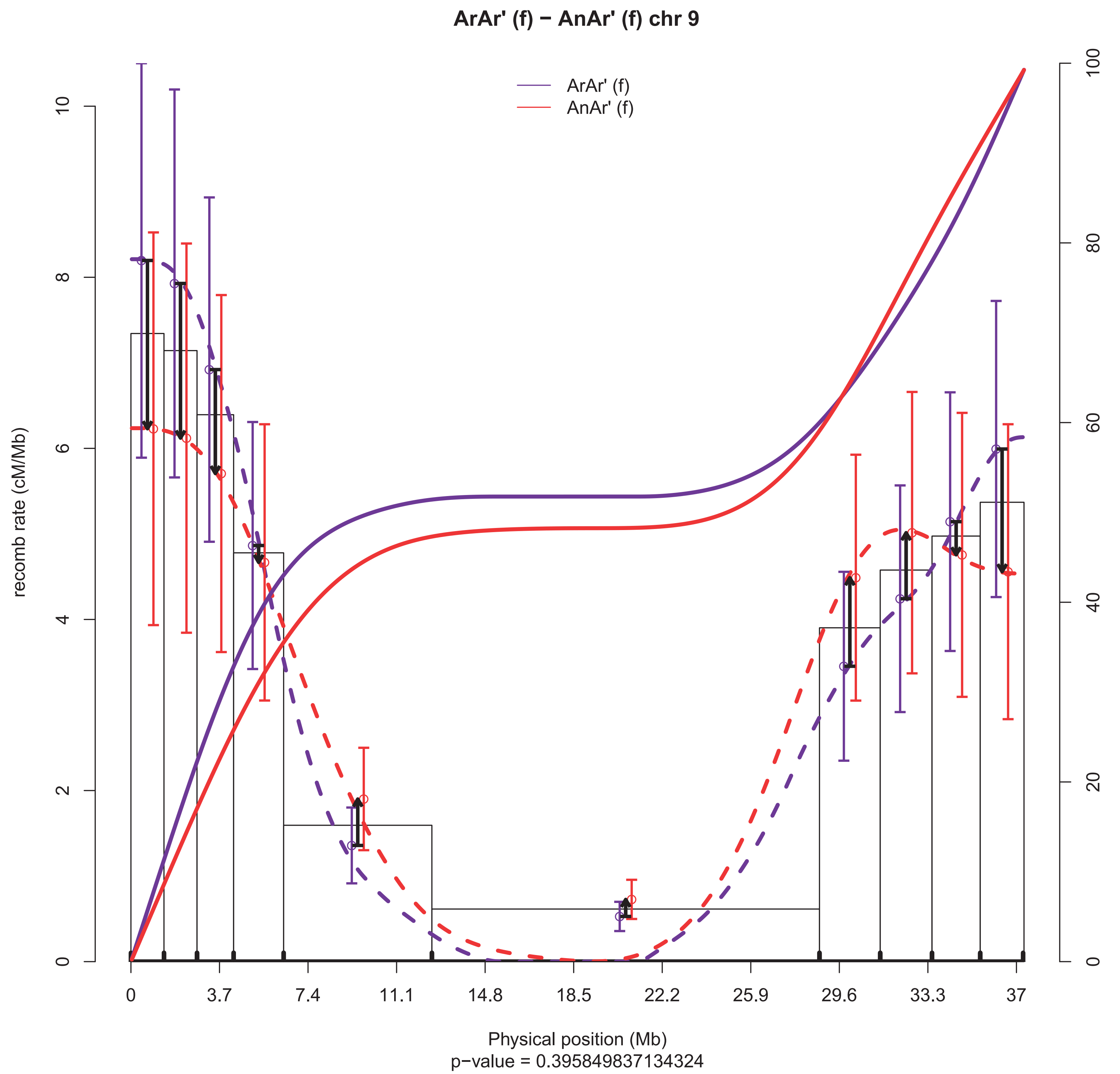

ArAr' (f) − AnAr' (f) chr 9
ArAr' (f)
AnAr' (f)
recomb rate (cM/Mb)
0
2
4
6
8
10
0
20
40
60
80
100
0
3.7
7.4
11.1
14.8
18.5
22.2
25.9
29.6
33.3
37
Physical position (Mb)
p−value = 0.395849837134324

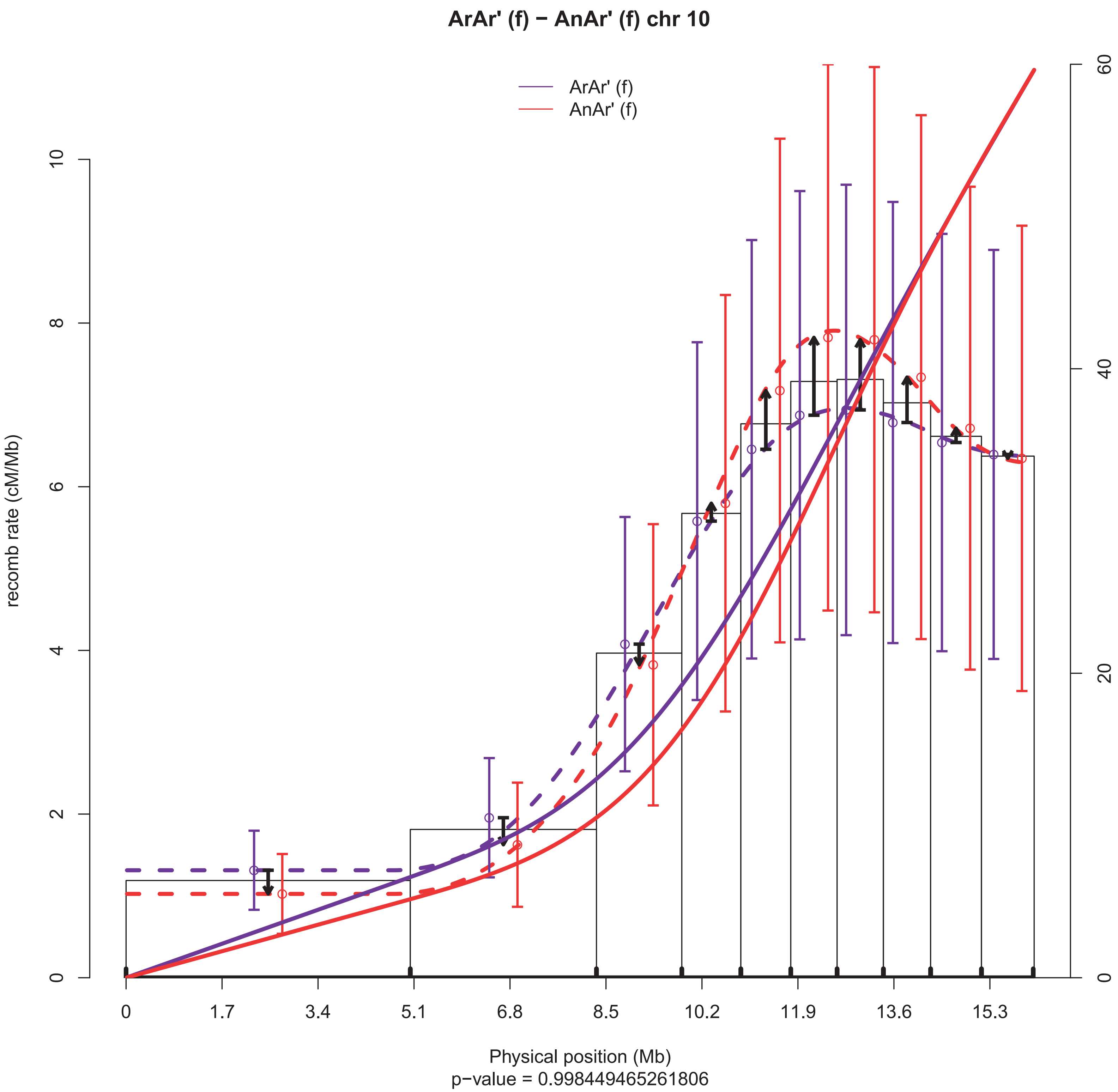
ArAr' (f) − AnAr' (f) chr 10
ArAr' (f)
AnAr' (f)
recomb rate (cM/Mb)
0
2
4
6
8
10
0
20
40
60
0
1.7
3.4
5.1
6.8
8.5
10.2
11.9
13.6
15.3
Physical position (Mb)
p−value = 0.998449465261806

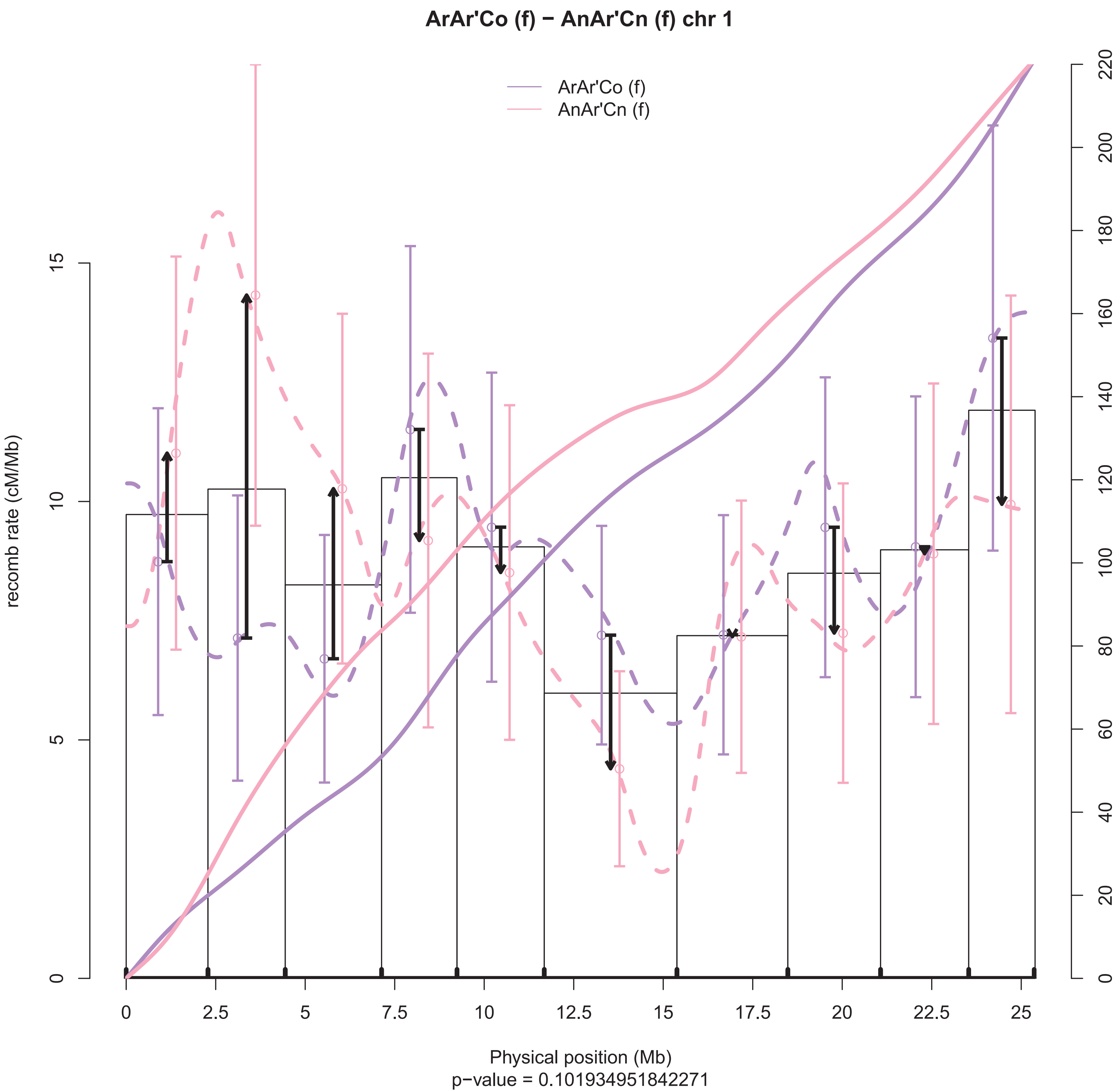

ArAr'Co (f) − AnAr'Cn (f) chr 1
ArAr'Co (f)
AnAr'Cn (f)
recomb rate (cM/Mb)
0
5
10
15
0
20
40
60
80
100
120
140
160
180
200
220
0
2.5
5
7.5
10
12.5
15
17.5
20
22.5
25
Physical position (Mb)
p−value = 0.101934951842271

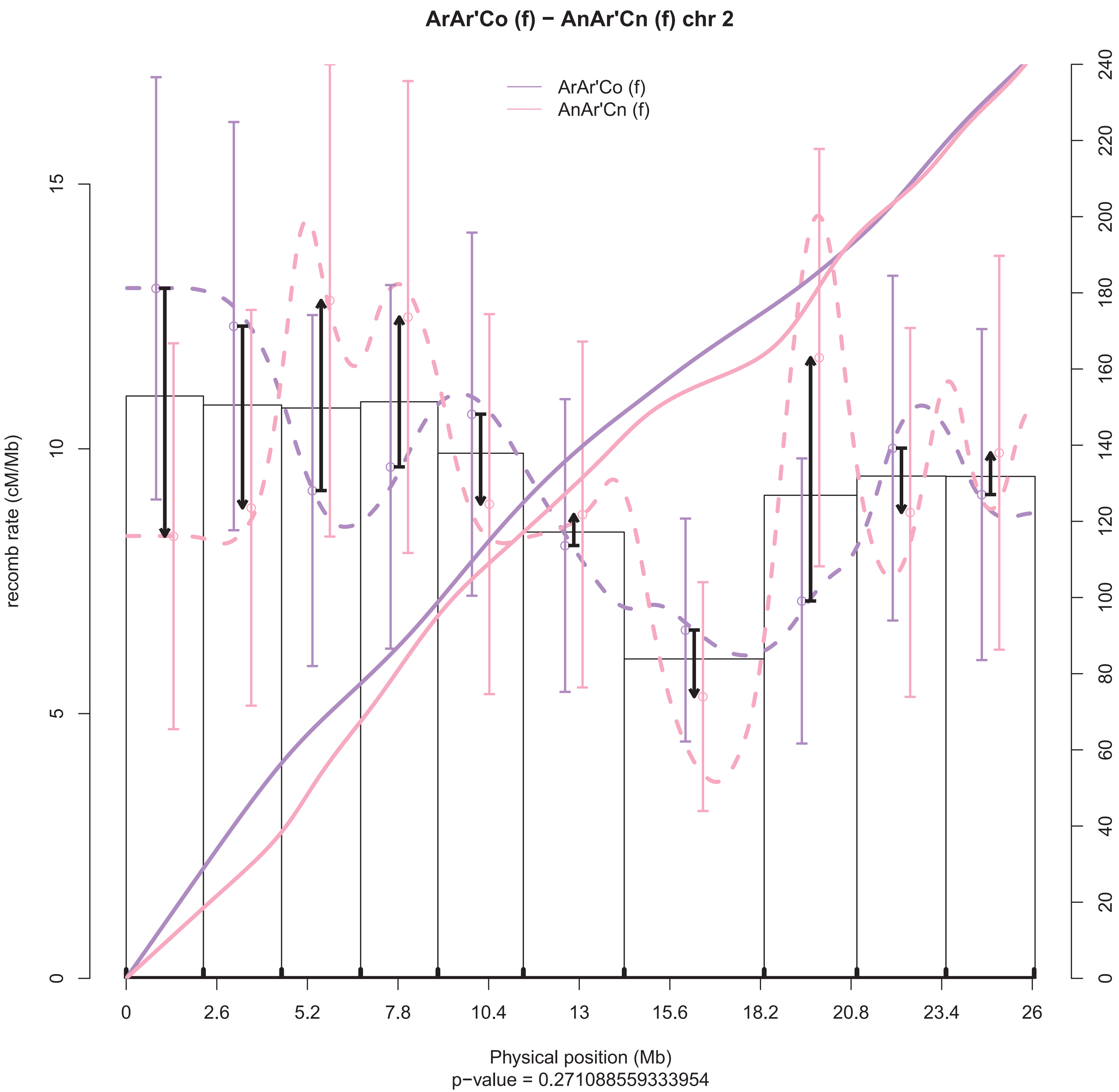

ArAr'Co (f) − AnAr'Cn (f) chr 2
ArAr'Co (f)
AnAr'Cn (f)
recomb rate (cM/Mb)
0
5
10
15
0
20
40
60
80
100
120
140
160
180
200
220
240
0
2.6
5.2
7.8
10.4
13
15.6
18.2
20.8
23.4
26
Physical position (Mb)
p−value = 0.271088559333954

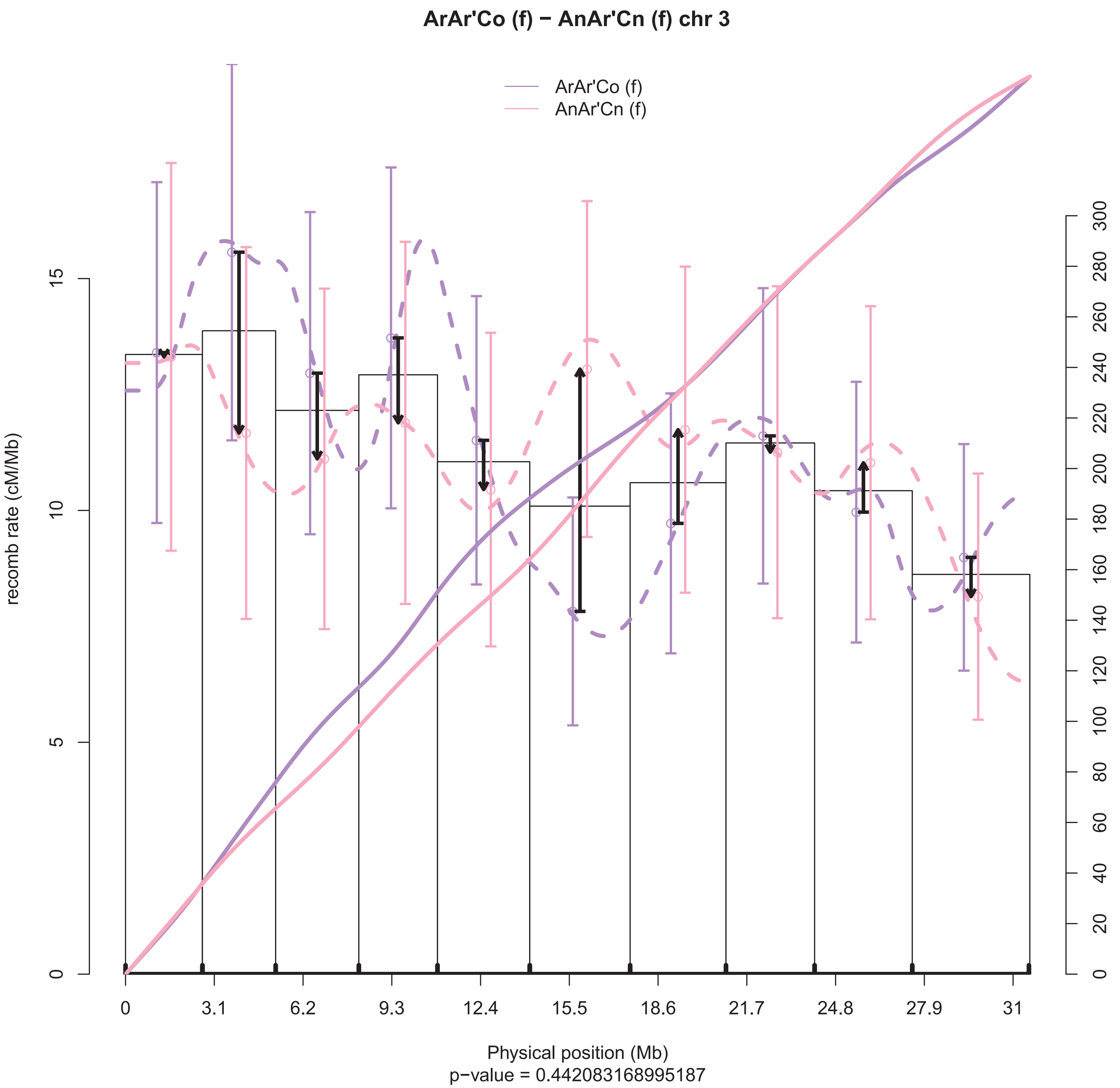
ArAr'Co (f) − AnAr'Cn (f) chr 3
ArAr'Co (f)
AnAr'Cn (f)
recomb rate (cM/Mb)
0
5
10
15
0
20
40
60
80
100
120
140
160
180
200
220
240
260
280
300
0
3.1
6.2
9.3
12.4
15.5
18.6
21.7
24.8
27.9
31
Physical position (Mb)
p−value = 0.442083168995187

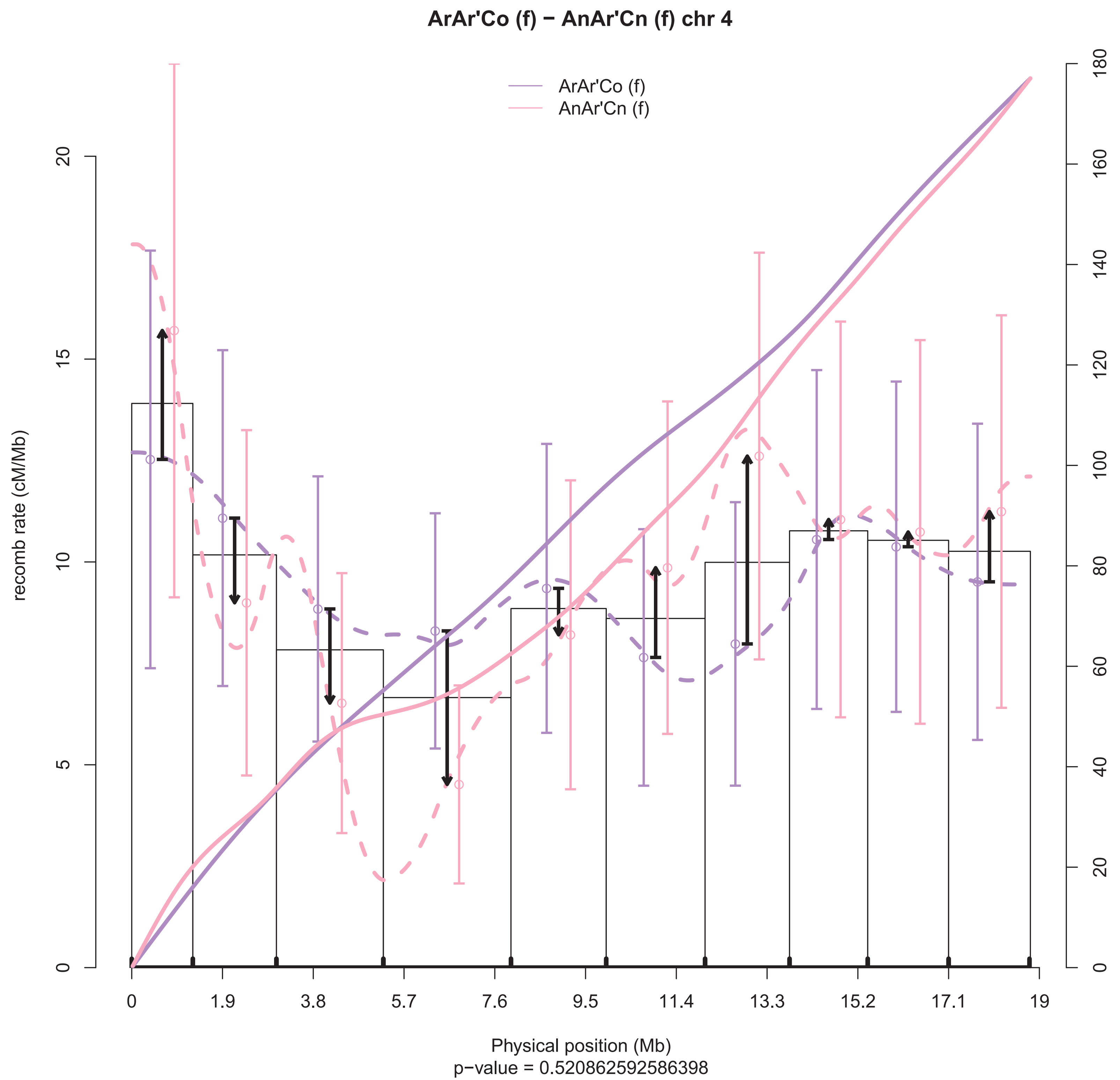
ArAr'Co (f) − AnAr'Cn (f) chr 4
ArAr'Co (f)
AnAr'Cn (f)
recomb rate (cM/Mb)
0
5
10
15
20
0
20
40
60
80
100
120
140
160
180
0
1.9
3.8
5.7
7.6
9.5
11.4
13.3
15.2
17.1
19
Physical position (Mb)
p−value = 0.520862592586398

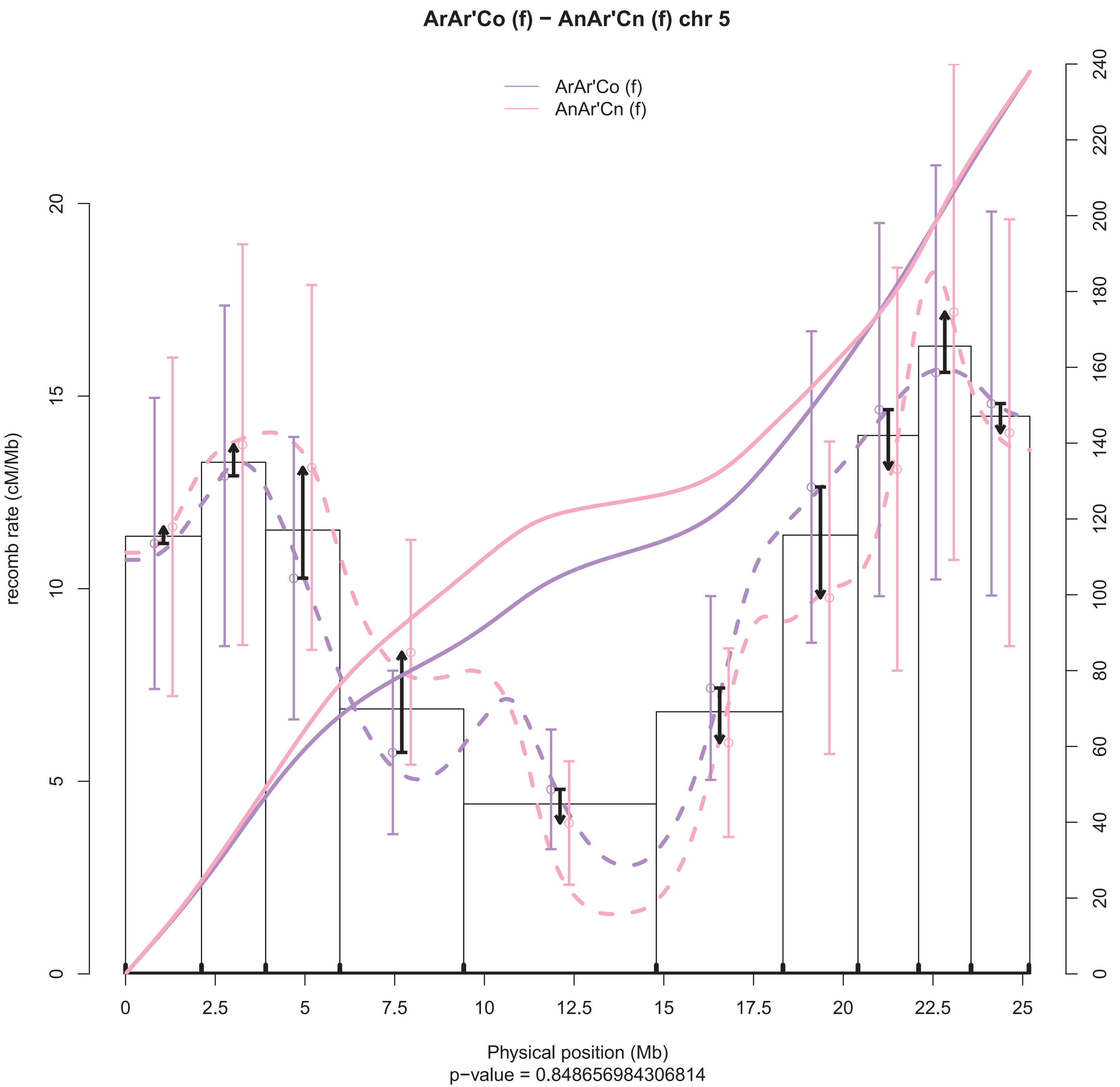

ArAr'Co (f) − AnAr'Cn (f) chr 5
ArAr'Co (f)
AnAr'Cn (f)
recomb rate (cM/Mb)
0
5
10
15
20
0
20
40
60
80
100
120
140
160
180
200
220
240
0
2.5
5
7.5
10
12.5
15
17.5
20
22.5
25
Physical position (Mb)
p−value = 0.848656984306814

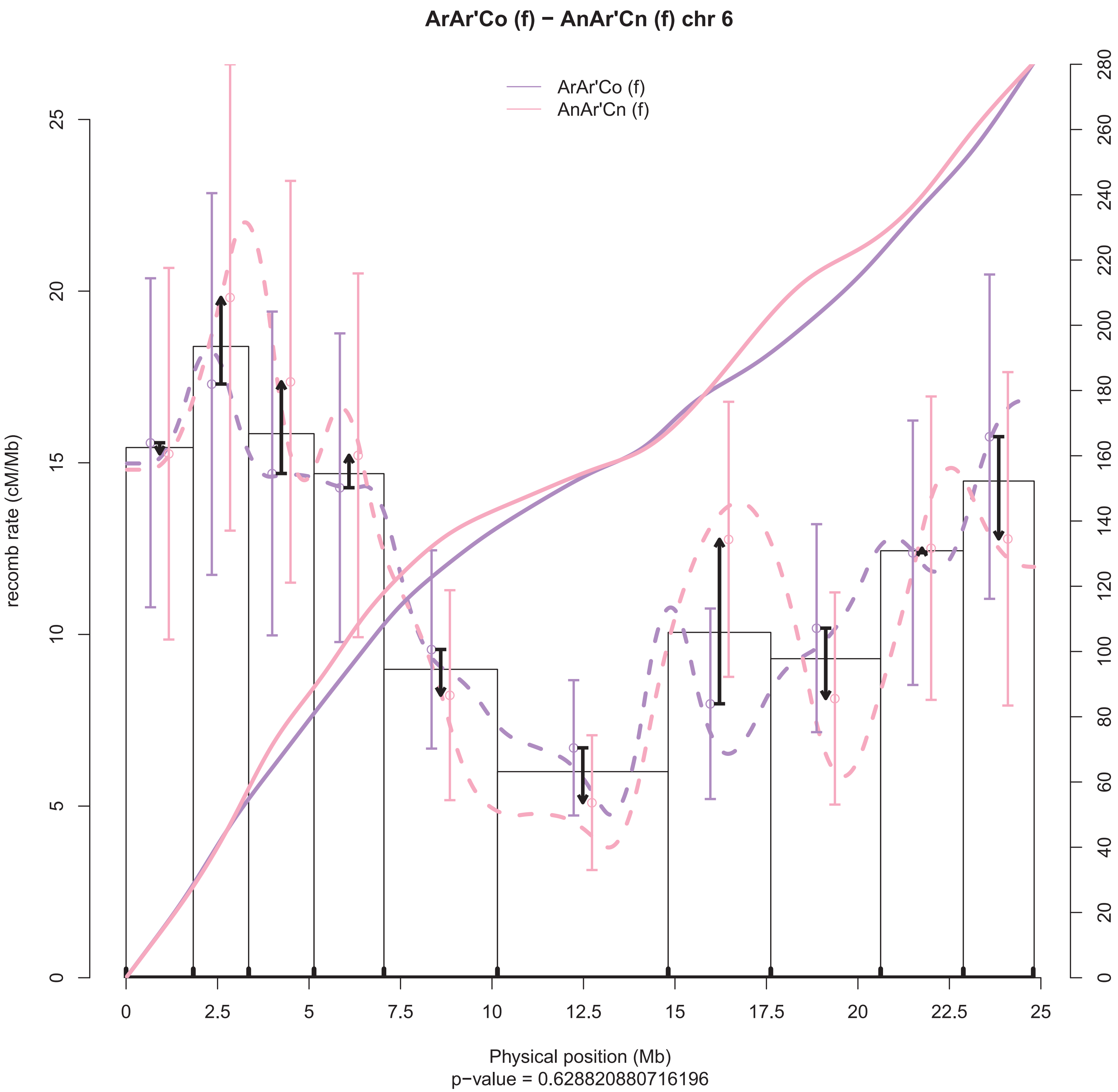

ArAr'Co (f) − AnAr'Cn (f) chr 6
ArAr'Co (f)
AnAr'Cn (f)
recomb rate (cM/Mb)
0
5
10
15
20
25
0
20
40
60
80
100
120
140
160
180
200
220
240
260
280
0
2.5
5
7.5
10
12.5
15
17.5
20
22.5
25
Physical position (Mb)
p−value = 0.628820880716196

ArAr'Co (f) − AnAr'Cn (f) chr 7

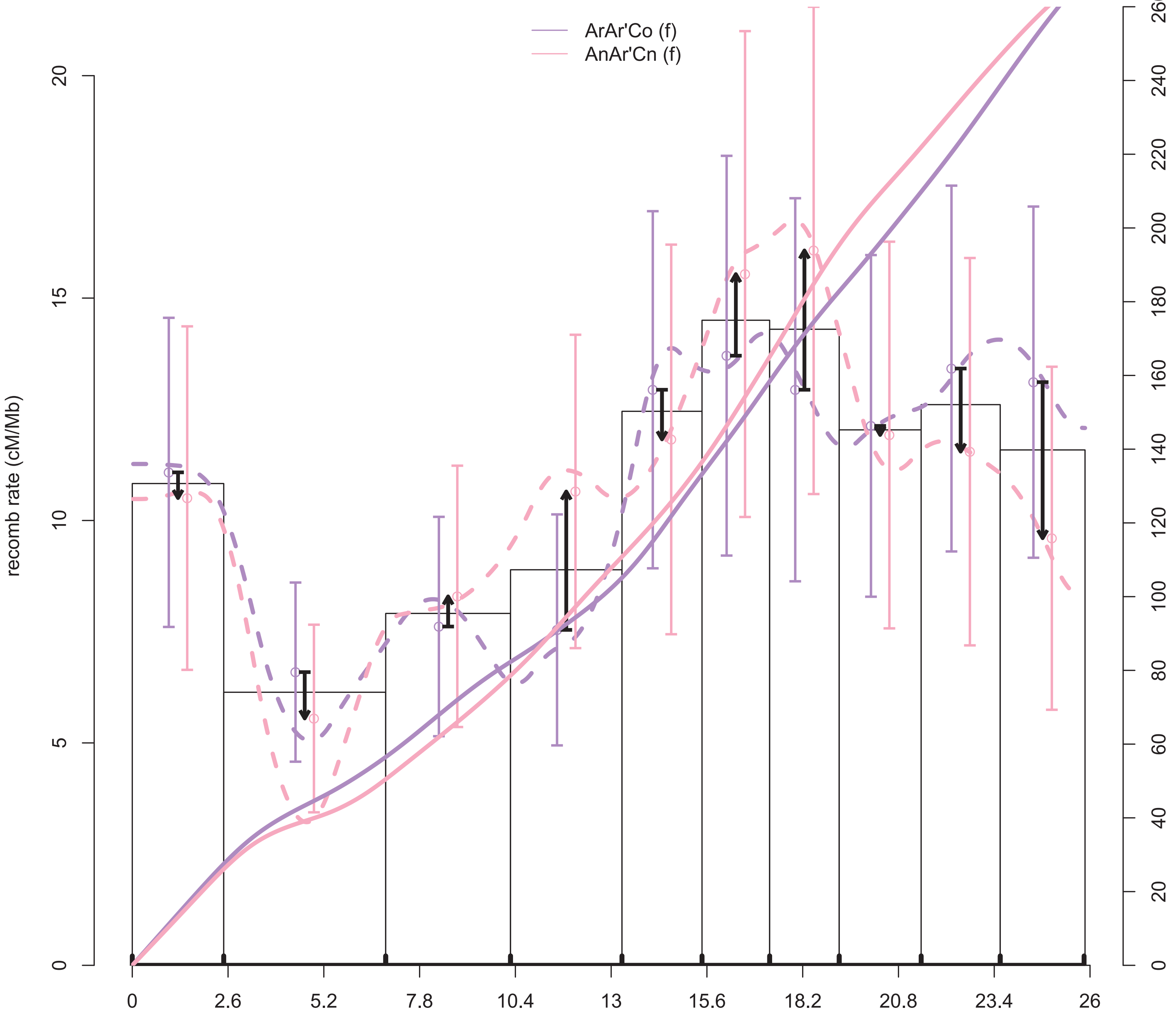

p−value = 0.836481682810341

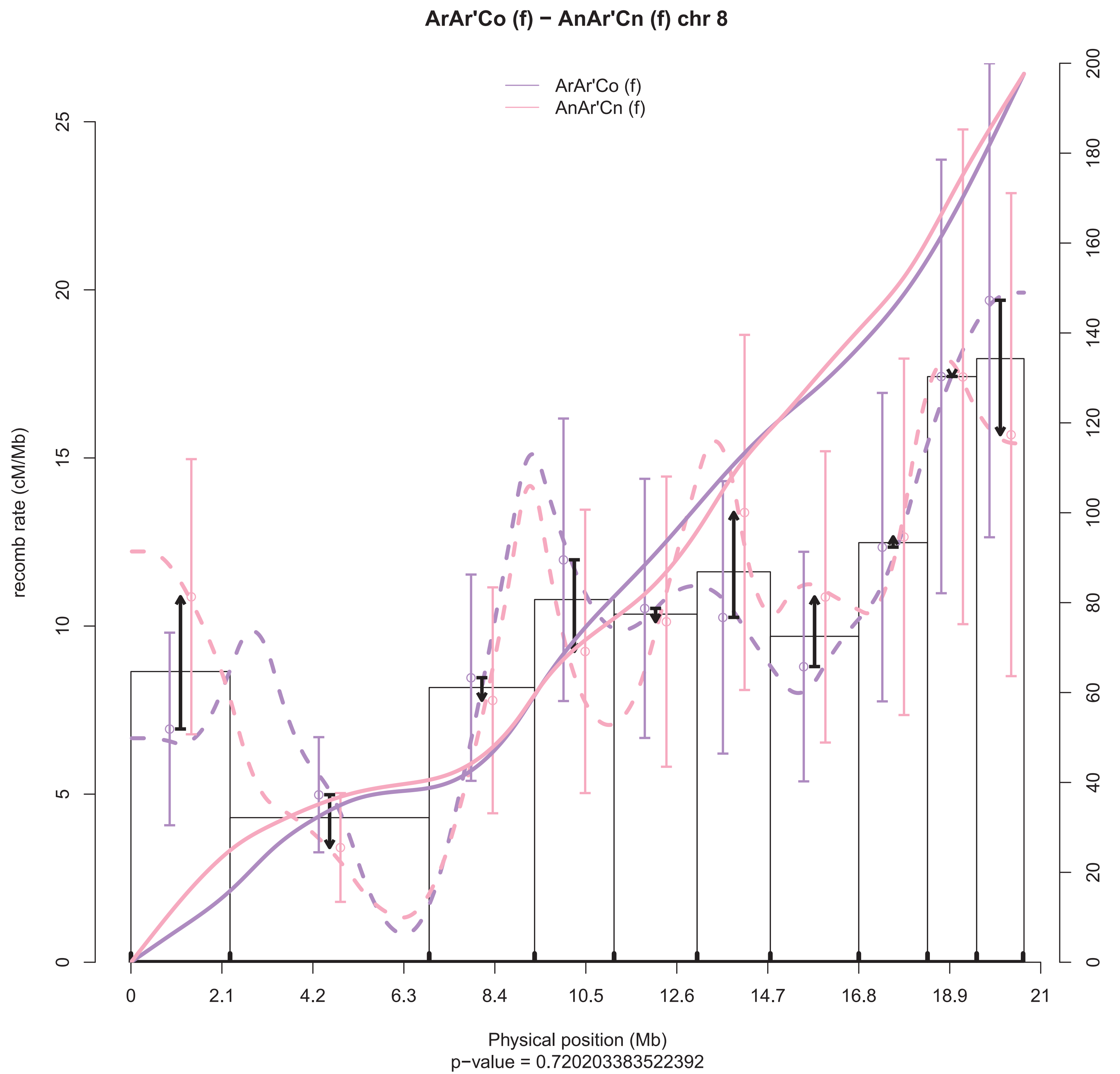
ArAr'Co (f) − AnAr'Cn (f) chr 8
ArAr'Co (f)
AnAr'Cn (f)
recomb rate (cM/Mb)
0
5
10
15
20
25
0
20
40
60
80
100
120
140
160
180
200
0
2.1
4.2
6.3
8.4
10.5
12.6
14.7
16.8
18.9
21
Physical position (Mb)
p−value = 0.720203383522392

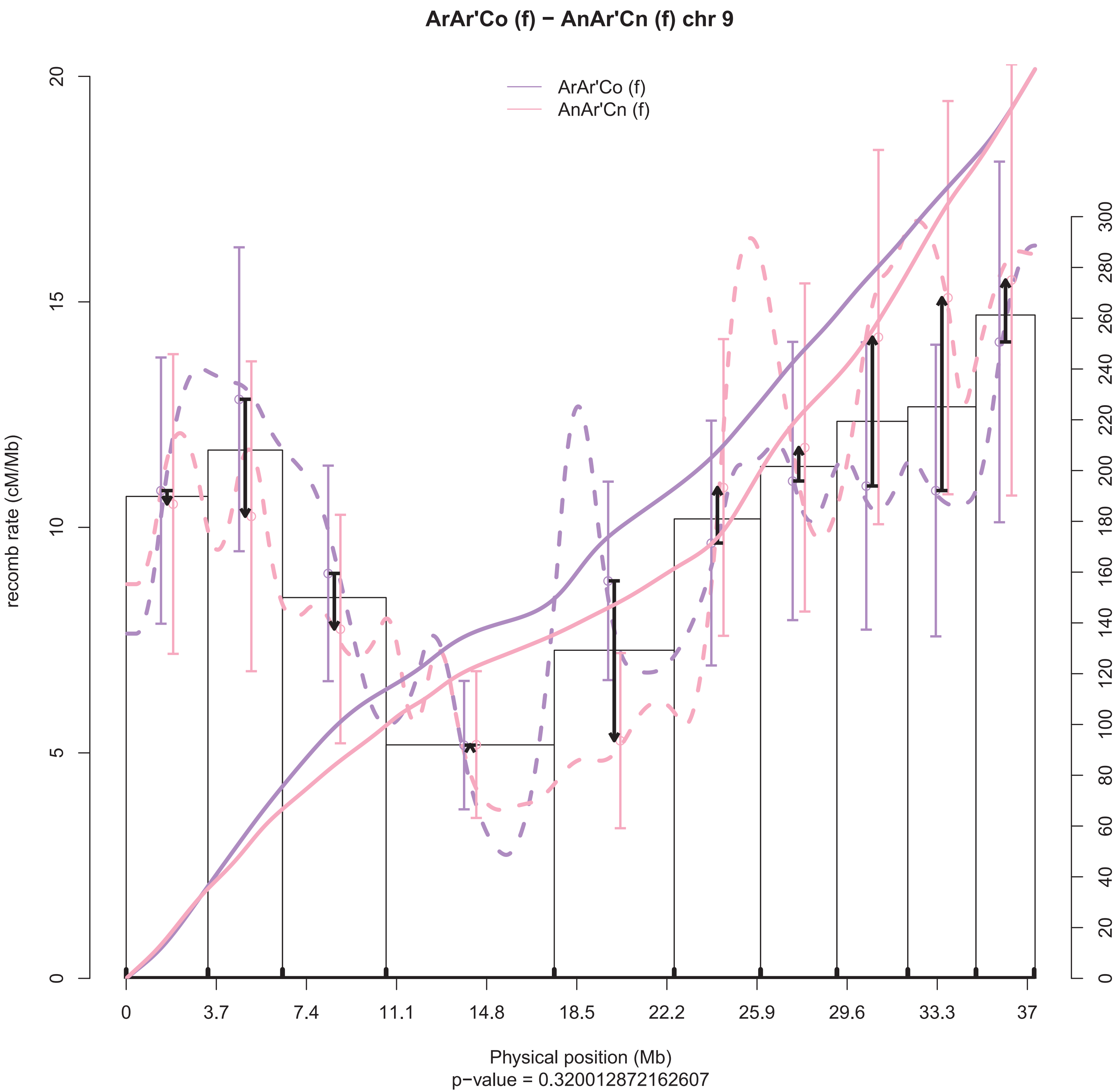

ArAr'Co (f) − AnAr'Cn (f) chr 9
ArAr'Co (f)
AnAr'Cn (f)
recomb rate (cM/Mb)
0
5
10
15
20
0
20
40
60
80
100
120
140
160
180
200
220
240
260
280
300
0
3.7
7.4
11.1
14.8
18.5
22.2
25.9
29.6
33.3
37
Physical position (Mb)
p−value = 0.320012872162607

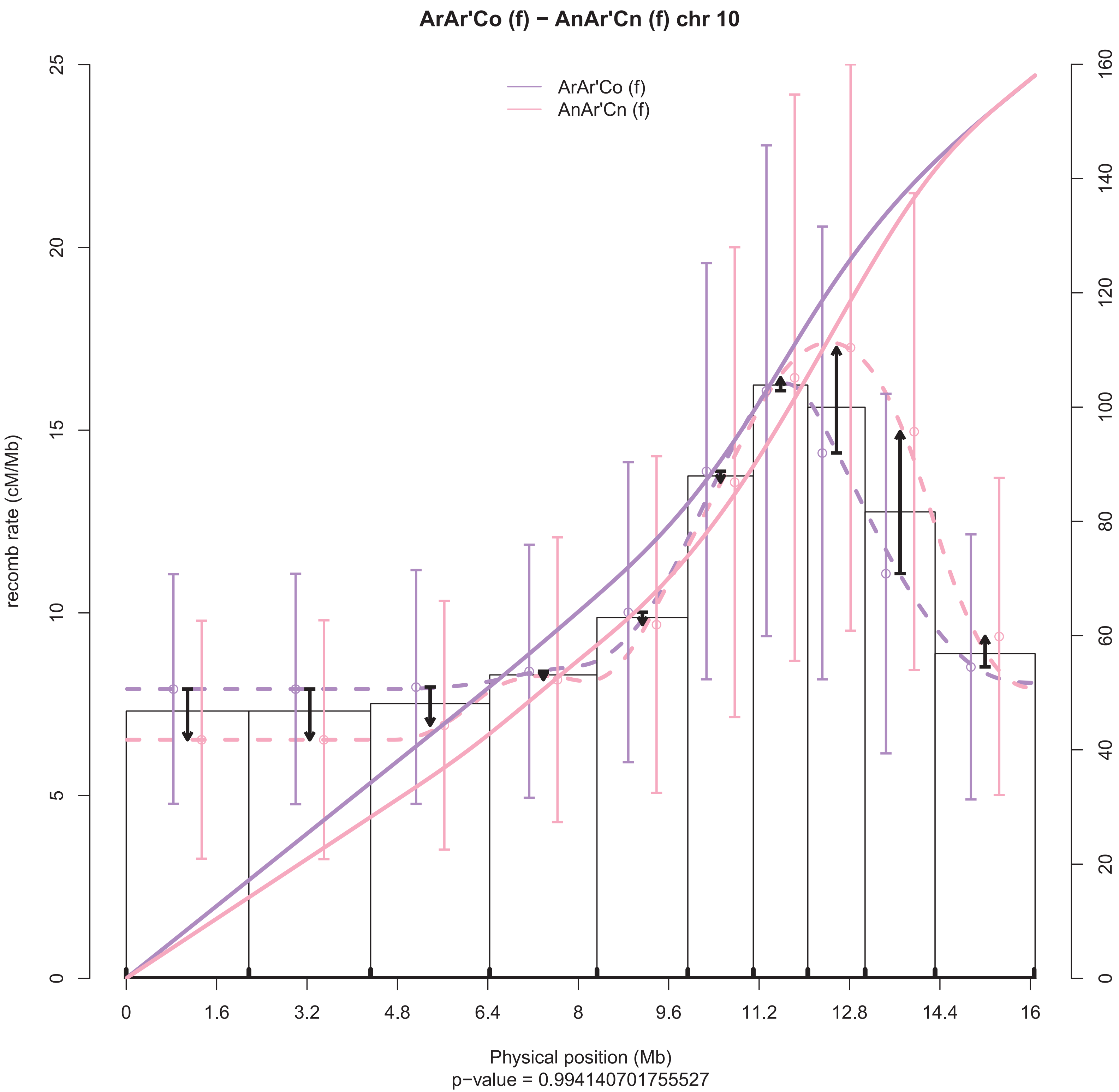

ArAr'Co (f) − AnAr'Cn (f) chr 10
ArAr'Co (f)
AnAr'Cn (f)
recomb rate (cM/Mb)
0
5
10
15
20
25
0
20
40
60
80
100
120
140
160
0
1.6
3.2
4.8
6.4
8
9.6
11.2
12.8
14.4
16
Physical position (Mb)
p−value = 0.994140701755527
