# Supplementary material for: Amplifying recombination genome-wide and reshaping crossover landscapes in Brassicas
Source: PLoS Genet. 2017 May 11;13(5):e1006794. doi: 10.1371/journal.pgen.1006794 (PMC5444851; doi:10.1371/journal.pgen.1006794)
Supplement: S10 Fig — The p-values and R2 are indicated in S7 Table. (PDF) [file pgen.1006794.s010.pdf]

ArAr' (f)

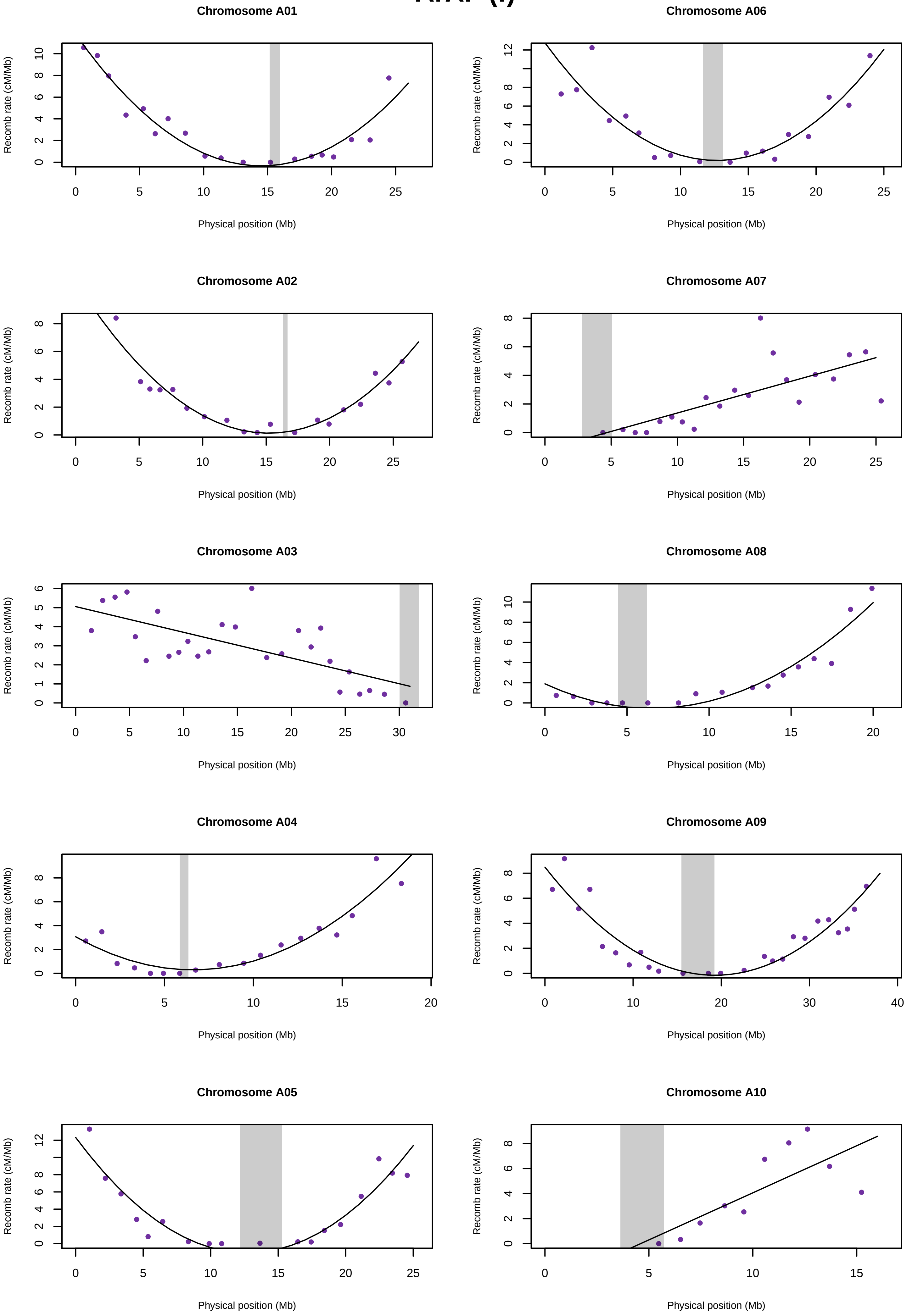
Chromosome A01
Chromosome A02
Chromosome A03
Chromosome A04
Chromosome A05
Chromosome A06
Chromosome A07
Chromosome A08
Chromosome A09
Chromosome A10
Recomb rate (cM/Mb)
Physical position (Mb)

ArAr'Co (f)

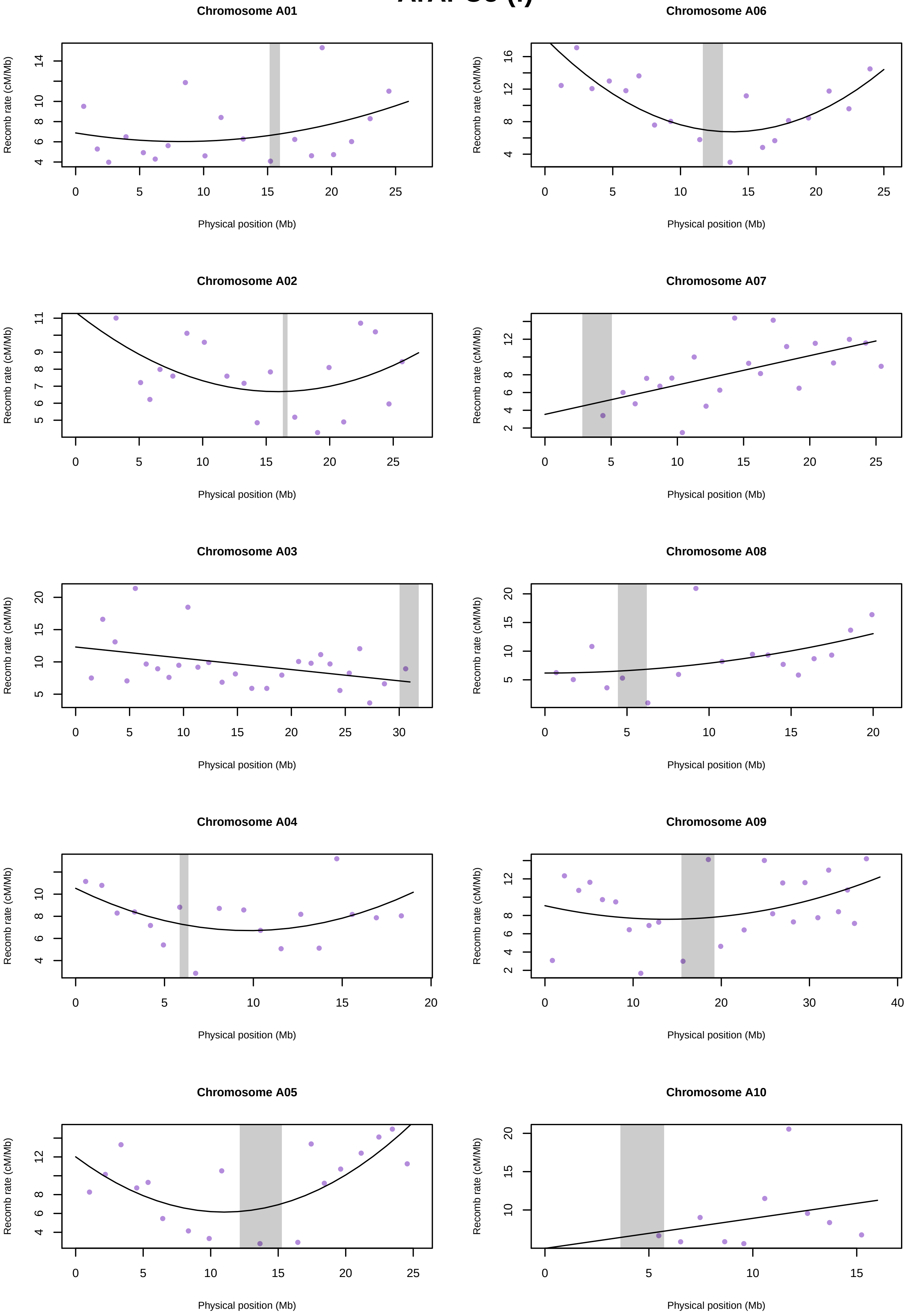
Chromosome A01
Recomb rate (cM/Mb)
Physical position (Mb)
Chromosome A06
Recomb rate (cM/Mb)
Physical position (Mb)
Chromosome A02
Recomb rate (cM/Mb)
Physical position (Mb)
Chromosome A07
Recomb rate (cM/Mb)
Physical position (Mb)
Chromosome A03
Recomb rate (cM/Mb)
Physical position (Mb)
Chromosome A08
Recomb rate (cM/Mb)
Physical position (Mb)
Chromosome A04
Recomb rate (cM/Mb)
Physical position (Mb)
Chromosome A09
Recomb rate (cM/Mb)
Physical position (Mb)
Chromosome A05
Recomb rate (cM/Mb)
Physical position (Mb)
Chromosome A10
Recomb rate (cM/Mb)
Physical position (Mb)

AnAr' (f)

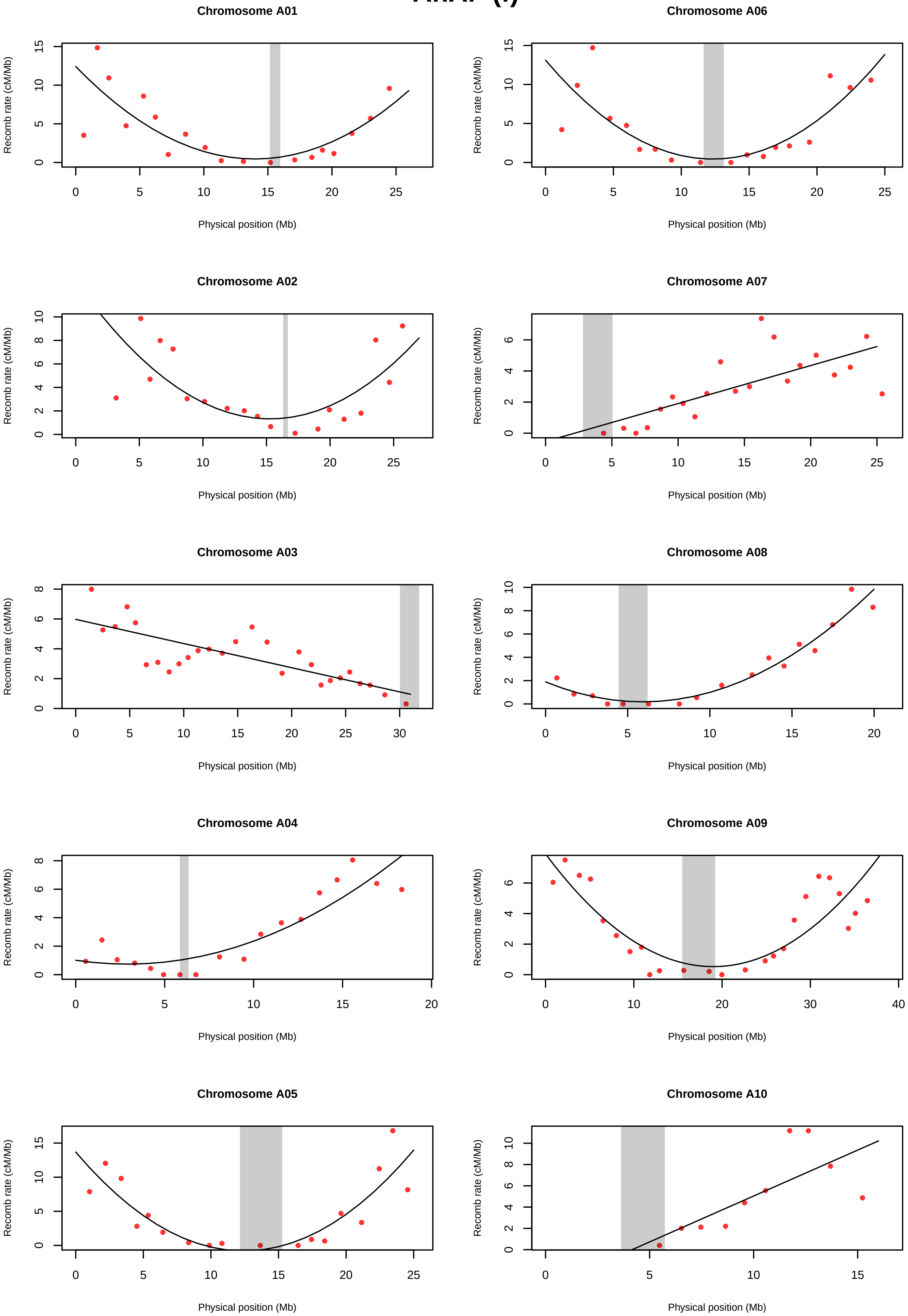
Chromosome A01
Chromosome A06
Chromosome A02
Chromosome A07
Chromosome A03
Chromosome A08
Chromosome A04
Chromosome A09
Chromosome A05
Chromosome A10
Recomb rate (cM/Mb)
Physical position (Mb)

AnAr'Cn (f)

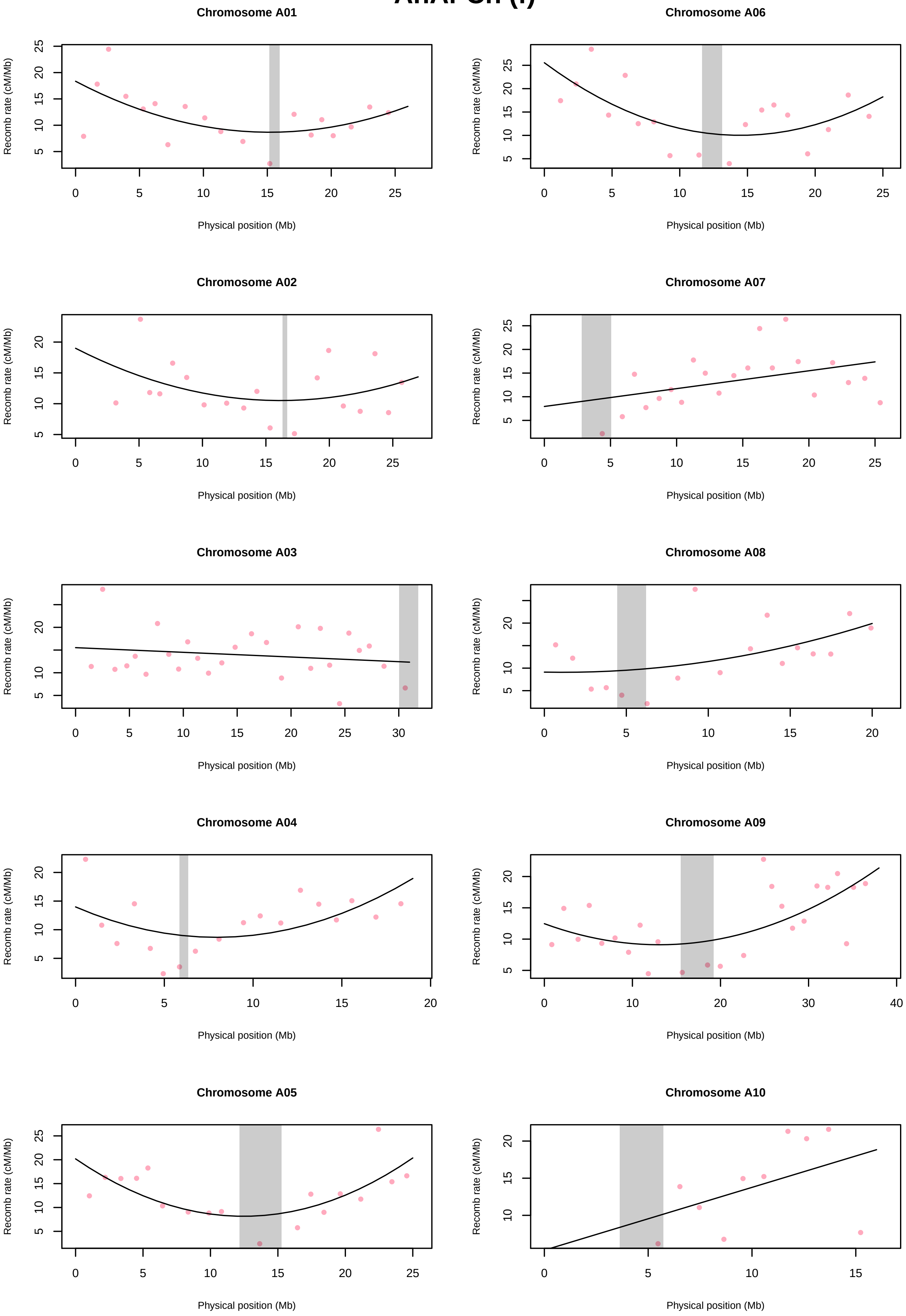
Chromosome A01
Chromosome A06
Chromosome A02
Chromosome A07
Chromosome A03
Chromosome A08
Chromosome A04
Chromosome A09
Chromosome A05
Chromosome A10
Recomb rate (cM/Mb)
Physical position (Mb)

AnAr' (m)

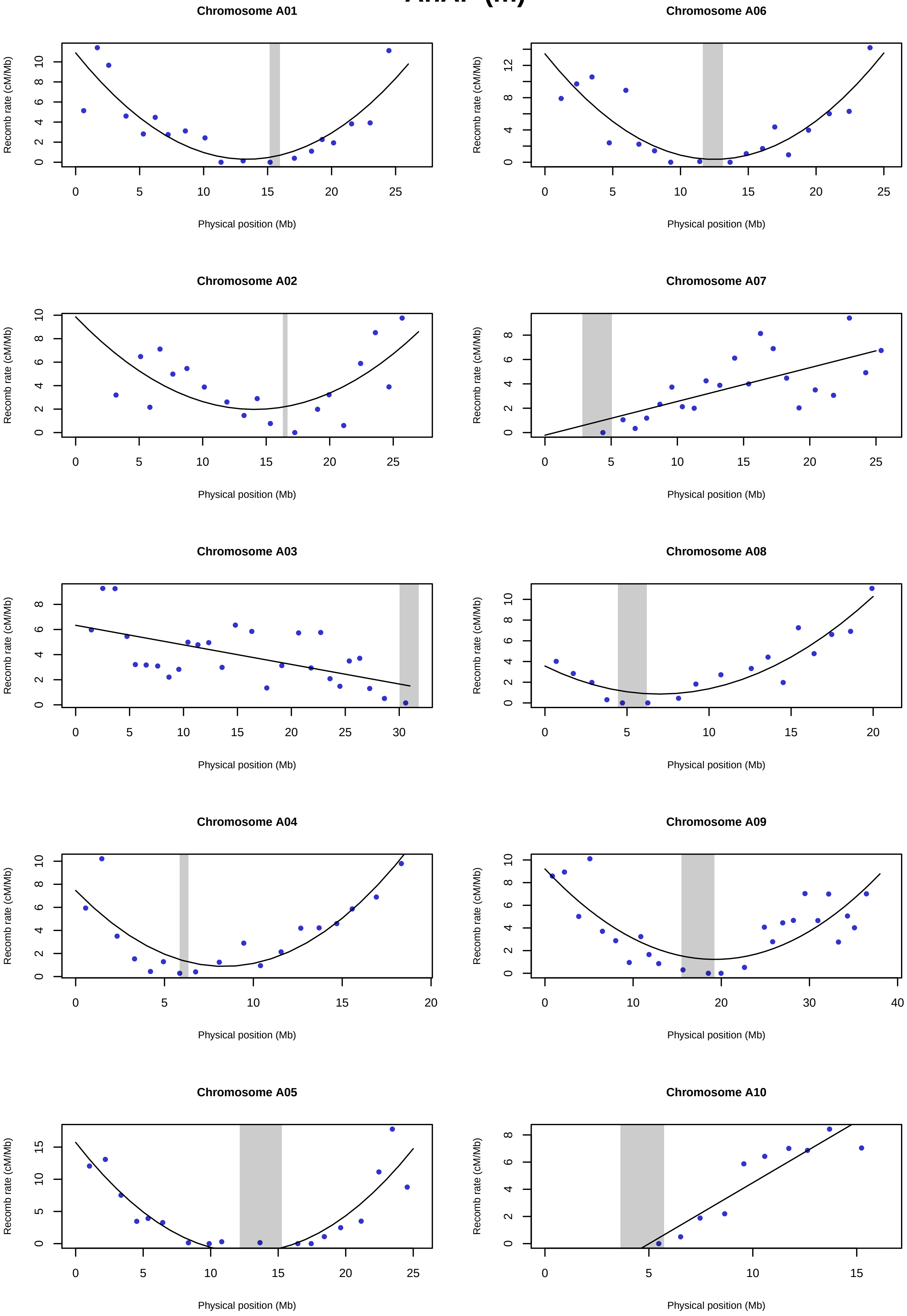
Chromosome A01
Chromosome A06
Chromosome A02
Chromosome A07
Chromosome A03
Chromosome A08
Chromosome A04
Chromosome A09
Chromosome A05
Chromosome A10
Recomb rate (cM/Mb)
Physical position (Mb)

AnAr'Cn (m)

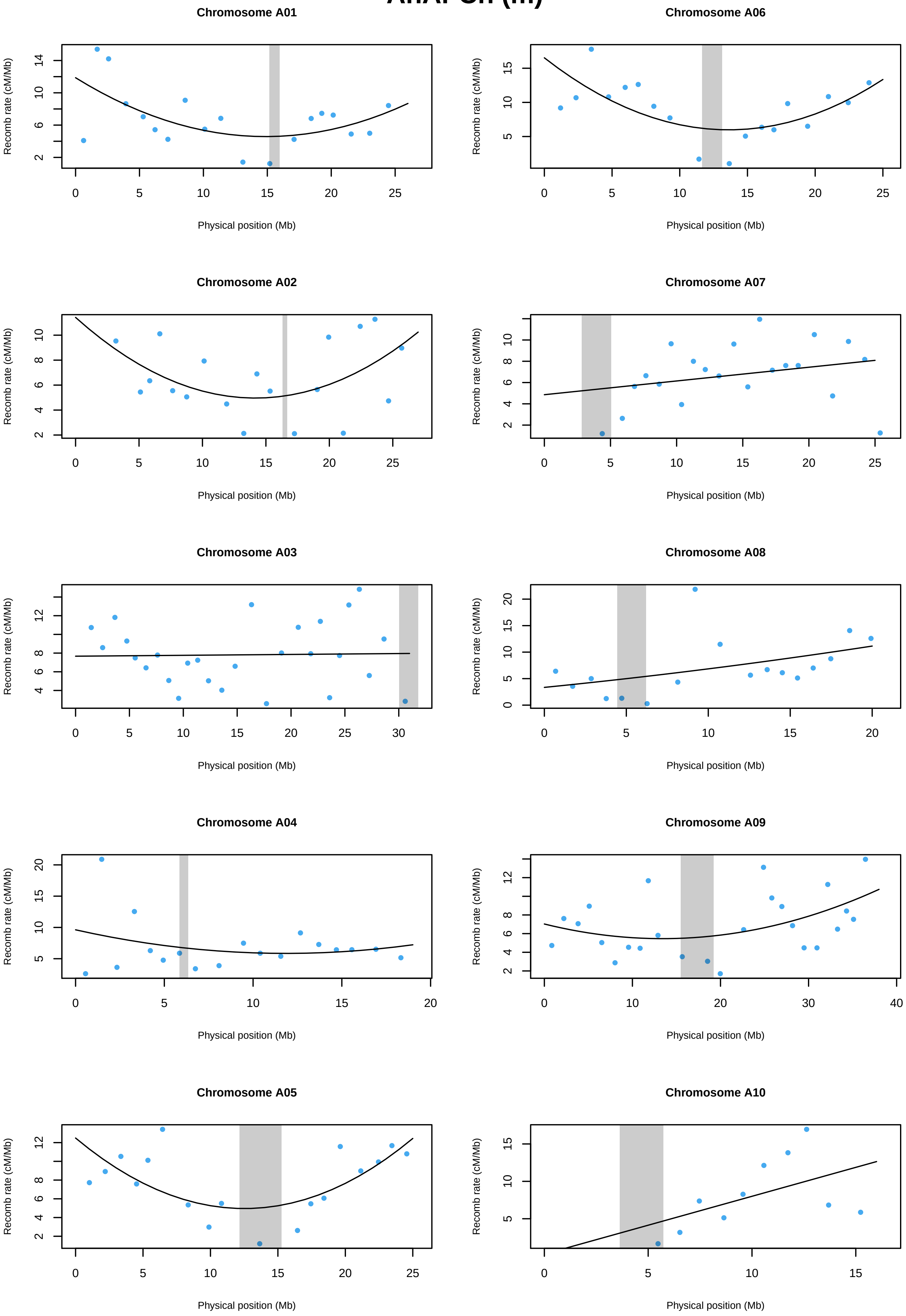
Chromosome A01
Chromosome A06
Chromosome A02
Chromosome A07
Chromosome A03
Chromosome A08
Chromosome A04
Chromosome A09
Chromosome A05
Chromosome A10
Recomb rate (cM/Mb)
Physical position (Mb)
