# Supplementary material for: Amplifying recombination genome-wide and reshaping crossover landscapes in Brassicas
Source: PLoS Genet. 2017 May 11;13(5):e1006794. doi: 10.1371/journal.pgen.1006794 (PMC5444851; doi:10.1371/journal.pgen.1006794)
Supplement: S11 Fig — Comparison of the distribution of genetic distances between successive COs from populations deriving of females ArAr’ (in purple) vs ArAr’Co (in light purple), females AnAr’ (in red) vs AnAr’Cn (in pink) and males AnAr’ (in blue) vs AnAr’Cn (in light blue). X-axis: genetic distance between successive COs. Solid lines correspond to experimental data. Dashed lines indicate the corresponding distributions in the "no-interference" situation, obtained by re-shuffling CO positions of experimental data (see Methods). For each population, the Küllback-Leibler divergence (KL Div.) from the experimental to the "no-interference" distribution provides a quantitative measurement of interference strength. p-value: one-sided p-value of the HO hypothesis that the diploids and triploids have the same the KL Div. index (and thus interference strength for the considered chromosome). Sufficiently small values indicate significantly higher interference in diploids than in allotriploids (see details in Methods). (PDF) [file pgen.1006794.s011.pdf]

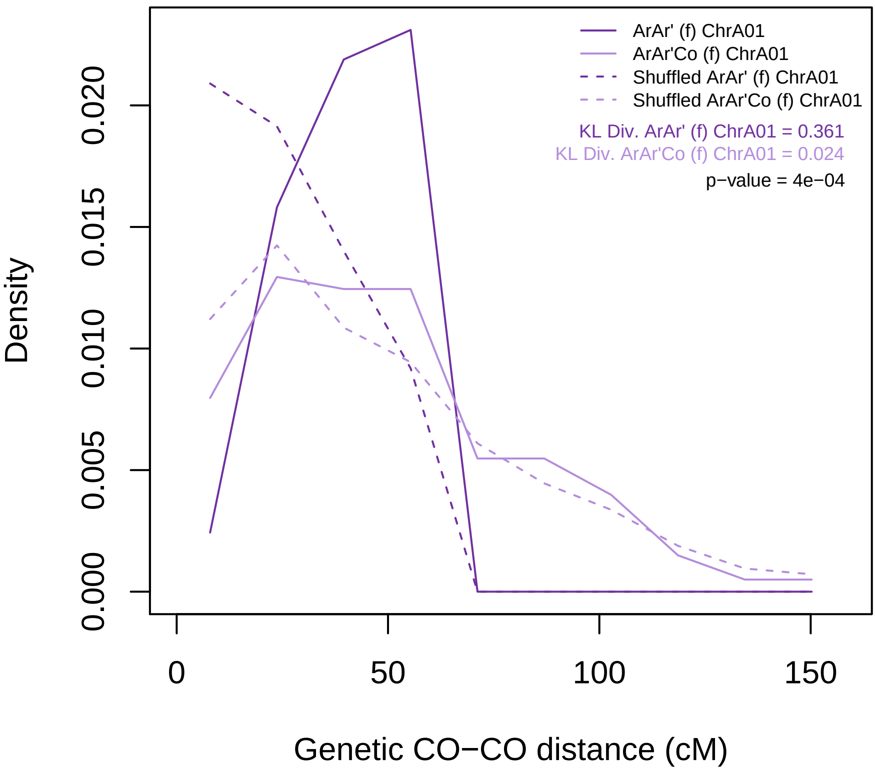
ArAr' (f) ChrA01
ArAr'Co (f) ChrA01
Shuffled ArAr' (f) ChrA01
Shuffled ArAr'Co (f) ChrA01
KL Div. ArAr' (f) ChrA01 = 0.361
KL Div. ArAr'Co (f) ChrA01 = 0.024
p−value = 4e−04
Density
0.000
0.005
0.010
0.015
0.020
0
50
100
150
Genetic CO−CO distance (cM)

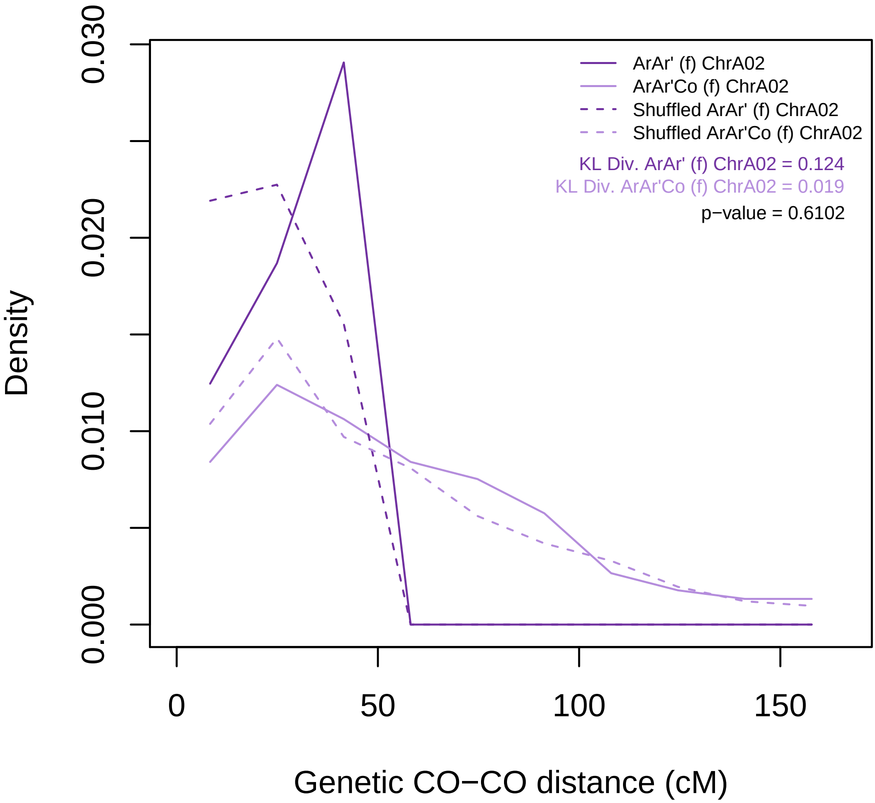

ArAr' (f) ChrA02
ArAr'Co (f) ChrA02
Shuffled ArAr' (f) ChrA02
Shuffled ArAr'Co (f) ChrA02
KL Div. ArAr' (f) ChrA02 = 0.124
KL Div. ArAr'Co (f) ChrA02 = 0.019
p−value = 0.6102
Density
0.000
0.010
0.020
0.030
0
50
100
150
Genetic CO−CO distance (cM)

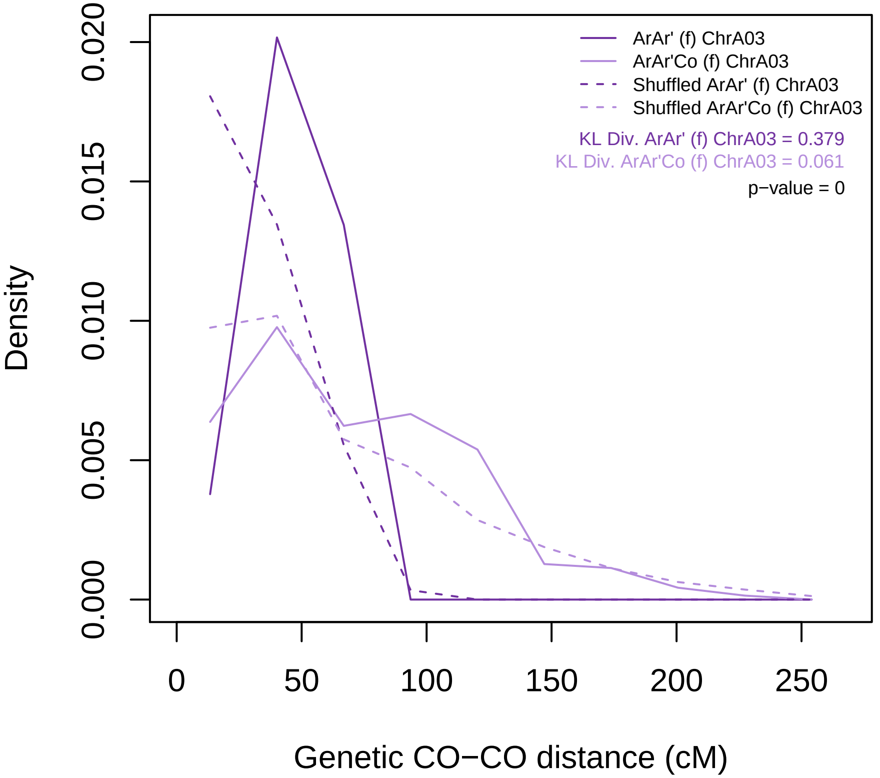
ArAr' (f) ChrA03
ArAr'Co (f) ChrA03
Shuffled ArAr' (f) ChrA03
Shuffled ArAr'Co (f) ChrA03
KL Div. ArAr' (f) ChrA03 = 0.379
KL Div. ArAr'Co (f) ChrA03 = 0.061
p−value = 0
Density
0.000
0.005
0.010
0.015
0.020
0
50
100
150
200
250
Genetic CO−CO distance (cM)

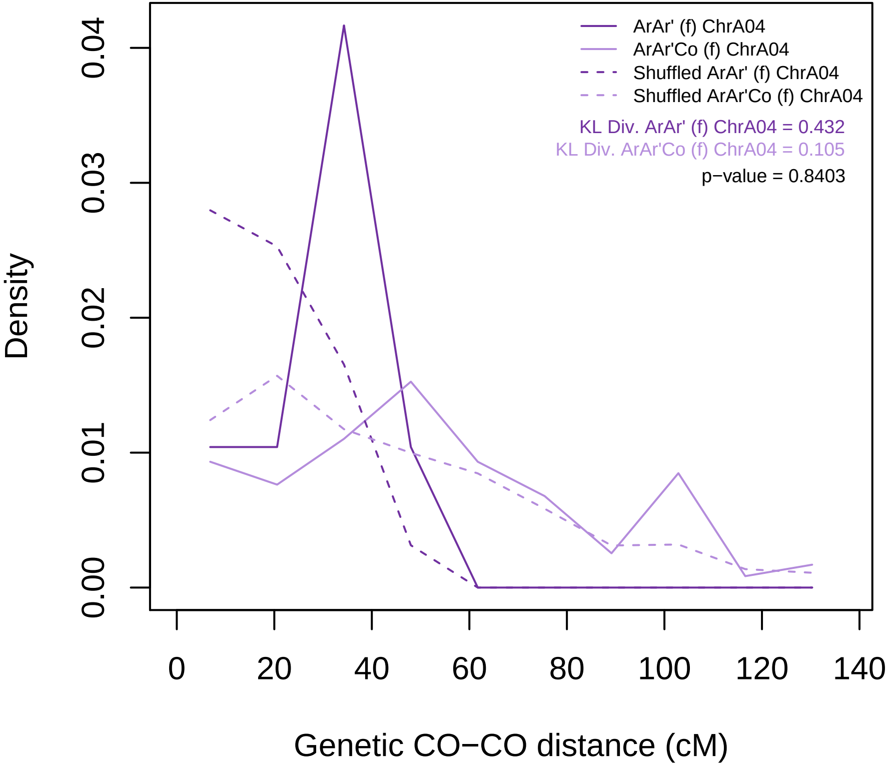
ArAr' (f) ChrA04
ArAr'Co (f) ChrA04
Shuffled ArAr' (f) ChrA04
Shuffled ArAr'Co (f) ChrA04
KL Div. ArAr' (f) ChrA04 = 0.432
KL Div. ArAr'Co (f) ChrA04 = 0.105
p−value = 0.8403
Density
0.00
0.01
0.02
0.03
0.04
0
20
40
60
80
100
120
140
Genetic CO−CO distance (cM)

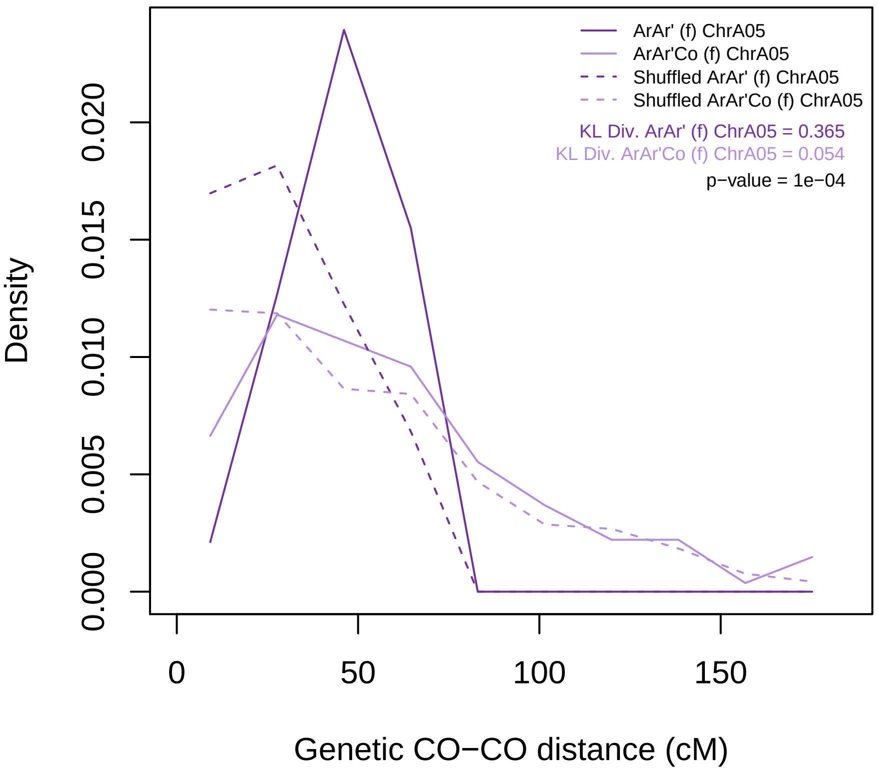

ArAr' (f) ChrA05
ArAr'Co (f) ChrA05
Shuffled ArAr' (f) ChrA05
Shuffled ArAr'Co (f) ChrA05
KL Div. ArAr' (f) ChrA05 = 0.365
KL Div. ArAr'Co (f) ChrA05 = 0.054
p−value = 1e−04
Density
0.000
0.005
0.010
0.015
0.020
0
50
100
150
Genetic CO−CO distance (cM)

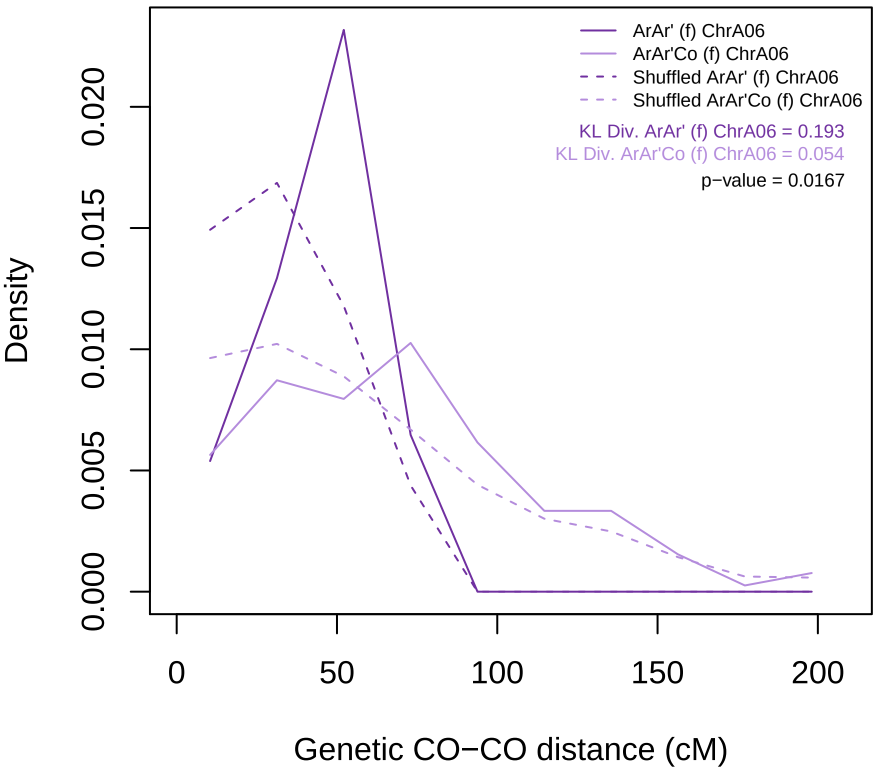
ArAr' (f) ChrA06
ArAr'Co (f) ChrA06
Shuffled ArAr' (f) ChrA06
Shuffled ArAr'Co (f) ChrA06
KL Div. ArAr' (f) ChrA06 = 0.193
KL Div. ArAr'Co (f) ChrA06 = 0.054
p−value = 0.0167
Density
0.000
0.005
0.010
0.015
0.020
0
50
100
150
200
Genetic CO−CO distance (cM)

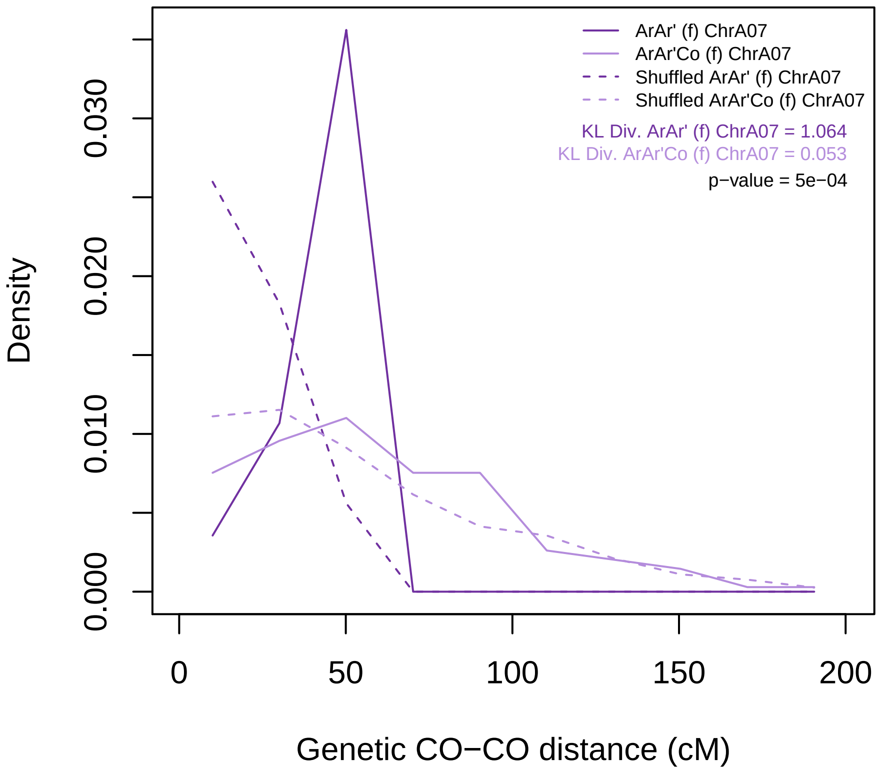

ArAr' (f) ChrA07
ArAr'Co (f) ChrA07
Shuffled ArAr' (f) ChrA07
Shuffled ArAr'Co (f) ChrA07
KL Div. ArAr' (f) ChrA07 = 1.064
KL Div. ArAr'Co (f) ChrA07 = 0.053
p−value = 5e−04
Density
0.000
0.010
0.020
0.030
0
50
100
150
200
Genetic CO−CO distance (cM)

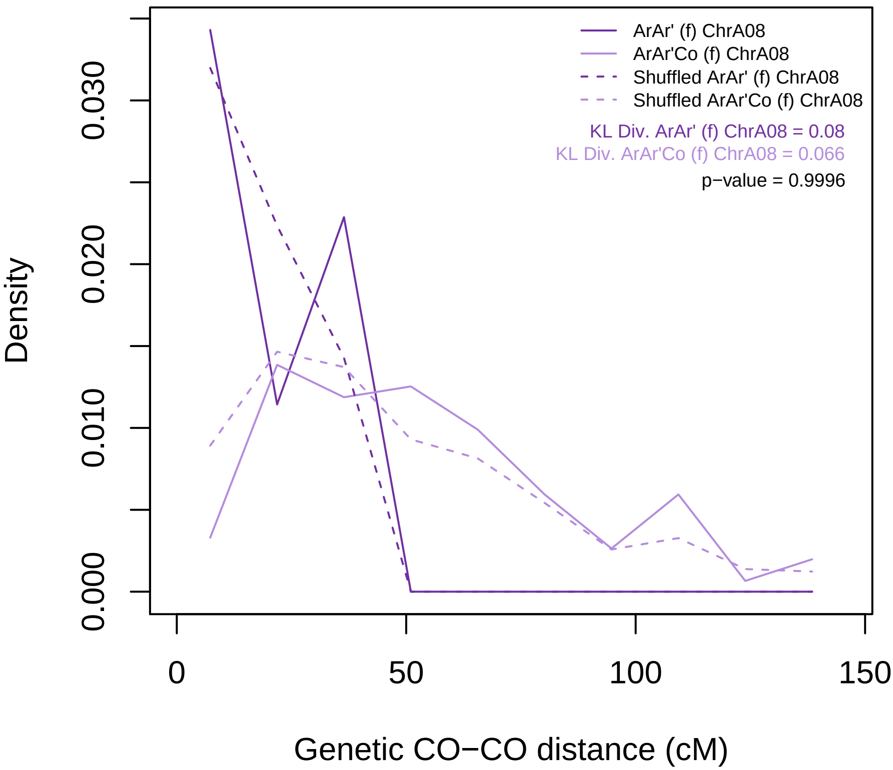

ArAr' (f) ChrA08
ArAr'Co (f) ChrA08
Shuffled ArAr' (f) ChrA08
Shuffled ArAr'Co (f) ChrA08
KL Div. ArAr' (f) ChrA08 = 0.08
KL Div. ArAr'Co (f) ChrA08 = 0.066
p−value = 0.9996
Density
0.000
0.010
0.020
0.030
0
50
100
150
Genetic CO−CO distance (cM)

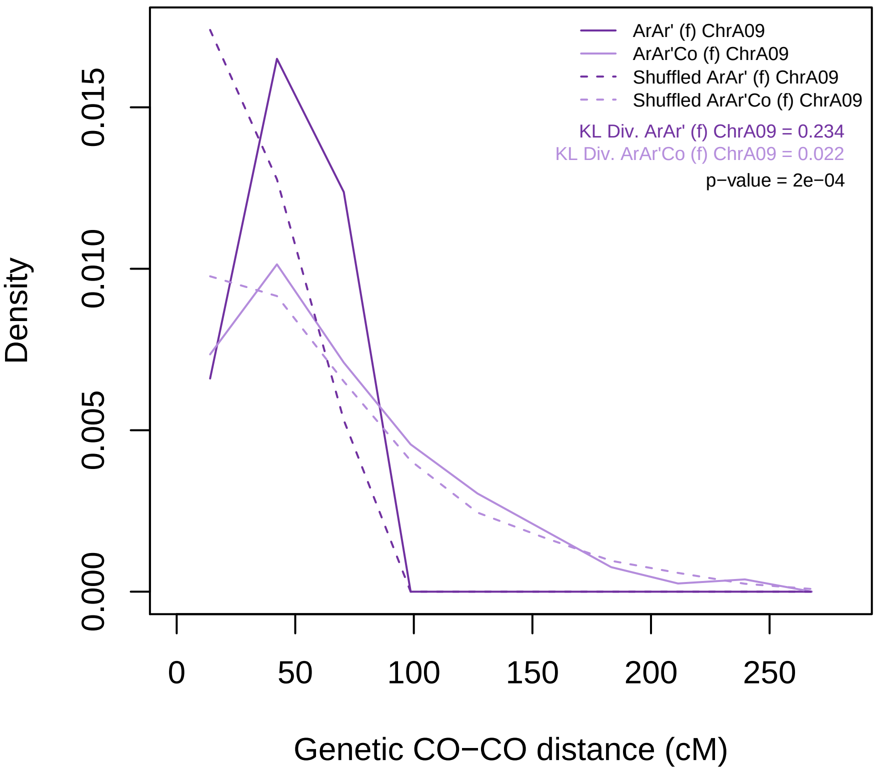

ArAr' (f) ChrA09
ArAr'Co (f) ChrA09
Shuffled ArAr' (f) ChrA09
Shuffled ArAr'Co (f) ChrA09
KL Div. ArAr' (f) ChrA09 = 0.234
KL Div. ArAr'Co (f) ChrA09 = 0.022
p−value = 2e−04
Density
0.000
0.005
0.010
0.015
0
50
100
150
200
250
Genetic CO−CO distance (cM)

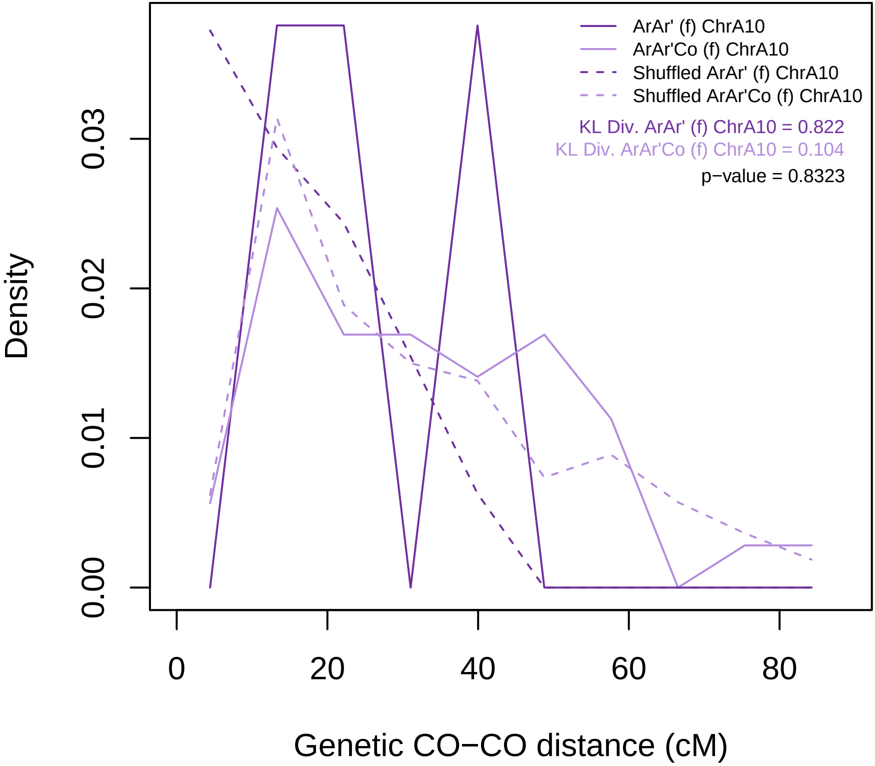

ArAr' (f) ChrA10
ArAr'Co (f) ChrA10
Shuffled ArAr' (f) ChrA10
Shuffled ArAr'Co (f) ChrA10
KL Div. ArAr' (f) ChrA10 = 0.822
KL Div. ArAr'Co (f) ChrA10 = 0.104
p−value = 0.8323
Density
0.00
0.01
0.02
0.03
0
20
40
60
80
Genetic CO−CO distance (cM)

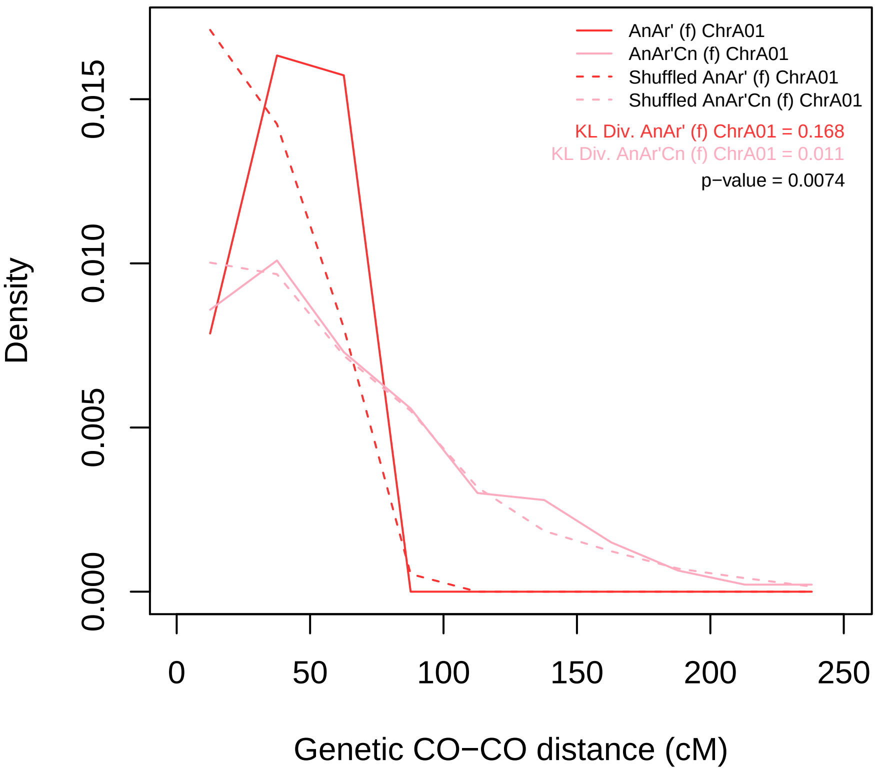

AnAr' (f) ChrA01
AnAr'Cn (f) ChrA01
Shuffled AnAr' (f) ChrA01
Shuffled AnAr'Cn (f) ChrA01
KL Div. AnAr' (f) ChrA01 = 0.168
KL Div. AnAr'Cn (f) ChrA01 = 0.011
p−value = 0.0074
Density
0.000
0.005
0.010
0.015
0
50
100
150
200
250
Genetic CO−CO distance (cM)

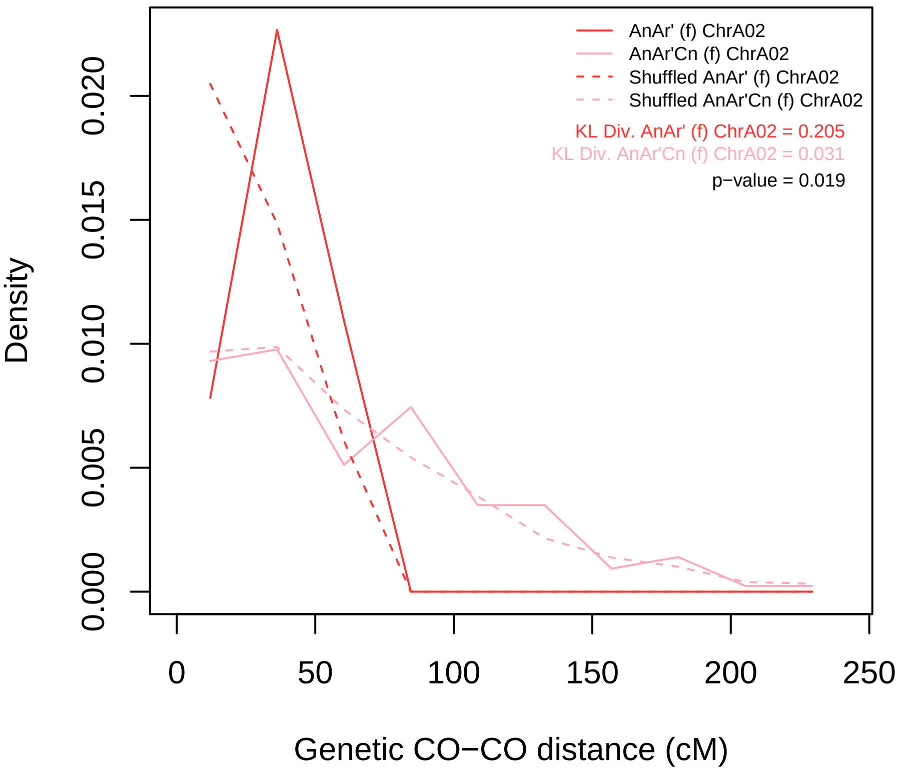

AnAr' (f) ChrA02
AnAr'Cn (f) ChrA02
Shuffled AnAr' (f) ChrA02
Shuffled AnAr'Cn (f) ChrA02
KL Div. AnAr' (f) ChrA02 = 0.205
KL Div. AnAr'Cn (f) ChrA02 = 0.031
p−value = 0.019
Density
0.000
0.005
0.010
0.015
0.020
0
50
100
150
200
250
Genetic CO−CO distance (cM)

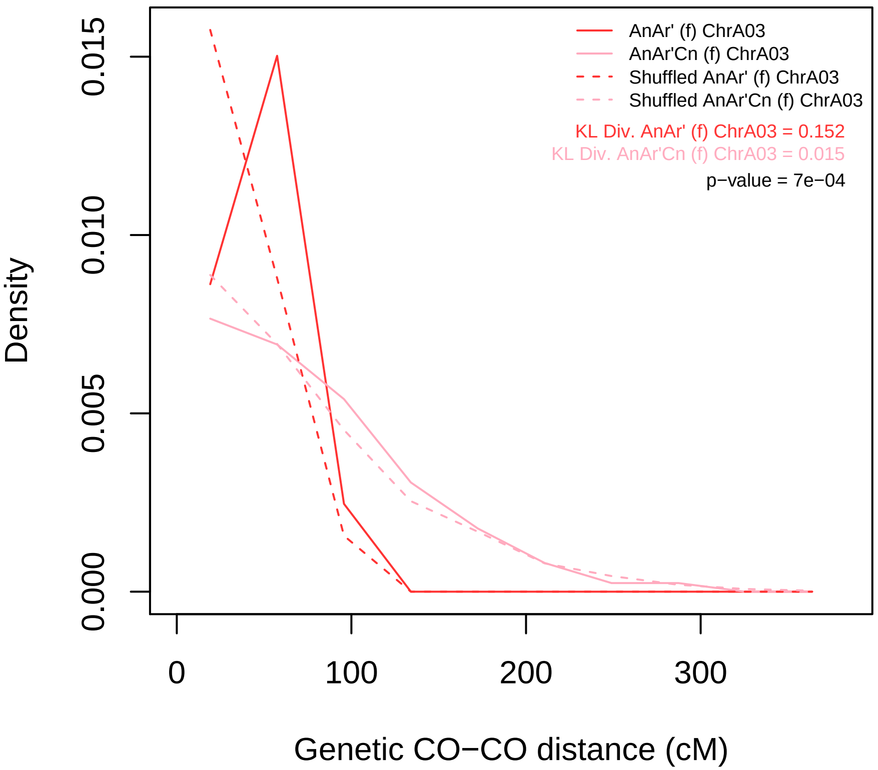

AnAr' (f) ChrA03
AnAr'Cn (f) ChrA03
Shuffled AnAr' (f) ChrA03
Shuffled AnAr'Cn (f) ChrA03
KL Div. AnAr' (f) ChrA03 = 0.152
KL Div. AnAr'Cn (f) ChrA03 = 0.015
p−value = 7e−04
Density
0.000
0.005
0.010
0.015
0
100
200
300
Genetic CO−CO distance (cM)

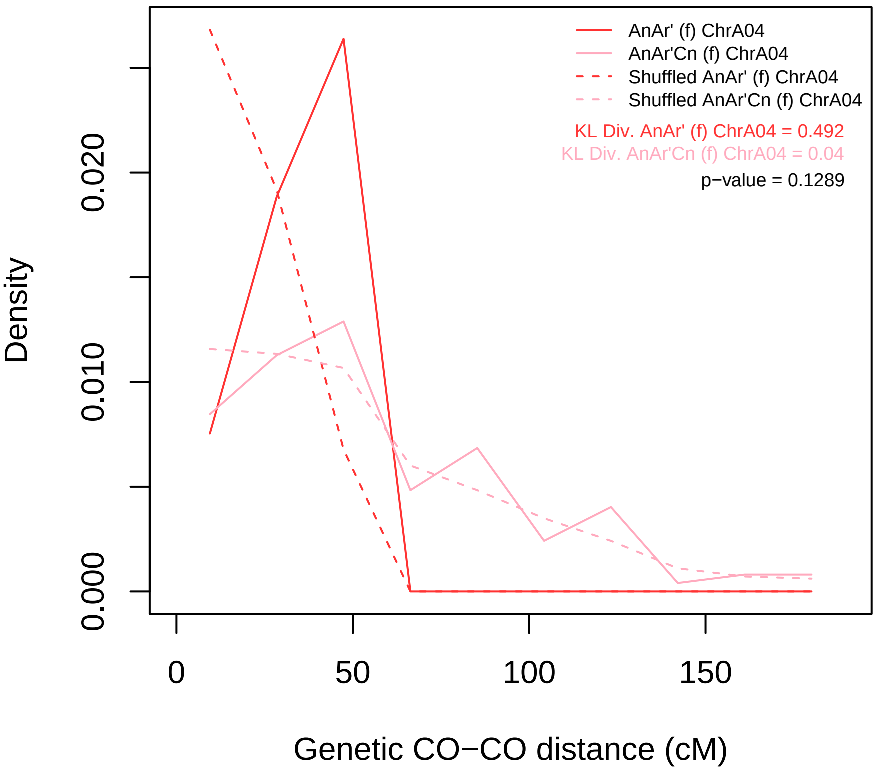

AnAr' (f) ChrA04
AnAr'Cn (f) ChrA04
Shuffled AnAr' (f) ChrA04
Shuffled AnAr'Cn (f) ChrA04
KL Div. AnAr' (f) ChrA04 = 0.492
KL Div. AnAr'Cn (f) ChrA04 = 0.04
p−value = 0.1289
Density
0.000
0.010
0.020
0
50
100
150
Genetic CO−CO distance (cM)

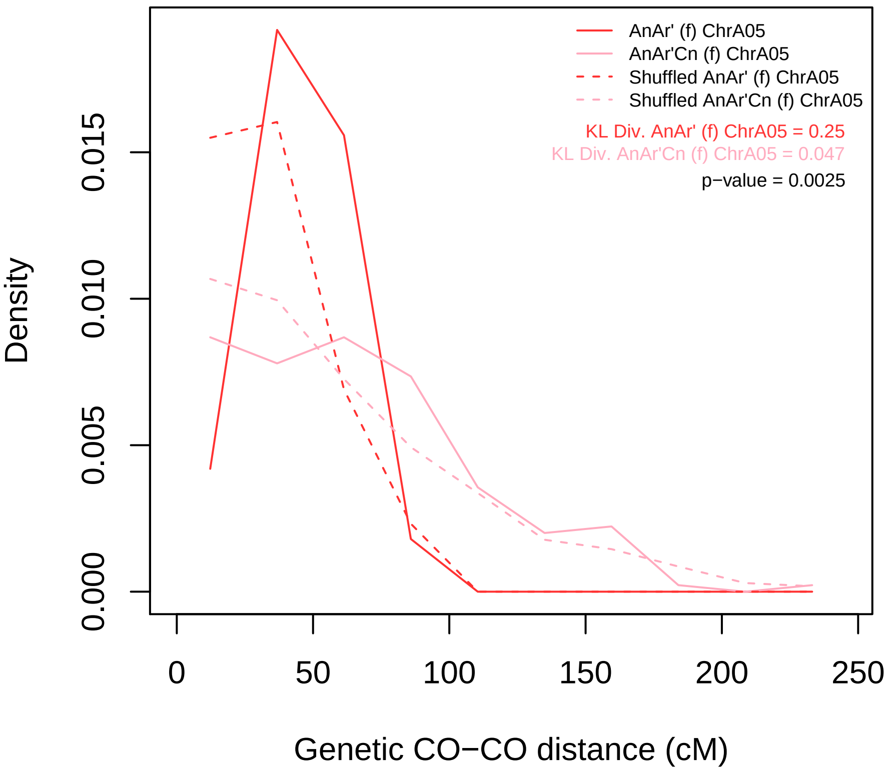

AnAr' (f) ChrA05
AnAr'Cn (f) ChrA05
Shuffled AnAr' (f) ChrA05
Shuffled AnAr'Cn (f) ChrA05
KL Div. AnAr' (f) ChrA05 = 0.25
KL Div. AnAr'Cn (f) ChrA05 = 0.047
p−value = 0.0025
Density
0.000
0.005
0.010
0.015
0
50
100
150
200
250
Genetic CO−CO distance (cM)

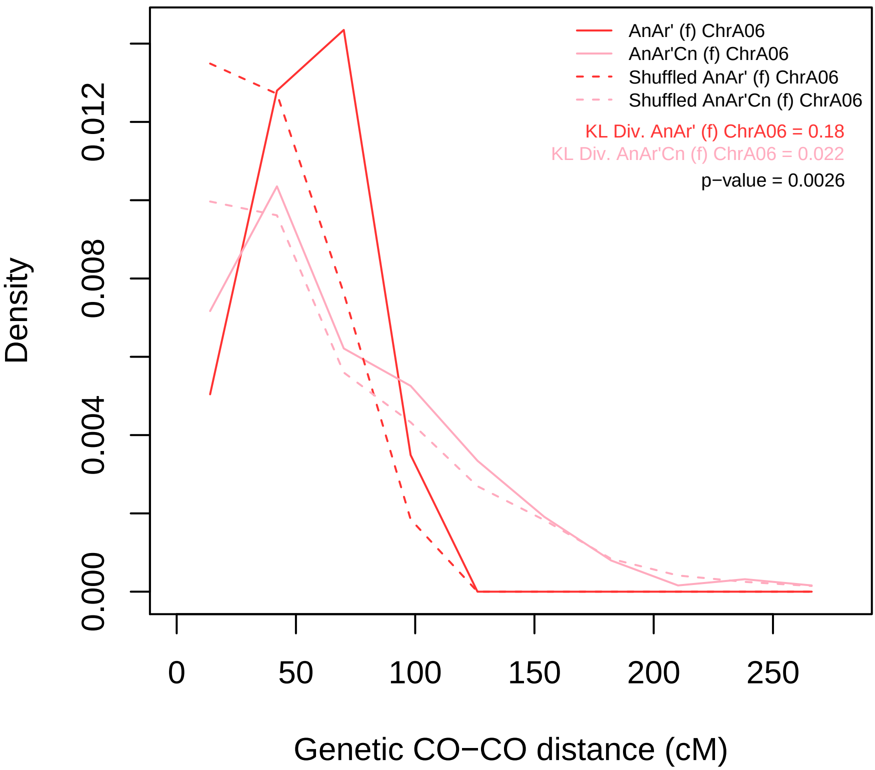
AnAr' (f) ChrA06
AnAr'Cn (f) ChrA06
Shuffled AnAr' (f) ChrA06
Shuffled AnAr'Cn (f) ChrA06
KL Div. AnAr' (f) ChrA06 = 0.18
KL Div. AnAr'Cn (f) ChrA06 = 0.022
p−value = 0.0026
Density
0.000
0.004
0.008
0.012
0
50
100
150
200
250
Genetic CO−CO distance (cM)

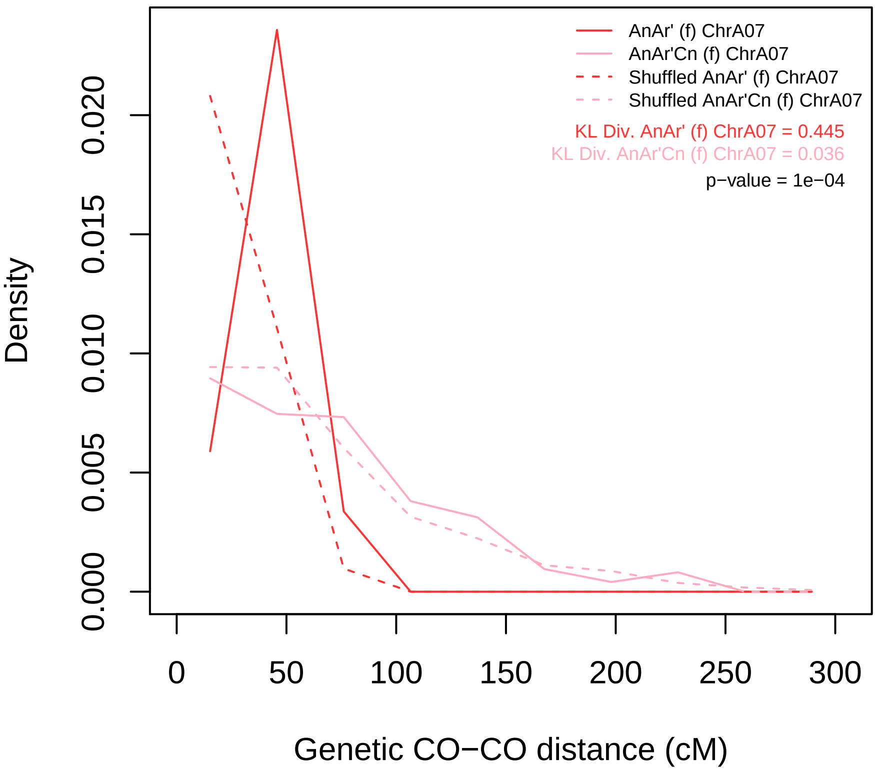

AnAr' (f) ChrA07
AnAr'Cn (f) ChrA07
Shuffled AnAr' (f) ChrA07
Shuffled AnAr'Cn (f) ChrA07
KL Div. AnAr' (f) ChrA07 = 0.445
KL Div. AnAr'Cn (f) ChrA07 = 0.036
p−value = 1e−04
Density
0.000
0.005
0.010
0.015
0.020
0
50
100
150
200
250
300
Genetic CO−CO distance (cM)

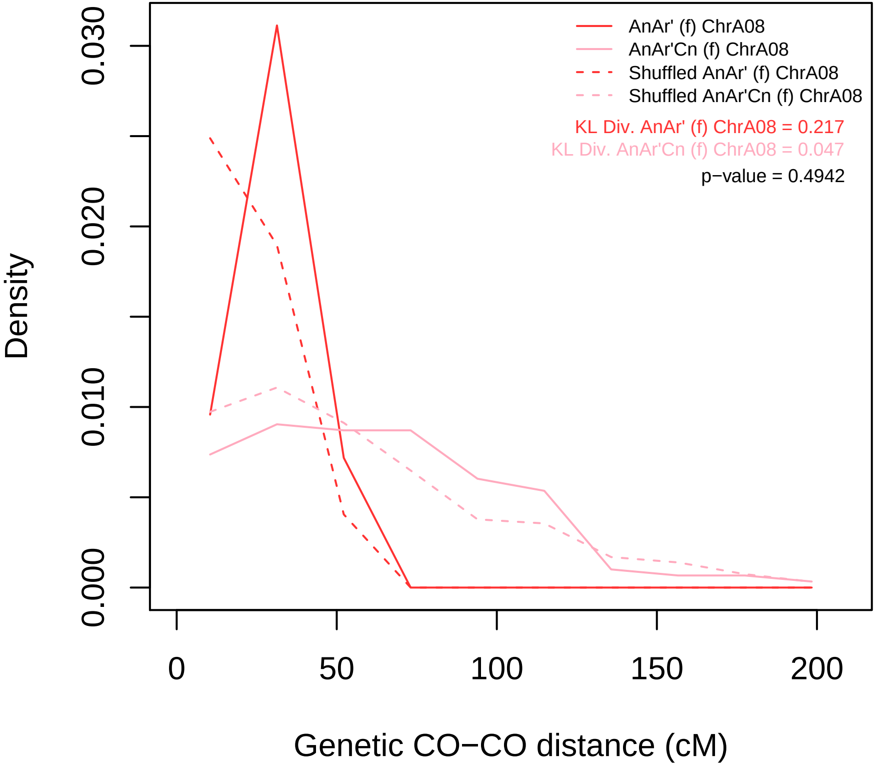

AnAr' (f) ChrA08
AnAr'Cn (f) ChrA08
Shuffled AnAr' (f) ChrA08
Shuffled AnAr'Cn (f) ChrA08
KL Div. AnAr' (f) ChrA08 = 0.217
KL Div. AnAr'Cn (f) ChrA08 = 0.047
p−value = 0.4942
Density
0.000
0.010
0.020
0.030
0
50
100
150
200
Genetic CO−CO distance (cM)

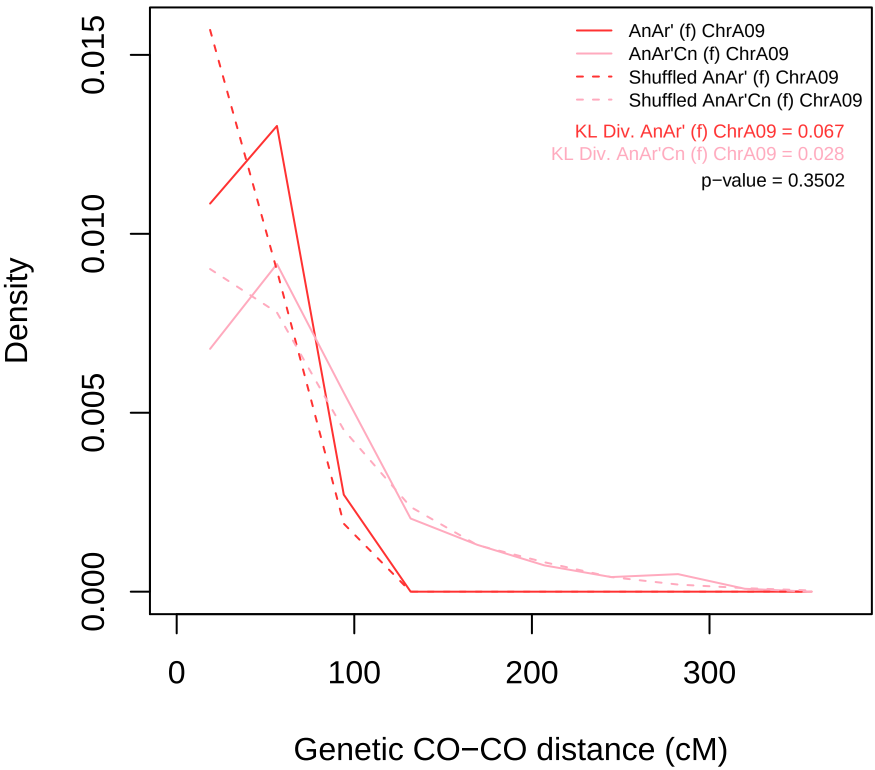

AnAr' (f) ChrA09
AnAr'Cn (f) ChrA09
Shuffled AnAr' (f) ChrA09
Shuffled AnAr'Cn (f) ChrA09
KL Div. AnAr' (f) ChrA09 = 0.067
KL Div. AnAr'Cn (f) ChrA09 = 0.028
p−value = 0.3502
Density
0.000
0.005
0.010
0.015
0
100
200
300
Genetic CO−CO distance (cM)

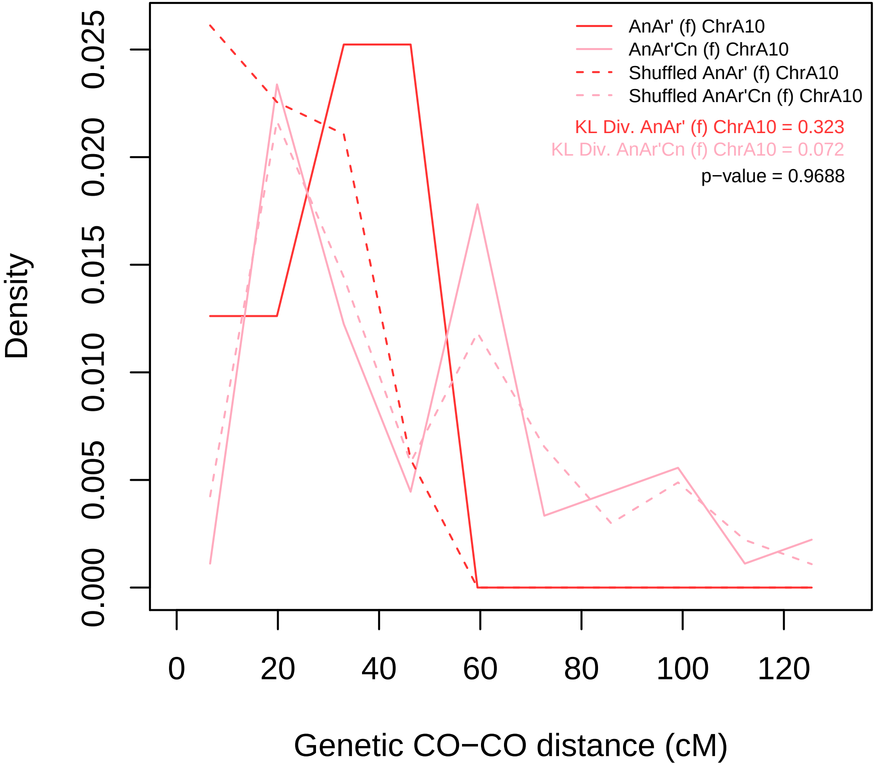
AnAr' (f) ChrA10
AnAr'Cn (f) ChrA10
Shuffled AnAr' (f) ChrA10
Shuffled AnAr'Cn (f) ChrA10
KL Div. AnAr' (f) ChrA10 = 0.323
KL Div. AnAr'Cn (f) ChrA10 = 0.072
p−value = 0.9688
Density
0.000
0.005
0.010
0.015
0.020
0.025
0
20
40
60
80
100
120
Genetic CO−CO distance (cM)

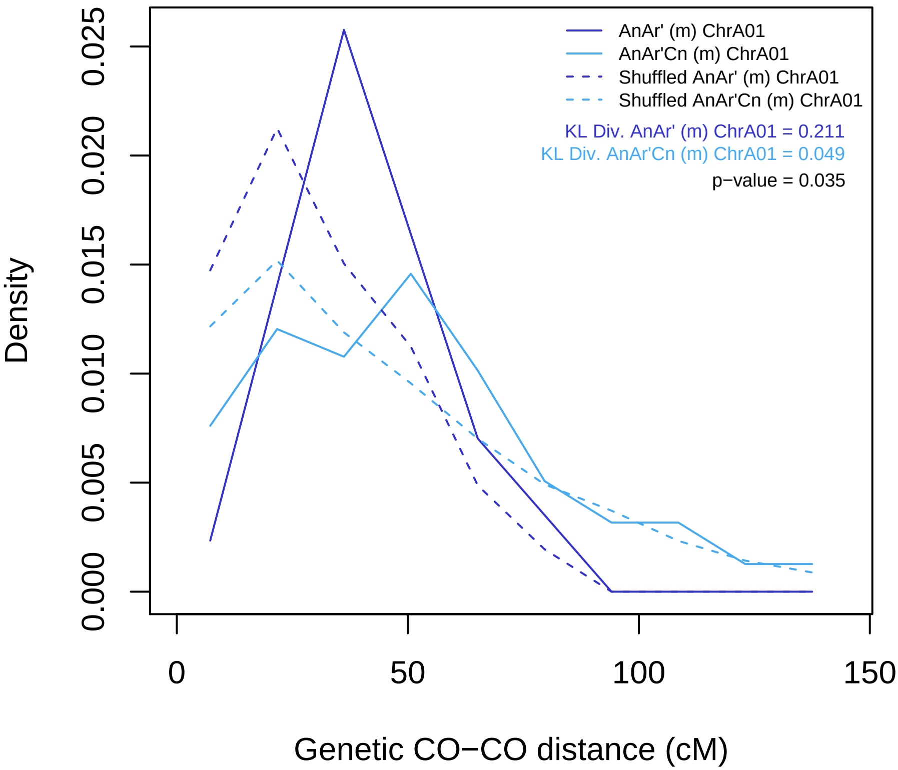

AnAr' (m) ChrA01
AnAr'Cn (m) ChrA01
Shuffled AnAr' (m) ChrA01
Shuffled AnAr'Cn (m) ChrA01
KL Div. AnAr' (m) ChrA01 = 0.211
KL Div. AnAr'Cn (m) ChrA01 = 0.049
p−value = 0.035
Density
0.000
0.005
0.010
0.015
0.020
0.025
0
50
100
150
Genetic CO−CO distance (cM)

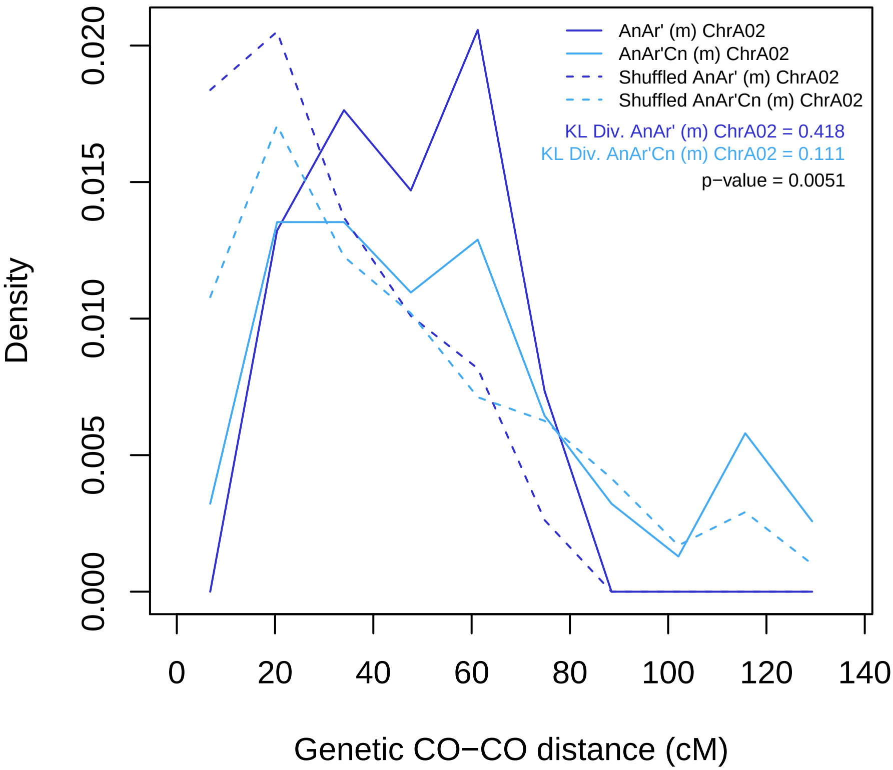

AnAr' (m) ChrA02
AnAr'Cn (m) ChrA02
Shuffled AnAr' (m) ChrA02
Shuffled AnAr'Cn (m) ChrA02
KL Div. AnAr' (m) ChrA02 = 0.418
KL Div. AnAr'Cn (m) ChrA02 = 0.111
p−value = 0.0051
Density
0.000
0.005
0.010
0.015
0.020
0
20
40
60
80
100
120
140
Genetic CO−CO distance (cM)

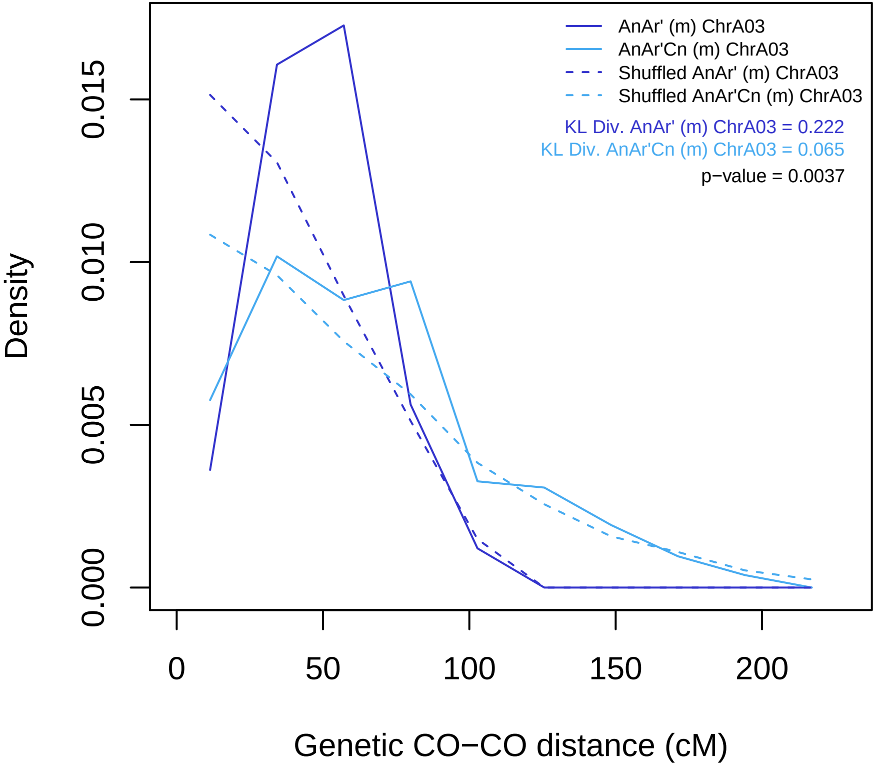

AnAr' (m) ChrA03
AnAr'Cn (m) ChrA03
Shuffled AnAr' (m) ChrA03
Shuffled AnAr'Cn (m) ChrA03
KL Div. AnAr' (m) ChrA03 = 0.222
KL Div. AnAr'Cn (m) ChrA03 = 0.065
p−value = 0.0037
Density
0.000
0.005
0.010
0.015
0
50
100
150
200
Genetic CO−CO distance (cM)

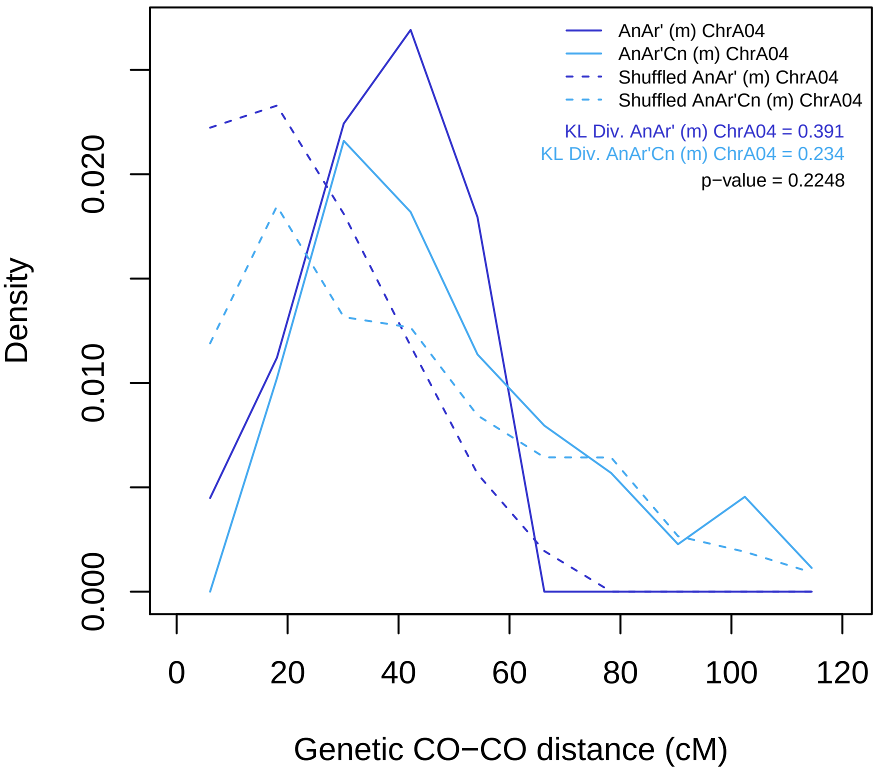
AnAr' (m) ChrA04
AnAr'Cn (m) ChrA04
Shuffled AnAr' (m) ChrA04
Shuffled AnAr'Cn (m) ChrA04
KL Div. AnAr' (m) ChrA04 = 0.391
KL Div. AnAr'Cn (m) ChrA04 = 0.234
p−value = 0.2248
Density
0.000
0.010
0.020
0
20
40
60
80
100
120
Genetic CO−CO distance (cM)

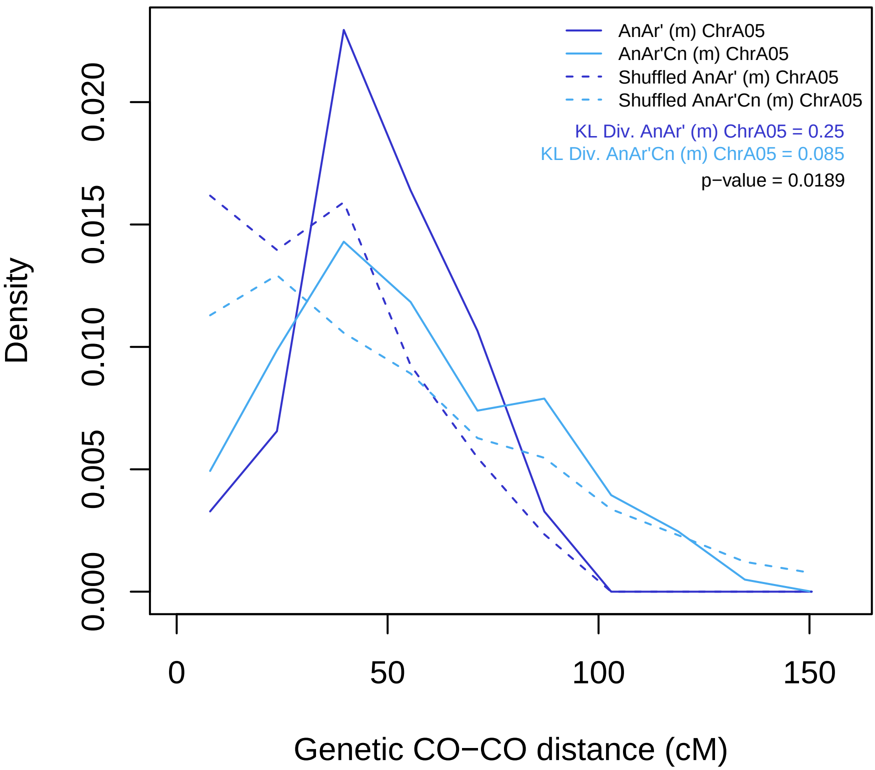
AnAr' (m) ChrA05
AnAr'Cn (m) ChrA05
Shuffled AnAr' (m) ChrA05
Shuffled AnAr'Cn (m) ChrA05
KL Div. AnAr' (m) ChrA05 = 0.25
KL Div. AnAr'Cn (m) ChrA05 = 0.085
p−value = 0.0189
Density
0.000
0.005
0.010
0.015
0.020
0
50
100
150
Genetic CO−CO distance (cM)

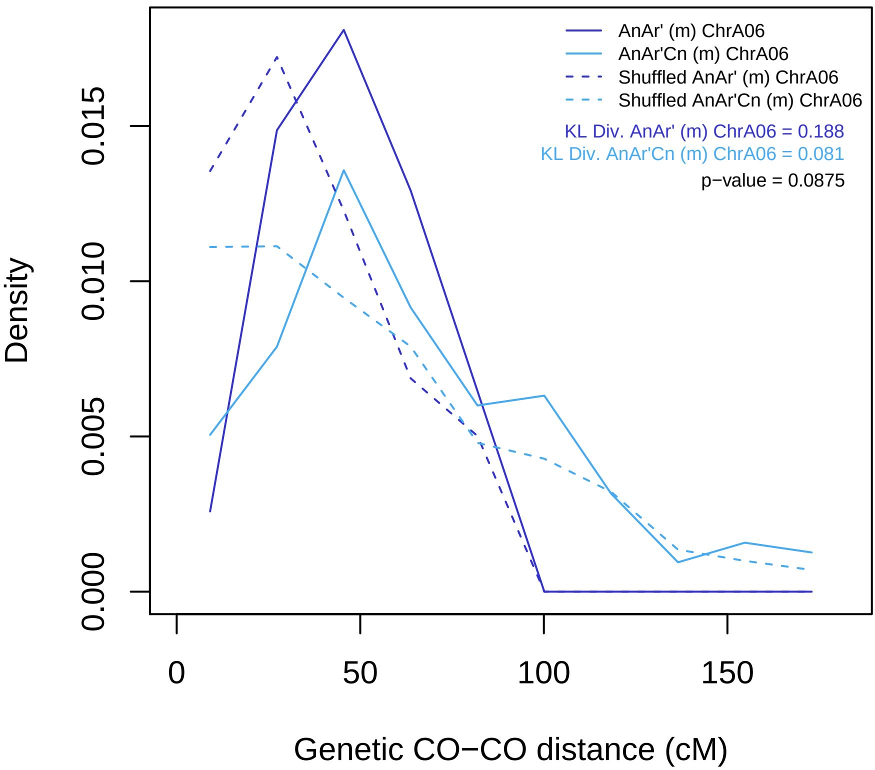

AnAr' (m) ChrA06
AnAr'Cn (m) ChrA06
Shuffled AnAr' (m) ChrA06
Shuffled AnAr'Cn (m) ChrA06
KL Div. AnAr' (m) ChrA06 = 0.188
KL Div. AnAr'Cn (m) ChrA06 = 0.081
p−value = 0.0875
Density
0.000
0.005
0.010
0.015
0
50
100
150
Genetic CO−CO distance (cM)

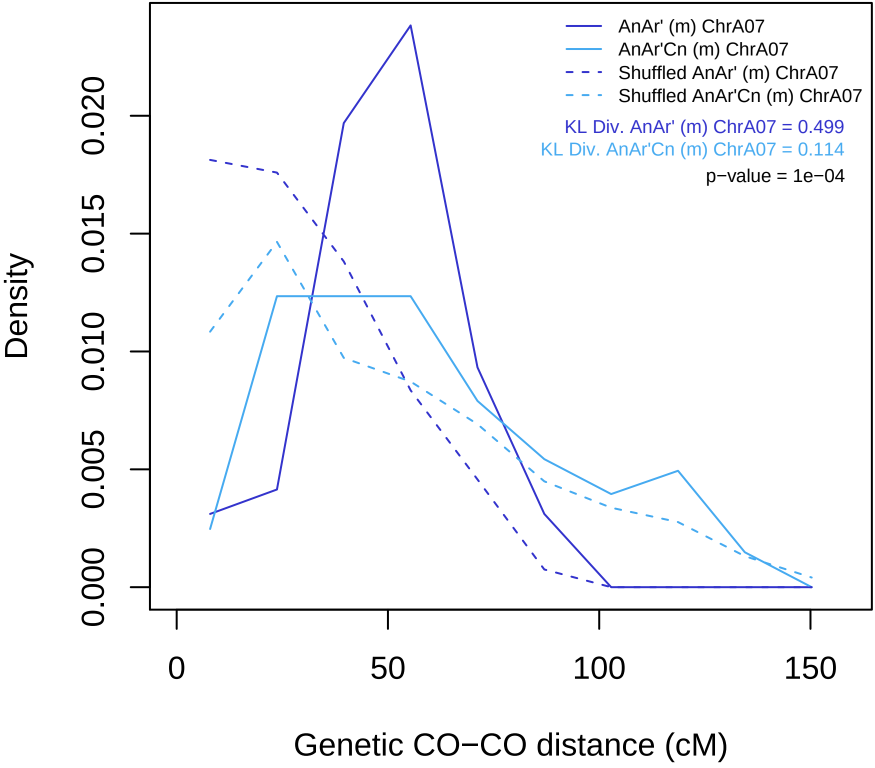

AnAr' (m) ChrA07
AnAr'Cn (m) ChrA07
Shuffled AnAr' (m) ChrA07
Shuffled AnAr'Cn (m) ChrA07
KL Div. AnAr' (m) ChrA07 = 0.499
KL Div. AnAr'Cn (m) ChrA07 = 0.114
p−value = 1e−04
Density
0.000
0.005
0.010
0.015
0.020
0
50
100
150
Genetic CO−CO distance (cM)

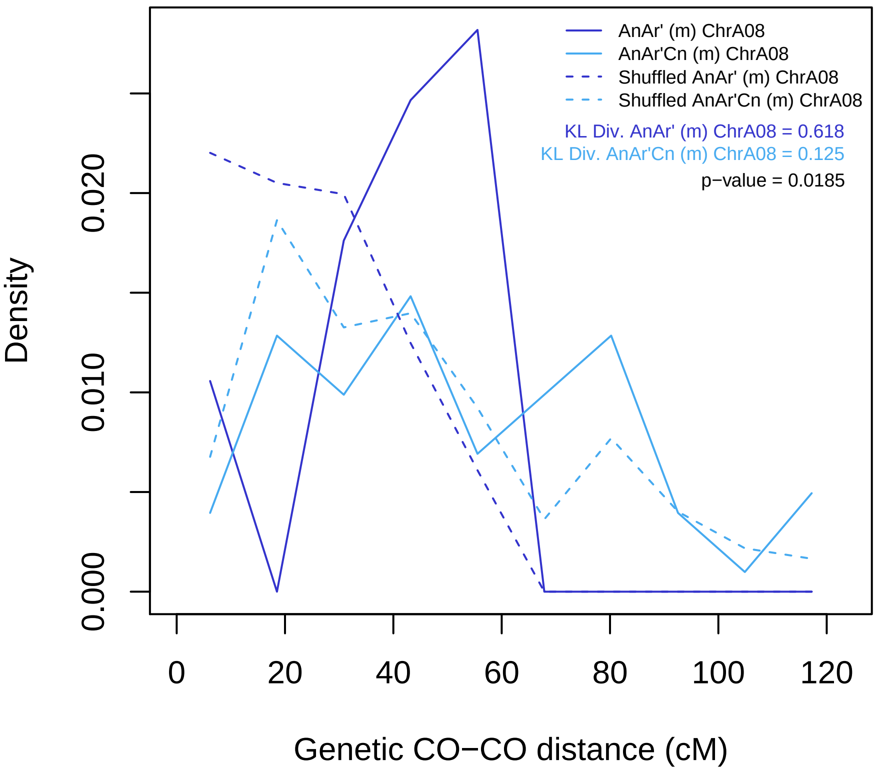

AnAr' (m) ChrA08
AnAr'Cn (m) ChrA08
Shuffled AnAr' (m) ChrA08
Shuffled AnAr'Cn (m) ChrA08
KL Div. AnAr' (m) ChrA08 = 0.618
KL Div. AnAr'Cn (m) ChrA08 = 0.125
p−value = 0.0185
Density
0.000
0.010
0.020
0
20
40
60
80
100
120
Genetic CO−CO distance (cM)

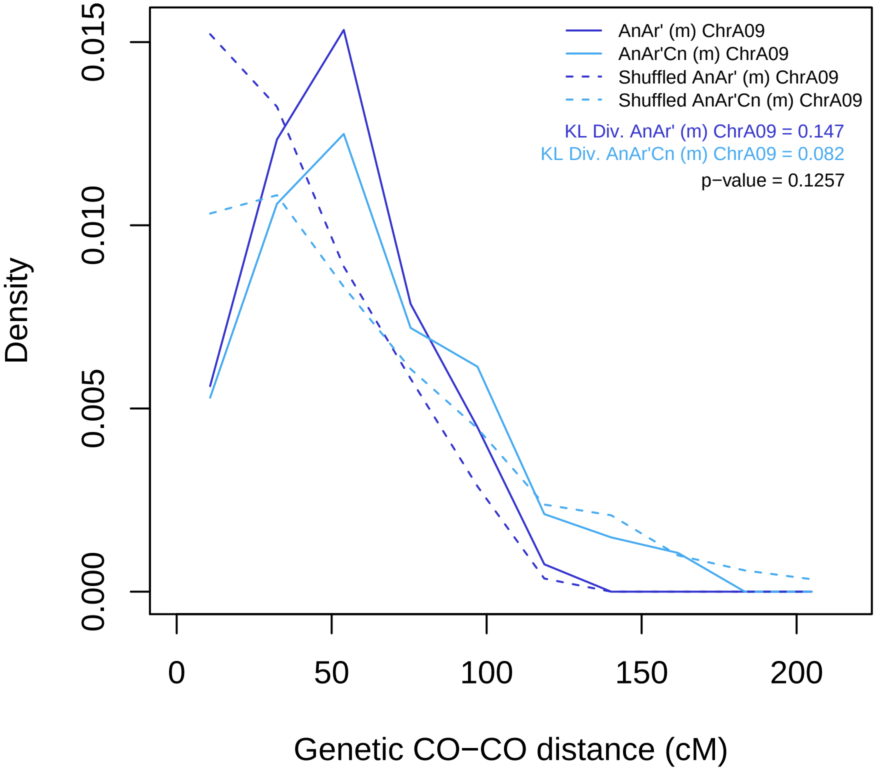

AnAr' (m) ChrA09
AnAr'Cn (m) ChrA09
Shuffled AnAr' (m) ChrA09
Shuffled AnAr'Cn (m) ChrA09
KL Div. AnAr' (m) ChrA09 = 0.147
KL Div. AnAr'Cn (m) ChrA09 = 0.082
p−value = 0.1257
Density
0.000
0.005
0.010
0.015
0
50
100
150
200
Genetic CO−CO distance (cM)

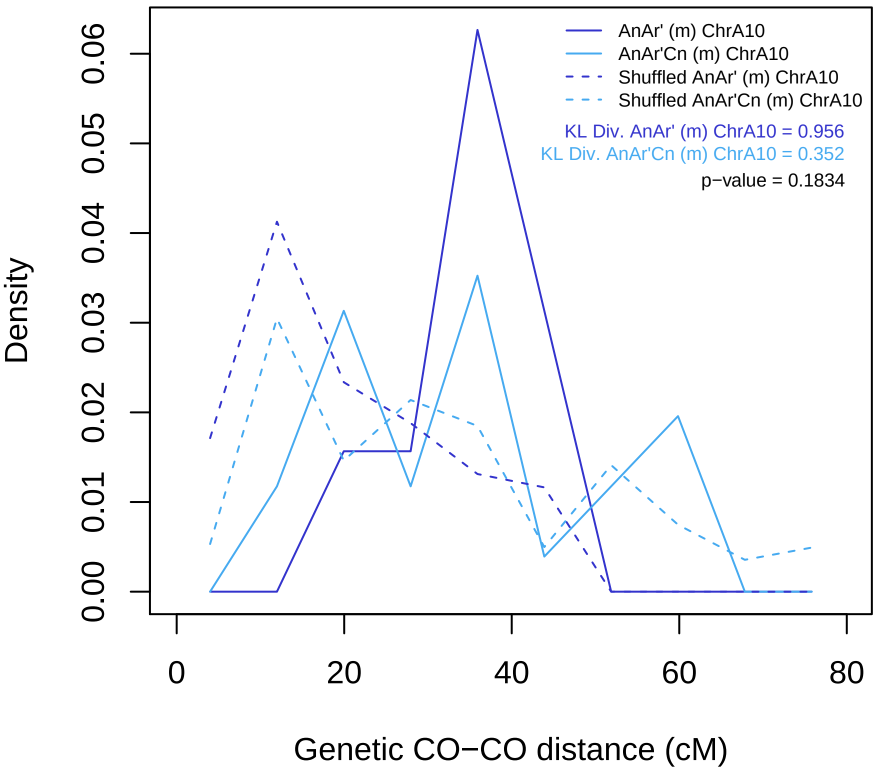

AnAr' (m) ChrA10
AnAr'Cn (m) ChrA10
Shuffled AnAr' (m) ChrA10
Shuffled AnAr'Cn (m) ChrA10
KL Div. AnAr' (m) ChrA10 = 0.956
KL Div. AnAr'Cn (m) ChrA10 = 0.352
p−value = 0.1834
Density
0.00
0.01
0.02
0.03
0.04
0.05
0.06
0
20
40
60
80
Genetic CO−CO distance (cM)
